# Transcriptomic profiles and 5-year results from the randomized CLL14 study of venetoclax plus obinutuzumab versus chlorambucil plus obinutuzumab in chronic lymphocytic leukemia

**DOI:** 10.1038/s41467-023-37648-w

**Published:** 2023-04-18

**Authors:** Othman Al-Sawaf, Can Zhang, Hyun Yong Jin, Sandra Robrecht, Yoonha Choi, Sandhya Balasubramanian, Alex Kotak, Yi Meng Chang, Anna Maria Fink, Eugen Tausch, Christof Schneider, Matthias Ritgen, Karl-Anton Kreuzer, Brenda Chyla, Joseph N. Paulson, Christian P. Pallasch, Lukas P. Frenzel, Martin Peifer, Barbara Eichhorst, Stephan Stilgenbauer, Yanwen Jiang, Michael Hallek, Kirsten Fischer

**Affiliations:** 1 University of Cologne, Faculty of Medicine and University Hospital Cologne, Department I of Internal Medicine, Center for Integrated Oncology Aachen Bonn Cologne Duesseldorf, Cologne, Germany; 2 Cancer Institute, University College London, London, UK; 3 Francis Crick Institute, London, UK; 4 Genentech Inc., South San Francisco, CA USA; 5 Roche Products Ltd, Welwyn Garden City, UK; 6 Hoffmann-la Roche, Mississauga, ON Canada; 7 Department III of Internal Medicine, Ulm University, Ulm, Germany; 8 Department II of Internal Medicine, University of Schleswig Holstein, Kiel, Germany; 9 AbbVie Inc., North Chicago, IL USA; 10 University of Cologne, Faculty of Medicine and University Hospital Cologne, Department of Translational Genomics, Cologne, Germany

**Keywords:** Chronic lymphocytic leukaemia, B-cell lymphoma, Cancer therapy

## Abstract

Data on long-term outcomes and biological drivers associated with depth of remission after BCL2 inhibition by venetoclax in the treatment of chronic lymphocytic leukemia (CLL) are limited. In this open-label parallel-group phase-3 study, 432 patients with previously untreated CLL were randomized (1:1) to receive either 1-year venetoclax-obinutuzumab (Ven-Obi, 216 patients) or chlorambucil-Obi (Clb-Obi, 216 patients) therapy (NCT02242942). The primary endpoint was investigator-assessed progression-free survival (PFS); secondary endpoints included minimal residual disease (MRD) and overall survival. RNA sequencing of CD19-enriched blood was conducted for exploratory post-hoc analyses. After a median follow-up of 65.4 months, PFS is significantly superior for Ven-Obi compared to Clb-Obi (Hazard ratio [HR] 0.35 [95% CI 0.26–0.46], *p* < 0.0001). At 5 years after randomization, the estimated PFS rate is 62.6% after Ven-Obi and 27.0% after Clb-Obi. In both arms, MRD status at the end of therapy is associated with longer PFS. MRD + ( ≥ 10^−4^) status is associated with increased expression of multi-drug resistance gene *ABCB1 (MDR1)*, whereas MRD6 (< 10^−6^) is associated with *BCL2L11* (*BIM*) expression. Inflammatory response pathways are enriched in MRD+ patient solely in the Ven-Obi arm. These data indicate sustained long-term efficacy of fixed-duration Ven-Obi in patients with previously untreated CLL. The distinct transcriptomic profile of MRD+ status suggests possible biological vulnerabilities.

## Abstract (web summary)

The CLL14 study (NCT02242942) explored the activity of obinutuzumab (anti-CD20) plus venetoclax (Bcl2 inhibitor) versus obinutuzumab plus chlorambucil in patients with previously untreated chronic lymphocytic leukemia (CLL). Here the authors report the 5-year long-term results of the clinical trial and transcriptional profiles associated with response to therapies.

## Introduction

Chronic lymphocytic leukemia (CLL) accounts for up to 30% of adult leukemia worldwide, making it one -of the most common types of blood malignancies^1^. The treatment landscape has undergone profound changes over the recent years, with targeted agents and lately also new targeted combination approaches being developed. In addition, new prognostic biomarkers have been discovered that have allowed identification of patients at risk of adverse outcomes^2–4^. Apart from certain pre-treatment genomic aberrations, such as deletion 17p [del(17p)] and/or *TP53* mutation, levels of minimal residual disease (MRD) have been understood as key surrogates for efficacy in the context of chemoimmunotherapy, but lately also of targeted therapy^5,6^.

Anti-apoptotic BCL2 signalling is a key feature of CLL that sustains proliferation and accumulation of mature B-cells^7^. Venetoclax is an oral BH3 mimetic that disrupts BCL2 signalling and ultimately leads to cell death^8^. As monotherapy, venetoclax induces remissions with undetectable MRD (uMRD) levels below 10^−4^ in approx. 30% of patients with relapsed/refractory CLL^9,10^. Outcome can be further increased when venetoclax is combined with the CD20 antibody rituximab, as seen in the Murano study, where uMRD rates of up to 64% were observed in the relapsed/refractory setting^11,12^. In the frontline setting, the CLL14 study explored the fixed-duration combination of venetoclax with the type 2 anti-CD20 antibody obinutuzumab (Ven-Obi) compared to chemoimmunotherapy of chlorambucil plus obinutuzumab (Clb-Obi) in patients with previously untreated CLL and co-existing conditions^13^. The study confirmed that 6 cycles of venetoclax-obinutuzumab combination therapy, followed by 6 cycles of venetoclax monotherapy, led to remissions with uMRD levels in 76% of patients, as assessed by ASO-PCR, compared to 35% after chlorambucil-obinutuzumab, which translated into a significant improvement of progression-free survival (PFS)^13^. However, the long-term outcomes of fixed-duration targeted therapy of CLL with regards to durability of remissions and survival is unknown. Moreover, while the majority of patients experience deep remissions at the end of treatment, a subgroup of patients showed limited or no response to treatment with detectable MRD levels; the biological drivers of MRD response or non-response (i.e. detectable MRD levels ≥10^−4^), long-term remission or early relapse, i.e. disease recurrence within one year after end of therapy, have so far not been elucidated.

Here, we report the 5-year long-term results from the randomized CLL14 study, with all patients being off-study treatment for at least 4 years, demonstrating deeper remissions and longer PFS after Ven-Obi compared with Clb-Obi. Via transcriptional profiling before start of treatment and at relapse, we demonstrate upregulation of resistance mediators like ABCB1 and increased expression of inflammatory gene sets in patients with detectable MRD at the end of Ven-Obi treatment, suggesting possible mechanisms of resistance and disease progression.

## Results

### Patients

Between 7 August 2015 and 4 August 2016, a total of 432 patients were randomized to receive either Ven-Obi (*n* = 216) or Clb-Obi (*n* = 216) (Supplementary Fig. 1). These patients constitute the intention-to-treat population used for all efficacy analyses. Baseline patient and disease characteristics are summarized in Tables 1 and 2. The median age of patients at enrolment was 72 years, median total cumulative illness rating scale (CIRS) score was 8 and median creatinine clearance was 66.3 ml/min. Sixty percent of patients had an unmutated IGHV status and 12% of patients had del(17p) and/or *TP53* mutation, with most of those patients having concomitant del(17p) and *TP53* mutation (Table 2). Sixty-four percent of patients had a high or very high-risk disease according to the CLL-International Prognostic Index (CLL-IPI), and 26% and 9% had intermediate or low-risk disease, respectively.

**Table 1:** Patient demographic and disease characteristics at baseline

Characteristic	Clb-Obi (*n* = 216)	Ven-Obi (*n* = 216)	Total (*n* = 432)
Age			
Median — yr (range)	71 (41–89)	72 (43–89)	72 (41–89)
≥75 yr — *n* (%)	78 (36.1)	72 (33.3)	150 (34.7)
Male sex — *n* (%)	143 (66.2)	146 (67.6)	289 (66.9)
Median time from diagnosis — mo (range)	29.2 (0.3–244.8)	31.2 (0.4–214.7)	30.5 (0.3–244.8)
Binet stage — *n* (%)			
A	44 (20.4)	46 (21.3)	90 (20.8)
B	80 (37.0)	76 (35.2)	156 (36.1)
C	92 (42.6)	94 (43.5)	186 (43.1)
B-symptoms present — *n* (%)^a^	112 (51.9)	103 (47.7)	215 (49.8)
Disease burden category (TLS risk category) — *n* (%)			
Low	26 (12.0)	29 (13.4)	55 (12.7)
Intermediate	147 (68.1)	139 (64.4)	286 (66.2)
High	43 (19.9)	48 (22.2)	91 (21.1)
Total CIRS score			
Median (range)	8 (1–28)	9 (0–23)	8 (0–28)
>6 — *n* (%)	177 (81.9)	186 (86.1)	363 (84.0)
Estimated creatinine clearance			
Median — ml/min (range)	67.4 (25.1–295.6)	65.2 (29.3–176.1)	66.3 (25.1–295.6)
<70 ml/min — *n* (%)	119/213 (55.9)	129/215 (60.0)	248/428 (57.9)
ECOG performance status score — *n* (%)^b^			
0	103/215 (47.9)	89/216 (41.2)	192/431 (44.5)
1	87/215 (40.5)	99/216 (45.8)	186/431 (43.2)
2	25/215 (11.6)	27/216 (12.5)	52/431 (12.1)
3	0	1/216 (0.5)	1/431 (0.2)
Serum β_2_ microglobulin			
Median — mg/l (range)	4.1 (1.2–14.2)	3.9 (1.0–11.5)	4.1 (1.0–14.2)
>3.5 mg/l — *n* (%)	128/207 (61.8)	120/202 (59.4)	248/409 (60.6)

Based on intention-to-treat population. *CIRS* Cumulative Illness Rating Scale, *ECOG* Eastern Cooperative Oncology Group, *TLS* tumour lysis syndrome.

^a^B-symptoms include the presence of fever, night sweats, significant fatigue or unintentional weight loss.

^b^ECOG performance status scores range from 0 to 5, with higher scores indicating greater disability; a score of 5 indicates death.

**Table 2:** CLL genetic characteristics at baseline

Characteristic	Clb-Obi (*n* = 216)	Ven-Obi (*n* = 216)	Total (*n* = 432)
Cytogenetic subgroups as per hierarchy^a^ — *n* (%)			
Deletion in 17p	14/208 (6.7)	17/210 (8.1)	31/418 (7.4)
Deletion in 11q	38/208 (18.3)	36/210 (17.1)	74/418 (17.7)
Trisomy in 12	40/208 (19.2)	36/210 (17.1)	76/418 (18.2)
No abnormalities	42/208 (20.2)	50/210 (23.8)	92/418 (22.0)
Deletion in 13q alone	74/208 (35.6)	71/210 (33.8)	145/418 (34.7)
IGHV mutational status — *n* (%)			
Mutated	83/208 (39.9)	76/200 (38.0)	159/408 (39.0)
Unmutated	123/208 (59.1)	121/200 (60.5)	244/408 (59.8)
Not evaluable	2/208 (1.0)	3/200 (1.5)	5/408 (1.2)
*TP53* mutational status — *n* (%)			
Mutated	19/210 (9.0)	23/211 (10.9)	42/421 (10.0)
Unmutated	191/210 (91.0)	188/211 (89.1)	379/421 (90.0)
Del(17p) and/or *TP53* mutation — *n* (%)	24/208 (11.5)	25/209 (12.0)	49/417 (11.8)
*TP53* groups — *n* (%)			
No deletion and no mutation	184/208 (88.5)	184/209 (88.0)	368/417 (88.2)
Deletion and no mutation	5/208 (2.4)	2/209 (1.0)	7/417 (1.7)
Mutation and no deletion	10/208 (4.8)	8/209 (3.8)	18/417 (4.3)
Mutation and deletion	9/208 (4.3)	15/209 (7.2)	24/417 (5.8)
CLL-IPI risk group [NEJM] — *n* (%)			
Low	19/200 (9.5)	17/187 (9.1)	36/387 (9.3)
Intermediate	55/200 (27.5)	47/187 (25.1)	102/387 (26.4)
High	118/200 (59.0)	112/187 (59.9)	230/387 (59.4)
Very high	8/200 (4.0)	11/187 (5.9)	19/387 (4.9)
Complex karyotype group — n (%)			
NCKT	167/197 (84.8)	166/200 (83.0)	333/397 (83.9)
CKT / HCKT	30/197 (15.2)	34/200 (17.0)	64/397 (16.1)

Based on intent-to-treat population. *Del* deletion, *IGHV* immunoglobulin heavy chain variable-region gene, *CLL-IPI* chronic lymphocytic leukemia international prognostic index, *CKT* complex karyotype, *NCKT* non-CKT, *HCKT* highly CKT.

^a^According to the hierarchical model of Döhner et al.^36^.

At the data cut-off on 8 November 2021, all patients had been off study treatment for at least 4 years.

### Efficacy

**Fig. 1:**
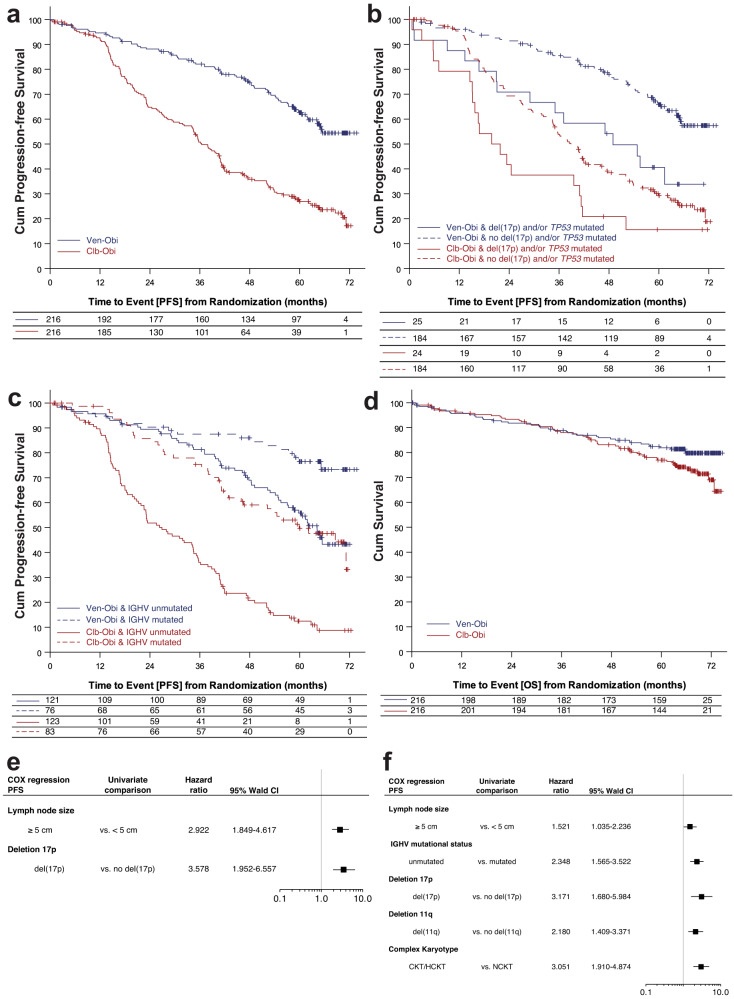
Progression-free survival (PFS) and overall survival (OS) analyses. **a** PFS according to study arm, **b** PFS according to *TP53* status, **c** PFS according to IGHV mutational status, **d** OS according to study arm, **e** multivariable analyses of PFS in Ven-Obi arm (*n* = 194) and **f** in Clb-Obi arm (*n* = 179); horizontal bars in **e** and **f** represent 95% confidence intervals of hazard ratios (indicated by squares) with the vertical lines representing a hazard ratio of 1.0.

After a median observation of 65.4 months (interquartile range [IQR] 52.6–69.4 months) and a median off-treatment duration of 54.6 months, patients in the Ven-Obi arm had a significantly longer PFS than patients in the Clb-Obi arm (Hazard ratio [HR] 0.35 [95% CI 0.26–0.46], *p* < 0.0001) (Fig. 1). Overall, 80 PFS events occurred in the Ven-Obi arm and 150 in the Clb-Obi arm. Of those PFS events, 52 were actual disease progressions (65.0% of PFS events) in the Ven-Obi arm and 132 (88.0% of PFS events) in the Clb-Obi arm. At 5-years after randomization, the estimated PFS rate was 62.6% [95% CI 55.7–69.6] in the Ven-Obi arm and 27.0% [20.6–33.4] in the Clb-Obi arm.

Patients with del(17p) and/or *TP53* mutation had a longer PFS when treated with Ven-Obi compared to Clb-Obi (5-year-PFS 40.6% vs 15.6%; HR 0.48, 95% CI 0.24–0.94) (Fig. 1b). In the Ven-Obi arm, of the 15 (20 in the Clb-Obi arm) PFS events in the del(17p) and/or *TP53* mutation group, 11 (16) were progressive disease and 4 (4) deaths (all unrelated to progressive disease). However, patients with del(17p) and/or *TP53* mutation had a shorter PFS than patients without del(17p) and/or *TP53* mutation, both in the Ven-Obi arm (5-year-PFS in patients without del(17p) and/or *TP53* mutation 65.8%; HR 2.37, 95% CI 1.34–4.17) as well as in the Clb-Obi arm (5-year-PFS in patients without del(17p) and/or *TP53* mutation 29.3%; HR 1.70, 95% CI 1.06–2.73).

Patients with an unmutated IGHV status had a significantly longer PFS in the Ven-Obi arm compared to the Clb-Obi arm (5-year PFS 55.8 vs 12.5%; HR 0.27, 95% CI 0.19–0.38). In the Ven-Obi arm, of the 55 PFS events in the unmutated IGHV group (Clb-Obi: 100 PFS events), 38 (Clb-Obi: 88) were disease progressions and 17 (Clb-Obi: 12) were deaths (all of these CLL unrelated). In both study arms, patients with unmutated IGHV status had a shorter PFS than patients with mutated IGHV status. Co-occurrence of del(17p) and/or *TP53* mutation and unmutated IGHV status was associated with the shortest PFS (Supplementary Fig. 2).

Time to next anti-leukemic treatment (TTNT) was significantly longer after Ven-Obi compared to Clb-Obi (5-year-TTNT 72.1% vs 42.8%; HR 0.42, 95% CI 0.31–0.57) (Supplementary Fig. 3). In both arms, patients with del(17p) and/or *TP53* mutation and/or unmutated IGHV status had a shorter TTNT than patients without high-risk features. Patients with del(17p) and/or *TP53* mutation had a shorter TTNT than patients without del(17p) and/or *TP53* mutation (Ven-Obi: 5-year-TTNT 48.0 vs 75.9%; HR 2.47, 95% CI 1.34–4.57; Clb-Obi: 5-year-TTNT 20.8% vs 46.7%; HR 2.23, 95% CI 1.36–3.64). BTK inhibitors were the most frequently used second-line treatment in the Ven-Obi arm (58.1%) and the Clb-Obi arm (54.3%) (Supplementary Table 1).

No significant difference in overall survival (OS) was observed between the Ven-Obi and the Clb-Obi arm (Fig. 1d). At 5 years after randomization, the estimated OS rate was 81.9% in the Ven-Obi arm and 77.0% in the Clb-Obi arm (HR 0.72, 95% CI 0.48–1.09). Of the 40 deaths in the Ven-Obi arm (57 in the Clb-Obi arm), 8 (20.0%) were related to CLL progression, i.e. associated with actual progressive CLL according to the investigator (23 [40.4%] in the Clb-Obi arm, respectively). In both arms, patients with del(17p) and/or *TP53* mutation had a shorter OS than patients without del(17p) and/or *TP53* mutation (Ven-Obi: 5-year OS 60.0% vs 85.7%; HR 2.96, 95% CI 1.44–6.09; Clb-Obi: 5-year OS 54.2% vs 80.7%; HR 2.65, 95% CI 1.39–5.04) (Supplementary Fig. 4A). While no significant difference in OS between patients with unmutated IGHV status compared to patients with mutated IGHV status was observed in the Ven-Obi arm (5-year OS 80.5% vs 86.6%; HR 1.48, 95% CI 0.73–3.03), patients with unmutated IGHV status treated in the Clb-Obi arm had a significantly shorter OS (5-year OS 70.8% vs 87.0%; HR 2.24, 95% CI 1.22–4.12) (Supplementary Fig. 4b).

For patients treated with Ven-Obi, a multivariable analysis suggested presence of deletion 17p, irrespective of *TP53* mutational status, and lymph node size ≥5 cm as independent prognostic factors for PFS (Fig. 1e), while serum β2 microglobulin and complex karyotype were independent prognostic factors for OS. For Clb-Obi-treated patients, unmutated IGHV status, deletion 11q, deletion 17p, complex karyotype and lymph node size ≥5 cm were independent adverse prognostic factors for PFS (Fig. 1F), and age ≥75 years, deletion 17p and unmutated IGHV status for OS.

### Safety

The safety population of the study, defined as patients who received at least one dose of study treatment, consisted of 212 patients in the Ven-Obi arm and 214 in the Clb-Obi arm. The median follow-up time was 66.7 months in the Ven-Obi arm and 67.6 months in the Clb-Obi arm. The majority of patients in both arms completed study treatment per protocol (Supplementary Fig. 1).

Serious adverse events (SAE) occurred in 127 (59.9%) of patients in the Ven-Obi arm and 102 (47.7%) in the Clb-Obi arm (Supplementary Tables 4, 5). The majority of adverse events (AE) was observed during the treatment phase of both study arms, whereas the rate of toxicities was reduced in the post-treatment phase (post-treatment AE rate 48.8% in the Ven-Obi arm and 29.8% in the Clb-Obi arm). In the Ven-Obi arm, 14.0% (12.2% in the Clb-Obi arm) of post-treatment AEs were deemed unrelated by the investigator (Supplementary Table 6). AE reporting (except related SAEs and secondary malignancies) was not required once a next line of treatment was started, thus fewer events might have been reported in the Clb-Obi arm due to more frequent second line therapies; see Supplementary Table 7.

Second primary malignancies were observed in 27 (12.7%) of patients in the Ven-Obi arm and 16 (7.5%) in the Clb-Obi arm (Supplementary Table 8). Twelve-month and 24-month cumulative incidence was 2.4% and 7.2%, respectively, in the Ven-Obi arm; in the Clb-Obi arm the cumulative incidences were 0.5% and 3.9%, respectively, with no significant difference in the cumulative incidence (*p* = 0.074) (Supplementary Fig. 5). The most frequent malignancies were solid tumours (including melanoma in 8 [3.8%] and 3 [1.4%] patients, respectively) and various other solid tumours (in 15 [7.1%] and 10 [4.7%] patients, respectively). Three cases of secondary haematological malignancies were reported in the Ven-Obi arm (one case of T-cell lymphoma, two cases of myelodysplastic syndrome) and two cases in the Clb-Obi arm (one case of acute myeloid leukemia, one case of plasma cell myeloma). Four patients died due to secondary malignancies in the Ven-Obi arm and 8 in the Clb-Obi arm (Supplementary Table 9).

### Minimal residual disease

**Fig. 2:**
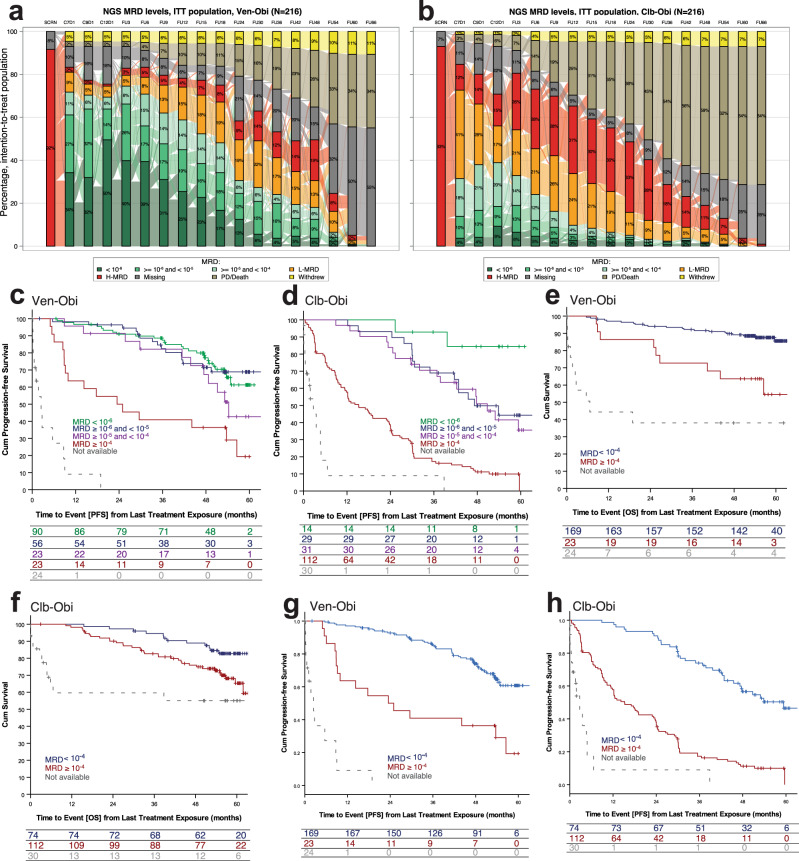
Minimal residual disease (MRD) status and outcomes. **a** Longitudinal MRD assessments by NGS in peripheral blood in the Ven-Obi arm and **b** in the Clb-Obi arm, **c** landmark PFS according to MRD status in the Ven-Obi arm and **d** in the Clb-Obi arm, **e** landmark OS according to MRD status in the Ven-Obi arm and **f** in the Clb-Obi arm, **g** landmark PFS according to MRD status in the Ven-Obi arm and **h** in the Clb-Obi arm; ‘Not available’ indicates patients without an end of treatment MRD status, due to earlier withdrawal or missing/unevaluable samples.

Two months after treatment completion (follow-up month 3), higher rates of uMRD (MRD < 10^−^^4^) in peripheral blood measured by next-generation sequencing (NGS), were observed with Ven-Obi compared to Clb-Obi (74.5% vs 32.9% of the intention-to-treat population) (Fig. 2a, b). Very deep MRD remissions below 10^−5^ and 10^−6^ were more frequent in patients after Ven-Obi therapy compared to Clb-Obi therapy (66.2% vs 19.0%, and 39.8% versus 6.5%, respectively). Forty-seven months after treatment completion (follow-up month 48), 39 (18.1%) patients in the Ven-Obi arm had maintained MRD levels below 10^−4^ compared to 4 (1.9%) in the Clb-Obi arm. Of those patients with available samples at follow-up month 48, 39/108 (36.1%) had MRD levels <10^−4^ in the Ven-Obi arm and 4/41 (9.8%) in the Clb-Obi arm (Supplementary Fig. 6). The median time to MRD conversion, i.e. an increase in patients with MRD levels <10^−4^ at end of treatment to MRD levels ≥10^−4^, was 21.1 months in the Ven-Obi arm and 6.0 months in the Clb-Obi arm (HR 0.36, 95% CI 0.26–0.48) (Supplementary Fig. 7).

A landmark analysis from last treatment exposure showed that within the CLL14 study population, patients with MRD < 10^−6^ at end of treatment had a longer PFS than patients with detectable MRD (Ven-Obi: HR 0.25, 95% CI 0.14–0.47; Clb-Obi: HR 0.06, 95% CI 0.02–0.25). Patients in the Clb-Obi arm with MRD < 10^−6^ at the end of therapy had a longer PFS than patients with MRD ≥ 10^−5^ and <10^−4^ (HR 0.22, 95% CI 0.05–0.94), although only 14 patients reached MRD < 10^−6^. PFS of patients with MRD < 10^−6^ after Ven-Obi was not longer than patients with MRD ≥ 10^−5^ and <10^−4^ (HR 0.60, 95% CI 0.30–1.21). No PFS difference between MRD < 10^−6^ and MRD ≥ 10^−5^ and <10^−4^ levels was observed in the Ven-Obi arm (HR 1.04, 95% CI 0.55–1.96) or in the Clb-Obi arm (HR 0.25, 95% CI 0.06–1.10) (Fig. 2c, d). In the Ven-Obi arm, 4 years after last treatment exposure, patients with MRD < 10^−6^ had a PFS rate of 77.1%, compared to 36.4% for patients with detectable MRD ( > 10^−4^) and 67.3% in patients with MRD ≥ 10^−5^ and <10^−4^. End of treatment MRD levels were also significantly associated with OS: Patients with MRD + status (MRD ≥ 10^−4^) at the end of therapy had a significantly shorter OS than patients with MRD ≥ 10^−5^ and <10^−4^(Ven-Obi: 4-year OS rate 63.6% vs 89.2%; HR 3.89, 95% CI 1.78–8.49; Clb-Obi: 4-year OS rate 75.9% vs 88.9%; HR 2.17, 95% CI 1.12–4.19) (Fig. 2e, f). Similar associations were seen with PFS (Fig. 2g, h).

### Transcriptomic profiles

Pre-treatment RNAseq data from CD19-enriched peripheral blood samples, i.e. enriched for the CLL cell fraction, were available for 405 of the 432 randomized patients (203 Ven-Obi, 202 Clb-Obi). RNAseq data from CD19-enriched peripheral blood samples at first disease relapse were available for 29 of 52 relapsed patients in the Ven-Obi arm (Supplementary Table 10); nodal relapses without peripheral lymphocytosis or missing sample collection caused limited sample availability at relapse. For the control arm, 16 of 132 representative relapses (early and late relapses [<1 year, ≥1 year after end of therapy]), mutated and unmutated IGHV and *TP53* status) in the Clb-Obi arm were manually selected (Supplementary Fig. 8). The median B-cell fraction prior to CD19-enrichment was 97% and RNAseq deconvolution confirmed high purity after CD19-enrichment (see Methods).

**Fig. 3:**
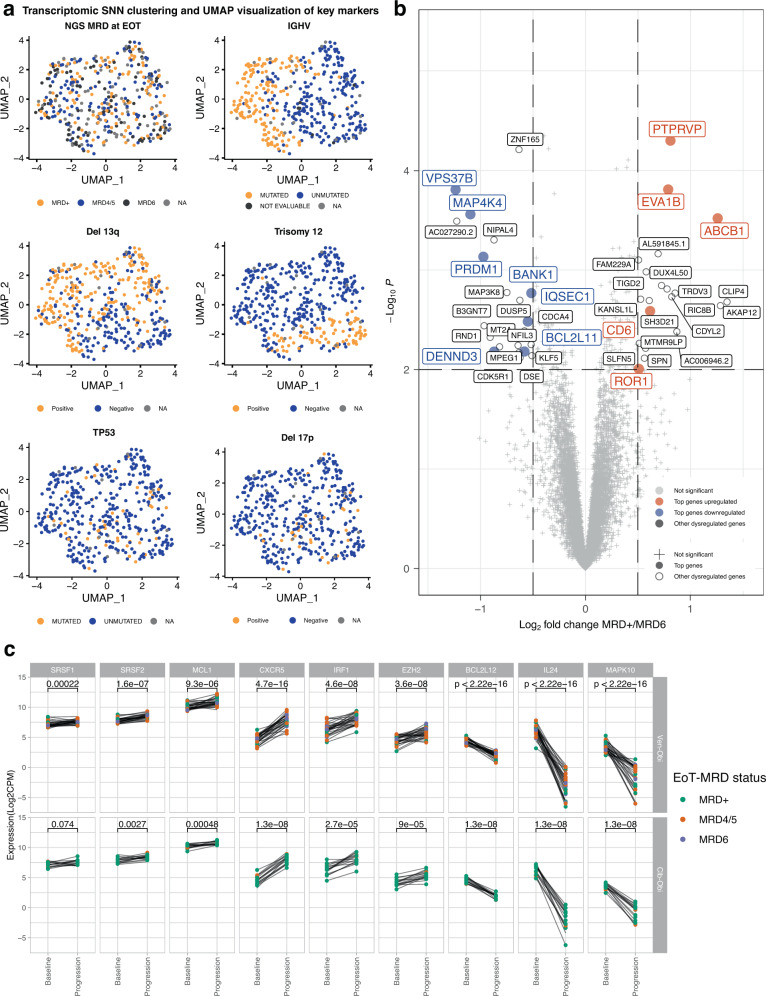
Differential gene expression according to minimal residual disease status and baseline versus relapse status. **a** UMAP (Uniform Manifold Approximation and Projection) visualization of shared nearest neighbour (SNN) clustering before treatment, with MRD, IGHV, deletion 17p, *TP53*, deletion 13q, and trisomy 12 status overlaid. **b** Pre-treatment differential gene expression between MRD6 vs MRD +, gene previously associated with CLL pathology are highlighted; dashed lines indicate significance cut-offs. *P*-values from moderated two-sided t-test without adjusting for multiple testing. **c** Paired patient-level comparison of differentially expressed genes at baseline and relapse (‘progression’) according to Ven-Obi arm (upper row) and Clb-Obi arm (lower row), *n* = 44, *p*-values from two-sided Wilcoxon signed-rank test without adjusting for multiple testing. Source data of differential gene expression analyses are provided as Source Data file.

To obtain a global overview on gene expression variation in CLL within a representative, treatment-naïve patient cohort, unsupervised shared nearest neighbour (SNN) clustering was performed. Patient samples were clustered by presence of IGHV mutational status, trisomy 12 and deletion 13q (Fig. 3a). No clustering according to other factors, such as *TP53* status, MRD status and age group was observed (Figs. 3a, S9).

To explore potential factors associated with MRD response and to optimize the discovery aspect, patients from both treatment arms were grouped into MRD responders if they had very deep response to therapy (MRD6, i.e. <10^−6^) or non-responders (MRD+, i.e. MRD ≥ 10^-4^) at follow-up month 3. Prior to treatment initiation, 41 differentially expressed-genes (DEG, *p*-value <0.01 and log2 fold change >0.5) were observed (Supplementary Data 1). MRD+ status was particularly associated with higher expression of resistance markers like *ABCB1 (MDR1)*, whereas MRD6 status was associated with higher pro-apoptotic *BCL2L11* (BIM) expression (Fig. 3B). Additionally, to consider all patients with available MRD at end of therapy and RNAseq data, patients were also grouped into responders by uMRD (i.e. all patients with MRD levels <10^−4^) or non-responders (MRD+). Again, higher expression of *ABCB1* was observed in the MRD+ compared to the uMRD group (Supplementary Fig. 10).

To elucidate transcriptomic differences associated with disease recurrence, differential gene expression between pre-treatment and relapse status was analysed. Differentially upregulated genes at relapse included *CXCR5*, *IRF1* and *EZH2*, whereas *BCL2L12*, *IL24* and *MAPK10* were downregulated at relapse (Supplementary Data 2, Supplementary Fig. 11A). In addition to this pseudobulk analysis via the Seurat software (see Methods), the observation was also confirmed when looking at these genes on a patient level between paired baseline and relapse timepoints (Fig. 3c). Notably, these patterns were consistent in both the Ven-Obi arm (upper row, Fig. 3c) and the Clb-Obi arm (lower row, Fig. 3c).

**Fig. 4:**
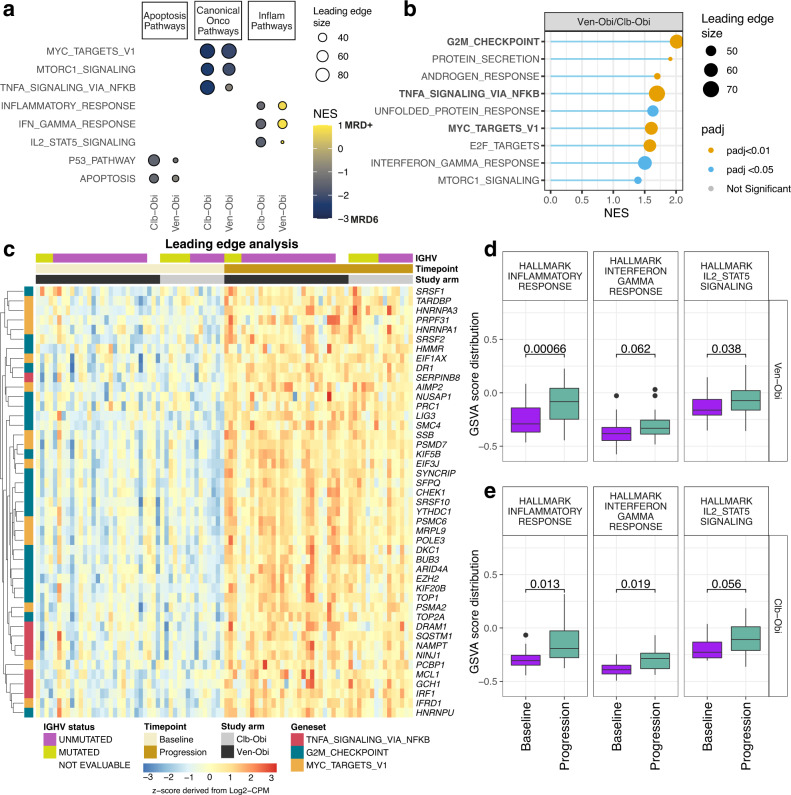
Gene set enrichment analyses according to minimal residual disease status and baseline versus relapse status. **a** Summarized pre-treatment gene set enrichment analysis of hallmark gene sets according to treatment arm and according to EOT MRD6 and MRD + status. Positive normalized enrichment scores (NES) indicates gene sets enriched in MRD + and negative NES indicates gene sets enriched in MRD6. **b** Patient-level gene set enrichment analysis of relapse versus baseline samples in both arms. Significantly enriched hallmark gene sets (adjusted *p*-value <0.05) are highlighted, positive NES indicate enrichment in relapsed samples. *P*-values derived from non-parametric permutation test and adjusted for multiple testing using the Benjamini-Hochberg procedure. **c** Leading edge analysis of baseline (left) versus relapse (right) samples based on ‘TNFa signalling via NFkB’, ‘G2M checkpoint’ and ‘MYC targets V1’ gene sets, which were significantly enriched at relapse. **d** Gene set variation analysis of selected inflammatory pathways before Ven-Obi (D, *n* = 29) or Clb-Obi (**e**, *n* = 15) treatment (baseline) and at relapse (progression); two-sided Wilcoxon signed-rank test, not adjusted for multiple testing. box plots represent lower quartile, median and upper quartile, whiskers extend to a maximum of 1.5 × IQR beyond the box, points indicate outliers. NES, normalized enrichment score; MRD, minimal residual disease, EOT, end of treatment. Source data of differential gene expression analyses are provided as Source Data file.

To further explore relevant biological differences between MRD responders and non-responders, a gene set enrichment analysis (GSEA) was conducted based on 50 hallmark gene sets. Irrespective of treatment arm, MRD6 was associated with apoptotic pathways (p53 and apoptosis) and canonical oncogenic pathways (MYC, mTORC1, TNFα/NFkB) (Fig. 4a). In contrast, treatment-specific association with three inflammatory gene sets (inflammatory response, IFNγ response, IL2/STAT5) was observed, which were consistently enriched in MRD+ patients specifically in Ven-Obi arm, but not in the Clb-Obi arm. This suggests a possible specific role of the inflammatory pathways in impaired MRD response to BCL2 inhibitors.

Next, the GSEA was repeated with respect to relapse versus baseline samples and enrichment of oncogenic pathways in relapsed samples was observed, including cellular proliferation, (G2M checkpoint, MYC targets) as well as inflammatory signalling (TNFα signalling via NFκB) in both study arms (Fig. 4b, Supplementary Fig. 11B). A leading-edge genes analysis of these gene sets indicated 45 differentially expressed genes (*p*-value <0.01 and log2-fold change >0.5), which included various RNA splicing factors such as *SRSF1* and *SRSF2* as well as apoptotic regulators like *MCL1* (Fig. 4c).

The association of inflammatory signalling with disease relapse was further corroborated on an individual patient level, where a gene set variation analysis (GSVA) demonstrated enrichment of inflammatory pathways at relapse compared to baseline for both the Ven-Obi arm (Fig. 4d) and the Clb-Obi arm (Fig. 4e). By generating GSVA scores for all sequenced baseline samples, patients were classified into a group with high (upper 20th GSVA score percentile) and a group with low (lower 80th GSVA score percentile) inflammatory response (Supplementary Table 12). In patients with high inflammatory response del(17p) and/or *TP53* mutation as well as trisomy 12 was more common (18% versus 11% and 32% versus 15%, respectively), whereas deletion 11q was less common (11% versus 20%) than in patients with low inflammatory response enrichment (Supplementary Table 11). The frequency of infections was similar in both groups (Supplementary Table 12).

In summary, distinct gene expression profiles were observed in patients with and without deep MRD response to venetoclax-based therapy. Patients with detectable MRD at the end of Ven-Obi treatment showed upregulation of resistance mediators like *ABCB1* and increased expression of inflammatory gene sets. Relapsed cases were also characterized by upregulation of inflammatory as well as oncogenic pathways at the time of relapse, suggesting possible mechanisms of resistance and disease progression that warrant further exploration.

## Discussion

The venetoclax-obinutuzumab regimen is the first fixed-duration frontline treatment without chemotherapy in CLL. Long-term observations are therefore of high interest, since they allow to gain an understanding of disease dynamics and clinical outcomes after cessation of therapy. In particular, the biological characteristics and determinants of MRD response and non-response to limited exposure to BCL2 inhibition have not been elucidated so far.

The first aim of this report was to analyse the long-term efficacy and durability of a fixed-duration regimen with Ven-Obi, compared to chemoimmunotherapy with Clb-Obi. All patients were off study treatment for at least 4 years. The majority of patients who had received Ven-Obi have remained in remission 4 years after treatment completion, suggesting possible achievement of long-term disease control in this group of elderly and unfit patients. In addition, over 70% of the patients in the Ven-Obi arm have not yet required another line of treatment suggesting that many patients might only require one line of CLL treatment in their lifetime. Furthermore, all treatment-related toxicity occurred during treatment exposure, with no long-term toxicity observed after end of treatment, concluding the fixed-duration approach was associated with reduced toxicity. This finding might avoid issues of cumulative toxicities observed with continuous treatment exposure^14^. The cumulative incidence of second primary malignancies was not statistically different between the two treatment arms at this point, but will continue to be closely monitored.

Patients with del(17p) and/or *TP53* mutation had a better outcome when receiving Ven-Obi compared to Clb-Obi; however, *TP53* status remains an adverse prognostic factor in both study arms, i.e. patients who carried *TP53* deletions or mutations had a significantly shorter PFS than patients without del(17p) and/or *TP53* mutation. Likewise, patients with unmutated IGHV status had a significantly shorter PFS than patients with a mutated IGHV status, however this was not found to be an independent prognostic factor for PFS. The multivariable analysis suggested that del(17p), but not *TP53* mutation, were independently associated with PFS for patients treated with Ven-Obi, however, the number of patients with isolated *TP53* mutation was overall limited. This difference was particularly driven by patients who had both, del(17p) and/or *TP53* mutation and unmutated IGHV status, indicating that patients with co-occurrence of these factors are at highest risk of early relapse.

While almost all patients in the Clb-Obi arm had experienced an MRD conversion to MRD levels ≥10^−4^ four years after treatment completion, a fifth of patients still had uMRD in the Ven-Obi arm. This finding indicates a subsequent re-growth of MRD after treatment cessation, which occurs slower in the Ven-Obi arm than in the Clb-Obi a﻿rm^15,16^. In particular, MRD conversion time in patients treated with Ven-Obi was significantly longer with a median of 21.1 months compared to 6.0 months in the Clb-Obi arm. While patients in the Ven-Obi arm more frequently reached very deep MRD levels of <10^−6^, no clear association with longer PFS was observed so far. Hence, the prognostic value of <10^−6^ versus shallower <10^−5^ or <10^−4^ still needs to be elaborated with longer follow-up in the first line setting. While the majority of patients in the Ven-Obi arm remained in remission according to iwCLL definition, the fact that only a minority of patients had maintained MRD < 10^−6^ at the current follow-up indicates that the regimen does not provide complete MRD eradication in most patients.

In light of the strong prognostic impact of follow-up month 3 MRD status, the transcriptional profiles of patients before treatment were classified into MRD responders and non-responders. Clustering according to gene expression showed that patients were grouped according to IGHV status, trisomy 12 and also deletion 13q, which has also been previously observed in CLL^17,18^, but not to MRD response or non-response. This finding suggests that individual pathways and genes, rather than global differences are associated with MRD response. High expression of BCL2L11 (*BIM)*, a pro-apoptotic regulator, prior to treatment was particularly associated with deep remissions <10^−6^, suggesting that BCL2 inhibition is particularly effective in this setting. In contrast, inflammatory pathways were upregulated in non-responders to therapy and notably also in patients who relapsed after Ven-Obi treatment, suggesting that inflammatory signalling might be associated with limited MRD response to venetoclax therapy. This was also seen in relapsed disease, where TNFα and NFκB associated pathways were upregulated; in addition, regulators of RNA splicing, such as *SRSF1*, and apoptosis, such as *MCL1*, were higher expressed than at pre-treatment, indicating a possible biological role in driving CLL progression after therapy. Of note, with the caveat of a limited number of sequenced Clb-Obi cases, upregulation of oncogenic pathways was observed in both treatment arms at relapse, which suggests a driving role of these pathways in both treatment contexts. The landscape of somatic mutations and copy number profiles of untreated CLL have been widely studied previously^19,20^. In the context of continuous venetoclax treatment, heterogeneous selection of mutations (e.g. *BCL2*, *BTG1*, *CDKN2A*/B, *BRAF*) and amplifications (e.g. PD-L1) have been previously described^21–23^. We have previously reported a lack of subclonal *BCL2* mutations after frontline Ven-Obi and similar findings were observed after Ven-Rituximab in relapsed/refractory CLL^24^. Ongoing studies will eventually provide a deeper understanding of mutational and copy number profiles after time-limited venetoclax exposure.

An open question is how the fixed-duration approach generally compares to the other cornerstone of CLL management, which is continuous BTK inhibitor therapy^25–28^. Depending on the patient population, studies have reported comparable 4-year PFS rates with continuous ibrutinib or acalabrutinib of 75 to 80%^29–31^. Head-to-head comparisons between continuous and fixed-duration treatment regimens are ongoing to identify which groups of patients benefit most from those paradigms (e.g. CLL17/NCT04608318). In addition, dedicated randomized studies that explore the long-term benefit of MRD-guided treatment extension or intensification compared to continuous or fixed-duration regimens are warranted, since patients with detectable post-treatment MRD levels are at high risk of shorter survival. A caveat of the present study might be the limitation to bulk, rather than single cell sequencing, which might provide additional dimensions. A selection bias within the relapse samples cannot be excluded, since some peripheral blood samples were not available at relapse as patients might have been too unwell to visit the study sites and were treated by their local physicians instead. While a link between inflammatory signalling and BCL2 and BCL-xL has been proposed in the past^32^, further functional in-vitro and in-vivo validation experiments are warranted to corroborate the role of inflammatory response signalling and response to BCL2 inhibition in CLL. Moreover, the clinical impact of aberrated inflammatory response signalling with regards to frequency of infections or autoimmune reactions in patients with CLL also requires further study. The study was limited to sequencing of CD19-enriched material and therefore focussed on the CLL fraction, but future endeavours will also allow for exploration of the myeloid and T-cell compartments before and after treatment, which was beyond the scope of this report.

In summary, these 5-year results suggest that in patients with previously untreated CLL, fixed-duration treatment with Ven-Obi continues to lead to significantly longer progression-free survival compared to chemoimmunotherapy with Clb-Obi. This finding was associated with a significant decrease in toxicity after treatment cessation and continuous deep remissions with high rates of undetectable MRD. Upregulation of inflammatory pathways and resistance markers such as *ABCB1* were associated with poorer MRD response to BCL2 inhibition, suggesting possible biological vulnerabilities that could be leveraged to improve treatment efficacy and outcomes of patients with CLL.

## Methods

### Study design and participants

This phase 3, randomized, open-label, parallel-group registrational study was conducted at 196 sites in 21 countries (Supplementary Table 13). The study was approved by the Central Ethics Committee of the University of Cologne, Germany. Patients were enrolled between 7 August 2015 and 4 August 2016. The study was registered at US and EU clinical trial registries (NCT02242942, EudraCT 2014-001810-24) and approved by ethical review boards responsible for each study site. The study was performed according to the principles of the Declaration of Helsinki. All patients provided written informed consent to participate. The study protocol is included in the Supplementary Information.

Patients were considered eligible for the study if they were 18 years or older, had previously untreated active CLL requiring treatment as per iwCLL criteria^33^ and were considered unfit due to coexisting conditions, as indicated by a CIRS score greater than 6 and/or an impaired renal function (creatinine clearance <70 ml/min). The full list of eligibility criteria is outlined in the study protocol (Supplementary Information).

### Procedures and outcomes

Patients were randomly assigned 1:1 to either six 28-day cycles of Ven-Obi, followed by single-agent venetoclax once daily for six cycles, or six cycles of Clb-Obi, followed by single-agent chlorambucil on day 1 and 15 for six cycles. Randomization procedure, dosing schedules and prophylactic measures have been previously published^13^.

Baseline assessments prior to study enrolment included immunophenotyping of circulating lymphocytes by flow cytometry, central analysis of genomic aberrations by FISH, sequencing of the IGHV and *TP53* gene by next generation DNA sequencing and assessment of lymph node size by physical examination and CT or MR imaging^13^. During post-treatment follow-up, no CT imaging was mandated or recommended as per iwCLL guidelines.

The primary endpoint was PFS, with disease progression or death from any cause constituting a PFS event, according to the iwCLL guidelines as determined by the study investigators. Secondary endpoints included MRD rates (measured by ASO-PCR, flow cytometry and NGS) in peripheral blood and bone marrow, TTNT and OS. The exploratory analyses included the relationship between various pre- and post-treatment markers and clinical outcome parameters (including cytogenetic aberrations, IGHV status, transcriptional profiles and MRD; particularly landmark analyses according to MRD status and analysis of time to MRD conversion).

AEs were reported until 28 days after the last dose of study treatment. Grade 3 or 4 AEs were to be reported for up to 6 months after last dose of study drug or until initiation of new anti-leukemic therpay. Grade ≥3 infections were reported for 2 years after last dose of study treatment, unless the patient received another line of anti-leukemic therapy after disease progression. After disease progression, only study treatment-related serious AEs and second primary malignancies were required to be reported by the investigator per protocol (Supplementary Table 7).

### RNA extraction, sequencing and transcriptomic analyses

Sample banking was conducted at centralised labs (University Hospitals Cologne, Kiel and Ulm, Germany, and Labcorp, NC, USA). CD19-positive cells (i.e. CLL cells) were enriched from peripheral blood mononuclear cells (PBMC) using CD19-positive microbeads following the manufacturer’s instruction (Miltenyi Biotec # 130-050-301). The median B-cell fraction of samples prior to CD19-enrichment, as assessed via immunophenotyping, was 97%. CD19-positive cells were lysed in RLT buffer and total RNA was isolated using RNeasy Mini Kit (QiAgen #74106). Thereby, RNA molecules longer than 200 nucleotides were enriched and eluted with nuclease-free water. RNA integrity (RIN) was measured by Agilent Bioanalyzer and accepted samples only passed QC. Sequencing libraries were created using Illumina TruSeq Standard mRNA method, which preferentially selects for messenger RNA (mRNA) by taking advantage of the polyadenylated tail. Briefly, total RNA samples were concentration normalized, and poly-adenylated RNA was purified using oligo-dT attached to magnetic beads. The purified mRNA was fragmented using heat in the presence of divalent cations and converted into double-stranded cDNA. They underwent end-repair, A-tailing, and polymerase chain reaction (PCR), after which they were quantified, normalized and pooled in preparation for sequencing. Libraries were sequenced using the Illumina sequencing-by-synthesis platform, with a sequencing protocol of 50 bp paired-end sequencing and total read depth of 50 M reads per sample.

RNA sequencing data in FASTQ were analysed using HTSeqGenie (v3.16.1) in BioConductor (v3.0) as follows: first, reads with low nucleotide qualities (70% of bases with quality <23) or matches to rRNA and adapter sequences were removed. The remaining reads were aligned to the human reference genome (human: GRCh38.p10) using GSNAP (PMID:20147302, 27008021) version ‘2013-10-10-v2’, allowing maximum of two mismatches per 75 base sequence (parameters: ‘-M 2 -n 10 -B 2 -i 1 -N 1 -w 200000 -E 1 --pairmax-rna=200000 --clip-overlap’). Transcript annotation was based on the Gencode genes database (GENCODE 27, biomaRt2.48.3). To quantify gene expression levels, the number of reads mapping unambiguously to the exons of each gene was calculated. The Raw counts mapped to chromosome X, chromosome Y, mitochondrial genes, long non-coding RNA genes and genes with minimal expression (less than 3 counts in 95% of samples) were removed. To estimate the cell type fraction as well as cell type-specific gene expression in each sequenced sample, data were deconvoluted and B-cell specific gene expression was imputed using CIBERSORTx^34^ (Supplementary Fig. 12).

A standard Limma and Voom pipeline was applied to transform the raw counts to log2 counts per million (Log2-CPM). Fold-change, *p*-values and moderated t-statistics from the Log2-CPM counts were calculated using *lmFit* and *eBayes* function and used for differential gene expression (DGE) analysis or Gene Set Enrichment Analysis (GSEA) (*fGSEA* package) with MSigDB Hallmark gene sets. *P*-values <0.01 and absolute log2 fold change >0.5 were used to identify differentially expressed genes. Volcano plots were used to represent the degree of fold-changes and statistical significance, and biologically relevant genes were additionally highlighted (*EnhancedVolcano* package).

To calculate the transcriptomic distance between patients, shared nearest neighbour (SNN) patient clustering was conducted using the *Seurat* package: Starting from linear-transformed TPM values derived from gene expression counts, we applied PCA for dimensionality reduction, determined the partitioning of distance by calculating k-nearest neighbours of each patient, and constructed a weighted network based on shared nearest neighbour (SNN) graph using Seurat’s *FindNeighbors* function. The final transcriptomic distance between patients was visualised in two dimensions via a non-linear dimension reduction method (Uniform Manifold Approximation and Projection [UMAP]), and the presence of mutations and cytogenetic markers of each patient were overlaid. Gene set variation analysis (GSVA) was conducted using the *GSVA* package^35^. GSVA scores of selected hallmark gene sets of interest were calculated and their distributions were stratified based on MRD response at follow-up month 3. Wilcoxon signed-rank test was applied to compare baseline and relapsed patients.

### Statistical analysis

The study sample size was calculated based on an assumed hazard ratio for progression or death of 0.65, with 170 events providing power of approximately 80% on the basis of a log-rank test stratified according to Binet stage and geographic region, with a two-sided statistical significance level of 0.05.

This report presents an updated analysis of survival and MRD status, as well association between gene expression and MRD response. Kaplan–Meier estimates were used to analyse the time-to-event data of PFS, OS and TTNT. Comparisons were done by a two-sided log-rank test and Cox proportional hazards regression model (both stratified by Binet stage and geographic region). Time to second primary malignancies referred to the time between randomization and date of first diagnosis of secondary malignancy. It was evaluated by competing risk analysis considering death as a competing risk and was compared by two-sided Gray’s test for equality of cumulative incidence functions. Patients without reported death or secondary malignancy were censored at the date when they were last known to be alive.

MRD response was defined as end of treatment MRD status <10^−6^ in peripheral blood, and MRD non-response was defined as end-of-treatment MRD status ≥10^−4^ in peripheral blood. Time to MRD conversion from the MRD assessment date at end of treatment was analysed using Kaplan-Meier methodology and was compared using non-stratified Cox proportional hazards regression model. An MRD conversion event was considered if MRD levels ≥ 10^−4^ in two consecutive visits were detected or patients had progression of disease or death due to progression of disease.

Landmark analyses from last treatment exposure were performed for PFS as pre-planned and for OS as post-hoc regarding MRD level at end of treatment. Treatment arm and baseline characteristics (as listed in Supplementary Table 2 and Supplementary Table 3) that were independently associated with PFS and OS in univariate analyses (non-stratified two-sided test level at 5%), were considered as candidates for the multivariate modelling. All reported p-values were exploratory without adjustments for multiple testing (two-sided test level at 5%).

All randomly assigned patients were included in the efficacy analyses (intention-to-treat population). All randomly assigned patients who received at least one dose of study medication (i.e., obinutuzumab, venetoclax, or chlorambucil) were included in the safety analyses (safety population).

Analyses were performed using SPSS version 28 (SPSS, Chicago, IL, USA), SAS version 9.4 (SAS Institute, Cary, NS, USA) and R version 3.6.1 (R Foundation, Vienna, Austria). R packages limma, Seurat, DESeq2, fGSEA and GSVA were used for the transcriptomic analyses.

### Reporting summary

Further information on research design is available in the Nature Portfolio Reporting Summary linked to this article.

### Supplementary information

Supplementary Information

Reporting Summary

Description of Additional Supplementary Files

Supplementary Data 1

Supplementary Data 2

### Source data

Source Data

## Data Availability

Individual patient-level data, including de-identified clinical metadata, raw RNAseq data, and processed RNAseq data are available to researchers at the European Genome-Phenome Archive under accession number EGAS00001006596. To request access to such data, researchers can contact the corresponding authors, who will facilitate the review by the GCLLSG/Roche/AbbVie data access committees. The data will be released to such requesters with necessary agreements to enforce terms such as security, patient privacy, and consent of specified data use, consistent with evolving, applicable data protection laws. Source data are provided with this paper, where applicable, and are also available at: github.com/othmanalsawaf/cll14_rna_5yr_paper. The study protocol is provided as Supplementary Note in the Supplementary Information file. The statistical analysis plan and informed consent form will be made available upon request to the corresponding authors. The remaining data are available within the Article, Supplementary Information or Source Data file. Source data are provided with this paper.

## Code availability

Codes are deposited on github.com/othmanalsawaf/cll14_rna_5yr_paper.
