# Author Correction: Transcriptomic profiles and 5-year results from the randomized CLL14 study of venetoclax plus obinutuzumab versus chlorambucil plus obinutuzumab in chronic lymphocytic leukemia

**DOI:** 10.1038/s41467-023-42562-2

**Published:** 2023-10-23

**Authors:** Othman Al-Sawaf, Can Zhang, Hyun Yong Jin, Sandra Robrecht, Yoonha Choi, Sandhya Balasubramanian, Alex Kotak, Yi Meng Chang, Anna Maria Fink, Eugen Tausch, Christof Schneider, Matthias Ritgen, Karl-Anton Kreuzer, Brenda Chyla, Joseph N. Paulson, Christian P. Pallasch, Lukas P. Frenzel, Martin Peifer, Barbara Eichhorst, Stephan Stilgenbauer, Yanwen Jiang, Michael Hallek, Kirsten Fischer

**Affiliations:** 1 Faculty of Medicine and University Hospital Cologne, Department I of Internal Medicine, Center for Integrated Oncology Aachen Bonn Cologne Duesseldorf, University of Cologne, Cologne, Germany; 2 Cancer Institute, University College London, London, UK; 3 Francis Crick Institute, London, UK; 4 Genentech Inc., South San Francisco, CA USA; 5 Roche Products Ltd, Welwyn Garden City, UK; 6 Hoffmann-la Roche, Mississauga, ON Canada; 7 Department III of Internal Medicine, Ulm University, Ulm, Germany; 8 Department II of Internal Medicine, University of Schleswig Holstein, Kiel, Germany; 9 AbbVie Inc., North Chicago, IL USA; 10 Faculty of Medicine and University Hospital Cologne, Department of Translational Genomics, University of Cologne, Cologne, Germany

**Keywords:** Chronic lymphocytic leukaemia, B-cell lymphoma, Cancer therapy

Correction to: *Nature Communications* 10.1038/s41467-023-37648-w, published online 18 April 2023

The original version of this article contained an error in the spelling of the author ‘Joseph N. Paulson’, which was incorrectly given as ‘Joe Paulson’.

The original version of this article contained an error in Figure 1d, in which the *y* axis was labeled ‘Cum Progression-free Survival’ rather than ‘Cum Survival’.

These errors have now been corrected in both the PDF and HTML versions of the article.

