# Supplementary material for: Transcriptomic profiles and 5-year results from the randomized CLL14 study of venetoclax plus obinutuzumab versus chlorambucil plus obinutuzumab in chronic lymphocytic leukemia
Source: Nat Commun. 2023 Apr 18;14:2147. doi: 10.1038/s41467-023-37648-w (PMC10113251; doi:10.1038/s41467-023-37648-w)
Supplement: Supplementary file 1 — Supplementary Information [file 41467_2023_37648_MOESM1_ESM.pdf]

SUPPLEMENTARY INFORMATION

Supplementary Table 1. Second-line anti-leukemic treatment (Intention-to-Treat Population)........................ 2
Supplementary Table 2. Univariate analyses for PFS in the Ven-Obi arm.. 2
Supplementary Table 3. Univariate analyses for PFS in the Clb-Obi arm... 5
Supplementary Table 4. Most frequent Grade 3-4 treatment emergent adverse event (Safety Population). ... 8
Supplementary Table 5. Most frequent treatment emergent serious adverse event (Safety Population)......... 9
Supplementary Table 6. Post-treatment treatment-related adverse events. 10
Supplementary Table 7. Reporting of adverse events. 12
Supplementary Table 8. Second-primary malignancies (SPM). 12
Supplementary Table 9. Grade 5 second-primary malignancies (SPM).. 13
Supplementary Table 10. Pre-treatment patient characteristics of relapsed RNAseq cohort. 14
Supplementary Table 11. Baseline patient characteristics according to inflammatory signature.................... 16
Supplementary Table 12. Frequency of infections according to inflammatory response enrichment. 17
Supplementary Table 13. CLL14 sites and investigators 18

Supplementary Figure 1. Consort diagram.. 22
Supplementary Figure 2. PFS according to IGHV mutational status and *TP53* aberrations.......................... 23
Supplementary Figure 2. TTNT, TTNT according to *TP53* aberrations and IGHV mutational status. 25
Supplementary Figure 4. OS according to *TP53* aberrations and IGHV mutational status............................ 28
Supplementary Figure 5. Time to Second-primary malignancy. 30
Supplementary Figure 6. MRD rates according to available samples. 31
Supplementary Figure 7. Time to MRD conversion (≥10^{-4}) by NGS from end of treatment........................... 32
Supplementary Figure 8. Sample selection for RNAseq sub-study... 33
Supplementary Figure 9. Pre-treatment transcriptomic SNN clustering and UMAP visualization. 34
Supplementary Figure 10. Differential gene expression between patients with uMRD and MRD+. 35
Supplementary Figure 11. Gene set enrichment analysis and differential gene expression between baseline and relapse.. 35
Supplementary Figure 12. Deconvolution of cell type fractions by CIBERSORTx. 36

Supplementary References.. 36

Supplementary Note: CONSORT checklist ……………………………………………………………………… 38
Supplementary Note: Study Protocol ……………………………………………………………………………… 40

Supplementary Table 1. Second-line anti-leukemic treatment (Intention-to-Treat Population).

	Clb-Obi<br><br>N (%)	Ven-Obi<br><br>N (%)	Total<br><br>N (%)
**Number of patients with 2nd-line treatment, N**	**94**	**31**	**125**
**BTK inhibitors**	**51 (54.3)**	**18 (58.1)**	**69 (55.2)**
Acalabrutinib (monotherapy or combination)	2 (2.1)	5 (16.1)	7 (5.6)
Zanubrutinib (monotherapy)	2 (2.1)	1 (3.2)	3 (2.4)
Ibrutinib (monotherapy or combination)	47 (50.0)	12 (38.7)	59 (47.2)
**Bcl2 inhibitors**	**12 (12.8)**	**5 (16.1)**	**17 (13.6)**
Venetoclax (monotherapy and combination)	12 (12.8)	5 (16.1)	17 (13.6)
**PI3K inhibitors**	**1 (1.1)**	**0**	**1 (0.8)**
Idelalisib-rituximab	1 (1.1)	0	1 (0.8)
**CD20 antibodies**	**1 (1.1)**	**0**	**1 (0.8)**
Rituximab monotherapy	1 (1.1)	0	1 (0.8)
**Chemo/Chemoimmunotherapy**	**29 (30.9)**	**8 (25.8)**	**37 (29.6)**
Bendamustine (± rituximab)	13 (13.8)	4 (12.9)	17 (13.6)
Chlorambucil (monotherapy and combination)	8 (8.5)	2 (6.4)	10 (8.0)
Cyclophosphamide, vincristine, prednisolone	1 (1.1)	0	1 (0.8)
Fludarabine, cyclophosphamide ± rituximab	3 (3.2)	0	3 (2.4)
R-CHOP	2 (2.1)	1 (3.2)	3 (2.4)
Other	2 (2.1)	1 (3.2)	3 (2.4)

Supplementary Table 2. Univariate analyses for PFS in the Ven-Obi arm.

P-values from univariate Cox regression analysis, not adjusted for multiple testing.

COX regression PFS, Ven-Obi	Patients<br><br>N (%)	Hazard ratio	95% Confidence Interval		P value
			Lower	Upper	
**Age (years)**	216				
≤ 65	36 (16.7)	Reference			
> 65	180 (83.3)	0.788	0.455	1.363	0.394
**Age (years)**	216				
≤ 74	144 (66.7)	Reference			
> 74	72 (33.3)	1.414	0.899	2.225	0.134
**Gender**	216				

Female	70 (32.4)	Reference			
Male	146 (67.6)	1.525	0.919	2.530	0.102
**Binet stage**	216				
A	46 (21.3)	Reference			
B/C	170 (78.7)	1.266	0.732	2.190	0.399
**Binet stage**	216				
A/B	122 (56.5)	Reference			
C	94 (43.5)	1.249	0.805	1.939	0.321
**Tumor Lysis Syndrome**	216				
Low	168 (77.8)	Reference			
Medium/High	48 (22.2)	0.834	0.441	1.576	0.576
**Tumor Lysis Syndrome**	216				
Medium/Low	29 (13.4)	Reference			
High	187 (86.6)	2.819	1.782	4.459	0,000009
**CIRS score**	216				
≤ 6	30 (13.9)	Reference			
> 6	186 (86.1)	1.299	0.626	2.697	0.483
**Creatinine clearance (Cockroft-Gault) (ml/min)**	215				
< 70	129 (60.0)	Reference			
≥ 70	86 (40.0)	0.750	0.474	1.188	0.221
**ECOG performance status score**	216				
0	89 (41.2)	Reference			
≥ 1	127 (58.8)	1.221	0.780	1.910	0.383
**B symptoms**	216				
No	113 (52.3)	Reference			
Yes	103 (47.7)	0.887	0.572	1.378	0.595
**Serum β2-microglobulin (mg/L)**	202				
≤ 3.5	82 (40.6)	Reference			

> 3.5	120 (59.4)	1.737	1.072	2.814	0.025
**Deletion in 17p**	210				
No	193 (91.9)	Reference			
Yes	17 (8.1)	3.174	1.745	5.773	0.000153
**Deletion in 11q**	210				
No	171 (81.4)	Reference			
Yes	39 (18.6)	1.412	0.843	2.366	0.190
**Trisomy in 12**	210				
No	170 (81.0)	Reference			
Yes	40 (19.0)	0.717	0.379	1.357	0.307
**Deletion in 13q**	210				
No	103 (49.0)	Reference			
Yes	107 (51.0)	0.820	0.527	1.276	0.379
**IGHV mutational status**	197				
Mutated	121 (61.4)	Reference			
Unmutated	76 (38.6)	2.481	1.438	4.282	0.001
**TP53 status**	209				
No Mutation/deletion	184 (88.0)	Reference			
Mutation and/or deletion	25 (12.0)	2.366	1.344	4.166	0.003
**TP53 mutational status**	211				
No mutation	188 (89.1)	Reference			
Mutation	23 (10.9)	2.376	1.330	4.244	0.003
**Complex karyotype**	200				
NCKT	166 (83.0)	Reference			
CKT/HCKT	34 (17.0)	1.507	0.866	2.623	0.147
**Max. lymph node size (cm)**	200				
< 5 cm	139 (69.5)	Reference			
≥ 5 cm	61 (30.5)	2.790	1.775	4.388	0.000009

**Absolute lymphocyte count**	163				
< 25	46 (28.2)	Reference			
≥ 25	117 (71.8)	0.730	0.457	1.163	0.185
**Absolute lymphocyte count**	163				
< 50	66 (40.5)	Reference			
≥ 50	97 (59.5)	0.985	0.631	1.538	0.946
**Absolute lymphocyte count**	163				
< 75	86 (52.8)	Reference			
≥ 75	77 (47.2)	1.206	0.767	1.895	0.418
**Absolute lymphocyte count**	163				
< 100	108 (66.3)	Reference			
≥ 100	55 (33.7)	0.986	0.598	1.627	0.956

Supplementary Table 3. Univariate analyses for PFS in the Clb-Obi arm.

P-values from univariate Cox regression analysis, not adjusted for mulitple testing.

COX regression PFS, Clb-Obi (1)	Patients N (%)	Hazard ratio	95% Confidence Interval		P value
			Lower	Upper	
**Age (years)**	216				
≤ 65	37 (17.1)	Reference			
> 65	179 (82.9)	1.181	0.756	1.845	0.464
**Age (years)**	216				
≤ 74	138 (63.9)	Reference			
> 74	78 (36.1)	0.926	0.660	1.299	0.656
**Gender**	216				
Female	73 (33.8)	Reference			
Male	143 (66.2)	0.826	0.590	1.158	0.268
**Binet stage**	216				
A	44 (20.4)	Reference			
B/C	172 (79.6)	0.957	0.644	1.422	0.828

**Binet stage**	216				
A/B	124 (57.4)	Reference			
C	92 (42.6)	0.871	0.628	1.206	0.405
**Tumor Lysis Syndrome**	216				
Low	173 (80.1)	Reference			
Medium/High	43 (19.9)	1.359	0.809	2.284	0.247
**Tumor Lysis Syndrome**	216				
Medium/Low	26 (12.0)	Reference			
High	190 (88.0)	1.556	1.060	2.283	0.024
**CIRS score**	216				
≤ 6	39 (18.1)	Reference			
> 6	177 (81.9)	1.359	0.889	2.078	0.157
**Creatinine clearance (Cockroft-Gault) (ml/min)**	213				
< 70	119 (55.9)	Reference			
≥ 70	94 (44.1)	1.036	0.747	1.435	0.833
**ECOG performance status score**	215				
0	103 (47.9)	Reference			
≥ 1	112 (52.1)	1.424	1.029	1.971	0.033
**B symptoms**	216				
No	104 (48.1)	Reference			
Yes	112 (51.9)	1.386	1.002	1.916	0.048
**Serum β2-microglobulin (mg/L)**	207				
≤ 3.5	79 (38.2)	Reference			
> 3.5	128 (61.8)	1.716	1.199	2.456	0.003
**Deletion in 17p**	208				
No	194 (93.3)	Reference			
Yes	14 (6.7)	3.157	1.769	5.633	0.0001

**Deletion in 11q**	208				
No	170 (81.7)	Reference			
Yes	38 (18.3)	1.939	1.299	2.895	0.001
**Trisomy in 12**	208				
No	164 (78.8)	Reference			
Yes	44 (21.2)	1.178	0.790	1.754	0.421
**Deletion in 13q**	208				
No	94 (45.2)	Reference			
Yes	114 (54.8)	0.762	0.547	1.060	0.106
**IGHV mutational status**	206				
Mutated	83 (40.3)	Reference			
Unmutated	123 (59.7)	3.088	2.124	4.489	3,4544E-9
**TP53 status**	208				
No Mutation/deletion	184 (88.5)	Reference			
Mutation and/or deletion	24 (11.5)	1.699	1.058	2.731	0.028
**TP53 mutational status**	210				
No mutation	191 (91.0)	Reference			
Mutation	19 (9.0)	1.580	0.924	2.702	0.094
**Complex karyotype**	197				
NCKT	167 (84.8)	Reference			
CKT/HCKT	30 (15.2)	2.855	1.825	4.466	0,000004
**Max. lymph node size (cm)**	201				
< 5 cm	149 (74.1)	Reference			
≥ 5 cm	52 (25.9)	1.645	1.148	2.357	0.007
**Absolute lymphocyte count**	171				
< 25	31 (18.1)	Reference			
≥ 25	140 (81.9)	1.710	1.076	2.718	0.023
**Absolute lymphocyte count**	171				
< 50	73 (42.7)	Reference			

≥ 50	98 (57.3)	1.585	1.131	2.220	0.007
**Absolute lymphocyte count**	171				
< 75	102 (59.6)	Reference			
≥ 75	69 (40.4)	1.701	1.223	2.365	0.002
**Absolute lymphocyte count**	171				
< 100	117 (68.4)	Reference			
≥ 100	54 (31.6)	1.912	1.352	2.704	0.000244

Supplementary Table 4. Most frequent Grade 3-4 treatment emergent adverse event (Safety Population).

Adverse events are reported according to Medical Dictionary for Regulatory Activities superclass and preferred terms and National Cancer Institute Common Terminology Criteria for Adverse Events grade. Shown are Grade 3, 4 events with frequency ≥1%.

	**Clb-Obi (n=214)**	**Ven-Obi (n=212)**
Total number of patients with at least one Grade 3,4 adverse event	163 (76.2%)	176 (83.0%)
NEUTROPENIA	102 (47.7%)	112 (52.8%)
THROMBOCYTOPENIA	32 (15.0%)	30 (14.2%)
INFUSION RELATED REACTION	22 (10.3%)	19 (9.0%)
ANAEMIA	14 (6.5%)	18 (8.5%)
PNEUMONIA	9 (4.2%)	14 (6.6%)
FEBRILE NEUTROPENIA	8 (3.7%)	11 (5.2%)
NEUTROPHIL COUNT DECREASED	10 (4.7%)	9 (4.2%)
LEUKOPENIA	10 (4.7%)	5 (2.4%)
ASPARTATE AMINOTRANSFERASE INCREASED	7 (3.3%)	5 (2.4%)
ALANINE AMINOTRANSFERASE INCREASED	7 (3.3%)	4 (1.9%)
HYPERGLYCAEMIA	3 (1.4%)	8 (3.8%)
TUMOUR LYSIS SYNDROME	7 (3.3%)	3 (1.4%)
DIARRHOEA	1 (0.5%)	8 (3.8%)
SYNCOPE	4 (1.9%)	4 (1.9%)
ATRIAL FIBRILLATION	3 (1.4%)	5 (2.4%)

HYPERTENSION	1 (0.5%)	7 (3.3%)
ASTHENIA	1 (0.5%)	6 (2.8%)
PROSTATE CANCER	3 (1.4%)	3 (1.4%)
SEPSIS	2 (0.9%)	4 (1.9%)
DYSPNOEA	1 (0.5%)	5 (2.4%)
LYMPHOPENIA	4 (1.9%)	1 (0.5%)
BRONCHITIS	3 (1.4%)	2 (0.9%)
HYPOTENSION	3 (1.4%)	2 (0.9%)
PYREXIA	3 (1.4%)	2 (0.9%)
PLATELET COUNT DECREASED	1 (0.5%)	4 (1.9%)
GRANULOCYTOPENIA	3 (1.4%)	1 (0.5%)
MYOCARDIAL INFARCTION	3 (1.4%)	1 (0.5%)
ARTHRALGIA	1 (0.5%)	3 (1.4%)
HYPERPHOSPHATAEMIA	1 (0.5%)	3 (1.4%)
HYPERURICAEMIA	1 (0.5%)	3 (1.4%)
VERTIGO	1 (0.5%)	3 (1.4%)
WHITE BLOOD CELL COUNT DECREASED	1 (0.5%)	3 (1.4%)
CHEST PAIN	0	4 (1.9%)
HEPATIC ENZYME INCREASED	0	4 (1.9%)
ACUTE MYOCARDIAL INFARCTION	0	3 (1.4%)
AORTIC STENOSIS	0	3 (1.4%)
BASAL CELL CARCINOMA	0	3 (1.4%)
CARDIAC FAILURE	0	3 (1.4%)
CYTOKINE RELEASE SYNDROME	0	3 (1.4%)

Supplementary Table 5. Most frequent treatment emergent serious adverse event (Safety Population).

Adverse events are reported according to Medical Dictionary for Regulatory Activities superclass and preferred terms and National Cancer Institute Common Terminology Criteria for Adverse Events grade. Shown are serious adverse events with frequency ≥1%.

	**Clb-Obi (n=214)**	**Ven-Obi (n=212)**
Total number of patients with at least one Grade 3,4 adverse event	102 (47.7%)	127 (59.9%)

PNEUMONIA	11 (5.1%)	15 (7.1%)
INFUSION RELATED REACTION	13 (6.1%)	10 (4.7%)
FEBRILE NEUTROPENIA	8 (3.7%)	11 (5.2%)
PYREXIA	7 (3.3%)	7 (3.3%)
SEPSIS	3 (1.4%)	8 (3.8%)
THROMBOCYTOPENIA	5 (2.3%)	2 (0.9%)
PROSTATE CANCER	3 (1.4%)	4 (1.9%)
ATRIAL FIBRILLATION	4 (1.9%)	2 (0.9%)
CHRONIC OBSTRUCTIVE PULMONARY DISEASE	3 (1.4%)	3 (1.4%)
TUMOUR LYSIS SYNDROME	4 (1.9%)	1 (0.5%)
MYOCARDIAL INFARCTION	3 (1.4%)	2 (0.9%)
SQUAMOUS CELL CARCINOMA OF SKIN	3 (1.4%)	2 (0.9%)
INFECTION	2 (0.9%)	3 (1.4%)
CARDIAC FAILURE	1 (0.5%)	4 (1.9%)
NEUTROPENIA	1 (0.5%)	4 (1.9%)
ASPARTATE AMINOTRANSFERASE INCREASED	4 (1.9%)	0
INFLUENZA	3 (1.4%)	1 (0.5%)
VERTIGO	1 (0.5%)	3 (1.4%)
ALANINE AMINOTRANSFERASE INCREASED	3 (1.4%)	0
SEPTIC SHOCK	3 (1.4%)	0
ACUTE MYOCARDIAL INFARCTION	0	3 (1.4%)
AORTIC STENOSIS	0	3 (1.4%)
CELLULITIS	0	3 (1.4%)
CEREBRAL ISCHAEMIA	0	3 (1.4%)

Supplementary Table 6. Post-treatment treatment-related adverse events.

Investigator-assessed relatedness.

	Clb-Obi (n=208)	Ven-Obi (n=201)
Total number of patients with at least one adverse event	10 (4.8%)	17 (8.5%)
NEUTROPENIA	2 (1.0%)	6 (3.0%)
FEBRILE NEUTROPENIA	1 (0.5%)	2 (1.0%)

PNEUMONIA	1 (0.5%)	1 (0.5%)
SEPSIS	1 (0.5%)	1 (0.5%)
ANAEMIA	0	1 (0.5%)
ATRIAL FIBRILLATION	1 (0.5%)	1 (0.5%)
HEADACHE	0	1 (0.5%)
BASAL CELL CARCINOMA	1 (0.5%)	0
BLADDER NEOPLASM	1 (0.5%)	0
CALCULUS BLADDER	1 (0.5%)	0
CANDIDA INFECTION	0	1 (0.5%)
COLON CANCER	0	1 (0.5%)
COVID-19	1 (0.5%)	0
DYSPNOEA	0	1 (0.5%)
HAEMOPTYSIS	1 (0.5%)	0
HEPATITIS VIRAL	1 (0.5%)	0
INFECTION	0	1 (0.5%)
MALIGNANT MELANOMA	0	1 (0.5%)
OPHTHALMIC HERPES ZOSTER	0	1 (0.5%)
PENILE CANCER	0	1 (0.5%)
PLASMA CELL MYELOMA	1 (0.5%)	0
PNEUMONIA STREPTOCOCCAL	0	1 (0.5%)
PNEUMONITIS	0	1 (0.5%)
PROSTATE CANCER	1 (0.5%)	0
SINUSITIS	0	1 (0.5%)
SINUSITIS ASPERGILLUS	0	1 (0.5%)
SPLENOMEGALY	0	1 (0.5%)
STAPHYLOCOCCAL BACTERAEMIA	1 (0.5%)	0
STREPTOCOCCAL INFECTION	1 (0.5%)	0
T-CELL LYMPHOMA	0	1 (0.5%)
THROMBOCYTOPENIA	0	1 (0.5%)

TOXICITY OF VARIOUS AGENTS	0	1 (0.5%)

Supplementary Table 7. Reporting of adverse events.

	RELATED		UNRELATED	
	Post-Treatment Reporting Period	Follow-up	Post-treatment Reporting Period	Follow-up
AEs Grades 1 and 2	28 days	Not required	28 days	Not required
AEs Grades 3 and 4	6 months or NLT	Until resolution to ≤Grade 2	6 months or NLT	Until resolution to ≤Grade 2
Major infections (Grades 3 and 4)	2 years or NLT	Until resolution stabilization or end of study	2 years or NLT	Until resolution stabilization or 1 year after onset
SAEs	Indefinitely	Until resolution stabilization or end of study	Throughout follow-up period	Until resolution stabilization or 1 year after onset
Secondary malignancies	Indefinitely	Not required	Indefinitely	Not required

AE = adverse event; SAE = serious adverse event; NLT = next leukemia treatment.

Supplementary Table 8. Second-primary malignancies (SPM).
Investigator reported terms. Non-melanoma skin cancers (not listed here) occurred in 18 patients in the Clb-Obi arm and 19 patients in the Ven-Obi arm.

	Clb-Obi N (%)	Ven-Obi N (%)
**Overall total number of SPM events**	17	28
**Number of patients with at least one SPM, N**	**16**	**27**
**Solid organ tumor**	**10 (62.5)**	**15 (55.6)**
Adenocarcinoma of colon	2 (12.5)	1 (3.7)
Bladder cancer	0	2 (7.4)
Bladder cancer recurrent	0	1 (3.7)
Bladder transitional cell carcinoma	0	1 (3.7)
Colon cancer	0	1 (3.7)
Hepatocellular carcinoma	1 (6.3)	0
Invasive breast carcinoma	0	1 (3.7)
Invasive ductal breast carcinoma	0	1 (3.7)
Lung adenocarcinoma	2 (12.5)	1 (3.7)
Lung adenocarcinoma stage IV	0	1 (3.7)
Lung neoplasm malignant	1 (6.3)	0
Pancreatic carcinoma metastatic	1 (6.3)	0
Prostate cancer	3 (18.8)	4 (14.8)

Pleomorphic malignant fibrous histiocytoma	1 (6.3)	0
Prostate cancer metastatic	0	1 (3.7)
**Melanoma**	**3 (18.8)**	**8 (29.6)**
Conjunctival melanoma	0	1 (3.7)
Malignant melanoma	1 (6.3)	3 (11.1)
Malignant melanoma in situ	2 (12.5)	3 (11.1)
Metastatic malignant melanoma	0	1 (3.7)
**Hematologic malignancies**	**2 (12.5)**	**3 (11.1)**
Acute myeloid Leukaemia	1 (6.3)	0
Myelodysplastic syndrome	0	1 (3.7)
Myeloid maturation arrest	0	1 (3.7)
Plasma cell myeloma	1 (6.3)	0
T-cell lymphoma	0	1 (3.7)
**Other**	**1 (6.3)**	**1 (3.7)**
Anal squamous cell carcinoma	1 (6.3)	0
Penile cancer	0	1 (3.7)

Supplementary Table 9. Grade 5 second-primary malignancies (SPM).

	**Clb-Obi N (%)**	**Ven-Obi N (%)**
**Overall total number of grade 5 SPM events**	8	4
**Number of patients with grade 5 SPM, N**	**8**	**4**
Acute myeloid leukaemia	1 (12.5)	0
Adenocarcinoma of colon	1 (12.5)	1 (25.0)
Bladder cancer	0	1 (25.0)
Hepatocellular carcinoma	1 (12.5)	0
Lung neoplasm malignant	1 (12.5)	0
Metastatic malignant melanoma	0	1 (25.0)
Myelodysplastic syndrome	0	1 (25.0)
Pancreatic carcinoma metastatic	1 (12.5)	0
Pleomorphic malignant fibrous histiocytoma	1 (12.5)	0

Supplementary Table 10. Pre-treatment patient characteristics of relapsed RNAseq cohort.

CIRS denotes Cumulative Illness Rating Scale, CLL-IPI International Prognostic Index for chronic lymphocytic leukemia, ECOG Eastern Cooperative Oncology Group, IGHV immunoglobulin heavy-chain variable-region gene, and TLS tumor lysis syndrome. † B-symptoms include the presence of fever, night sweats, significant fatigue or unintentional weight loss. ‡ ECOG performance status scores range from 0 to 5, with higher scores indicating greater disability; a score of 5 indicates death. § According to the hierarchical model of Döhner at al. [1]

**Characteristic**	**Clb-Obi** (N=15)	**Ven-Obi** (N=29)	**Total** (N=44)
**Patients with available RNAseq data at relapse, N**	**15**	**29**	**44**
Age			
Median — yr (range)	71 (66-78)	71 (45-84)	71 (45-84)
≥75 yr — N (%)	5 (33.3)	8 (27.6)	13 (29.5)
Male sex — N (%)	8 (53.3)	20 (69.0)	28 (63.6)
Median time from diagnosis — mo (range)	25.3 (0.6-244.8)	23.9 (0.7-85.7)	24.6 (0.6-244.8)
Binet stage — N (%)			
A	4 (26.7)	8 (27.6)	12 (27.3)
B	7 (46.7)	13 (44.8)	20 (45.5)
C	4 (26.7)	8 (27.6)	12 (27.3)
B-symptoms present — N (%)†	8 (53.3)	12 (41.4)	20 (45.5)
Disease burden category (TLS risk category) — N (%)			
Low	2 (13.3)	2 (6.9)	4 (9.1)
Intermediate	11 (73.3)	10 (34.5)	21 (47.7)
High	2 (13.3)	17 (58.6)	19 (43.2)
Total CIRS score			
Median (range)	9 (2-14)	9 (5-23)	9 (2-23)
>6 — N (%)	14 (93.3)	28 (96.6)	42 (95.5)
Estimated creatinine clearance			
Median — ml/min (range)	64.5 (31.6-116.4)	63.5 (38.2-176.1)	63.7 (31.6-176.1)
<70 ml/min — N (%)	10 (66.7)	20 (69.0)	30 (68.2)
ECOG performance status score — N (%)‡			
0	6 (40.0)	14 (48.3)	20 (45.5)
1	8 (53.3)	11 (37.9)	19 (43.2)
2	1 (6.7)	4 (13.8)	5 (11.4)
3	0	0	0
Serum β_2 microglobulin			

**Characteristic**	**Clb-Obi** (N=15)	**Ven-Obi** (N=29)	**Total** (N=44)
**Patients with available RNAseq data at relapse, N**	**15**	**29**	**44**
Median — mg/l (range)	4.5 (2.1-12.5)	4.6 (1.5-10.5)	4.5 (1.5-12.5)
>3.5 mg/l — N (%)	10/15 (66.7)	20/28 (71.4)	30/43 (69.8)
Cytogenetic subgroups as per hierarchy§ — N (%)			
Deletion in 17p	0	5 (17.2)	5 (11.4)
Deletion in 11q	4 (26.7)	8 (27.6)	12 (27.3)
Trisomy in 12	3 (20.0)	4 (13.8)	7 (15.9)
No abnormalities	3 (20.0)	4 (13.8)	7 (15.9)
Deletion in 13q alone	5 (33.3)	8 (27.6)	13 (29.5)
IGHV mutational status — N (%)			
Mutated	7/15 (46.7)	4/28 (14.3)	11/43 (25.6)
Unmutated	8/15 (53.3)	22/28 (78.6)	30/43 (69.8)
Not evaluable	0	2/28 (7.1)	2/43 (4.7)
TP53 mutational status — N (%)			
Mutated	1 (6.7)	4 (13.8)	5 (11.4)
Unmutated	14 (93.3)	25 (86.2)	39 (88.6)
TP53 deleted and/or mutated — N (%)	1 (6.7)	5 (17.2)	6 (13.6)
CLL-IPI risk group [NEJM] — N (%)			
Low	1/15 (6.7)	1/27 (3.7)	2/42 (4.8)
Intermediate	3/15 (20.0)	3/27 (11.1)	6/42 (14.3)
High	11/15 (73.3)	21/27 (77.8)	32/42 (76.2)
Very high	0	2/27 (7.4)	2/42 (4.8)
Complex karyotype group — N (%)			
NCKT	11 (78.6)	22 (75.9)	33 (76.7)
CKT / HCKT	3 (21.4)	7 (24.1)	10 (23.3)

Supplementary Table 11. Baseline patient characteristics according to inflammatory signature.

Characteristic	High inflammatory response (N=84)	Low inflammatory response (N=334)	Total (N=418)
Age			
Median — yr (range)	72.5 (49-88)	71.5 (41-89)	72 (41-89)
≥75 yr — N (%)	30 (35.7)	115 (34.4)	145 (34.7)
Male sex — N (%)	50 (59.5)	225 (67.4)	275 (65.8)
Median time from diagnosis — mo (range)	19.6 (0.4-224.8)	33.5 (0.3-244.8)	30.9 (0.3-244.8)
Binet stage — N (%)			
A	25 (29.8)	65 (19.5)	90 (21.5)
B	32 (38.1)	119 (35.6)	151 (36.1)
C	27 (32.1)	150 (44.9)	177 (42.3)
B-symptoms present — N (%)	47 (56.0)	158 (47.3)	205 (49.0)
Disease burden category (TLS risk category) — N (%)			
Low	22 (26.2)	28 (8.4)	50 (12.0)
Intermediate	49 (58.3)	226 (67.7)	275 (65.8)
High	13 (15.5)	80 (24.0)	93 (22.2)
Total CIRS score			
Median (range)	8 (1-26)	8 (0-28)	8 (0-28)
>6 — N (%)	73 (86.9)	277 (82.9)	350 (83.7)
Estimated creatinine clearance			
Median — ml/min (range)	67.3 (30.4-196.3)	65.8 (25.1-295.6)	66.0 (25.1-295.6)
<70 ml/min — N (%)	48/84 (57.1)	195/330 (59.1)	243/414 (58.7)
ECOG performance status score — N (%)			
0	39 (46.4)	149 (44.6)	188 (45.0)
1	28 (33.3)	148 (44.3)	176 (42.1)
2	17 (20.2)	36 (10.8)	53 (12.7)
3	0	1 (0.3)	1 (0.2)
Serum β_2 microglobulin			
Median — mg/l (range)	4.4 (1.0-20.0)	4.0 (1.3-14.2)	4.1 (1.0-20.0)
>3.5 mg/l — N (%)	52/81 (64.2)	194/321 (60.4)	246/402 (61.2)
Cytogenetic subgroups as per hierarchy — N (%)			

Characteristic	High inflammatory response (N=84)	Low inflammatory response (N=334)	Total (N=418)
Deletion in 17p	8/81 (9.9)	23/322 (7.1)	31/403 (7.7)
Deletion in 11q	9/81 (11.1)	65/322 (20.2)	74/403 (18.4)
Trisomy in 12	26/81 (32.1)	47/322 (14.6)	73/403 (18.1)
No abnormalities	20/81 (24.7)	66/322 (20.5)	86/403 (21.3)
Deletion in 13q alone	18/81 (22.2)	121/322 (37.6)	139/403 (34.5)
IGHV mutational status — N (%)			
Mutated	29/78 (37.2)	120/314 (38.2)	149/392 (38.0)
Unmutated	48/78 (61.5)	189/314 (60.2)	237/392 (60.5)
Not evaluable	1/78 (1.3)	5/314 (1.6)	6/392 (1.5)
TP53 mutational status — N (%)			
Mutated	11/80 (13.8)	32/325 (9.8)	43/405 (10.6)
Unmutated	69/80 (86.3)	293/325 (90.2)	362/405 (89.4)
TP53 deleted and/or mutated — N (%)	14/80 (17.5)	35/321 (10.9)	49/401 (12.2)
CLL-IPI risk group [NEJM] — N (%)			
Low	11/74 (14.9)	24/296 (8.1)	35/370 (9.5)
Intermediate	17/74 (23.0)	79/296 (26.7)	96/370 (25.9)
High	42/74 (56.8)	179/296 (60.5)	221/370 (59.7)
Very high	4/74 (5.4)	14/296 (4.7)	18/370 (4.9)
Complex karyotype group — N (%)			
NCKT	68/79 (86.1)	253/304 (83.2)	321/383 (83.8)
CKT / HCKT	11/79 (13.9)	51/304 (16.8)	62/383 (16.2)

Supplementary Table 12. Frequency of infections according to inflammatory response enrichment.

Characteristic	High inflammatory response (N=84)	Low inflammatory response (N=334)	Total (N=418)
At least one infection — N (%)	43 (51.2)	184 (55.1)	227 (54.3)
At least one infection of CTC Grade ≥ 3 — N (%)	16 (19.0)	73 (21.9)	89 (21.3)

Supplementary Table 13. CLL14 sites and investigators

Investigator	Country	Institution
FANTL, DOROTEA	Argentina	Hospital Italiano de Buenos Aires
Opat, Stephen	Australia	Monash Medical Centre Clayton
SCHWARER, ANTHONY	Australia	Box Hill Hospital
Harvey, Michael	Australia	South Western Sydney Local Health District - Liverpool Hospital
TO, BIK	Australia	Royal Adelaide Hospital
Marlton, Paula	Australia	Princess Alexandra Hospital
Ho, Prahlad	Australia	The Northern Hospital
Sia, Hanlon	Australia	The Tweed Hospital
Wickham, Nicholas	Australia	Ashford Cancer Centre Research
Lai, Hock-Choong	Australia	Townsville Hospital (TTH)
Heintel, Daniel	Austria	Wilhelminenspital
Noesslinger, Thomas	Austria	Hanusch-Krankenhaus
JAEGER, ULRICH G.	Austria	Medizinische Universität Wien
Steurer, Michael	Austria	Medizinische Universität Innsbruck
Renata Russo	Brazil	Hospital Santa Marcelina
Fogliatto, Laura Maria	Brazil	Hospital de Clínicas de Porto Alegre
Santucci, Rodrigo	Brazil	IEP São Lucas - Instituto de Ensino e Pesquisa
da Silva, Celso	Brazil	Hospital Sirio Libanés
Buccheri, Valeria	Brazil	Hospital das Clínicas da Faculdade de Medicina da Universidade de São Paulo
Sivcheva, Liliya	Bulgaria	Multiprofile Hospital for Active Treatment "Hristo Botev"
TZVETKOV, NIKOLAY	Bulgaria	University Multiprofile Hospital for Active Treatment "Dr. G. Stranski" EAD
SPASOV, EMIL	Bulgaria	University Multiprofile Hospital for Active Treatment "Sveti Georgi" EAD
Nikolova-Yaprakova, Raya	Bulgaria	University Multiprofile Hospital For Active Treatment "Sveti Ivan Rilski" EAD
NENOVA, IVANKA	Bulgaria	University Multiprofile Hospital for Active Treatment "Sveti Georgi" EAD
Owen, Carolyn	Canada	Tom Baker Cancer Centre
ROBINSON, SUE	Canada	Queen Elizabeth II Health Sciences Centre
Assouline, Sarit	Canada	Jewish General Hospital
AURER, IGOR	Croatia	Klinički Bolnički Centar Zagreb
Ostojic, Slobodanka	Croatia	Klinička Bolnica Merkur
Niemann, Carsten	Denmark	Rigshospitalet
Bjørn Poulsen, Christian	Denmark	Roskilde Sygehus
Engaard, Lisbeth	Denmark	Herlev Hospital

PLESNER, TORBEN	Denmark	Sygehus Lillebaelt - Vejle Sygehus
VIIGIMAA, IIGE	Estonia	North Estonia Medical Centre Foundation
KAARE, AIN	Estonia	Tartu University Hospital
Le Du, Katell	France	Centre Jean Bernard
MAHE, BEATRICE	France	CHU de Nantes - Hôtel-Dieu
VILQUE, JEAN-PIERRE	France	CHU Caen
Molina, Lysiane	France	CHU de Grenoble
ZINI, JEAN MARC	France	Hôpital Saint-Louis
Dupuis, Jehan-Michel	France	CHU Hôpital Henri Mondor
De Guibert, Sophie	France	CHU de Rennes - Hôpital Pontchaillou
LEPRETRE, STEPHANE	France	Centre Henri Becquerel - Centre de Lutte Contre le Cancer
Cartron, Guillaume	France	CHRU Montpellier - Saint Eloi
Sohn, Claudine	France	Hôpital Sainte Musse
BELHABRI, AMINE	France	Centre Léon Bérard
TOURNILHAC, OLIVIER	France	CHU Estaing
RIBRAG, VINCENT	France	Gustave Roussy
Brion, Annie	France	CHU Besançon - Hôpital Jean Minjoz
Casasnovas, Olivier	France	CHU Dijon - Hôpital François Mitterrand
Jacobasch, Lutz	Germany	Gemeinschaftspraxis Hämatologie - Onkologie
Müller, Martin	Germany	Universitätsklinikum Tübingen
Hebart, Holger	Germany	Stauferklinikum Schwäbisch Gmünd
Dreger, Peter	Germany	Universitätsklinikum Heidelberg
Schultheis, Beate Susanne	Germany	Marien Hospital Herne
Bogner, Christian	Germany	Klinikum rechts der Isar der TU München
Schetelig, Johannes	Germany	Universitätsklinikum Carl Gustav Carus Dresden
Wendtner, Clemens	Germany	Klinikum Schwabing
Mannal, Rebekka	Germany	Klinikum Esslingen GmbH
Dierks, Christine	Germany	Universitätsklinikum Freiburg
GASKA, TOBIAS	Germany	Brüderkrankenhaus St. Josef Paderborn
Pezzutto, Antonio	Germany	Charité - Universitätsmedizin Berlin
HALLEK, MICHAEL	Germany	Universitätsklinikum Köln
Fischer von Weikersthal, Ludwig	Germany	Klinikum St. Marien
Graeven, Ullrich	Germany	Kliniken Maria Hilf GmbH - Krankenhaus St. Franziskus
Kiehl, Michael	Germany	Klinikum Frankfurt (Oder) GmbH
Klaproth, Holger	Germany	Gemeinschaftspraxis Dr. Med. Peter Schmidt/Dr. Med. Holger Klaproth

Heilmeier, Bernhard	Germany	Krankenhaus Barmherzige Brüder Regensburg
zur Hausen, Kordelia	Germany	Universitätsklinikum Aachen
Pezzutto, Antonio	Germany	Charité - Universitätsmedizin Berlin - Campus Benjamin Franklin (CBF)
Denzlinger, Claudio	Germany	Marienhospital Stuttgart
van Roye, Christoph	Germany	Institut für Versorgungsforschung in der Onkologie
Dürig, Jan	Germany	Universitätsklinikum Essen
Vöhringer, Matthias	Germany	Robert-Bosch-Krankenhaus
Funck, Robert	Germany	MVZ Weiden GmbH
LIBERATI, ANNA MARINA	Italy	Azienda Ospedaliera di Perugia - Ospedale Santa Maria della Misericordia
Coscia, Marta	Italy	Azienda Ospedaliera Universitaria Citta della Salute e della Scienza di Torino - Ospedale Molinette
Mannina, Donato	Italy	Azienda Ospedaliera Papardo
CUNEO, ANTONIO	Italy	Azienda Ospedaliero-Universitaria di Ferrara - Arcispedale Sant'Anna
Bassan, Renato	Italy	Ospedale dell'Angelo
Laurenti, Luca	Italy	Fondazione Policlinico Universitario Agostino Gemelli
GHIA, PAOLO	Italy	Università Vita-Salute San Raffaele
VALDES-CERDA, CARLOS ALBERTO	Mexico	Hospital General de Culiacán
BUTLER, ANDREW	New Zealand	Canterbury Health Laboratories
Weinkove, Robert	New Zealand	Wellington Hospital
Kilfoyle, Allanah	New Zealand	Palmerston North Hospital
McDiarmid, Bridgett	New Zealand	Dunedin Hospital
ROBAK, TADEUSZ	Poland	Wojewódzkie Wielospecjalistyczne Centrum Onkologii i Traumatologii im. M. Kopernika w Łodzi
Grosicki, Sebastian	Poland	Samodzielny Publiczny Zaklad Opieki Zdrowotnej w Dzialdowie
Homenda, Wojciech	Poland	Wojewódzki Szpital Specjalistyczny im. Janusza Korczaka
KULICZKOWSKI, KAZIMIERZ	Poland	Samodzielny Publiczny Szpital Kliniczny Nr. 1
VASILICA, MARIANA	Romania	Institutul Clinic Fundeni
Oltean, Galafteon	Romania	Spitalul Clinic Judetean de Urgenta Tirgu-Mures
BURCOVEANU, CRISTINA	Romania	Institutul Regional de Oncologie Iași
Chagorova, Tatiana	Russia	Penza Regional Oncology Dispensary
SAMOYLOVA, OLGA	Russia	Nizhny Novgorod Regional Clinical Hospital named after N.A. Semashko
Golubeva, Marina	Russia	Clinical MSCh No. 1
Maryin, Dmitriy	Russia	City Clinical Hospital No. 52
Kuzmin, Alexey	Russia	Republican Clinical Oncology Dispensary
SHATOKHIN, YURIY	Russia	Rostov State Medical University

Bakirov, Bulat	Russia	Bashkir State Medical University
Loscertales Pueyo, Javier	Spain	Hospital Universitario de la Princesa
Abella Monreal, Eugenia	Spain	Hospital del Mar
Carrillo Cruz, Estrella/ SOLE	Spain	Hospital Universitario Virgen del Rocío
Bosch Albareda, Francesc	Spain	Hospital Universitario Vall d'Hebron
De La Serna Torroba Francisco, Javier	Spain	Hospital Universitario 12 de Octubre
Gonzalez Diaz, Marcos	Spain	Hosp Universitario Salamanca
Goni, Maria Angeles	Spain	Complejo Hospitalario de Navarra
Terol Castera, M. Jose	Spain	Hospital Clinico Universitario de Valencia
Lopez Martinez, Aurelio	Spain	Hospital Universitario Arnau de Vilanova
Baltasar Tello, Patricia	Spain	Hospital Universitario La Paz
Casado Montero, Luis Felipe	Spain	Hospital Virgen de la Salud
Delgado Gonzalez, Julio	Spain	Hospital Clinic i Provincial
Machado, Patricia	Spain	Hospital Universitario De Canarias
Widmer, Anouk	Switzerland	UniversitätsSpital Zürich
Gregor, Michael	Switzerland	Luzerner Kantonsspital
Andres, Martin	Switzerland	Inselspital, Universitätsspital Bern
Scott, Fiona	United Kingdom	NHS Lothian - Western General Hospital
Kallmeyer, Charlotte	United Kingdom	United Lincolnshire Hospitals NHS Trust - Lincoln County Hospital
Forconi, Francesco	United Kingdom	University Hospital Southampton NHS Foundation Trust - Southampton General Hospital
Kishore, Bhuvan	United Kingdom	Heart of England NHS Foundation Trust - Heartlands Hospital
Rinaldi, Ciro	United Kingdom	United Lincolnshire Hospitals NHS Trust Pilgrim Hospital
Pinilla-Ibarz, Javier	USA	H. Lee Moffitt Cancer Center & Research Institute
Janakiraman, Nalini	USA	Henry Ford Hospital
Heffner, Leonard (Tom)	USA	The Winship Cancer Institute of Emory University
Quick, Donald	USA	Joe Arrington Cancer Research and Treatment Center (JACC)
Kozloff, Mark	USA	Ingalls Memorial Hospital
Siddiqi, Tanya	USA	City of Hope
Kipps, Thomas	USA	University of California, San Diego (UCSD) - Moores Cancer Center
Bessudo, Alberto	USA	California Cancer Associates for Research and Excellence, Inc.
Kluеppelberg, Horst	USA	Banner MD Anderson Cancer Center

Supplementary Figure 1. Consort diagram.

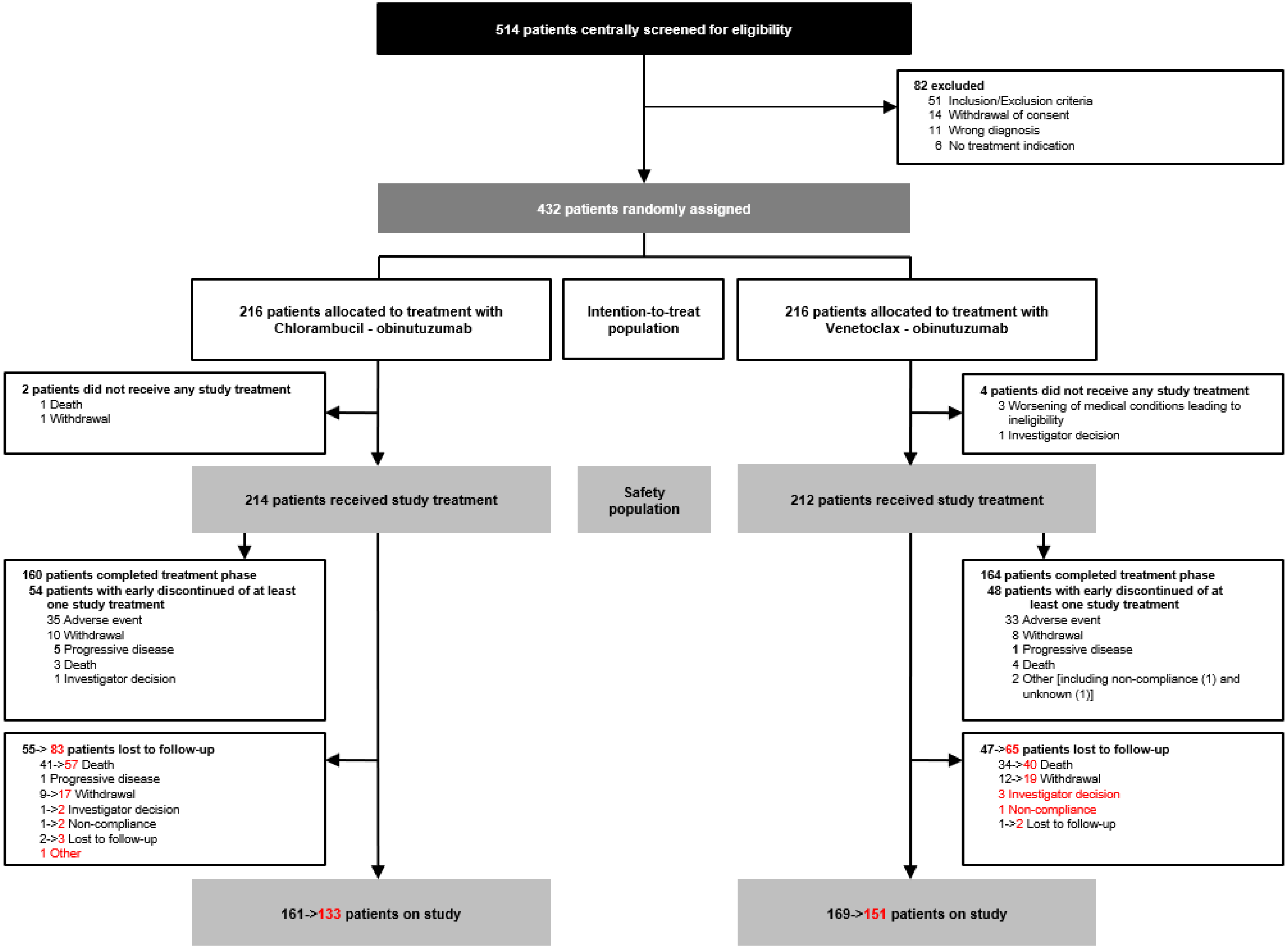

T

Supplementary Figure 2. PFS according to IGHV mutational status and TP53 aberrations.

A. Clb-Obi arm.

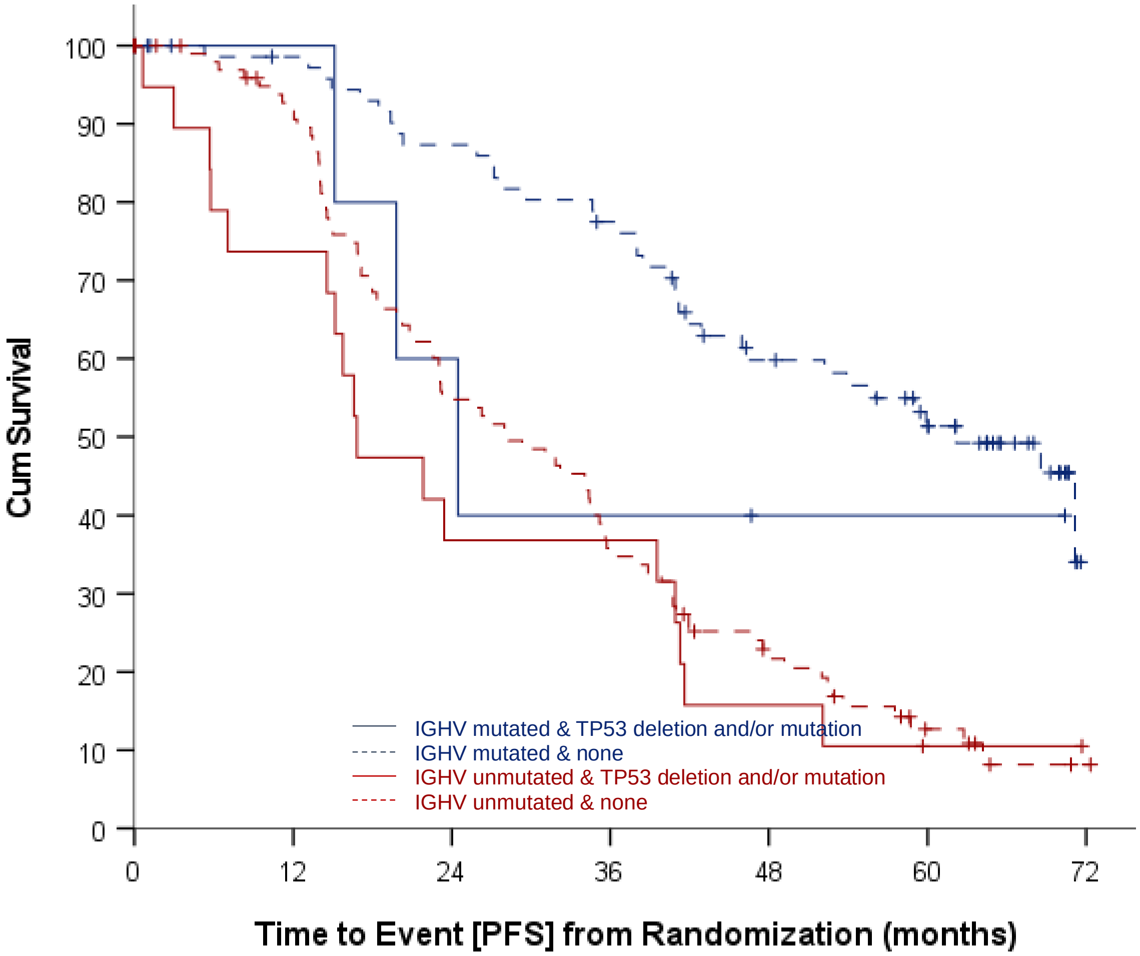

Patients at risk							
IGHV mutated & *TP53* deletion and/or mutation	5	5	3	2	1	1	0
IGHV mutated & none	77	70	62	54	38	28	0
IGHV unmutated & *TP53* deletion and/or mutation	19	14	7	7	3	1	0
IGHV unmutated & none	104	87	52	34	18	7	1

B. Ven-Obi arm.

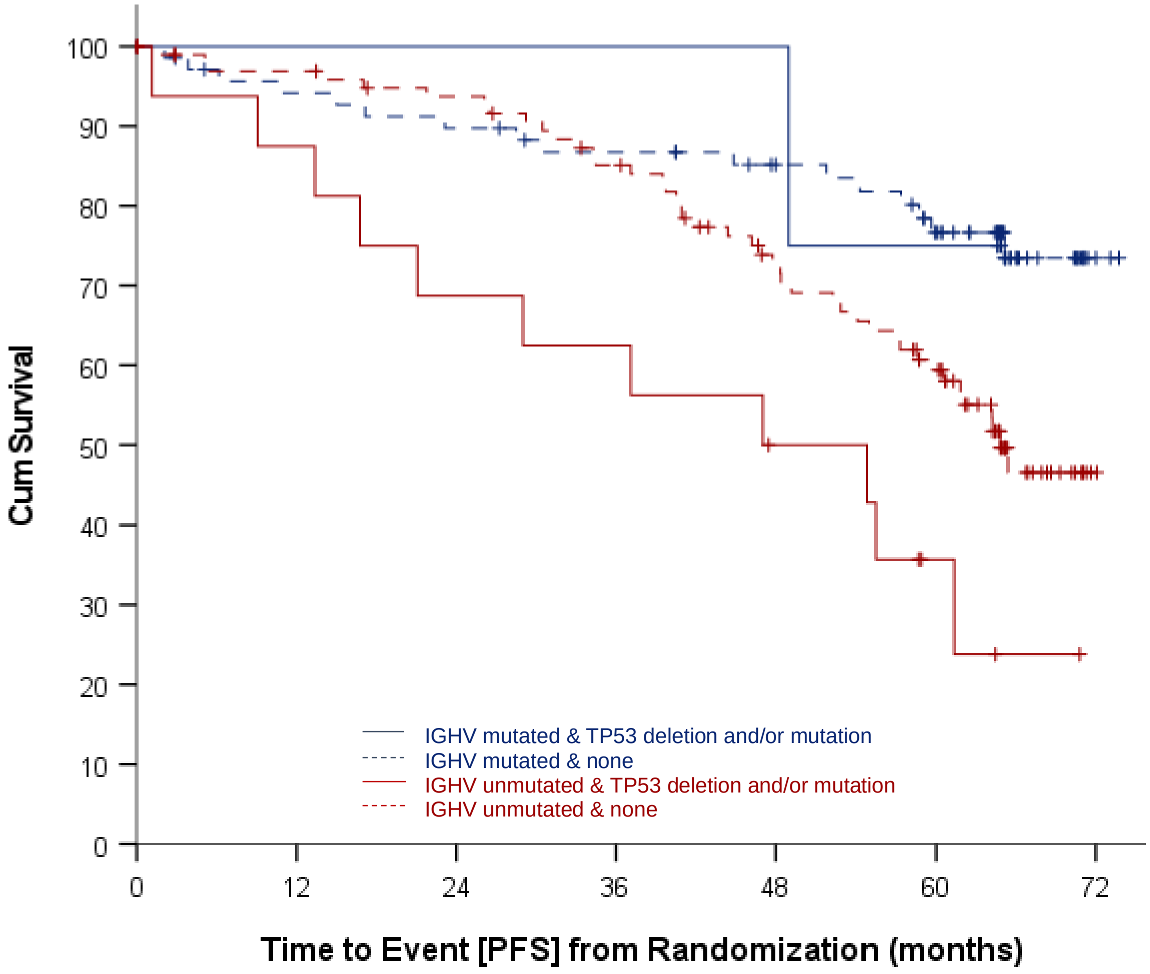

Patients at risk							
IGHV mutated & *TP53* deletion and/or mutation	5	4	4	4	4	3	0
IGHV mutated & none	71	64	61	57	51	42	3
IGHV unmutated & *TP53* deletion and/or mutation	16	14	11	10	7	2	0
IGHV unmutated & none	103	93	88	78	61	46	1

Supplementary Figure 2. TTNT (A), TTNT according to TP53 aberrations (B) and IGHV mutational status (C).
A

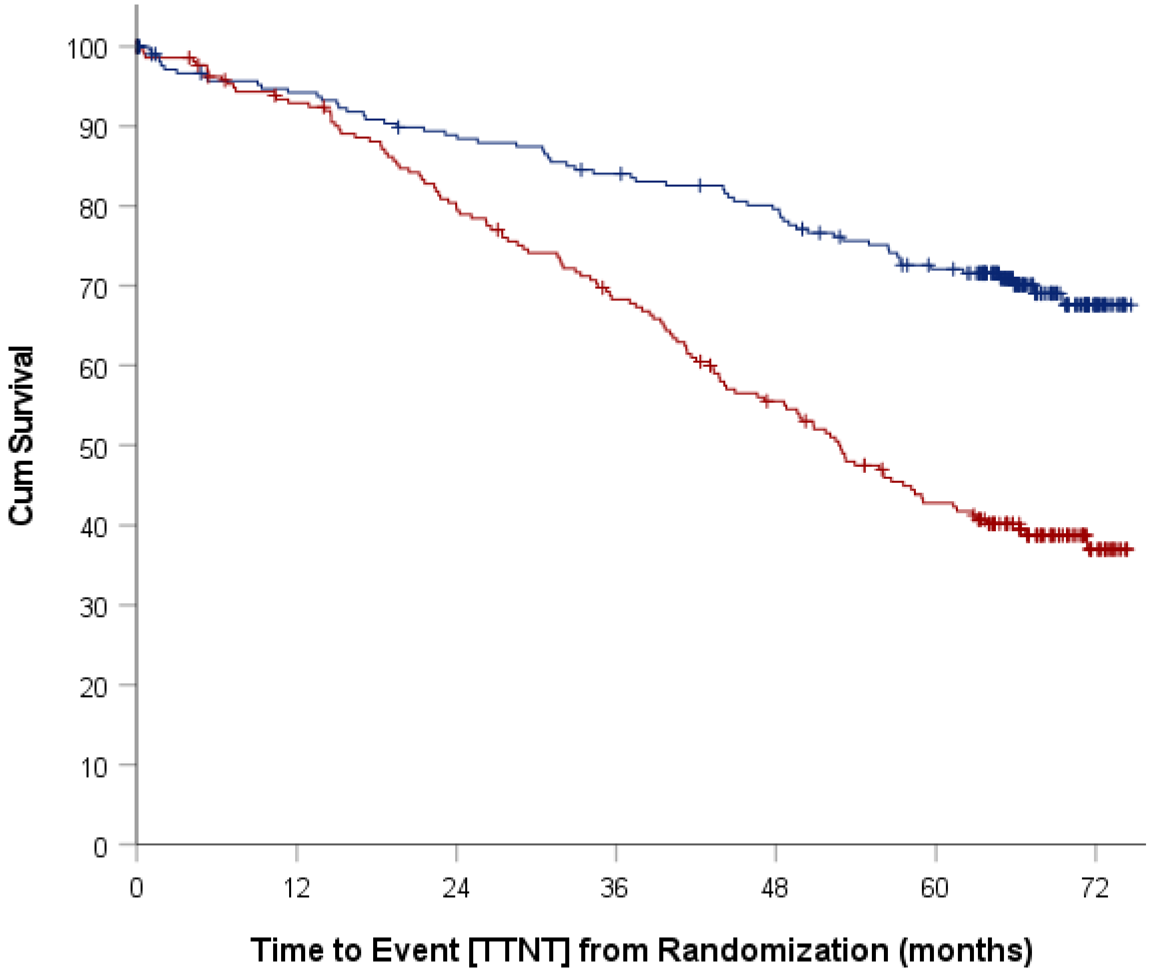

Patients at risk							
Ven-Obi	216	195	183	172	161	140	22
Clb-Obi	216	194	166	140	111	83	17

B

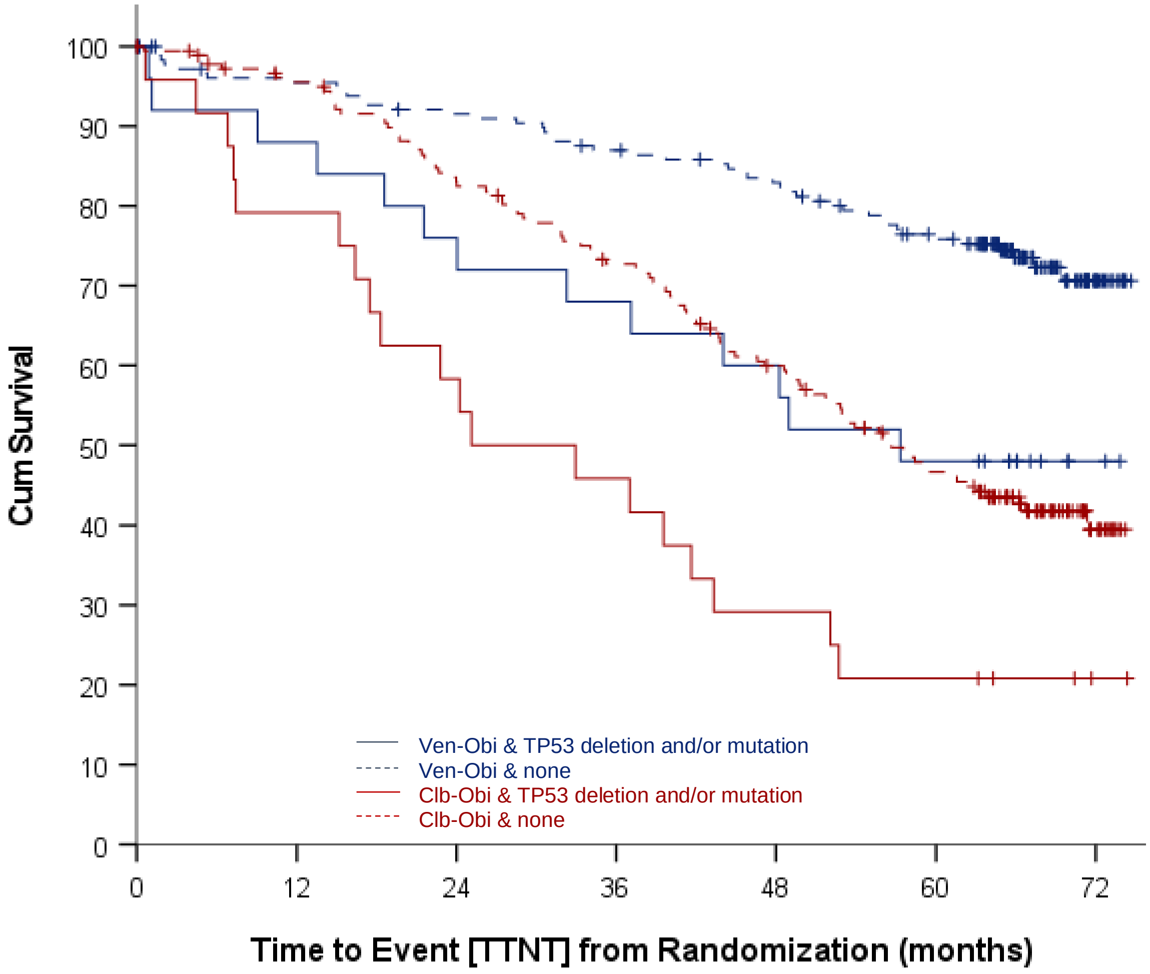

Patients at risk							
Ven-Obi & *TP53* deletion and/or mutation	25	22	19	17	15	12	2
Ven-Obi & none	184	169	161	152	143	125	19
Clb-Obi & *TP53* deletion and/or mutation	24	19	14	11	7	5	1
Clb-Obi & none	184	169	146	126	101	76	14

C

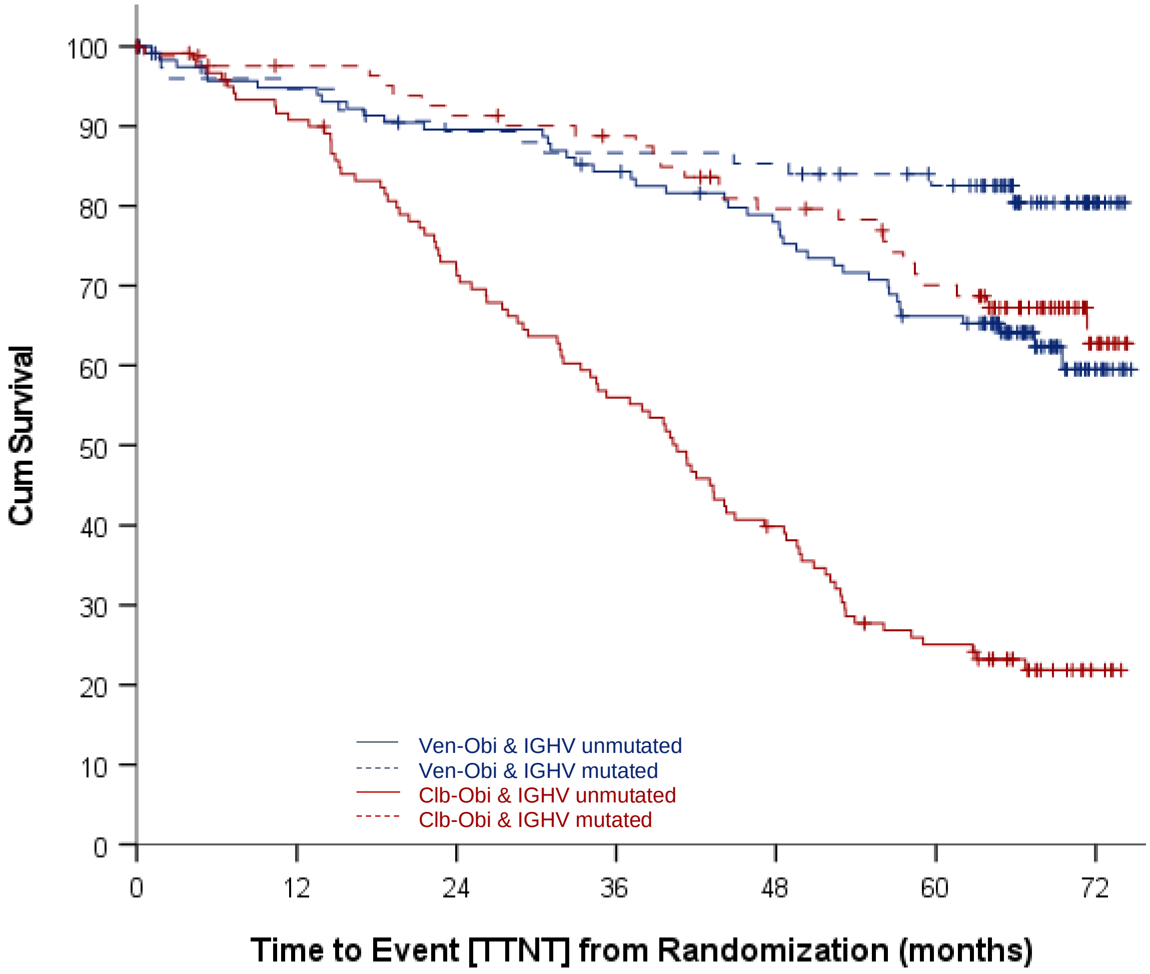

Patients at risk							
Ven-Obi & IGHV unmutated	121	109	102	95	86	72	11
Ven-Obi & IGHV mutated	76	71	67	65	64	57	10
Clb-Obi & IGHV unmutated	123	108	85	66	46	28	4
Clb-Obi & IGHV mutated	83	78	73	69	60	51	12

Supplementary Figure 4. OS according to TP53 aberrations (A) and IGHV mutational status (B).

A

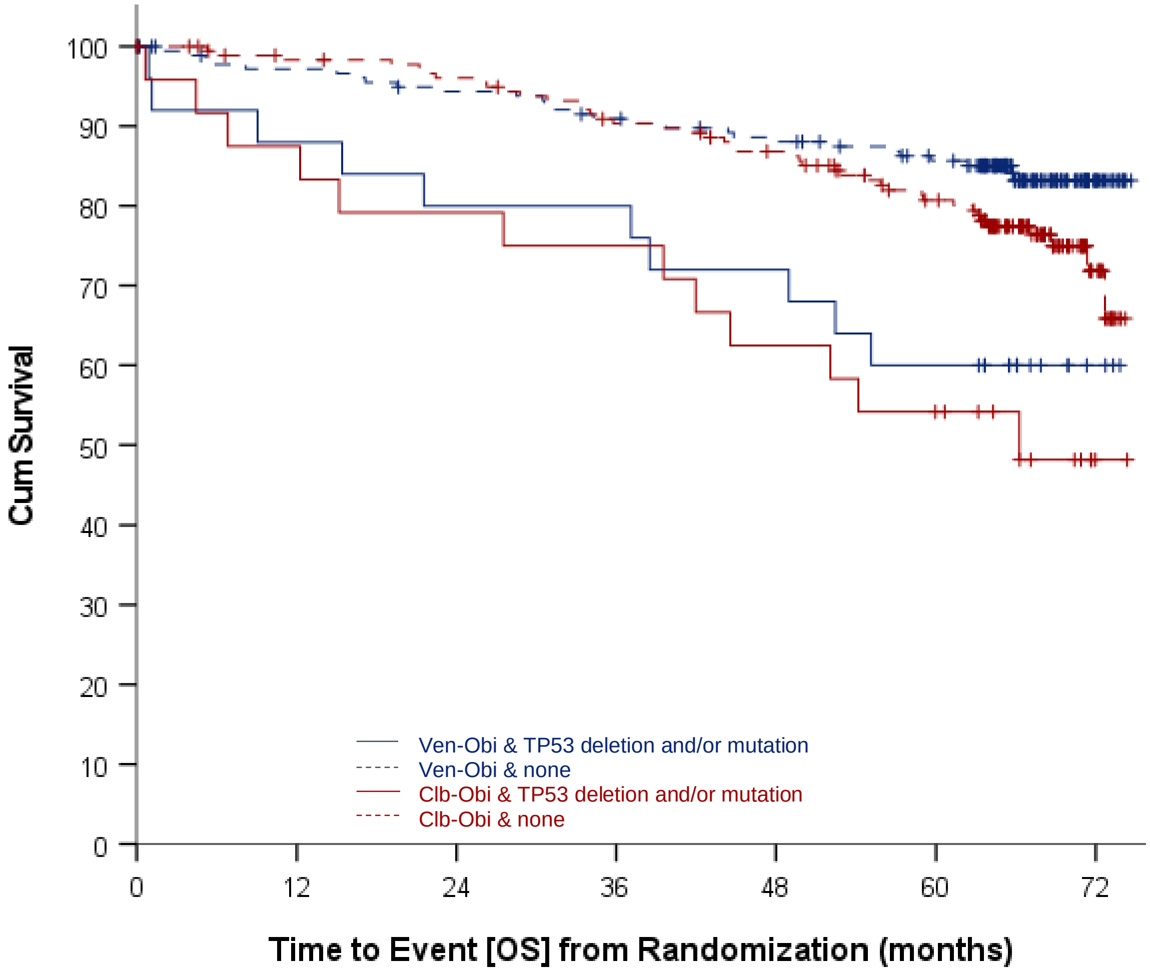

**Patients at risk**							
Ven-Obi & *TP53* deletion and/or mutation	25	22	20	20	18	15	3
Ven-Obi & none	184	172	166	159	152	141	21
Clb-Obi & *TP53* deletion and/or mutation	24	21	19	18	15	12	1
Clb-Obi & none	184	174	169	157	147	127	18

B

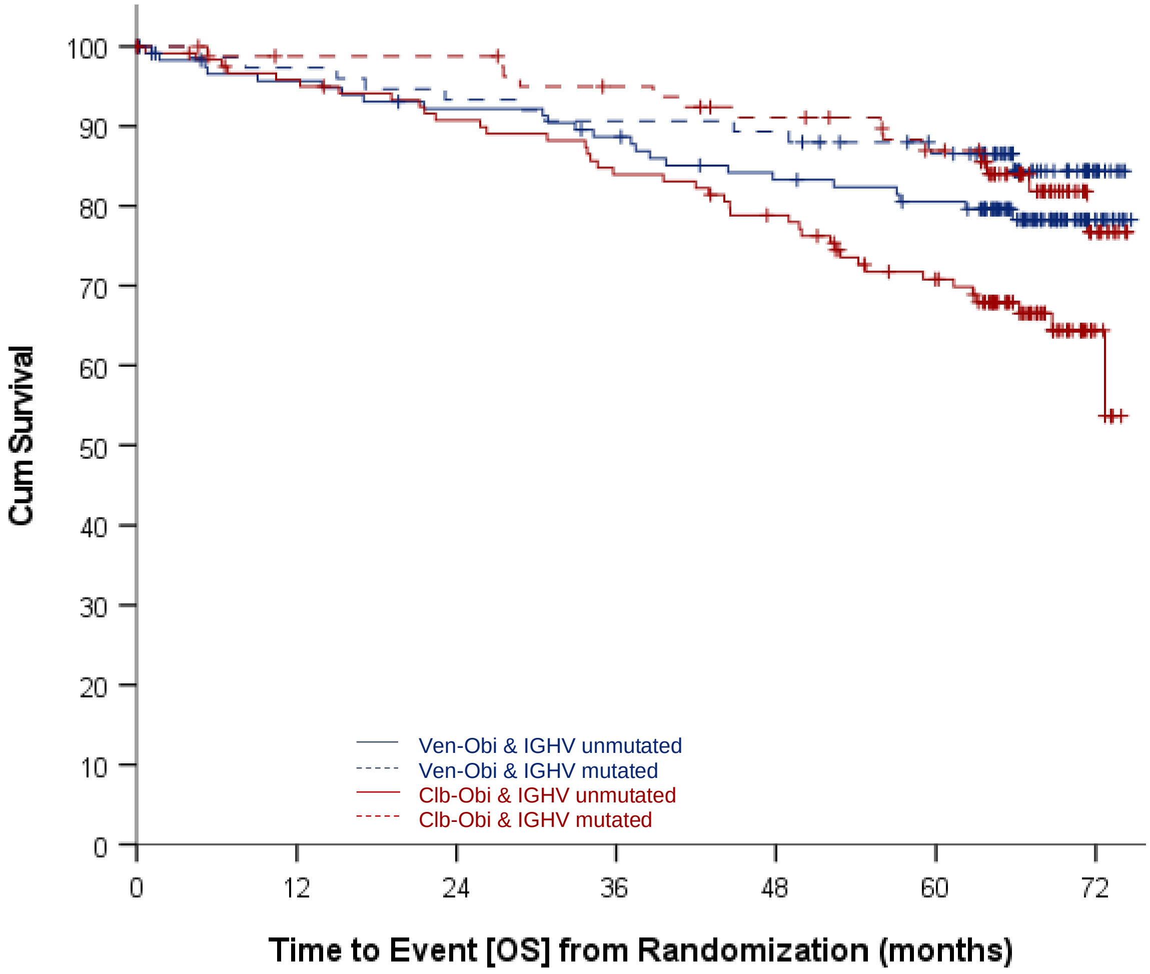

**Patients at risk**							
Ven-Obi & IGHV unmutaed	121	110	105	100	92	87	14
Ven-Obi & IGHV mutated	76	73	70	68	67	60	10
Clb-Obi & IGHV unmutaed	123	114	107	99	91	75	7
Clb-Obi & IGHV mutated	83	79	79	74	69	62	12

Supplementary Figure 5. Time to Second-primary malignancy.
P-values derived from two-sided Gray's test.

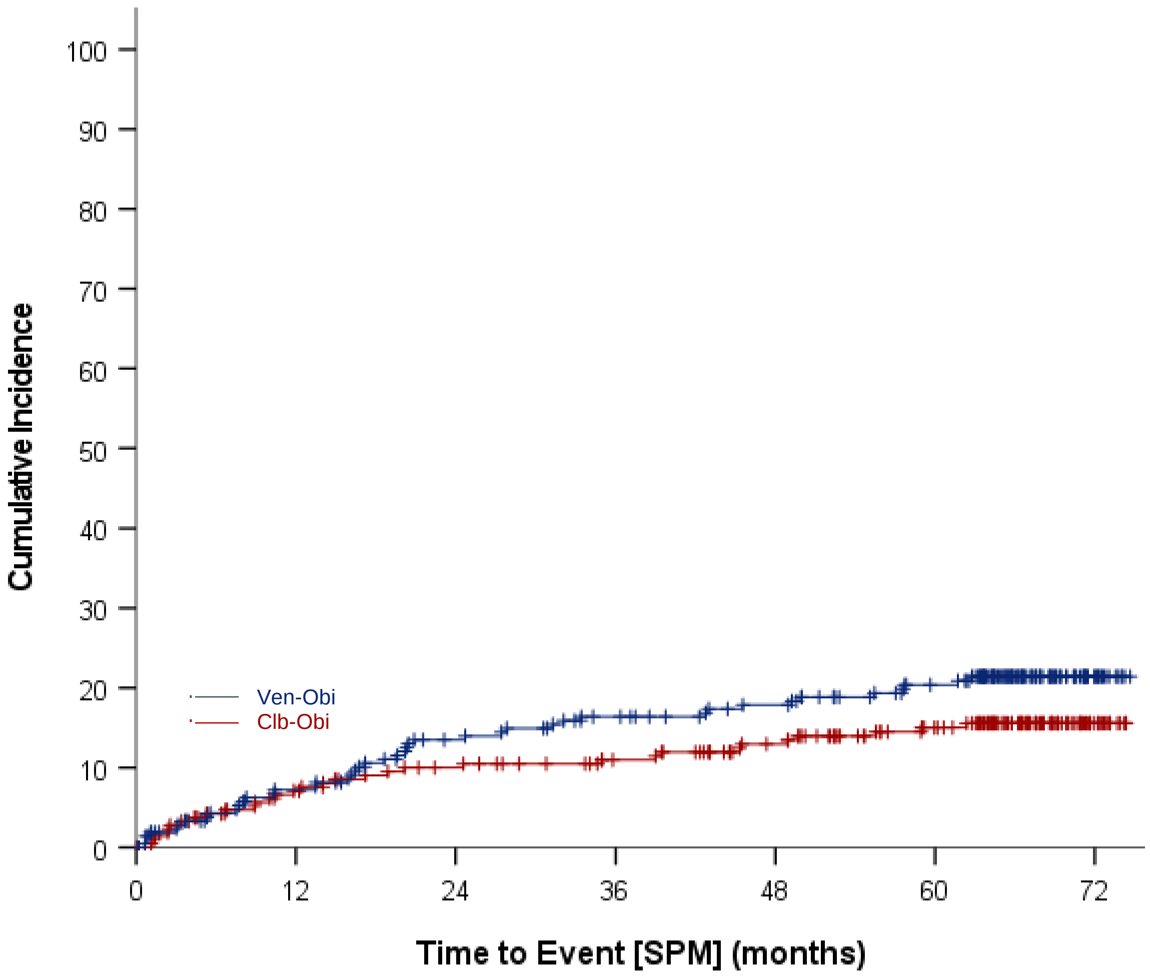

Patients at risk							
Ven-Obi	212	196	180	170	160	143	22
Clb-Obi	214	201	189	176	161	136	20

Gray's Test for Equality of Cumulative Incidence Functions	Univariate comparison	Chi-Square	*p*-value
SPM incidence **and treatment**			
Ven-Obi	***vs.*** Clb-Obi	3.18635	0.0743

Supplementary Figure 6. MRD rates according to available samples.

A Ven-Obi

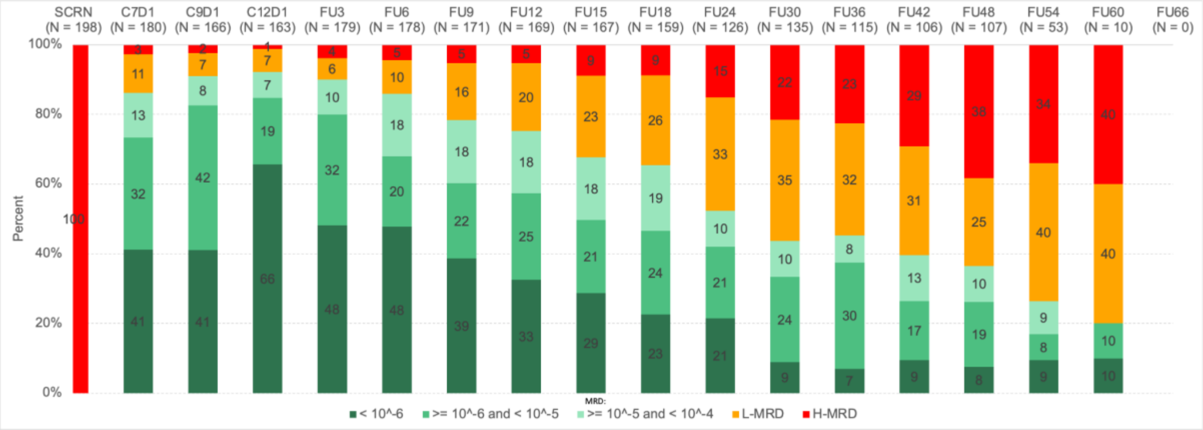

B Clb-Obi

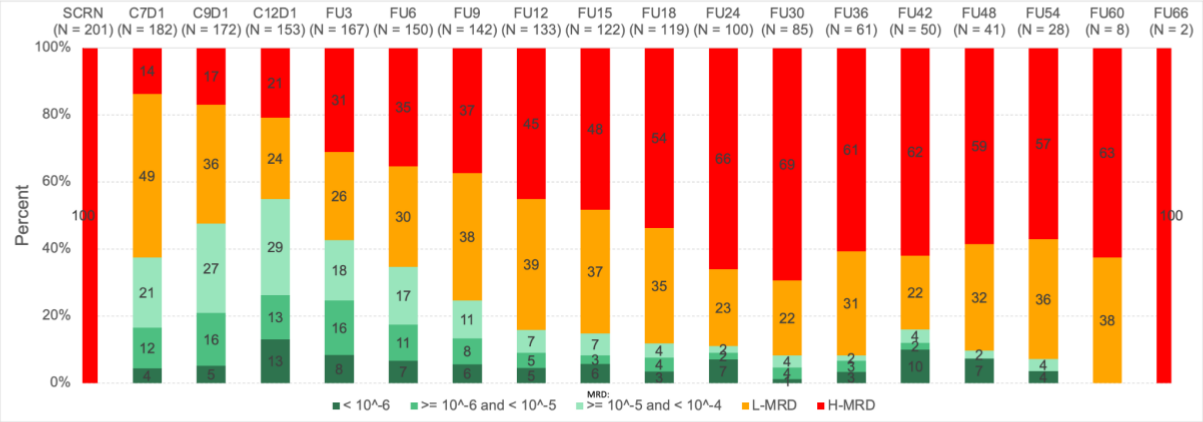

Supplementary Figure 7. Time to MRD conversion (≥10^{-4}) by NGS from end of treatment.

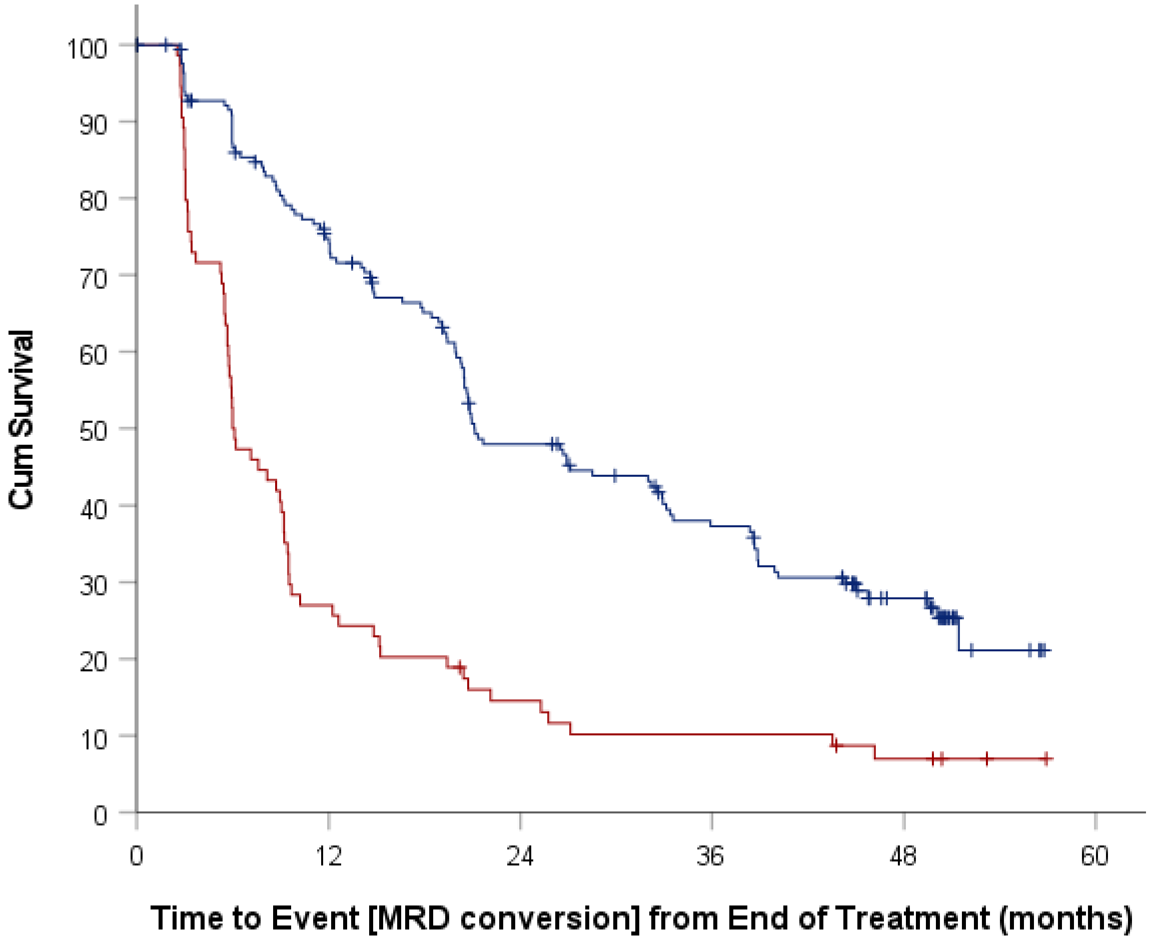

Patients at risk						
Ven-Obi	169	118	72	51	25	0
Clb-Obi	74	20	10	7	4	0

Supplementary Figure 8. Sample selection for RNAseq sub-study.

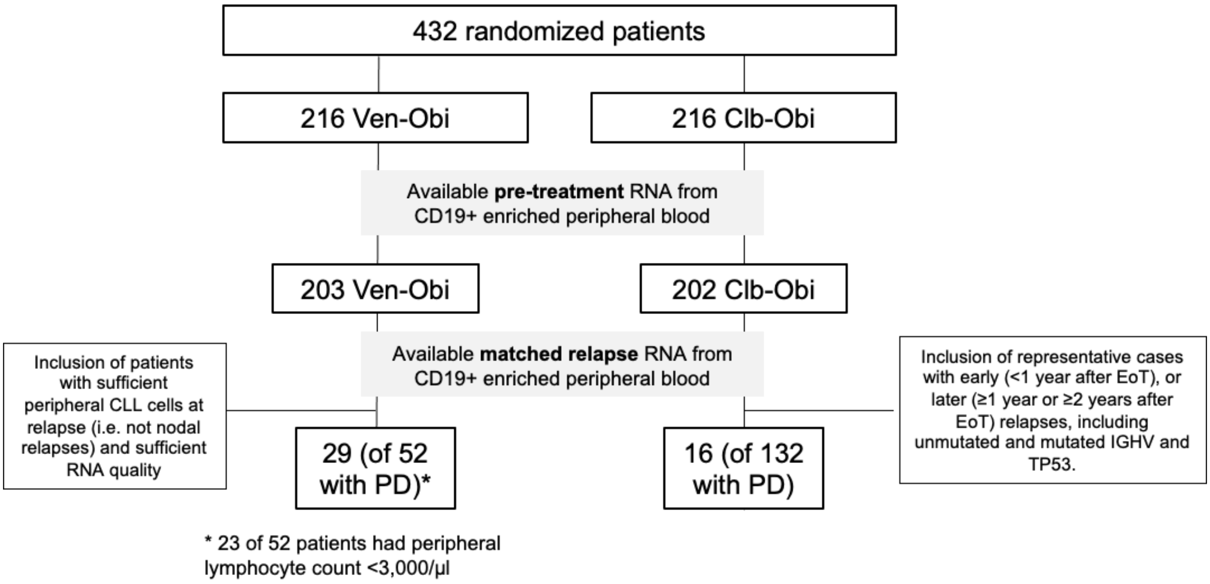

Supplementary Figure 9. Pre-treatment transcriptomic SNN clustering and UMAP visualization.

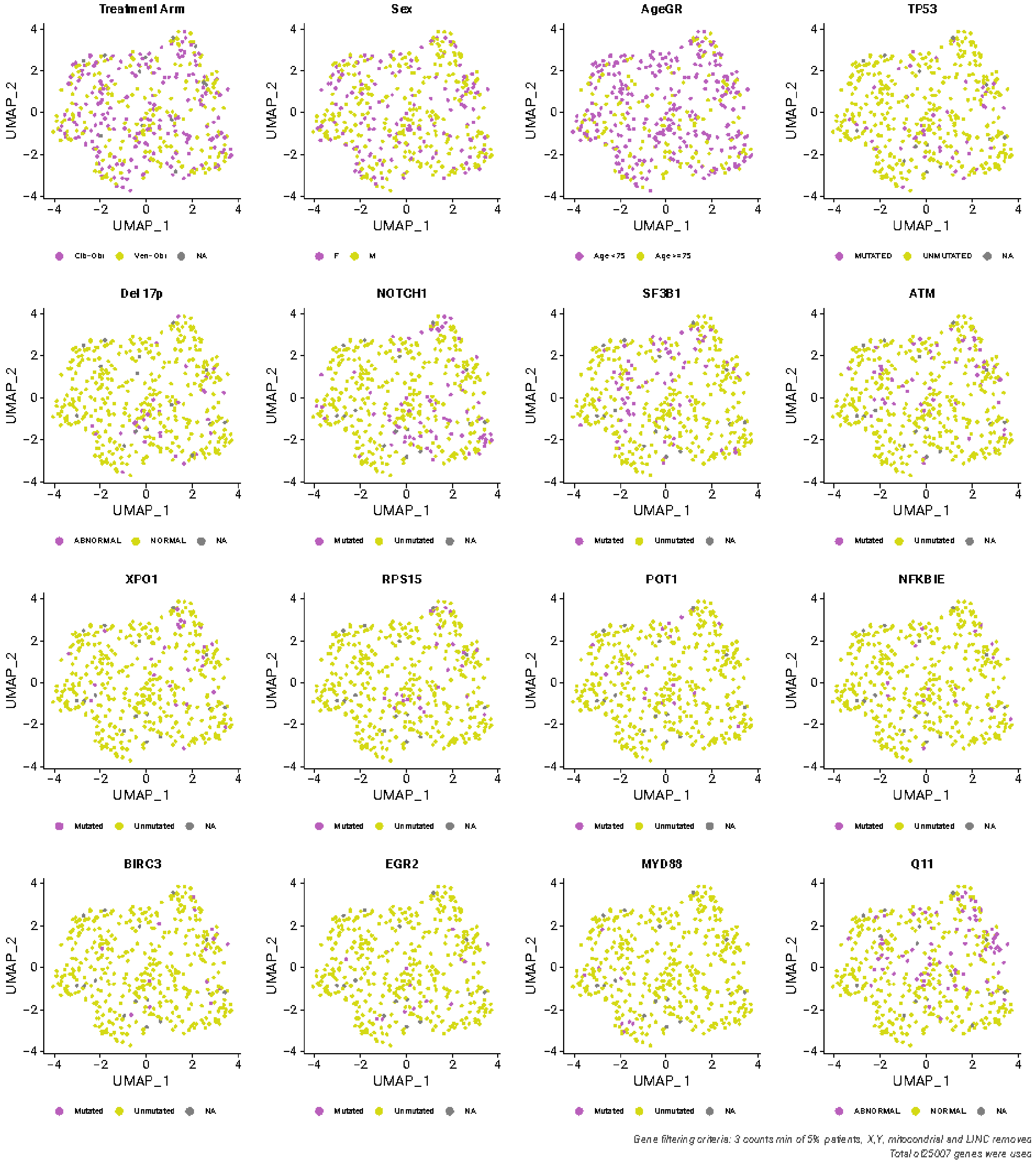

Supplementary Figure 10. Differential gene expression between patients with uMRD (<10^{-4}) and MRD+ (≥10^{-4}).

P- values from moderated two-sided t-test without adjusting for multiple testing.

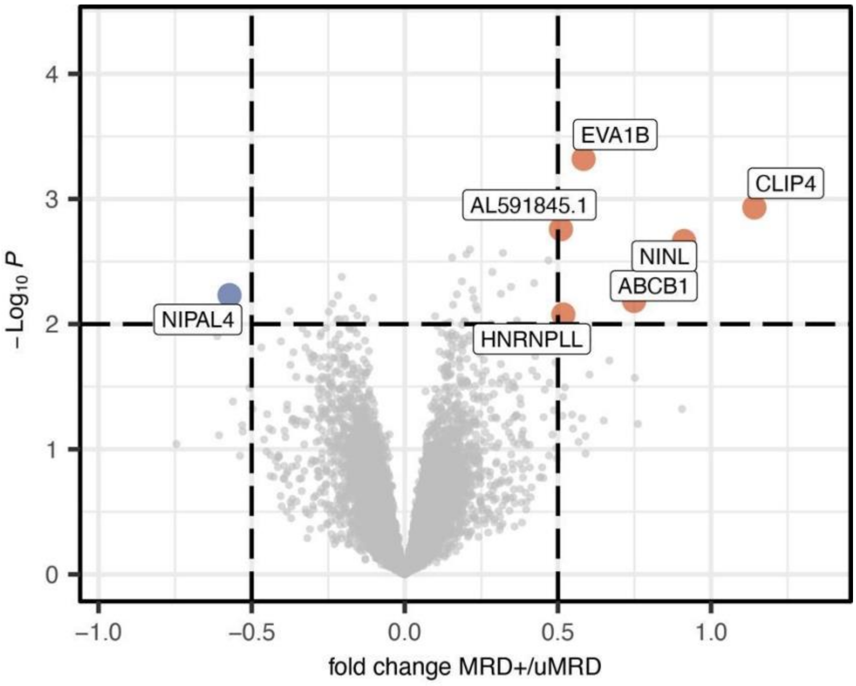

Supplementary Figure 11. Gene set enrichment analysis and differential gene expression between baseline and relapse

(A) P- values from moderated two-sided t-test without adjusting for multiple testing. (B) P-values derived from non-parametric permutation test and adjusted for multiple testing using the Benjamini-Hochberg procedure.

A Differential gene expression

Clb-Obi

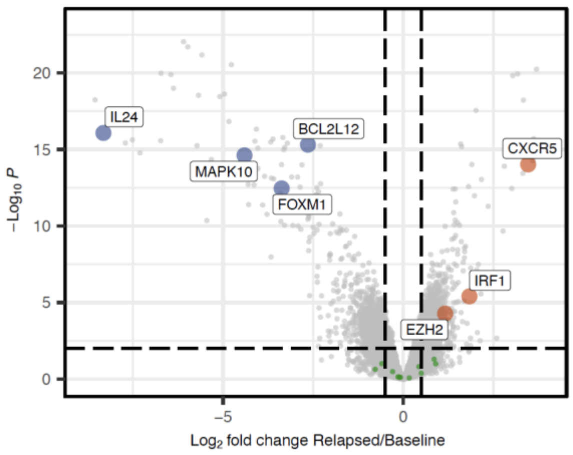

Ven-Obi

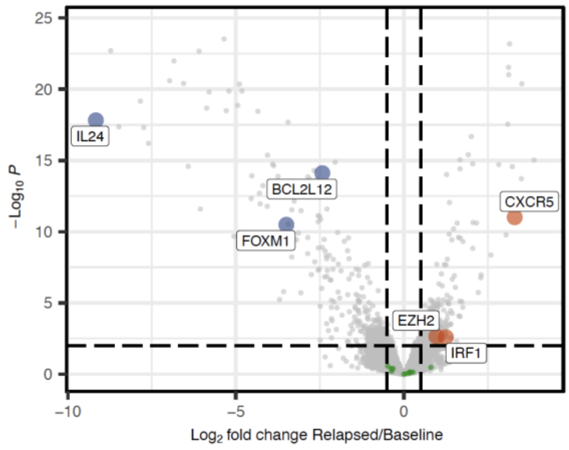

B Gene set enrichment analysis

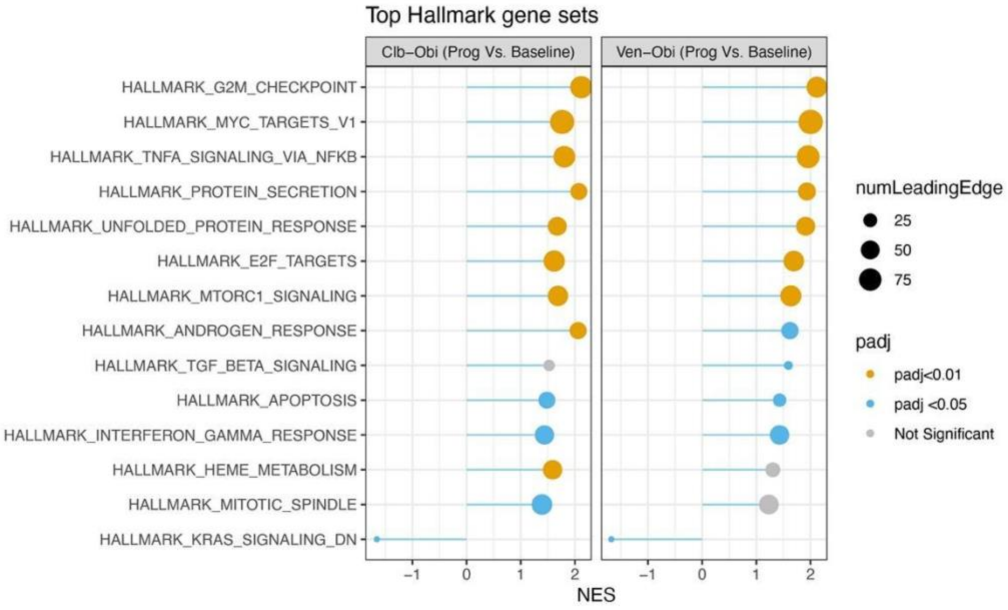

Supplementary Figure 12. Deconvolution of cell type fractions by CIBERSORTx.

X-axis indicates one sample per column, y-axis indicates fraction of cells.

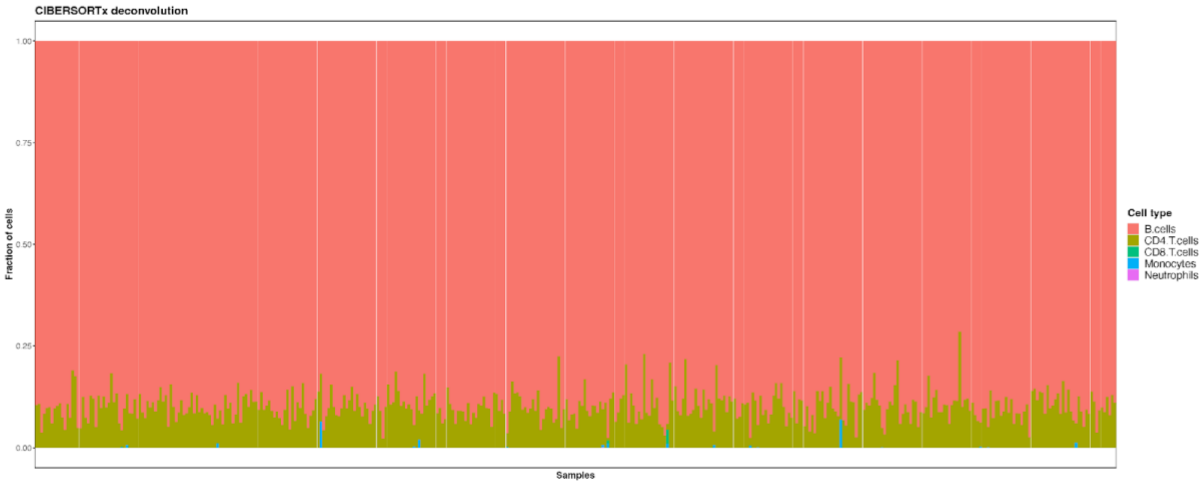

Supplementary References

1. Dohner, H., *et al.* Genomic aberrations and survival in chronic lymphocytic leukemia. *The New England journal of medicine* **343**, 1910-1916 (2000).

CONSORT 2010 checklist of information to include when reporting a randomised trial

Section/Topic	Item No	Checklist item	Reported on page No
**Title and abstract**			
	1a	Identification as a randomised trial in the title	1
	1b	Structured summary of trial design, methods, results, and conclusions (for specific guidance see CONSORT for abstracts)	1
**Introduction**			
Background and objectives	2a	Scientific background and explanation of rationale	2
	2b	Specific objectives or hypotheses	3
**Methods**			
Trial design	3a	Description of trial design (such as parallel, factorial) including allocation ratio	17-18
	3b	Important changes to methods after trial commencement (such as eligibility criteria), with reasons	Not applicable
Participants	4a	Eligibility criteria for participants	18
	4b	Settings and locations where the data were collected	19-20
Interventions	5	The interventions for each group with sufficient details to allow replication, including how and when they were actually administered	18
Outcomes	6a	Completely defined pre-specified primary and secondary outcome measures, including how and when they were assessed	19
	6b	Any changes to trial outcomes after the trial commenced, with reasons	Not applicable
Sample size	7a	How sample size was determined	22
	7b	When applicable, explanation of any interim analyses and stopping guidelines	Not applicable
Randomisation:			
Sequence generation	8a	Method used to generate the random allocation sequence	19
	8b	Type of randomisation; details of any restriction (such as blocking and block size)	19

Allocation concealment mechanism	9	Mechanism used to implement the random allocation sequence (such as sequentially numbered containers), describing any steps taken to conceal the sequence until interventions were assigned	19
Implementation	10	Who generated the random allocation sequence, who enrolled participants, and who assigned participants to interventions	19
Blinding	11a	If done, who was blinded after assignment to interventions (for example, participants, care providers, those assessing outcomes) and how	Not applicable
	11b	If relevant, description of the similarity of interventions	Not applicable
Statistical methods	12a	Statistical methods used to compare groups for primary and secondary outcomes	22
	12b	Methods for additional analyses, such as subgroup analyses and adjusted analyses	23
**Results**			
Participant flow (a diagram is strongly recommended)	13a	For each group, the numbers of participants who were randomly assigned, received intended treatment, and were analysed for the primary outcome	4, 7
	13b	For each group, losses and exclusions after randomisation, together with reasons	19 (Supplementary Material)
Recruitment	14a	Dates defining the periods of recruitment and follow-up	4
	14b	Why the trial ended or was stopped	Not applicable
Baseline data	15	A table showing baseline demographic and clinical characteristics for each group	29-30
Numbers analysed	16	For each group, number of participants (denominator) included in each analysis and whether the analysis was by original assigned groups	32, 35-40
Outcomes and estimation	17a	For each primary and secondary outcome, results for each group, and the estimated effect size and its precision (such as 95% confidence interval)	4-7
	17b	For binary outcomes, presentation of both absolute and relative effect sizes is recommended	Not applicable
Ancillary analyses	18	Results of any other analyses performed, including subgroup analyses and adjusted analyses, distinguishing pre-specified from exploratory	5-7, 8-13
Harms	19	All important harms or unintended effects in each group (for specific guidance see CONSORT for harms)	7-8
**Discussion**			

Limitations	20	Trial limitations, addressing sources of potential bias, imprecision, and, if relevant, multiplicity of analyses	15-17
Generalisability	21	Generalisability (external validity, applicability) of the trial findings	13-17
Interpretation	22	Interpretation consistent with results, balancing benefits and harms, and considering other relevant evidence	13-17
**Other information**			
Registration	23	Registration number and name of trial registry	18
Protocol	24	Where the full trial protocol can be accessed, if available	18
Funding	25	Sources of funding and other support (such as supply of drugs), role of funders	27

PROTOCOL

**TITLE:**	**A PROSPECTIVE, OPEN-LABEL, MULTICENTER RANDOMIZED PHASE III TRIAL TO COMPARE THE EFFICACY AND SAFETY OF A COMBINED REGIMEN OF OBINUTUZUMAB AND VENETOCLAX (GDC-0199/ABT-199) VERSUS OBINUTUZUMAB AND CHLORAMBUCIL IN PREVIOUSLY UNTREATED PATIENTS WITH CLL AND COEXISTING MEDICAL CONDITIONS**
**PROTOCOL NUMBER:**	BO25323/CLL14
**VERSION NUMBER:**	7
**EUDRACT NUMBER:**	2014-001810-24
**IND NUMBER:**	110159
**TEST PRODUCTS:**	Venetoclax (GDC-0199 [ABT-199]; RO5537382), Obinutuzumab (GA101; RO5072759), and Chlorambucil
**MEDICAL MONITOR:**	Maneesh Tandon, MB ChB
**SPONSOR:**	F. Hoffmann-La Roche Ltd in collaboration with the German Chronic Lymphocytic Leukaemia Study Group
**CO-SPONSOR (United States only):**	AbbVie, Inc.
**DATE FINAL:**	Version 1: July 23, 2014
**DATES AMENDED:**	Version 2: October 21, 2014<br>Version 3: November 7, 2014<br>Version 4: May 21, 2015<br>Version 5: November 2, 2015<br>Version 6: March 29, 2017<br>Version 7: See electronic date stamp below

PROTOCOL AMENDMENT APPROVAL

Approver's Name	Title	Date and Time (UTC)
Mobasher, Mehrdad (mobasher	Company Signatory	12-Feb-2018 18:28:50

CONFIDENTIAL

This clinical study is being sponsored globally by F. Hoffmann-La Roche Ltd of Basel, Switzerland. However, it may be implemented in individual countries by Roche's local affiliates, including Genentech, Inc. in the United States. The information contained in this document, especially any unpublished data, is the property of F. Hoffmann-La Roche Ltd (or under its control) and therefore is provided to you in confidence as an investigator, potential investigator, or consultant, for review by you, your staff, and an applicable Ethics Committee or Institutional Review Board. It is understood that this information will not be disclosed to others without written authorization from Roche except to the extent necessary to obtain informed consent from persons to whom the drug may be administered.

PROTOCOL AMENDMENT, VERSION 7: RATIONALE

Protocol BO25323 has been amended to include the option to add an additional earlier interim efficacy analysis to mitigate the potential for an undue delay in the delivery of the study read-out; to prolong collection of blood samples to characterize the minimal residual disease kinetics in patients who might be responding longer than originally expected; and to add complete response as a stand-alone secondary endpoint in order to more comprehensively explore the potential depth of response to study treatment. Clarifications have been made regarding the time windows for assessments in the study and timing of blood sampling for safety monitoring/B-cell recovery. Additionally, an inconsistency in the reporting instructions for unrelated serious adverse events has been removed.

Changes to the protocol, along with a rationale for each change, are summarized below:

- The option to add an earlier interim analysis for efficacy has been added to reflect accumulating data, including minimal residual disease (MRD) response rate data from the 12 patients in the safety run-in portion of the study (Fischer et al. 2017) and early-phase Study GP28331 in front-line chronic lymphocytic leukemia (CLL) patients (Flinn et al. 2017) who have all been treated with the obinutuzumab and venetoclax regimen. Data from these patients have indicated levels of response that are anticipated to translate into longer progression-free survival (PFS), such that events of disease progression are taking longer to accrue than originally projected in the study protocol. As a result of these preliminary findings, it is expected that the original interim analysis might be significantly delayed, and the statistical analysis for the study has now been modified to introduce a possible additional earlier interim efficacy analysis. The early additional interim analysis may be conducted at or after 1 year after the last patient's last venetoclax dose (i.e., Month 37 of the study [August 2018]), provided that at least 85 PFS events (50% of the total of 170 PFS events) have occurred. If 85 PFS events have not been observed by 1 year after the last patient's last venetoclax dose, then the interim analysis will be conducted once a minimum of 85 PFS events have occurred. PFS will be tested such that the overall two-sided type I error rate will be maintained at the 0.05 level. The study would only stop for efficacy if a treatment effect HR of 0.35 or better is observed when the interim analysis is based on 85 events. This approach is designed to mitigate the potential for a protracted waiting period for the necessary PFS events to accrue, which would result in an undue delay in the study read-out and availability of a potential new treatment option for patients with chronic lymphocytic leukemia (CLL) (Section 6.11).
- The wording of the original interim analysis text has been changed to simplify the protocol and will be done when a minimum of 110 PFS events have occurred now that it is clear that 128 events will not have been observed by 28th February 2018. There is no change to the timing of this analysis (Section 6.11).

- The duration of the collection of blood samples for MRD assessments by ASO-PCR and NGS has been extended to beyond 18 months after treatment until 5 years after last patient enrolled to reflect the MRD response rate data from the 12 patients in the safety run-in portion of the study in order to enable characterization of the MRD kinetics in patients who might be responding longer than originally projected (Appendix 3 and Appendix 13).An additional secondary endpoint of complete response has been added to more comprehensively explore the potential depth of response to study treatment in a clinically meaningful manner and further differniate response beyond ORR (Section 3.4.2 and Section 6.4.2).
- The time windows for study assessments have been aligned with Section 4.5.10, such that all assessments during the treatment period and follow-up Day 28 visit should be performed within 7 days of the scheduled visit, unless otherwise specified. On treatment days, all assessments should be performed prior to dosing, unless otherwise specified. Following the end-of-treatment assessment (3 months after treatment completion/early termination), all other follow-up assessments, whether tumor assessments or other study assessments, will be done within ±14 days for 3-monthly and within a 1 month for 6-monthly assessments of the scheduled visits (Appendix 1 and Appendix 3).
- Wording for blood sampling for safety monitoring/B-cell recovery has been clarified for consistency with Section 4.5.14 throughout the protocol. Timing of samples after treatment completion/early termination has been clarified to be required at 28 days; 12, 18, and 24 months; and then every 6 months until 5 years from last patient enrolled (Appendix 2 and Appendix 3).
- A prior inconsistency in the reporting period for serious adverse events considered by the investigator to be unrelated to study medication has been removed. There is now a reference to Appendix 14 for details of the duration and reporting of adverse events in Section 5.4.2.2.

Substantive new information appears in italics. This amendment represents cumulative changes to the original protocol.

Additional minor changes have been made to improve clarity and consistency.

PROTOCOL SIGNATURE PAGE

PROTOCOL VERSION 7
CLL14 STUDY OF THE GCLLSG
(BO25323)

A prospective, open-label, multicenter randomized phase III trial to compare the efficacy and safety of a combined regimen of obinutuzumab and venetoclax (GDC-0199 [ABT-199]) versus obinutuzumab and chlorambucil in previously untreated patients with CLL and coexisting medical conditions

Signatures were collected based on Version 7 of this protocol provided to GCLLSG on 6 February 2018. No modifications to the protocol have been made since this date except for the final electronic publishing of the protocol in the Roche system. The final date of Version 7 of the protocol is provided in the electronic date stamp on the title page; the version number remains unchanged.

Head of the German CLL Study Group, and Principal Investigator of CLL14:

Prof. Dr. Michael Hallek
Dept Internal Medicine I,
Cologne University Hospital

M. Hallek

Signature

09. FEB. 2018

Date

Statistician of the German CLL Study Group:

Dr. Jasmin Bahlo
Dept Internal Medicine I,
Cologne University Hospital

Signature

Date

Head of the German CLL Study Office, and Coordinating Physician:

Dr. Kirsten Fischer
Dept Internal Medicine I,
Cologne University Hospital

Signature

Date

PROTOCOL SIGNATURE PAGE

PROTOCOL VERSION 7
CLL14 STUDY OF THE GCLLSG
(BO25323)

A prospective, open-label, multicenter randomized phase III trial to compare the efficacy and safety of a combined regimen of obinutuzumab and venetoclax (GDC-0199 [ABT-199]) versus obinutuzumab and chlorambucil in previously untreated patients with CLL and coexisting medical conditions

Signatures were collected based on Version 7 of this protocol provided to GCLLSG on 6 February 2018. No modifications to the protocol have been made since this date except for the final electronic publishing of the protocol in the Roche system. The final date of Version 7 of the protocol is provided in the electronic date stamp on the title page; the version number remains unchanged.

Head of the German CLL Study Group, and Principal Investigator of CLL14:

Prof. Dr. Michael Hallek
Dept Internal Medicine I,
Cologne University Hospital

Signature ______________________ Date ______________

Statistician of the German CLL Study Group:

Dr. Jasmin Bahlo
Dept Internal Medicine I,
Cologne University Hospital

Signature ______________________ Date 06.02.2018

Head of the German CLL Study Office, and Coordinating Physician:

Dr. Kirsten Fischer
Dept Internal Medicine I,
Cologne University Hospital

Signature ______________________ Date 06.02.2018

TABLE OF CONTENTS

PROTOCOL AMENDMENT ACCEPTANCE FORM 14

PROTOCOL SYNOPSIS 15

1. BACKGROUND 27

1.1 Background on Chronic Lymphocytic Leukemia 27

1.2 Background on Venetoclax (GDC-0199) 29

1.3 Study Rationale and Benefit-Risk Assessment 29

1.3.1 Efficacy 29

1.3.2 Safety 30

1.3.3 Rationale for the Combination of Venetoclax with Obinutuzumab 31

1.3.4 Benefit-Risk Assessment 32

2. OBJECTIVES 32

2.1 Efficacy Objectives 32

2.2 Safety Objectives 32

2.3 Pharmacokinetic Objective 33

2.4 Patient-Reported Outcome Objectives 33

2.5 Health Economic Objective 33

2.6 Exploratory Objectives 33

3. STUDY DESIGN 34

3.1 Description of Study 34

3.1.1 Assessment of Efficacy 34

3.1.2 Summary of Study Procedures 35

3.1.2.1 Screening/Baseline Tests 35

3.1.2.2 Treatment Period 36

3.1.2.3 Monitoring of Disease Progression during Treatment Period 37

3.1.2.4 Post-Treatment Follow-Up Visits 38

3.2 End of Study 38

3.3 Rationale For Design 38

3.3.1 Rationale for Dose and Schedule 38

3.3.1.1 Venetoclax 38
3.3.1.2 Obinutuzumab 39
3.3.1.3 Chlorambucil 39
3.3.2 Rationale for the Combination of Venetoclax with Obinutuzumab 40
3.3.3 Rationale for Patient Population 40
3.3.4 Rationale for Control Group 41
3.3.5 Rationale for Minimal Residual Disease Assessments 42
3.3.6 Rationale for Biomarker Assessments 42
3.3.7 Rationale for Chronic Lymphocytic Leukemia Diagnostic Laboratory Assessments 43
3.3.8 Rationale for Pharmacokinetic Assessments 44
3.3.9 Rationale for Patient-Reported Outcome Assessments 44
3.4 Outcome Measures 45
3.4.1 Efficacy Outcome Measures 45
3.4.2 Secondary Efficacy Outcome Measures 45
3.4.3 Safety Outcome Measures 46
3.4.4 Pharmacokinetic Outcome Measures 46
3.4.5 Pharmacodynamic Outcome Measures 46
3.4.6 Patient-Reported Outcome Measures 46
3.4.7 Health Economic Outcome Measure 47
3.4.8 Exploratory Outcome Measures 47
4. MATERIALS AND METHODS 47
4.1 Patients 47
4.1.1 Inclusion Criteria 47
4.1.2 Exclusion Criteria 49
4.2 Method of Treatment Assignment and Blinding 50
4.3 Study Treatment 51
4.3.1 Formulation, Packaging, and Handling 51
4.3.1.1 Venetoclax 51
4.3.1.2 Chlorambucil 51
4.3.1.3 Obinutuzumab 51

4.3.2 Dosage, Administration, and Compliance 52
4.3.3 Investigational Medicinal Products 59
4.3.4 Non-Investigational Medicinal Products................................. 59
4.3.5 Post-Trial Access to Venetoclax 60
4.4 Concomitant Therapy 60
4.4.1 Permitted Therapy 60
4.4.2 Prohibited Therapy 60
4.4.2.1 Drug–Drug Interaction 61
4.4.3 Other Prohibited Medication 64
4.4.4 Prohibited Food 64
4.5 Study Assessments 64
4.5.1 Informed Consent Forms and Screening Log 64
4.5.2 Procedures for Enrollment of Eligible Subjects...................... 64
4.5.3 Medical History and Demographic Data 65
4.5.4 Physical Examinations... 65
4.5.5 Performance Status 66
4.5.6 Clinical Staging.. 66
4.5.7 Criteria for Initiation of First-Line Treatment 66
4.5.8 Vital Signs.. 67
4.5.9 Tumor and Response Evaluations... 67
4.5.10 Tumor Response 68
4.5.11 Response Criteria 69
4.5.12 MRD Response Criteria... 71
4.5.13 Other Disease-Specific Assessments.................................... 72
4.5.13.1 Complete Blood Count... 72
4.5.13.2 Bone Marrow 72
4.5.13.3 Lymphadenopathy and Hepatomegaly/Splenomegaly.. 72
4.5.13.4 B-Symptoms 73
4.5.14 Laboratory, Biomarker, and Other Biological Assessments (Local and Central).. 73
4.5.15 Electrocardiograms.. 77
4.5.16 Patient-Reported and Clinician-Reported Outcomes 77

4.5.17 German Chronic Lymphocytic Leukaemia Study Group Biobanking Samples 79
4.5.17.1 Overview of the GCLLSG Biobanking 79
4.5.17.2 Approval by the Institutional Review Board or Ethics Committee 80
4.5.17.3 Sample Collection 80
4.5.17.4 Confidentiality 80
4.5.17.5 Consent to Participate in the GCLLSG Biobanking 80
4.5.17.6 Withdrawal from the GCLLSG Biobanking 81
4.6 Patient, Treatment, Study, and Site Discontinuation 81
4.6.1 Patient Discontinuation 81
4.6.2 Study Treatment Discontinuation 82
4.6.3 Study and Site Discontinuation 82

5. ASSESSMENT OF SAFETY 82
5.1 Safety Plan 82
5.1.1 Risks Associated with Venetoclax 83
5.1.1.1 Tumor Lysis Syndrome 83
5.1.1.2 Cytopenia 84
5.1.1.3 Infectious Complications 84
5.1.1.4 Effects on Cardiac Function 84
5.1.1.5 Effects on Fertility 85
5.1.1.6 Drug Interactions 85
5.1.2 Risks Associated with Obinutuzumab 85
5.1.2.1 Infusion-Related Reactions 86
5.1.2.2 Tumor Lysis Syndrome 86
5.1.2.3 Neutropenia 86
5.1.2.4 Lymphopenia 86
5.1.2.5 Thrombocytopenia 86
5.1.2.6 Infection 87
5.1.2.7 Immunization 90
5.1.2.8 Gastrointestinal Perforation 90

5.1.2.9 Risks Associated with the Combination of Venetoclax and Obinutuzumab 90
5.1.3 Management of Specific Adverse Events 91
5.1.3.1 Prophylaxis and Management of Tumor Lysis Syndrome for Patients Being Treated with Venetoclax 94
5.1.3.2 Prophylaxis for Infections 108
5.1.3.3 Prophylaxis for Infusion-Related Reactions 108
5.1.3.4 Life-Threatening Infusion-Related Reactions and Anaphylaxis 109
5.2 Safety Parameters and Definitions 109
5.2.1 Adverse Events 109
5.2.2 Serious Adverse Events (Immediately Reportable to the Sponsor) 110
5.2.3 Adverse Events of Special Interest (Immediately Reportable to the Sponsor) 110
5.3 Methods and Timing for Capturing and Assessing Safety Parameters 111
5.3.1 Adverse Event Reporting Period 111
5.3.2 Eliciting Adverse Event Information 111
5.3.3 Assessment of Severity of Adverse Events 112
5.3.4 Assessment of Causality of Adverse Events 112
5.3.5 Procedures for Recording Adverse Events 113
5.3.5.1 Infusion-Related Reactions 113
5.3.5.2 Diagnosis versus Signs and Symptoms 113
5.3.5.3 Adverse Events That Are Secondary to Other Events 113
5.3.5.4 Persistent or Recurrent Adverse Events 114
5.3.5.5 Abnormal Laboratory Values 114
5.3.5.6 Abnormal Vital Sign Values 115
5.3.5.7 Abnormal Liver Function Tests 116
5.3.5.8 Deaths 116
5.3.5.9 Preexisting Medical Conditions 116
5.3.5.10 Lack of Efficacy or Worsening of Chronic Lymphocytic Leukemia 117
5.3.5.11 Hospitalization or Prolonged Hospitalization 117

5.3.5.12 Adverse Events Associated with an Overdose or Error in Drug Administration 117
5.3.5.13 Patient-Reported Outcome Data 118
5.4 Immediate Reporting Requirements from Investigator to Sponsor.. 118
5.4.1 Emergency Medical Contacts 119
5.4.2 Reporting Requirements for Serious Adverse Events and Adverse Events of Special Interest 119
5.4.2.1 Events That Occur prior to Study Drug Initiation.................. 119
5.4.2.2 Events That Occur after Study Drug Initiation...................... 119
5.4.3 Reporting Requirements for Pregnancies............................ 120
5.4.3.1 Pregnancies in Female Patients 120
5.4.3.2 Pregnancies in Female Partners of Male Patients............... 120
5.4.3.3 Abortions 121
5.4.3.4 Congenital Anomalies/Birth Defects 121
5.5 Follow-Up of Patients after Adverse Events 121
5.5.1 Investigator Follow-Up 121
5.5.2 Sponsor Follow-Up 122
5.6 Post-study Adverse Events... 122
5.7 Expedited Reporting to Health Authorities, Investigators, Institutional Review Boards, and Ethics Committees.. 122

6. STATISTICAL CONSIDERATIONS AND ANALYSIS PLAN..................... 123
6.1 Determination of Sample Size 123
6.2 Summaries of Conduct of Study 124
6.3 Summaries of Treatment Group Comparability 124
6.4 Efficacy Analyses 124
6.4.1 Primary Efficacy Endpoint.. 124
6.4.2 Secondary Efficacy Endpoints 125
6.5 Safety Analyses... 126
6.6 Pharmacodynamic Analyses 127
6.7 Pharmacokinetic Analyses.. 127
6.8 Patient-Reported Outcome Analyses 127
6.9 Health Economics Analysis 128

6.10 Exploratory Analyses ... 128
6.11 Interim Analyses ... 129

7. DATA COLLECTION AND MANAGEMENT ... 129
7.1 Data Quality Assurance ... 129
7.2 Electronic Case Report Forms ... 130
7.3 Source Data Documentation ... 130
7.4 Use of Computerized Systems ... 131
7.5 Retention of Records ... 131

8. ETHICAL CONSIDERATIONS ... 131
8.1 Compliance with Laws and Regulations ... 131
8.2 Informed Consent ... 132
8.3 Institutional Review Board or Ethics Committee ... 133
8.4 Confidentiality ... 133
8.5 Financial Disclosure ... 134

9. STUDY DOCUMENTATION, MONITORING, AND ADMINISTRATION ... 134
9.1 Study Documentation ... 134
9.2 Protocol Deviations ... 134
9.3 Site Inspections ... 134
9.4 Administrative Structure ... 134
9.4.1 Roles and Responsibilities ... 134
9.4.2 Independent Data Monitoring Committee ... 134
9.4.3 Independent Review Committee ... 135
9.5 Publication of Data and Protection of Trade Secrets ... 135
9.6 Protocol Amendments ... 136

10. REFERENCES ... 137

LIST OF TABLES

Table 1 Treatment Arm A: Obinutuzumab + Venetoclax Study Visits ... 36
Table 2 Treatment Arm B: Obinutuzumab + Chlorambucil ... 37

Table 3 Administration of First Infusion of Obinutuzumab, if Split Over Two Days, and Subsequent Infusions of Obinutuzumab 57
Table 4 Excluded and Cautionary Medications for Patients Randomized to Arm A (Venetoclax + Obinutuzumab) 63
Table 5 Management of Hepatitis B Virus Reactivation 89
Table 6 Guidelines for Management of Specific Adverse Events (for Patients Randomized to Arms A or B) 91
Table 7 National Cancer Institute Grading Scale for Chronic Lymphocytic Leukemia 94
Table 8 Risk Categories for Developing Tumor Lysis Syndrome 95
Table 9 Summary of Tumor Lysis Syndrome Prophylaxis for Venetoclax and Monitoring Measures 96
Table 10 Adverse Event Severity Grading Scale for Events Not Specifically Listed in NCI CTCAE 112

LIST OF FIGURES

Figure 1 Study Schema 34
Figure 2 Difference in Plasma S-Warfarin Concentration Following Treatment with Warfarin + Venetoclax versus Warfarin Alone 62

LIST OF APPENDICES

Appendix 1 Schedule of Assessments Treatment-Period Visits (RAMP-UP to 400 mg) 141
Appendix 2 Schedule of Assessments Cycle 3–Cycle 12 and Follow-Up Day 28 149
Appendix 3 Schedule of Assessments (Follow-Up Period) 152
Appendix 4 Schedule of Pharmacokinetic Assessments 156
Appendix 5 Sample List of Excluded and Cautionary Medications for Patients Randomized to Arm A (Venetoclax + Obinutuzumab) 157
Appendix 6 The Modified Cumulative Illness Rating Scale (CIRS) 159
Appendix 7 Standard Cockcroft and Gault Formula for Calculated Creatinine Clearance 175
Appendix 8 Body Surface Area Calculation (BSA) Calculation Using the Mosteller Formula 176
Appendix 9 National Cancer Institute Common Terminology Criteria 177
Appendix 10 Recommendations for Initial Management of Electrolyte Imbalances and Prevention of Tumor Lysis Syndrome 178
Appendix 11 Adverse Events Commonly Associated with Chronic Lymphocytic Leukemia Study Population and/or Progression of Chronic Lymphocytic Leukemia 180

Appendix 12 International Workshop on Chronic Lymphocytic Leukemia (IWCLL) Guidelines ... 182
Appendix 13 *Schedule of Response and Minimal Residual Disease Assessments* ... 210
Appendix 14 Duration and Reporting of Adverse Events ... 211
Appendix 15 EQ-5D-3L Questionnaire ... 212
Appendix 16 Eastern Cooperative Oncology Group Performance Status ... 214
Appendix 17 Matutes Scoring System ... 215
Appendix 18 M.D. Anderson Symptom Inventory (MDASI-CLL) ... 216
Appendix 19 European Organisation for Research and Treatment of Cancer Quality of Life Questionnaire Core 30 (EORTC QLQ-C30) ... 218
Appendix 20 Guidelines for Defining Tumor Lysis Syndrome ... 220
Appendix 21 The Lawton Instrumental Activities of Daily Living Scale ... 222

PROTOCOL AMENDMENT ACCEPTANCE FORM

**TITLE:**	**A PROSPECTIVE, OPEN-LABEL, MULTICENTER RANDOMIZED PHASE III TRIAL TO COMPARE THE EFFICACY AND SAFETY OF A COMBINED REGIMEN OF OBINUTUZUMAB AND VENETOCLAX (GDC-0199/ABT-199) VERSUS OBINUTUZUMAB AND CHLORAMBUCIL IN PREVIOUSLY UNTREATED PATIENTS WITH CLL AND COEXISTING MEDICAL CONDITIONS**
**PROTOCOL NUMBER:**	BO25323/CLL14
**VERSION NUMBER:**	7
**EUDRACT NUMBER:**	2014-001810-24
**IND NUMBER:**	110159
**TEST PRODUCTS:**	Venetoclax (GDC-0199 [ABT-199]; RO5537382), Obinutuzumab (GA101; RO5072759), and Chlorambucil
**MEDICAL MONITOR:**	Maneesh Tandon, MB ChB
**SPONSOR:**	F. Hoffmann-La Roche Ltd in collaboration with the German Chronic Lymphocytic Leukaemia Study Group
**CO-SPONSOR (United States only):**	AbbVie, Inc.

I agree to conduct the study in accordance with the current protocol.

____

Principal Investigator's Name (print)

______________________________________ ______________

Principal Investigator's Signature Date

Please return the signed original of this form as instructed by your local study monitor. Please retain a copy for your study files.

PROTOCOL SYNOPSIS

**TITLE:**	**A PROSPECTIVE, OPEN-LABEL, MULTICENTER RANDOMIZED PHASE III TRIAL TO COMPARE THE EFFICACY AND SAFETY OF A COMBINED REGIMEN OF OBINUTUZUMAB AND VENETOCLAX (GDC-0199/ABT-199) VERSUS OBINUTUZUMAB AND CHLORAMBUCIL IN PREVIOUSLY UNTREATED PATIENTS WITH CLL AND COEXISTING MEDICAL CONDITIONS**
**PROTOCOL NUMBER:**	**BO25323/CLL14**
**VERSION NUMBER:**	7
**EUDRACT NUMBER:**	2014-001810-24
**IND NUMBER:**	110159
**TEST PRODUCT:**	Venetoclax (GDC-0199 [ABT-199]; RO5537382), Obinutuzumab (GA101; RO5072759), and Chlorambucil
**PHASE:**	III
**INDICATION:**	Previously untreated patients with chronic lymphocytic leukemia with coexisting medical conditions
**SPONSOR:**	F. Hoffmann-La Roche Ltd in collaboration with the German CLL Study Group (GCLLSG)
**CO-SPONSOR (United States only)**	AbbVie, Inc.

Objectives

Efficacy Objectives

The primary efficacy objective for Study BO25323/CLL14 is as follows:

- To determine efficacy by investigator-assessed progression-free survival (PFS) of a combined regimen of obinutuzumab + venetoclax compared with obinutuzumab + chlorambucil (GClb) in previously untreated patients with chronic lymphocytic leukemia (CLL) who have coexisting medical conditions

The secondary efficacy objective for this study is as follows:

- To determine efficacy as assessed by additional outcome measures (including PFS *assessed by Independent Review Committee [IRC]*, overall response, *complete response,* and MRD response rate as measured by allele-specific oligonucleotide polymerase chain reaction [ASO-PCR]*)*

Note: IRC-assessed PFS will be considered primary for U.S. regulatory purposes.

Safety Objective

The safety objective for this study is as follows:

- To evaluate the safety of the combination of obinutuzumab and venetoclax, compared with GClb, in patients with previously untreated CLL and coexisting medical conditions, focusing on the nature, frequency, and severity of Grade 3 and 4 adverse events and of serious adverse events

Pharmacokinetic Objectives

The pharmacokinetic (PK) objective for this study is as follows:

- To characterize the pharmacokinetics of venetoclax and of obinutuzumab (including population PK [popPK] techniques). Standard non-compartmental analysis (NCA)/descriptive tables, listings, and graphs (TLGs) and popPK approaches will be considered.

Patient-Reported Outcome Objectives

The quality-of-life (patient-reported outcome [PRO]) objectives for this study are as follows:

- To compare disease and treatment-related symptoms following treatment with the combination of obinutuzumab + venetoclax compared with GClb in patients with previously untreated CLL and coexisting medical conditions as measured by M.D. Anderson Symptom Inventory (MDASI-CLL)
- To evaluate changes in role functioning and global health status/quality of life (QoL) following treatment with the combination of obinutuzumab + venetoclax compared with GClb in patients with previously untreated CLL and coexisting medical conditions between arms as measured by European Organisation for Research and Treatment of Cancer Quality of Life Questionnaire Core 30 (EORTC QLQ-C30)

Health Economic Objective

The health economic objective for this study should be considered as a special PRO and is as follows:

- To compare the health utility effects of treatment with combination of obinutuzumab and venetoclax compared with GClb in patients with previously untreated CLL and coexisting medical conditions measured by the EuroQol 5 Dimension questionnaire (EQ-5D-3L).

Exploratory Objectives

The exploratory objectives for this study are as follows:

- To assess MRD using new technologies, including flow cytometry and next-generation sequencing, to compare results with MRD measured by ASO-PCR
- To evaluate the relationship between PFS and MRD response rate
- To evaluate the relationship between various baseline prognostic markers and clinical outcome parameters
- To evaluate the relationship between biomarkers measured at baseline and disease progression to understand mechanisms of response and resistance

Study Design

Description of Study

This is an open-label, multicenter, randomized Phase III trial to compare the efficacy and safety of a combined regimen of obinutuzumab and venetoclax versus GClb in patients with CLL and coexisting medical conditions.

Initially, there will be a 12-patient safety run-in phase, (including at least 1 patient at high risk of developing TLS) wherein patients will receive obinutuzumab + venetoclax in a non-randomized fashion. After the twelfth patient has reached the end of Cycle 3, a formal review will be undertaken by Roche and the German CLL Study Group ([GCLLSG]; together, hereafter referred to as the Sponsor) and an independent Data Monitoring Committee (iDMC).

The following are stopping criteria for the run-in phase of this study:

- One treatment-related death

  or

- One Grade 4 adverse event related to a clinical tumor lysis syndrome (TLS) despite protocol-specified prophylaxis, either following the administration of the first dose of venetoclax or during dose escalation

If any of these criteria are met, the main study will not be opened for recruitment, and the Sponsor will then re-evaluate the study design and amend the protocol accordingly. If the stopping criteria are not met, randomization into the trial will commence.

Number of Patients

A total of 420 patients will be enrolled in the randomized part of the study, and 12 patients will be enrolled in the safety run-in phase.

Target Population

Inclusion Criteria

Patients must meet the following criteria for study entry:

- Have documented previously untreated CLL according to International Workshop on Chronic Lymphocytic Leukemia (IWCLL) criteria
- CLL that requires treatment according to the IWCLL criteria
- Total Cumulative Illness Rating Scale (CIRS) score >6 or creatinine clearance (CrCl) <70 mL/min
- Adequate marrow function independent of growth factor or transfusion support within 2 weeks of screening as follows, unless cytopenia is due to marrow involvement of CLL:

  Absolute neutrophil count $\geq 1.0 \times 10^9$/L

  Platelet counts $\geq 30 \times 10^9$/L; in cases of thrombocytopenia clearly due to marrow involvement of CLL (per the discretion of the investigator); platelet count should be $\geq 10 \times 10^9$/L if there is bone marrow involvement

  Total hemoglobin $\geq$ 9 g/dL (without transfusion support, unless anemia is due to marrow involvement of CLL)

- Adequate liver function as indicated by a total bilirubin, AST, and ALT ≤ 2 times the institutional upper limit of normal (ULN) value unless directly attributable to the patient's CLL
- For those patients with a screening lymphocyte count $<5{,}000$ cells/μL, historical data that confirms a lymphocyte count $\geq 5{,}000$ cells/μL at the time of diagnosis is required
- 18 years of age or older
- Life expectancy >6 months
- Signed informed consent and, in the investigator's judgment, able to comply with the study protocol
- For women who are not postmenopausal (≥ 12 months of non-therapy-induced amenorrhea) or surgically sterile (absence of ovaries and/or uterus): agreement to remain abstinent or use single or combined contraceptive methods that result in a failure rate of $<1\%$ per year during the treatment period and for at least 30 days after the last dose of venetoclax or 18 months after the last dose of obinutuzumab, whichever is longer

  Abstinence is only acceptable if it is in line with the preferred and usual lifestyle of the patient. Periodic abstinence (e.g., calendar, ovulation, symptothermal, or post-ovulation methods) and withdrawal are not acceptable methods of contraception.

  Examples of contraceptive methods with a failure rate of $<$ 1% per year include tubal ligation, male sterilization, hormonal implants, established, proper use of combined oral or injected hormonal contraceptives, and certain intrauterine devices. Alternatively, two methods (e.g., two barrier methods such as a condom and a cervical cap) may be

combined to achieve a failure rate of < 1% per year. Barrier methods must always be supplemented with the use of a spermicide.

- For men: agreement to remain abstinent or use a condom plus an additional contraceptive method that together result in a failure rate of < 1% per year during the treatment period and for at least 90 days after the last dose of venetoclax or 18 months after the last dose of obinutuzumab, whichever is longer, and agreement to refrain from donating sperm during the treatment period and for at least 90 days after the last dose of venetoclax

  Men with a pregnant partner must agree to remain abstinent or use a condom for the duration of the pregnancy.

- Abstinence is only acceptable if it is in line with the preferred and usual lifestyle of the patient. Periodic abstinence (e.g., calendar, ovulation, symptothermal, or post-ovulation methods) and withdrawal are not acceptable methods of contraception.

Exclusion Criteria

Patients who meet any of the following criteria will be excluded from study entry:

- Transformation of CLL to aggressive non-Hodgkin's lymphoma (NHL) (Richter's transformation or pro-lymphocytic leukemia)
- Known central nervous system involvement
- Patients with a history of confirmed progressive multifocal leukoencephalopathy
- An individual organ/system impairment score of 4 as assessed by the CIRS definition limiting the ability to receive the treatment regimen of this trial with the exception of eyes, ears, nose, throat organ system (note that symptoms related to CLL should not be included in the patient's screening CIRS score). Investigators should consult the General Rules for Severity Rating as well as the Organ-Specific Categories when assigning scores for certain conditions (i.e., pulmonary embolism) and consider the level of morbidity associated with a patient's condition.
- Patients with uncontrolled autoimmune hemolytic anemia or immune thrombocytopenia
- Inadequate renal function: CrCl < 30 mL/min
- History of prior malignancy, except for conditions as listed below if patients have recovered from the acute side effects incurred as a result of previous therapy:

  Malignancies surgically treated with curative intent and with no known active disease present for ≥ 3 years before randomization

  Adequately treated non-melanoma skin cancer or lentigo maligna without evidence of disease

  Adequately treated cervical carcinoma in situ without evidence of disease

  Surgically/adequately treated low grade, early stage, localized prostate cancer without evidence of disease

- Patients with active infections requiring IV treatment (Grade 3 or 4) within the last 2 months prior to enrollment
- History of severe allergic or anaphylactic reactions to humanized or murine monoclonal antibodies or known sensitivity or allergy to murine products
- Hypersensitivity to chlorambucil, obinutuzumab, or venetoclax or to any of the excipients (e.g., trehalose)
- Pregnant women and nursing mothers
- Vaccination with a live vaccine ≤ 28 days prior to randomization
- Prisoners or patients who are institutionalized by regulatory or court order or persons who are in dependence to the Sponsor or an investigator
- History of illicit drug or alcohol abuse within 12 months prior to screening, in the investigator's judgment

- Positive test results for chronic hepatitis B virus (HBV) infection (defined as positive hepatitis B surface antigen [HBsAg] serology)

  Patients with occult or prior HBV infection (defined as negative HBsAg and positive total hepatitis B core antibody [HBcAb]) may be included if HBV DNA is undetectable, provided that they are willing to undergo monthly DNA testing. Patients who have protective titers of hepatitis B surface antibody (HBsAb) after vaccination or prior but cured hepatitis B are eligible.

- Positive test result for hepatitis C (hepatitis C virus [HCV] antibody serology testing)

  Patients who are positive for HCV antibody are eligible only if PCR is negative for HCV RNA.

- Patients with known infection with human immunodeficiency virus (HIV) or Human T-Cell Leukemia Virus 1 (HTLV-1)

  In countries where mandatory testing by health authorities is required, HIV testing will be performed.

  HTLV testing is required in patients from endemic countries (Japan, countries in the Caribbean basin, South America, Central America, sub-Saharan Africa, and Melanesia).

- Any serious medical condition or abnormality in clinical laboratory tests that, in the investigator's judgment, precludes the patient's safe participation in and completion of the study
- Patients who have received:

  Strong and moderate CYP3A inhibitors within 7 days prior to the first dose of study drug administration

  Strong and moderate CYP3A inducers within 7 days prior to the first dose of study drug administration

  Consumed grapefruit, grapefruit products, Seville oranges (including marmalade containing Seville oranges), or star fruit within 3 days prior to the first dose of study drug and throughout venetoclax administration

- Inability to swallow a large number of tablets

Length of Study

The approximate length of study will be 6 years and 8 months calculated from an estimated 20-month recruitment period, and the end of study is defined below.

End of Study

The end of this study is defined as 5 years from last patient enrolled (unless all patients have died).

<u>Outcome Measures</u>

Efficacy Outcome Measures

The primary efficacy outcome measure for this study is as follows:

- PFS, defined as the time from randomization to the first occurrence of progression, relapse, or death from any cause as assessed by the investigator. Disease progression will be assessed by the investigators using the IWCLL criteria (2008).

The secondary efficacy outcome measures for this study are as follows:

- PFS based on Independent Review Committee (IRC)-assessments (primary outcome for U.S. regulatory purposes), defined as the time from randomization to the first occurrence of progression or relapse or death from any cause
- Overall response rate (ORR; defined as rate of a clinical response of complete response [CR], complete response with incomplete bone marrow recovery [CRi], or PR) at the completion of treatment assessment, as determined by the investigator according to the IWCLL guidelines (2008)

- *Complete response rate (CRR; defined as rate of a clinical response of CR or CRi) at the completion of treatment assessment, as determined by the investigator according to the 2008 IWCLL guidelines*
- MRD response rate (determined as the proportion of patients with MRD-negativity) measured in the peripheral blood at the completion of treatment assessment and MRD response rate as measured in the bone marrow at the completion of treatment, both measured by ASO-PCR
- ORR at completion of combination treatment response assessment (Cycle 7, Day 1 or 28 days after last intravenous [IV] infusion)
- MRD response rates in the peripheral blood at completion of combination treatment assessment (Cycle 9, Day 1 or 3 months after last IV infusion) and also MRD response rate in the bone marrow, both as measured by ASO-PCR

Note: the secondary endpoint analyses will be on the basis of MRD assessment performed centrally by ASO-PCR with MRD negativity defined using a cutoff of 10^{-4} (less than 1 cell in 10,000 leukocytes). As well as evaluating MRD response rate, it will further be evaluated using results of both measures, where a patient will be considered MRD-positive if either blood or bone marrow result is positive or both are missing. MRD response rates at other timepoints during treatment and follow-up where MRD is measured in the peripheral blood will also be summarized.

- Overall survival (OS), defined as the time between the date of randomization and the date of death due to any cause
- Duration of objective response, defined as the time from the first occurrence of a documented objective response to the time of progressive disease (PD) as determined by the investigator or death from any cause
- Best response achieved (CR, CRi, partial response [PR], stable disease, or PD) up to and including the assessment at completion of treatment assessment (within 3 months of last day of treatment)
- Event-free survival, defined as the time between date of randomization and the date of disease progression/relapse on the basis of investigator-assessment, death, or start of a new anti-leukemic therapy
- Time to next anti-leukemic treatment, defined as time between the date of randomization and the date of first intake of new anti-leukemic therapy

Safety Outcome Measures

The safety outcome measures for this study are as follows:

- Nature, frequency, and severity of adverse events and serious adverse events
- Changes in vital signs, physical findings, and clinical laboratory results during and following study treatment
- Lymphocyte immunophenotyping and incidence of human–anti-human antibodies
- Premature withdrawals

Pharmacodynamic Outcome Measures

For each visit at which CD19 + CD5 + and CD19 + CD5 − B-cell measurements are taken, B-cell data will be listed for individual patients by treatment arm.

Pharmacokinetic Outcome Measures

The PK outcome measure for this study is as follows:

- Apparent clearance, apparent volume of distribution, and/or other appropriate PK parameters of venetoclax and of obinutuzumab characterized with the use of popPK techniques. Standard NCA/descriptive TLGs and popPK approaches will be considered.

Patient-Reported Outcome Measures

The PRO measures for this study are as described below. The first assessment will be done during the first obinutuzumab infusion, and PROs will be followed until end of study as defined by 5 years after last randomized patient:

- To evaluate changes following treatment in disease and treatment-related symptoms in MDASI-CLL scores.
- To evaluate changes in role functioning and global health status/QoL scales following treatment with the EORTC QLQ-C30.

Health Economic Outcome Measures

The health economic outcome measure for this study is as follows:

- The EQ-5D-3L questionnaire

Exploratory Outcome Measures

The exploratory outcome measures for this study are as follows (provided these data are available in a sufficiently timely and complete form to perform these analyses at the time of the final primary analysis):

- MRD negativity in peripheral blood measured using new technologies, including flow cytometry and next-generation sequencing with MRD negativity defined using a cutoff of 10^{-4} (less than 1 cell in 10,000 leukocytes) for comparison with ASO-PCR, and secondly by the limit of sensitivity of each of the above technologies.
- Relationship between MRD and PFS on the basis of peripheral blood assessed using ASO-PCR
- Relationship between various baseline markers and clinical outcome parameters in patients from both arms of the study (including but not limited to CLL fluorescence in-situ hybridization [FISH; 17p-, 11q-, 13p-, + 12q], immunoglobulin heavy chain variable [IGHV] mutation status, p53 mutation status, serum parameters, Bcl-2 expression and other CLL disease markers)

__Investigational Medicinal Products__

All investigational medicinal products (IMPs) required for completion of this study (chlorambucil, obinutuzumab, and venetoclax) will be provided by the Sponsor where required by local health authority regulations. The study site will acknowledge receipt of IMPs and confirm the shipment condition and content. Any damaged shipments will be replaced.

IMPs will either be disposed of at the study site according to the study site's institutional standard operating procedure or returned to the Sponsor with the appropriate documentation. The site's method of IMP destruction must be agreed to by the Sponsor. The site must obtain written authorization from the Sponsor before any IMP is destroyed, and IMP destruction must be documented on the appropriate form.

Accurate records of all IMPs received at, dispensed from, returned to, and disposed of by the study site should be recorded on the Drug Inventory Log.

__Venetoclax__

Venetoclax, a highly selective, orally bioavailable, small-molecule Bcl-2 family inhibitor in the biarylacylsulfonamide chemical class, will be administered as an oral tablet. Patients will receive venetoclax as follows:

- 20 mg daily during Cycle 1, Days 22–28
- 50 mg daily during Cycle 2, Days 1–7
- 100 mg daily during Cycle 2, Days 8–14
- 200 mg daily during Cycle 2, Days 15–21
- 400 mg daily during Cycle 2, Days 22–28 and on Days 1–28 for all subsequent cycles until the end of Cycle 12

Obinutuzumab

Obinutuzumab, a novel, humanized, type II glycoengineered monoclonal antibody (mAb) directed against the CD20 antigen, will be administered as an IV infusion. Patients will receive obinutuzumab as follows:

- 100 mg or 1000 mg, depending on splitting rules, at Cycle 1, Day 1 (if 100 mg was received on Day 1, 900 mg will be administered on Cycle 1, Day 2).
- 1000 mg at Cycle 1, Day 8 and Day 15
- 1000 mg at Day 1 for all subsequent cycles until the end of Cycle 6

Chrorambucil

Chlorambucil will be administered orally. Patients will receive chlorambucil as follows:

- 0.5 mg/kg at Day 1 and Day 15 for Cycles 1–12

Non-Investigational Medicinal Products

Rasburicase

Rasburicase (Fasturtec™ in Europe; Elitek™ in the United States) enzymatically converts uric acid in the blood to allantoin and hydrogen peroxide, thereby preventing the formation of uric acid crystals and potential renal blockage. It reduces uric acid levels within 4 hours both in pediatric and adult patients, and several studies confirm its safety, effectiveness, and tolerability both in the prevention and treatment of TLS, although the prevention of acute renal failure fails in over 25% of patients.

Note: Rasburicase may be supplied by the Sponsor in countries where rasburicase is not approved or cannot be supplied locally.

Statistical Methods

Primary Analysis

The primary efficacy endpoint is investigator-assessed PFS, defined as the time from randomization to the first occurrence of progression or relapse (determined using standard IWCLL guidelines [2008]), or death from any cause, whichever occurs first. Progression-free survival on the basis of IRC assessments will be considered primary for U.S. regulatory purposes (details will be provided in the Statistical Analysis Plan [SAP]). For patients who have not progressed, relapsed, or died at the time of analysis, PFS will be censored on the date of the last disease assessment. If no disease assessments were performed after the baseline visit, PFS will be censored at the time of randomization + 1 day. All patients, including patients who discontinue all components of study therapy prior to disease progression (e.g., for toxicity), will continue in the study and will be followed for progressive disease and survival regardless of whether or not they subsequently receive new anti-leukemic therapy.

The primary objective of the study is to test the following hypothesis:

- Progression-free survival of obinutuzumab + venetoclax versus GClb (i.e., H0: obinutuzumab + venetoclax = GClb versus H1: obinutuzumab + venetoclax ≠ GClb)

Treatment comparisons will be made using a two-sided log-rank test (at 0.05 significance-level, adjusted for the interim *analyses*), stratified by Binet stage. If the null hypothesis is rejected and the observed HR is favorable for the obinutuzumab + venetoclax experimental arm, then it is concluded that obinutuzumab + venetoclax significantly lowers the risk of PFS events more than GClb. A two-sided non-stratified log-rank test will be performed to support the primary analysis. Median PFS and the 95% confidence limits will be estimated using Kaplan-Meier survival methodology, with the Kaplan-Meier survival curve presented to provide a visual description. PFS rates for 1, 2, and 3 years after randomization with 95% CIs will be reported. Estimates of the treatment effect will be expressed as HR including 95% confidence limits estimated through a Cox proportional-hazards analysis stratified by Binet stage. Primary analysis for U.S. Food and Drug Administration (FDA) submission will be based on assessment of PFS by an IRC.

Determination of Sample Size

The sample size for the study is determined given the requirements to perform a hypothesis test for clinically relevant statistical superiority in the primary endpoint of PFS.

Estimates of the number of events required to demonstrate efficacy with regard to PFS are based on the following assumptions:

- Log-rank test at the two-sided 0.05 level of significance
- Median PFS for obinutuzumab and chlorambucil control arm (27 months)
- 80% power to detect HR = 0.65 for the comparison of obinutuzumab + venetoclax experimental arm versus GClb, with median PFS for obinutuzumab + venetoclax increased to 41.5 months
- Exponential distribution of PFS
- Annual drop-out rate of 10%
- One interim analysis for efficacy after 75% of PFS events, utilizing a stopping boundary according to the γ family error spending function with parameter $\gamma = -9.21$.

The addition of an optional early analysis requires no adjustment to the sample size, as the impact on the statistical power calculation is negligible.

Based on these assumptions, a total of 170 PFS events are required for the final analysis of PFS.

The minimum detectable difference at the final analysis corresponds approximately to an HR = 0.74.

The sample size calculation was performed using EAST version 6.2.

Interim *Analyses*

In addition to the periodic safety data reviews, the iDMC will evaluate efficacy and safety at *up to two* formal interim *analyses* of PFS and recommend if the study should be stopped early for efficacy.

Summaries and analyses will be prepared by an independent data coordinating center and presented by treatment arm for the iDMC's review.

An interim efficacy analysis may be conducted at or after 1 year after the last patient's last venetoclax dose (i.e., Month 37 of the study [August 2018]), provided that at least 85 PFS events (50% of the total of 170 PFS events) have occurred. If 85 PFS events have not been observed by 1 year after the last patient's last venetoclax dose, then the interim analysis will be conducted once a minimum of 85 PFS events have occurred. PFS will be tested at the significance level determined using the gamma family error spending function with parameter $\gamma = -21.12$ so that the overall two-sided type I error rate will be maintained at the 0.05 level. This gamma family boundary only allows the study to stop for efficacy if a treatment effect HR of 0.35 or better is observed when the interim analysis is based on 85 events.

Because this option is added to mitigate a potential delay in the study read-out, the Sponsor's decision to conduct this interim analysis will be based on a number of factors including, but not limited to, the number of events observed by 1 year after the last patient's last venetoclax dose and the subsequent predicted time to reach 110 events. If the Sponsor does not conduct this interim analysis or if this interim analysis is conducted and is negative, then the Sponsor will proceed with the later original interim analysis as follows.

Provided the above early interim analysis is not done or not passed, the original interim analysis for efficacy will be performed once *a minimum of* 110 PFS events have occurred. PFS will be tested at the significance level determined using the gamma family error spending function with parameter $\gamma = -9.21$ so that the overall two-sided type I error rate will be maintained at the 0.05 level. *If the early interim analysis (at a minimum of 85 events) is performed and is passed, the later original interim analysis will not be undertaken.*

The final analysis will be performed after 170 events have occurred. The significance level will be adjusted to incorporate the α spent at *either* interim *analysis*, so that the overall two-sided type I error rate will be maintained at the 0.05 level.

Abbreviation	Definition
ALL	acute lymphoblastic lymphoma
AML	acute myeloid leukemia
ALC	absolute lymphocyte count
ANC	absolute neutrophil count
ASO-PCR	allele-specific oligonucleotide polymerase chain reaction
CIRS	Cumulative Illness Rating Scale
CLL	chronic lymphocytic leukemia
CR	complete response
CrCl	creatinine clearance
CRi	complete response with incomplete bone marrow recovery
CT	computed tomography
CTCAE	Common Terminology Criteria for Adverse Events
DLBCL	diffuse large B-cell lymphoma
EC	Ethics Committee
ECOG	Eastern Cooperative Oncology Group
eCRF	electronic Case Report Form
EDC	electronic data capture
EDTA	ethylenediaminetetraacetic acid
EFS	event-free survival
EORTC QLQ-C30	European Organisation for Research and Treatment of Cancer Quality of Life Questionnaire Core 30
EQ-5D-3L	EuroQol 5-Dimension questionnaire
FC	fludarabine+cyclophosphamide
FCR	fludarabine+cyclophosphamide+rituximab
FDA	U.S. Food and Drug Administration
FFPE	formalin-fixed paraffin embedded
FISH	fluorescence in-situ hybridization
FL	follicular lymphoma
GClb	obinutuzumab+chlorambucil
GCLLSG	German Chronic Lymphocytic Leukaemia Study Group
HBcAb	hepatitis B core antibody
HBsAb	hepatitis B surface antibody
HBsAg	hepatitis B surface antigen
HBV	hepatitis B virus
HCV	hepatitis C virus
HR	hazard ratio
HSC	hematopoietic stem cell

Abbreviation	Definition
IADL	instrumental activities of daily living
ICF	informed consent form
ICH	International Conference on Harmonisation
iDMC	independent Data Monitoring Committee
IGHV	immunoglobulin heavy chain variable
IHC	immunohistochemsitry
INR	international normalized ratio
IMP	investigational medicinal product
IRB	Institutional Review Board
IRC	Independent Review Committee
IRR	infusion-related reaction
IWCLL	International Workshop on Chronic Lymphocytic Leukemia
IV	intravenous
IxRS	interactive voice-/web-based system
JCV	John Cunningham Virus
LDT	lymphocyte doubling time
LVEF	left ventricular ejection fraction
mAb	monoclonal antibody
MDASI-CLL	M.D. Anderson Symptom Assessment Inventory—Chronic Lymphocytic Leukemia
MCL	mantle cell lymphoma
MM	multiple myeloma
MRD	minimal residual disease
MRI	magnetic resonance imaging
NCA	non-compartmental analysis
NCI	National Cancer Institute
NGS	next-generation sequencing
NHL	non-Hodgkin's lymphoma
NLT	new leukemia treatment
ORR	overall response rate
OS	overall survival
PD	progressive disease
PFS	progression-free survival
PK	pharmacokinetic
PML	progressive multifocal leukoencephalopathy
popPK	population pharmacokinetic
PR	partial response

Abbreviation	Definition
PRO	patient-reported outcome
PS	performance status
PVC	polyvinyl chloride
QoL	quality of life
QTcF	Corrected (Fridericia's correction) QTc interval
RClb	rituximab + chlorambucil
RCR	Roche Clinical Repository
SAP	Statistical Analysis Plan
SLL	small lymphocytic lymphoma
SmPC	summary of product characteristics
SNP	single nucleotide polymorphism
TLG	table, listing, and graph
TLS	tumor lysis syndrome
ULN	upper limit of normal

1. BACKGROUND

1.1 BACKGROUND ON CHRONIC LYMPHOCYTIC LEUKEMIA

With an age-adjusted incidence of 4.1/100,000 inhabitants in the United States, chronic lymphocytic leukemia (CLL) is the most common type of leukemia in western countries. More than 15,000 newly diagnosed cases and 4,500 deaths are currently estimated annually (Jemal et al. 2007; National Cancer Institute 2010). The median age at diagnosis is between 67 and 72 years (Molica 2006). More male than female patients (1.7:1) are affected (Molica 2006; Morton et al. 2006; Watson et al. 2008). Because the incidence rate increases with age, the prevalence and mortality of CLL are likely to increase over the next decades because of the demographic changes associated with an aging population. Moreover, the proportion of younger patients with early-stage CLL and minimal symptoms seems to increase because of more frequent blood testing (Mauro et al. 1999). CLL is characterized by clonal proliferation and accumulation of mature, typically CD5-positive, B cells within the blood, bone marrow, lymph nodes, and spleen (Rozman et al. 1995). It has recently been reported that in CLL, the capacity to generate clonal B cells might be acquired at the hematopoietic stem cell (HSC) stage (Kikushige et al. 2011), suggesting that the primary leukemogenic event in CLL might involve multipotent, self-renewing HSCs. The leukemic transformation is initiated by specific genetic alterations that cause the deletion of specific micro-RNA genes and increase the resistance of B cells toward apoptosis (Calin et al. 2002). Deletions on the long arm of chromosome 13, specifically involving band 13q14 (del(13q14)), represent the single most frequently observed cytogenetic aberration in CLL, occurring in approximately 55% of all cases. An isolated del(13q14) is typically characterized by a benign course of the disease. Deletions of the long arm of chromosome 11 (del(11q)) can be found in approximately 25% of chemotherapy-naive patients with advanced disease stages and in 10% of patients with early-stage disease (Zenz et al. 2010a; Quesada et al. 2011). Some of the poor prognostic features of del(11q) seem to be overcome by the use of chemoimmunotherapy (Hallek et al. 2010).

Deletions of the short arm of chromosome 17 (del(17p)) are found in 5%–8% of chemotherapy-naive patients. These deletions almost always include band 17p13, where the prominent tumor suppressor gene *TP53* is located. Patients with CLL who carry a del(17p) clone show marked resistance against genotoxic chemotherapies that cannot be overcome by the addition of anti-CD20 antibodies in the context of state-of-the art chemoimmunotherapy (Hallek et al. 2010). Mutations of *TP53* are found in 4%–37% of patients with CLL and have been associated with poor prognosis (ultra-high risk) in a number of studies (Zenz et al. 2010b).

The recently reported whole genome sequencing projects in CLL have revealed a number of recurrent somatic gene mutations that occur in parallel to the above mentioned structural genomic aberrations. These include the genes *NOTCH1, MYD88, TP53, ATM, SF3B1, FBXW7, POT1, CHD2*, and others (Puente et al. 2011; Quesada et al. 2011).

More than 50% of CLL patients are asymptomatic at diagnosis and require no treatment. Symptoms appear as the disease progresses. Treatment is initiated when a patient's disease becomes symptomatic or progressive as defined by the international workshop on Chronic Lymphocytic Leukemia (iwCLL) updated guidelines for diagnosis and treatment of CLL (Hallek et al. 2008; see Appendix 12).

During the past 10 years, the combination of fludarabine + cyclophosphamide + rituximab (FCR) has become the standard front-line treatment for most of the physically fit patients with CLL. The German Chronic Lymphocytic Leukaemia Study Group (GCLLSG) performed a randomized trial, Study CLL8/ML17102, that compared FCR with fludarabine + cyclophosphamide (FC) chemotherapy. FCR produced an overall response rate (ORR) of 95%; the complete response (CR) rate seen with FCR was double that seen with FC alone (44% vs. 22%). A recent update showed that the median progression-free survival (PFS) with FCR is 5 years, and this was the first trial to show a survival advantage for a front-line regimen in treatment of CLL (Hallek et al. 2010).

However, for a large subgroup of elderly patients with coexisting conditions and elderly patients with CLL, FCR is not a suitable treatment. Therefore, a large, randomized Phase III trial CLL11/BO21004 was conducted which investigated first-line chemoimmunotherapy in patients with CLL with coexisting medical conditions (i.e., patients typically treated in daily practice) (Chiorazzi et al. 2005). In the final Stage 2 analysis with efficacy and safety, results of the head-to-head comparison between obinutuzumab + chlorambucil (GClb) and rituximab + chlorambucil (RClb) were evaluated.

In the GClb versus RClb arm, the ORR was 78% versus 65%, and CR was 21% versus 7%, respectively. The number of patients with minimal residual disease (MRD)-negative blood samples at end of treatment was more than 10-fold higher with GClb compared with RClb (63 of 214 patients [29.4%] versus 6 of 243 patients [2.5%]). Grade 3–4 infusion-related reaction (IRR) with GClb occurred at first infusion only. Treatment with GClb, as compared with RClb, resulted in prolongation of PFS (hazard ratio [HR] $=$ 0.39; 95% confidence interval [CI]: 0.31, 0.49; $p < 0.001$). Median PFS was 26.7 months with GClb and 16.3 months with RClb (Goede et al. 2014).

It can be concluded that GClb demonstrates statistically significant and clinically meaningful prolongation of PFS and a higher CR rate and MRD response rate compared with RClb in previously untreated patients with CLL and coexisting medical conditions. IRRs, thrombocytopenia, and neutropenia were more common with GClb without an increase in infections or deaths due to adverse events. Furthermore, GClb versus chlorambucil alone demonstrated a significant prolongation of overall survival (OS). Overall, GClb is superior to RClb and a highly active treatment in this typical CLL–patient population.

1.2 BACKGROUND ON VENETOCLAX (GDC-0199)

Venetoclax (also known as GDC-0199 or ABT-199) is a highly selective, orally bioavailable, small-molecule Bcl-2 family inhibitor in the biarylacylsulfonamide chemical class. Venetoclax binds with high affinity ($Ki < 0.010$ nM) to anti-apoptotic protein Bcl-2 and with lower affinity to other anti-apoptotic Bcl-2 family proteins, including Bcl-XL and Bcl-w (>4000-fold and >2000-fold to >20,000-fold lower affinity than to Bcl-2, respectively) (Souers et al. 2013).

Anti-apoptotic Bcl-2 family members are associated with tumor initiation, disease progression, and chemotherapy resistance (Fesik 2005), as well as autoimmunity (Mérino et al. 2009). Overexpression of Bcl-2 is a major contributor to the pathogenesis of some lymphoid malignancies; antagonism of the action of these proteins may enhance response to therapy and overcome resistance, and, thus, these proteins are compelling targets for anti-tumor therapy. In vitro, venetoclax demonstrated cell killing activity against patient-derived CLL cells and acute myeloid leukemia (AML) cells and a variety of lymphoma and leukemia cell lines, including B-cell follicular lymphoma (FL), mantle cell lymphoma (MCL), diffuse large B-cell lymphoma (DLBCL), and multiple myeloma (MM) (Venetoclax Investigator’s Brochure, 2014). Venetoclax was especially potent against cell lines expressing high levels of Bcl-2. Venetoclax inhibits subcutaneous xenograft growth of human tumor cell lines derived from acute lymphoblastic leukemia (ALL), non-Hodgkin’s lymphoma (NHL), and AML and is highly efficacious when using various doses and combined with other regimens. The drug is also active in a model of disseminated ALL. Venetoclax enhanced the activity of a broad variety of chemotherapeutic agents in other human hematological models (Venetoclax Investigators Brochure, 2014). Specifically, venetoclax enhances the efficacy of obinutuzumab and rituximab in models of MCL and DLBCL (Venetoclax /Obinutuzumab Investigators Brochure, 2014). Furthermore, venetoclax demonstrated potential to enhance the efficacy of bortezomib in a transgenic murine lymphoma model (Vandenberg and Cory 2013) and shows single agent efficacy in a human model of multiple myeloma (Touzeau et al. 2014).

See the Investigator’s Brochure for additional details on nonclinical and clinical studies.

1.3 STUDY RATIONALE AND BENEFIT-RISK ASSESSMENT

1.3.1 <u>Efficacy</u>

Despite the progress made in the treatment of patients with CLL, a significant number of patients experience relapsed disease that is associated with progressively shorter durations of response to therapy. The only potentially curative strategy for CLL is an allogeneic hematopoietic stem cell transplantation for which the majority of patients with CLL are not eligible because of age or coexisting medical conditions. As a result, a cure for patients with CLL necessitates more effective drugs that also have a safety profile suited for patients who are unfit and patients who are elderly. A chemotherapy-free regimen could meet these expectations.

Preliminary data from ongoing clinical studies with venetoclax demonstrate its high activity in patients with CLL. Preliminary efficacy results were available for patients with CLL/small lymphocytic lymphoma (SLL) in Studies M12-175 (Arm A) and M13-365 as of 09 April 2014 and 17 January 2014, respectively.

Chronic Lymphocytic Leukemia/Small Lymphocytic Lymphoma

In Arm A of the Phase I Study M12-175 (relapsed/refractory CLL/SLL treated with venetoclax monotherapy), the ORR, consisting of CR/CR with incomplete bone marrow recovery (CRi) plus partial response (PR), was 77% (60 of 78 evaluable patients) with CR/CRi in 18 patients (23%) and PR in 42 patients (54%). The ORR was 75% in the 24 patients with unmutated immunoglobulin heavy chain variable region (IGHV) status with 29% achieving CR/Cri (Venetoclax Investigator's Brochure, 2014).

The ORR was 79% in the 19 patients harboring the deletion of the p13 locus on chromosome 17 with 26% achieving CR/CRi. The ORR was 76% in the 41 patients who were fludarabine-refractory with 9 of 41 patients (22%) achieving CR/CRi. In Study M13-365 (relapsed CLL treated with venetoclax + rituximab), the ORR was 78% (14 of 18 evaluable patients) with 7 of 18 patients (39%) achieving CR/CRi and 7 of 18 patients (39%) achieving PR.

1.3.2 Safety

Known and potential risks associated with venetoclax administration include tumor lysis syndrome (TLS), hematological effects (including lymphopenia, neutropenia/febrile neutropenia, thrombocytopenia and anemia/hemoglobin decreased), serious and/or opportunistic infections, nausea, and diarrhea.

Clinical evaluations of the risks identified from previous preclinical studies (single-cell necrosis, allergic reactions, liver enzyme elevations, and hair color change) did not reveal any clinically significant concerns, and, thus, routine surveillance is recommended in ongoing studies. Clinical evaluation of adverse events that could potentially be associated with decreased left ventricular ejection fraction (LVEF) and evaluation of available pre- and post-dose LVEF data showed no risk of decreased LVEF following venetoclax dosing. Because study populations in venetoclax oncology studies are likely to be elderly and have received multiple prior chemotherapeutic agents, baseline LVEF assessments are recommended per the investigator's discretion.

Principal risks identified for the combination regimen of obinutuzumab with venetoclax include: TLS, IRRs to obinutuzumab, cytopenia (i.e., neutropenia and thrombocytopenia especially during the first cycle of obinutuzumab, or lymphopenia), infections, transient transaminitis, and other malignancies.

However, toxicities expected to be associated with the combined treatment of obinutuzumab and venetoclax can largely be managed (e.g., first dose TLS and IRRs),

or are commonly encountered with the treatment of CLL and can be mitigated with the measures outlined in this protocol.

The protocol includes the following features to address these risks:

- TLS appears to be an adverse event that can be mitigated by starting therapy with venetoclax at the low dose of 20 mg, by slow dose ramp-up, and by appropriate preventive measures. Measures to prevent TLS include prophylactic hydration and medication prior to initial dosing with careful monitoring during the first 24 hours after dosing for metabolic or clinical signs of impending TLS (Section 5.1.1.1).
- Staggered dosing of venetoclax initiated on Day 22 during the first cycle of chemoimmunotherapy
- Prophylactic medication (Section 5.1.1.3) will be administered, and careful monitoring guidelines will be used to manage IRRs to obinutuzumab.
- Frequent blood testing throughout the treatment period to monitor for cytopenia
- Dose delays or dose reductions are mandated for severe cytopenia. In addition, all supportive measures, including transfusion, antibiotics, and the use of growth factors are allowed.
- Neutropenia will be closely monitored and may be treated with growth factors, and any signs of infection will be treated with appropriate medication.

    Note: Anti-infection prophylaxis is not mandated for all patients because of the potential risks of adding additional concomitant medications but may be prescribed by the investigator in specific cases where patients are at especially high risk. Patients with pre-existing infections and past hepatitis are excluded from the study to avoid reactivation.

- Incidences of prolonged neutropenia are of concern and will be monitored carefully.
- Guidelines to use with caution or avoid concomitant medications are provided in view of the possible drug–drug interactions (see Appendix 5).

1.3.3 Rationale for the Combination of Venetoclax with Obinutuzumab

Obinutuzumab (Gazyva™ also known as GA101 and RO5072759) is a novel, humanized, type II glycoengineered monoclonal antibody (mAb) directed against the CD20 antigen, which is found on most malignant and benign cells of B-cell origin. Obinutuzumab was derived by humanization of the parental B Ly1 mouse antibody and subsequent glycoengineering, leading to the following characteristics: high-affinity binding to the CD20 antigen, low complement-dependent cytotoxicity activity, high direct cell death induction, high antibody-dependent cellular cytotoxicity, and antibody-dependent cellular phagocytosis.

Obinutuzumab was approved on 1 November 2013 by the U.S. Food and Drug Administration (FDA) for the treatment of patients with previously untreated CLL on the basis of the primary analysis of data from the pivotal study, BO21004/CLL11

(Goede et al. 2014). On 22 May 2014, the European Medicines Agency (EMA) adopted a positive opinion recommending a marketing authorization for obinutuzumab for the treatment, in combination with chlorambucil, of adult patients with previously untreated CLL with coexisting medical conditions that made them unsuitable for full-dose fludarabine based therapy.

1.3.4 Benefit-Risk Assessment

It is anticipated that because obinutuzumab and venetoclax exhibit different mechanisms of action, a combination of both drugs could improve tumor response and delay progression of disease or completely avoid the occurrence of resistance. Safety profiles of both drugs seem to be compatible, especially in light of a chemotherapy-free setting. The run-in phase of the study will provide initial safety data on the combination of obinutuzumab and venetoclax. The head-to-head design of the study, precautionary safety measures, and regular monitoring of safety by an independent Data Monitoring Committee (iDMC) and the sponsor enable early identification of safety signals in the study and minimize the risk to patients enrolled. In conclusion, it is considered that the benefit-risk ratio for this study is favorable.

2. OBJECTIVES

2.1 EFFICACY OBJECTIVES

The primary objective of Study BO25323/CLL14 is as follows:

- To determine efficacy by investigator-assessed PFS of a combined regimen of obinutuzumab + venetoclax compared with GClb in previously untreated patients with CLL who have coexisting medical conditions

The secondary efficacy objective of the study is as follows:

- To determine efficacy as assessed by additional outcome measures (as specified in Section 3.4.2, including PFS *assessed by Independent Review Committee [IRC]*, overall response, *complete response,* and MRD response rate as measured by allele-specific oligonucleotide polymerase chain reaction [ASO-PCR] (see secondary safety objective in Section 2.2)

Note: IRC-assessed PFS will be considered primary for United States regulatory purposes.

2.2 SAFETY OBJECTIVES

The safety objective for this study is as follows:

- To evaluate the safety of the combination of obinutuzumab and venetoclax, compared with GClb, in patients with previously untreated CLL and coexisting medical conditions, focusing on the nature, frequency, and severity of Grade 3 and 4 adverse events and of serious adverse events

2.3 PHARMACOKINETIC OBJECTIVE

The pharmacokinetic (PK) objective for this study is as follows:

- To characterize the pharmacokinetics of venetoclax and of obinutuzumab (including population PK [popPK] techniques). Standard NCA/descriptive TLGs and popPK approaches will be considered.

2.4 PATIENT-REPORTED OUTCOME OBJECTIVES

The quality of life (patient-reported outcome [PRO]) objectives for this study are as follows:

- To compare disease and treatment-related symptoms following treatment with the combination of obinutuzumab + venetoclax compared with GClb in patients with previously untreated CLL and coexisting medical conditions as measured by M.D. Anderson Symptom Inventory (MDASI-CLL; see Appendix 18)
- To evaluate changes in role functioning and global health status/quality of life (QoL) following treatment with the combination of obinutuzumab + venetoclax compared with GClb in patients with previously untreated CLL and coexisting medical conditions between arms as measured by European Organisation for Research and Treatment of Cancer Quality of Life Questionnaire Core 30 (EORTC QLQ-C30)

2.5 HEALTH ECONOMIC OBJECTIVE

The health economic objective for this study should be considered as a special PRO and is as follows:

- To compare the health utility effects of treatment with combination of obinutuzumab and venetoclax compared with GClb in patients with previously untreated CLL and coexisting medical conditions measured by the EuroQol 5 Dimension questionnaire (EQ-5D-3L)

2.6 EXPLORATORY OBJECTIVES

The exploratory objectives for this study are as follows:

- To assess MRD using new technologies, including flow cytometry and next-generation sequencing, to compare results with MRD measured by ASO-PCR
- To evaluate the relationship between PFS and MRD response rate
- To evaluate the relationship between various baseline prognostic markers and clinical outcome parameters
- To evaluate the relationship between biomarkers measured at baseline and disease progression to understand mechanisms of response and resistance

3. STUDY DESIGN

3.1 DESCRIPTION OF STUDY

This is an open-label, multicenter, randomized Phase III trial to compare the efficacy and safety of a combined regimen of obinutuzumab and venetoclax versus GClb in patients with CLL and coexisting medical conditions. Figure 1 illustrates the design of the study.

Initially, there will be a 12-patient safety run-in phase, (including at least 1 patient at high risk of developing TLS) wherein patients will receive obinutuzumab + venetoclax in a non-randomized fashion. After the twelfth patient has reached the end of Cycle 3, a formal review will be undertaken by Roche and the German CLL Study Group ([GCLLSG]; together, hereafter referred to as the Sponsor) and an iDMC.

The following are stopping criteria for the run-in phase of this study:

- One treatment-related death

  or

- One Grade 4 adverse event related to a clinical TLS despite protocol-specified prophylaxis, either following the administration of the first dose of venetoclax or during dose escalation

If any of these criteria are met, the main study will not be opened for recruitment, and the Sponsor will then re-evaluate the study design and amend the protocol accordingly. If the stopping criteria are not met, randomization into the trial will commence.

Figure 1 Study Schema

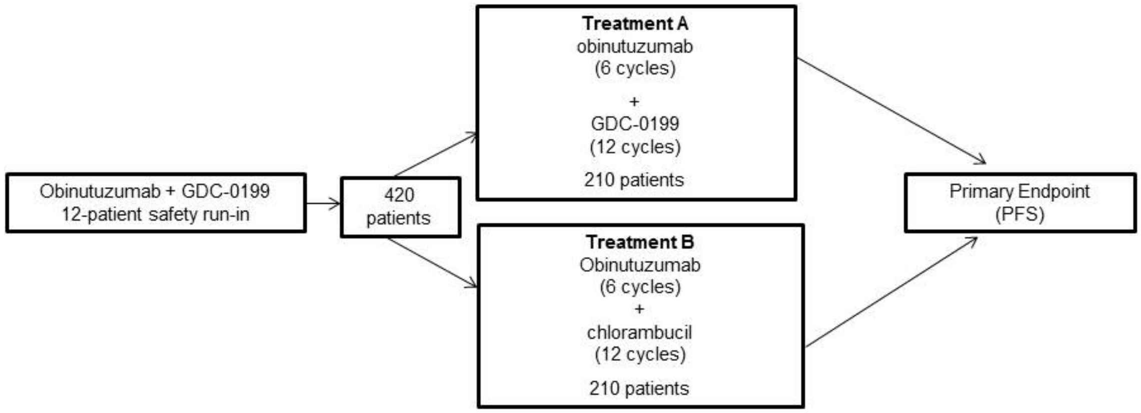

GDC-0199 = venetoclax; PFS = progression-free survival.

3.1.1 Assessment of Efficacy

Response will be assessed according to the 2008 International Workshop on Chronic Lymphocytic Leukemia (IWCLL) guidelines. An IRC that is blinded to treatment groups will assess all patients for progression on the basis of peripheral blood counts, bone

marrow biopsy results, and reports of physical examination. Computed tomography (CT) scan reports from pre- and post-treatment will be provided to the IRC to support the evaluation.

3.1.2 Summary of Study Procedures

Written informed consent will be obtained before any study specific procedures are undertaken.

3.1.2.1 Screening/Baseline Tests

All screening assessments documented in the schedule of assessments will be performed a maximum of 28 days prior to either starting obinutuzumab + venetoclax, the safety run-in phase, or to randomization into the main study (Appendix 2 and Appendix 3). Those patients who fail screening based on no immediate requirement for treatment can be rescreened in the future when their CLL is in need of treatment. Similarly, potential patients who fail screening because of recent infection (patients with infections requiring IV treatment [Grade 3 or 4] within the last 2 months prior to enrollment) may be rescreened once their infections resolve (see Section 4.1.2).

Randomization will be performed by an interactive voice-/web-based system (IxRS). Patients will be assigned to the two treatment arms via a 1:1 randomization procedure. Patients will be stratified by Binet stage and by country/region. Baseline tests will be used to assess the Binet Stage. If a patient's Binet stage progresses between screening and randomization, then the Binet stage at the screening timepoint should be used for stratification.

3.1.2.2 Treatment Period

Table 1 Treatment Arm A: Obinutuzumab + Venetoclax Study Visits

Cycle, Day	Dose
Obinutuzumab[a]	
Cycle 1, Day 1	100 mg or 1000 mg (follow splitting rules)
Cycle 1, Day 2 [b]	900 mg (if 100 mg on Cycle 1 Day 1)
Cycle 1, Day 8	1000 mg
Cycle 1, Day 15	1000 mg
Cycles 2–6, Day 1	1000 mg
Venetoclax [c, d]	
Cycle 1, Day 22–28	20 mg daily
Cycle 2, Day 1–Day 7	50 mg daily
Cycle 2, Day 8–Day 14	100 mg daily
Cycle 2, Day 15–Day 21	200 mg daily
Cycle 2, Day 22–Day 28	400 mg daily
Cycle 3, Day 1–end of Cycle 12	400 mg daily

IV = intravenous; TLS = tumor lysis syndrome.

Note: Cycles will comprise 28 days. Treatment for obinutuzumab will be for six cycles, and venetoclax will end at Cycle 12.

[a] IV infusion. Overnight hospitalization may be required on Day 1 following the first infusion of obinutuzumab (100 mg).

[b] Only the first dose (1000 mg) of obinutuzumab drug administration can be split over 2 days. Two infusion bags should be prepared for the infusion on Days 1 and 2 (100 mg for Day 1 and 900 mg for Day 2). If the first bag is completed without modifications of the infusion rate or interruptions, the second bag may be administered on the same day (no dose delay necessary, no repetition of premedication), provided that appropriate time, conditions, and medical supervision are available throughout the infusion. If there are any modifications of the infusion rate or interruptions during the first 100 mg, the second bag should be administered the following day. If patients require a delay of greater than 24 hours between the 100- and 900-mg infusions of obinutuzumab, please consult Section 4.3.1.3 and the Obinutuzumab Investigator's Brochure regarding drug stability.

[c] The 20 mg and 50 mg doses of venetoclax will be administered in the hospital for patients who are at high risk of TLS, or if indicated to hospitalize, and thereafter at home daily for 7 days. The dose will increase every 7 days to the target dose of 400 mg, and venetoclax will be administered at home unless a patient is indicated to hospitalize.

[d] Oral tablets.

Guidance on patient management for TLS prophylaxis prior to and following venetoclax dosing is provided in Section 5.1.3.1.

Because it is possible that de-bulking from obinutuzumab therapy before starting venetoclax dosing already significantly reduces the risk of TLS, TLS prophylactic

measures will be re-assessed at the time of the first safety review conducted by the iDMC (Section 9.4.2).

Table 2 Treatment Arm B: Obinutuzumab + Chlorambucil

Cycle, Day	Dose
Obinutuzumab	
Cycle 1, Day 1 [a, b]	100 mg or 1000 mg (follow splitting rules)
Cycle 1, Day 2	900 mg (if 100 mg on Cycle 1 Day 1)
Cycle 1, Day 8	1000 mg
Cycle 1, Day 15	1000 mg
Cycles 2–6, Day 1	1000 mg
Chlorambucil [c]	
Cycle 1–Cycle 12, Day 1	0.5 mg/kg oral
Cycle 1–Cycle 12, Day 15	0.5 mg/kg oral

Note: Cycles will comprise 28 days. Treatment for obinutuzumab will be for six cycles and 12 cycles for chlorambucil.

[a] Intravenous infusion. Overnight hospitalization may be required on Day 1 following the first infusion of obinutuzumab (100 mg).

[b] Only the first dose (1000 mg) of obinutuzumab drug administration can be split over 2 days. Two infusion bags should be prepared for the infusion on Days 1 and 2 (100 mg for Day 1 and 900 mg for Day 2). If the first bag is completed on Day 1 without modifications of the infusion rate or interruptions, the second bag may be administered on the same day (no dose delay necessary, no repetition of premedication), provided that appropriate time, conditions, and medical supervision are available throughout the infusion. If there are any modifications of the infusion rate or interruptions during the first 100 mg, the second bag should be administered the following day. If patients require a delay of greater than 24 hours between the 100- and 900-mg infusions of obinutuzumab, please consult Section 4.3.1.3 and the Obinutuzumab Investigator's Brochure regarding drug stability.

[c] Chlorambucil is given orally on Days 1 and 15 at a dose of 0.5 mg/kg.

From Cycle 2 (treatment arm B only) onward, the patients have the option to have the Day 15 hematology and biochemistry tests performed at a local laboratory. The results will be communicated to the investigator, and, if satisfactory, the investigator will instruct the patient either to take the Day 15 chlorambucil dose at home or to return to the clinic for a formal visit.

3.1.2.3 Monitoring of Disease Progression during Treatment Period

All patients should be continually monitored for disease progression (PD) during the treatment period and this should be clearly documented in the electronic Case Report Form (eCRF). Interim staging assessments will be performed at Cycle 4, Day 1; Cycle 7, Day 1; and Cycle 9, Day 1. In addition to an assessment of hematological status, this will also include a full physical examination to assess any lymphadenopathy and hepatomegaly/splenomegaly.

Unless disease progression is confirmed, the patient is withdrawn due to toxicity, or the patient has died, patients should receive up to six cycles of study treatment of obinutuzumab and 12 cycles of either venetoclax or chlorambucil.

3.1.2.4 Post-Treatment Follow-Up Visits

All patients will be followed until 5 years after last patient randomization. Response will be followed at all visits by clinical/laboratory signs and symptoms until progression is identified. A CT scan should be performed 3 months after end of treatment in order to confirm CR and PR if lymphadenopathy was present at baseline. In those patients who have achieved a CR or CRi, a bone marrow aspirate and biopsy will be obtained. If PD is detected by physical examination in the absence of any objective hematological progression, a CT scan of the involved nodes will be performed. After PD, patients will be followed until the next anti-leukemic treatment is given and then for survival for a maximum of 5 years from the last patient enrolled unless all patients have died. To capture PROs post progression among those receiving subsequent therapies, PRO assessment will be made at the time of the subsequent treatment visit, but before any procedures or drug infusions are performed. A schedule of assessments is provided in Appendix 2 and Appendix 3.

3.2 END OF STUDY

The approximate length of study will be 6 years and 8 months calculated from an estimated 20-month recruitment period, and the end of study is defined below.

The end of this study is defined as 5 years from last patient enrolled (unless all patients have died).

3.3 RATIONALE FOR DESIGN

3.3.1 Rationale for Dose and Schedule

3.3.1.1 Venetoclax

Venetoclax dosing for this study was based on the experience from the Phase I dose-escalation Study M12-175, which examined single-agent venetoclax in relapsed and refractory patients with CLL and NHL, and the safety analysis of 58 patients enrolled in the study after enhanced TLS prophylaxis measures were introduced and who completed the dosing ramp-up period.

This experience has determined that starting venetoclax at 20 mg for 1 week followed by a gradual ramp-up of venetoclax over the period of 5 weeks is an adequate measure in order to safely administer venetoclax. The dosing regimen proved to reduce the risk for TLS by more gradually reducing the leukemia cell burden prior to administration of the full target dose with no loss of effect.

The starting dose of venetoclax in Study M12-175 was 20 mg followed by weekly increases in dose levels to a maximum dose of 600 mg. However, the dose to be used in combination with anti-CD20 monoclonal antibodies and other agents may be below

the maximum dose established as a single agent. Preliminary data from Study M12-175 show that the 400-mg venetoclax dose as a single agent results in exposure that causes > 80% reduction in lymphocyte counts, tumor size, and bone marrow infiltrates in most patients.

In Arm A of Study M12-175 (relapsed/refractory CLL/SLL treated with venetoclax monotherapy), the ORR, consisting of CR/CRi plus PR was 77% (60 of 78 evaluable patients) with CR/CRi in 18 patients (23%) and PR in 42 patients (54%). The ORR was 75% in the 24 patients with unmutated IGHV status with 29% achieving CR/CRi.

The ORR was 79% in the 19 patients harboring the deletion of the p13 locus on chromosome 17 with 26% achieving CR/CRi. The ORR was 76% in the 41 patients who were fludarabine-refractory with 9 of 41 patients (22%) achieving CR/CRi. In Study M13-365 (relapsed CLL treated with venetoclax + rituximab), the ORR was 78% (14 of 18 evaluable patients) with 7 of 18 patients (39%) achieving CR/CRi and 7 of 18 patients (39%) achieving PR (see Section 1.3.1).

The number of cycles of dosing (combination therapy × six 28-day cycles followed by monotherapy for six 28-day cycles) is designed to provide a duration of treatment consistent with other therapies for CLL that have been shown to be sufficient to provide durable responses.

In the single-agent, Phase I, dose-escalation Study M12-175, responses to single-agent venetoclax have been seen to improve over time at the 36–50-week timepoint as treatment continues; therefore, the treatment duration has been determined to be 1 year.

To date, no evidence of unexpected late toxicities has emerged from continued treatment with venetoclax beyond 6 months.

3.3.1.2 Obinutuzumab

The approved dose of obinutuzumab for front-line treatment will be used in this study.

- Cycle 1: 1000 mg Days 1 (or split dose Day 1 and Day 2), 8, and 15 (see Section 4.3.2 for details)
- Cycles 2–6: 1000 mg on Day 1

Treatment will be on a 28-day cycle, for a total of six cycles.

Evaluation of potential PK interactions between venetoclax and obinutuzumab is incorporated into the PK assessment plan in an ongoing Phase Ib study (Study GP28331).

3.3.1.3 Chlorambucil

The rationale for the chlorambucil dose and schedule are based on the findings from the GCLLSG CLL5 trial. This trial demonstrated that chlorambucil is equally effective as

fludarabine monotherapy in elderly (and in the subgroup of medically unfit) patients with CLL. In the GCLLSG-CLL5 trial protocol, chlorambucil was scheduled with 0.4-mg/kg body weight initially with a subsequent increase of the dose to a maximum of 0.8-mg/kg body weight. However, there was no such dose escalation in the majority of patients, mostly due to adverse events, and median PFS was relatively long (17 months). In clinical practice, chlorambucil may be given at higher doses than 0.5 mg/kg body weight. Higher doses can be achieved with a fixed starting dose or with dose escalations. Starting with a fixed high dose of chlorambucil (e.g., 0.8-mg/kg body weight) could be of risk in elderly and medically unfit patients because it will increase toxicity without necessarily ensuring better efficacy. If the outcome in the different trials is compared, there are no convincing data that higher doses of chlorambucil are more effective than low-dose schedules. Although there is no definitive conclusion that efficacy of chlorambucil significantly increases with duration of treatment, the majority of trials used up to 12 cycles of treatment. To ensure clinical equipoise in the duration of therapy received in both treatment arms in the planned trial, a total of 12 cycles of chlorambucil therapy will be used in the control arm as well.

3.3.2 Rationale for the Combination of Venetoclax with Obinutuzumab

Obinutuzumab is a novel, humanized, type II glycoengineered monoclonal antibody (mAb) directed against the CD20 antigen, which is found on most malignant and benign cells of B-cell origin. Obinutuzumab was derived by humanization of the parental B Ly1 mouse antibody and subsequent glycoengineering, leading to the following characteristics: high-affinity binding to the CD20 antigen, low complement-dependent cytotoxicity activity, high direct cell death induction, high antibody-dependent cellular cytotoxicity, and antibody-dependent cellular phagocytosis.

Because obinutuzumab and venetoclax exhibit different mechanisms of action, a combination of both drugs could delay or completely avoid the occurrence of resistance. The safety profiles of both drugs seem to be compatible, especially in light of a chemotherapy-free setting.

3.3.3 Rationale for Patient Population

The target population for the study is patients with previously untreated CLL and coexisting medical conditions. Such patients are compromised by these conditions or an age-related decline in organ function.

Various scores for systematic evaluation of the coexisting medical conditions of geriatric oncology patients have been developed and evaluated over the last decades. In the Cumulative Illness Rating Scale (CIRS) score, the number and severity of the coexisting medical conditions are qualitatively documented and quantified (Linn et al. 1968). Several studies have documented a high reliability and validity of the CIRS score (Miller et al. 1992; Waldman et al. 1992; Parmelee et al. 1995). For a geriatric non-oncology patient population, CIRS threshold scores have been defined, which

permit differentiation of different patient groups with different risks of hospitalization and mortality (Keller and Potter 1994). In geriatric patients, the CIRS score correlates with mortality, rate, and duration of hospitalization; need for medication; functional impairment; and psychological status (Miller et al. 1992; Keller and Potter 1994) as well as with post-mortem findings (Conwell et al. 1993). In some more recent studies, the extent of coexisting conditions as determined by the CIRS score has proved to be prognostically relevant for the overall survival of tumor patients (Firat et al. 2002; Hall et al. 2002). Two further studies also describe the distribution of CIRS scores in a sample of older cancer patients with different cancers (Extermann et al. 1998; Chen et al. 2003). In approximately half of the patients studied, a total CIRS score of 0–6 (i.e., no to mild coexisting medical conditions) was determined, while in the other half, the total CIRS score was 7–18 (i.e., moderate to high coexisting medical conditions) (Extermann et al. 1998). A CIRS score of 6 or higher is reached in the presence of multiple coexisting medical conditions. Patients with CLL who present with such a coexisting medical condition burden have been reported to have inferior outcome compared with patients with CLL who are not suffering from multiple conditions (Goede et al. 2005; Cramer et al. 2006). A CIRS threshold of six, thus, may be a suitable risk discriminator in patients with CLL. With the help of the CIRS score, the extent of coexisting medical conditions for a potential study patient can be assessed qualitatively and quantitatively with relatively little effort before the patient is admitted to the study. Appendix 6 outlines CIRS criteria.

A possible surrogate for a decline in functional organ reserve is the glomerular filtration rate. This can be estimated by calculating the creatinine clearance (CrCl) using the Cockcroft-Gault formula (Appendix 7).

Currently, there is limited clinical experience for venetoclax in patients with CrCl 30–50mL/min. Therefore, these patients should receive additional consideration by the investigator with regard to their management, including the decision on whether to administer IV hydration and to hospitalize the patient at the initial dosing as well as at each first dose during the ramp-up period.

3.3.4 Rationale for Control Group

Obinutuzumab was approved on 1 November 2013 by the U. S. Food and Drug Administration (FDA) and on 22 May 2014, the European Medicines Agency (EMA) granted a positive opinion for the registration of obinutuzumab for the treatment in combination with chlorambucil, of adult patients with previously untreated CLL with coexisting medical conditions that make them unsuitable for full-dose fludarabine-based therapy, on the basis of the primary analysis of data from the pivotal study, BO21004/CLL11.

The combination of chlorambucil with obinutuzumab is considered to be the standard-of-care in the elderly unfit patient population with coexisting medical conditions (Section 1.1 and NCCN Guidelines, NHL 2014 v.2).

3.3.5 Rationale for Minimal Residual Disease Assessments

The eradication of MRD is becoming the desirable endpoint in the treatment of CLL (Böttcher et al. 2012). Evidence suggests that patients who achieve a MRD-negative remission have significantly lower relapse rates, longer PFS, and better OS. MRD has been demonstrated to have a high correlation with PFS in multiple studies. MRD data in the pivotal CLL8 study (ML17102) showed that significantly more patients in the rituximab + FC group achieved MRD-negative status compared with the FC-only group (MRD response rate 68% versus 31%; $p < 0.0001$; [Böttcher et al. 2012]). Patients reaching MRD-negative status had a better PFS outcome irrespective of the treatment they received ($HR = 4.46$; $p < 0.0001$). As a result, understanding the relationship of the peripheral blood MRD status and PFS is important to the use of MRD response rate as a primary endpoint in future CLL trials.

MRD can be measured in peripheral blood or bone marrow, and both will be collected in this study. Previous studies have shown that some patients who were MRD-negative in peripheral blood at the final response assessment were MRD-positive in bone marrow (Böttcher et al. 2012; Goede et al. 2014). Reasons for this difference may be the eradication of CLL in the blood and the limited ability of current therapies to efficiently eradicate the disease in bone marrow. Therefore, MRD negativity will be measured in bone marrow in responding patients on this study at the end-of-treatment response assessment after completion of combination therapy and again at the completion of treatment. Refer to Appendix 13 for more detail on the methodology for the MRD assessments in this study.

3.3.6 Rationale for Biomarker Assessments

Venetoclax inhibits the ability of cancer cells to evade cell death (apoptosis) by blocking the activity of the anti-apoptotic protein Bcl-2. Nonclinical studies have demonstrated a pattern of response to venetoclax on the basis of the levels of Bcl-2 family proteins. High levels of Bcl-2 and low levels of Mcl-1 are generally predictive of response to this drug in vitro. In addition, high levels of at least one pro-apoptotic “sensor,” such as Noxa or Bim, are required. Measurement of relevant RNAs and proteins (including those in the Bcl-2 family) in CLL cells will be examined pre-treatment and at the time of progression for correlation with outcome.

Additionally, several prognostic markers have been described that can be used as stratification factors for CLL therapies. These include del(17p), del(11q), del(13q14), trisomy 12, IGHV, and TP53 mutations as well as serum parameters (thymidine kinase, β2 microglobulin). There is increasing evidence from prospective clinical trials that detection of certain chromosomal deletions has prognostic significance in CLL. For example, patients with leukemia cells that have del(17p) and/or p53 mutations have an inferior prognosis and appear resistant to standard chemotherapy regimens. Therefore, detection of these and other cytogenetic abnormalities could have prognostic value and may guide therapeutic decisions (Fink et al. 2013; Goede et al. 2014; Zenz et al. 2009).

Various studies have demonstrated that other molecular characteristics can influence the prognosis of patients with CLL with respect to time to progression and survival. Therefore, additional exploratory prognostic markers, such as rare cytogenetic abnormalities as detected by metaphase cytogenetics and telomere length will also be evaluated. Telomere length is a potential prognostic marker that predicts sensitivity to DNA damaging agents, such as Clb (Mansouri et al. 2013).

CLL remains an incurable disease because of residual CLL cells in the microenvironment, especially in lymph nodes. Interaction of CD40:CD40L induces several pro-survival pathways and, thereby, up-regulates mitochondrial, anti-apoptotic molecules, including BCL2, MCL1, or BCL-XL, which can affect sensitivity to venetoclax. Analyses will be performed to determine in vitro venetoclax drug sensitivity in CLL cells after stimulation with CD40L-expressing feeder cells as well as western blot analysis for Bcl-2, Bcl-xL, and Mcl-1. This experiment will explore the hypothesis that CD40:CD40L interaction in the lymph nodes will induce the pro-survival pathway and effect drug sensitivity to venetoclax.

Recurrent somatic mutations, (i.e., in genes such as *NOTCH1*, *SF3B1*, *TP53*), which have recently been identified in CLL in small subsets of patients (approximately 2%–10% of patients), might evolve over time (clonal evolution) and associate with clinical long-term prognosis (Dreger et al. 2013; Schnaiter et al. 2013; Stilgenbauer et al. 2014). A blood sample will be used to sequence commonly mutated genes mentioned above, as well as genomic variation in genes associated with the cellular DNA damage response, cellular key pathways in CLL, (e.g., B-cell receptor signaling), and genes encoding for new treatment targets, including BCL2.

In addition, resistance mechanisms will also be evaluated by genome-wide analyses (e.g., whole exome sequencing, whole genome sequencing, RNA sequencing) on paired samples taken pre-treatment and at the time of relapse in patients who consent to the GCLLSG biobanking program.

Biomarkers that are described here may have both predictive and prognostic value; both arms of the study will be evaluated, and their potential association with disease progression will also be explored. These studies may help identify responsive patient populations and develop better therapies for patients with CLL. The analyses described in this section are exploratory and will be done retrospectively.

3.3.7 Rationale for Chronic Lymphocytic Leukemia Diagnostic Laboratory Assessments

Lymphocyte Count

The clinical diagnosis of CLL requires an absolute lymphocytosis with a lower threshold of at least 5000 mature-appearing lymphocytes/μL in the peripheral blood. Lymphocyte counts may fluctuate from time of diagnosis to the point at which treatment is required.

Lymphocyte Immunophenotyping

Patients must have documented CD20-positive CLL according to the IWCLL criteria (see Appendix 12) and the Matutes Scoring system (Matutes et al. 1994; see Appendix 17). CLL cells co-express the T-cell antigen CD5 and B-cell antigen (CD19, CD20, and CD23). CD20 and CD16 median fluorescence intensity should be used to facilitate prediction of IRR risk after obinutuzumab infusion. The levels of surface immune-globulins, CD20 and CD79b, are characteristically low compared with those found on normal B cells. Each B cell is monoclonal with regard to expression of either κ or λ. Variations of the intensity of expression of these markers may exist and do not exclude entry in the study.

3.3.8 Rationale for Pharmacokinetic Assessments

PK is defined in this protocol as a dual timepoint drug level testing (18 mL blood at Cycle 4, Day 1 in all patients receiving venetoclax [Arm A]; see Appendix 4).

Venetoclax is a small molecule administered orally and is primarily metabolized by CYP3A4, and obinutuzumab is a mAb administered by intravenous (IV) infusion. mAbs do not interact directly with cytochrome P450 (CYP) isoforms and other metabolizing enzymes. Therefore, the risk for obinutuzumab to significantly alter the pharmacokinetics of venetoclax is expected to be low.

Tumor burden affects the clearance of obinutuzumab, especially at the beginning of treatment. With decreasing tumor burden, the clearance was shown to reach an asymptote. This finding is most likely the result of proteolytic metabolic clearance. Consequently, some patients with a high tumor burden may appear to clear the drug from the blood faster than patients with a low tumor burden because obinutuzumab binds to the CD20+tumor cells and is effectively removed from the blood. As a result, the clearance of the drug is expected to vary with time because repeated doses of obinutuzumab should reduce the number of CD20+tumor cells. Venetoclax has the potential to alter tumor burden and, therefore, affect the clearance of obinutuzumab. The drug interaction potential between venetoclax and obinutuzumab is being investigated in the Phase Ib Study GP28331. Sparse PK samples for venetoclax and obinutuzumab will also be collected in all patients in Arm A (obinutuzumab+venetoclax) of this study to assess any interaction and explore potential exposure–response (efficacy or safety) relationships.

3.3.9 Rationale for Patient-Reported Outcome Assessments

Unfit patients with CLL experience a symptom burden from the underlying disease process that is compounded by the side effects of currently available therapies. These symptoms and treatment-related side effects can impact function and, subsequently, health-related quality of life. There is strong reason to believe that if the anticipated clinical benefit is observed between the combination of venetoclax+obinutuzumab in the trial, patients might report an accompanying improvement in distinct key symptoms of the disease (i.e., reduction in fatigue, reduction in nodular pain, and decrease in night

sweats). PROs will be used to comprehensively capture a patient's report of disease symptom changes as well as evaluate the impact of treatment-related side effects. Specifically, with PFS being a primary clinical endpoint, understanding the time to disease progression through PROs and how patients perceive disease symptoms during this time can be used to support PFS. Additionally, quantifying the importance of MRD from the patient perspective on change in symptom burden could better support this novel endpoint.

3.4 OUTCOME MEASURES

3.4.1 Efficacy Outcome Measures

The primary efficacy outcome measure for this study is as follows:

- PFS, defined as the time from randomization to the first occurrence of progression, relapse, or death from any cause as assessed by the investigator. Disease progression will be assessed by the investigators using the IWCLL criteria (2008).

3.4.2 Secondary Efficacy Outcome Measures

The secondary efficacy outcome measures for this study are as follows:

- PFS based on IRC-assessments (primary outcome for U.S. regulatory purposes), defined as the time from randomization to the first occurrence of progression or relapse or death from any cause
- ORR (defined as rate of a clinical response of CR, CRi, or PR) at the completion of treatment assessment, as determined by the investigator according to the IWCLL guidelines (2008)
- *Complete response rate (CRR; defined as rate of a clinical response of CR or CRi) at the completion of treatment assessment, as determined by the investigator according to the IWCLL guidelines (2008)*
- MRD response rate (determined as the proportion of patients with MRD-negativity) measured in the peripheral blood at the completion of treatment assessment and MRD response rate as measured in the bone marrow at the completion of treatment, both measured by ASO-PCR
- ORR at completion of combination treatment response assessment (Cycle 7, Day 1 or 28 days after last IV infusion)
- MRD response rates in the peripheral blood at completion of combination treatment assessment (Cycle 9, Day 1 or 3 months after last IV infusion) and also MRD response rate in the bone marrow, both as measured by ASO-PCR

Note: the secondary endpoint analyses will be on the basis of MRD assessment performed centrally by ASO-PCR with MRD negativity defined using a cutoff of 10^{-4} (less than 1 cell in 10,000 leukocytes). As well as evaluating MRD response rate, it will further be evaluated using results of both measures, where a patient will be considered MRD-positive if either blood or bone marrow result is positive or both are missing. MRD

response rates at other timepoints during treatment and follow-up where MRD is measured in the peripheral blood will also be summarized.

- OS, defined as the time between the date of randomization and the date of death due to any cause
- Duration of objective response, defined as the time from the first occurrence of a documented objective response to the time of PD as determined by the investigator or death from any cause
- Best response achieved (CR, CRi, PR, stable disease, or PD) up to and including the assessment at completion of treatment assessment (within 3 months of last day of treatment)
- Event-free survival (EFS), defined as the time between date of randomization and the date of disease progression/relapse on the basis of investigator-assessment, death, or start of a new anti-leukemic therapy
- Time to next anti-leukemic treatment, defined as time between the date of randomization and the date of first intake of new anti-leukemic therapy

3.4.3 Safety Outcome Measures

The safety outcome measures for this study are as follows:

- Nature, frequency, and severity of adverse events and serious adverse events
- Changes in vital signs, physical findings, and clinical laboratory results during and following study treatment
- Lymphocyte immunophenotyping and incidence of human–anti-human antibodies
- Premature withdrawals

3.4.4 Pharmacokinetic Outcome Measures

The PK outcome measure for this study is follows:

- Apparent clearance, apparent volume of distribution, and/or other appropriate PK parameters of venetoclax and of obinutuzumab characterized with the use of popPK techniques. Standard NCA/descriptive TLGs and popPK approaches will be considered.

3.4.5 Pharmacodynamic Outcome Measures

For each visit at which CD19+, CD5+, and CD19+CD5– B-cell measurements are taken, B-cell data will be listed for individual patients by treatment arm.

3.4.6 Patient-Reported Outcome Measures

The PRO measures for this study are as described below. The first assessment will be done during the first obinutuzumab infusion, and PROs will be followed until end of study as defined by 5 years after the last randomized patient:

- To evaluate changes following treatment in disease and treatment-related symptoms in MDASI-CLL scores

- To evaluate changes in role functioning and global health status/QoL scales following treatment with the EORTC QLQ-C30

3.4.7 Health Economic Outcome Measure

The health economic outcome measure for this study is as follows:

- The EQ-5D-3L questionnaire

3.4.8 Exploratory Outcome Measures

The exploratory outcome measures for this study are as follows (provided these data are available in a sufficiently timely and complete form to perform these analyses at the time of the final primary analysis):

- MRD negativity in peripheral blood, measured using new technologies, including flow cytometry and next-generation sequencing with MRD negativity defined using a cutoff of 10^{-4} (less than 1 cell in 10,000 leukocytes) for comparison with ASO-PCR, and secondly by the limit of sensitivity of each of the above technologies
- Relationship between MRD and PFS on the basis of peripheral blood assessed using ASO-PCR
- Relationship between various baseline markers and clinical outcome parameters in patients from both arms of the study (including but not limited to CLL FISH [17p-, 11q-, 13p-, +12q], IGHV mutation status, p53 mutation status, serum parameters, Bcl-2 expression, and other CLL disease markers)

4. MATERIALS AND METHODS

4.1 PATIENTS

4.1.1 Inclusion Criteria

A total of 420 patients will be enrolled in the randomized part of the study, and 12 patients will be enrolled in the safety run-in phase. Patients must meet the following criteria for study entry:

- Have documented previously untreated CLL according to IWCLL criteria (Appendix 12)
- CLL that requires treatment according to the IWCLL criteria
- Total CIRS score >6 or CrCl <70 mL/min
- Adequate marrow function independent of growth factor or transfusion support within 2 weeks of screening as follows, unless cytopenia is due to marrow involvement of CLL:

  Absolute neutrophil count $\geq 1.0 \times 10^9$/L

  Platelet counts $\geq 30 \times 10^9$/L; in cases of thrombocytopenia clearly due to marrow involvement of CLL (per the discretion of the investigator); platelet count should be $\geq 10 \times 10^9$/L if there is bone marrow involvement

  Total hemoglobin ≥ 9 g/dL (without transfusion support, unless anemia is due to marrow involvement of CLL)

- Adequate liver function as indicated by a total bilirubin, AST, and ALT ≤ 2 times the institutional upper limit of normal (ULN) value unless directly attributable to the patient's CLL
- For those patients with a screening lymphocyte count < 5,000 cells/μL, historical data that confirms a lymphocyte count ≥ 5,000 cells/μL at the time of diagnosis is required
- 18 years of age or older
- Life expectancy > 6 months
- Signed informed consent and, in the investigator's judgment, able to comply with the study protocol
- For women who are not postmenopausal (≥ 12 months of non-therapy-induced amenorrhea) or surgically sterile (absence of ovaries and/or uterus): agreement to remain abstinent or use single or combined contraceptive methods that result in a failure rate of < 1% per year during the treatment period and for at least 30 days after the last dose of venetoclax or 18 months after the last dose of obinutuzumab, whichever is longer

    Abstinence is only acceptable if it is in line with the preferred and usual lifestyle of the patient. Periodic abstinence (e.g., calendar, ovulation, symptothermal, or post-ovulation methods) and withdrawal are not acceptable methods of contraception.

    Examples of contraceptive methods with a failure rate of < 1% per year include tubal ligation, male sterilization, hormonal implants, established, proper use of combined oral or injected hormonal contraceptives, and certain intrauterine devices. Alternatively, two methods (e.g., two barrier methods such as a condom and a cervical cap) may be combined to achieve a failure rate of < 1% per year. Barrier methods must always be supplemented with the use of a spermicide.

- For men: agreement to remain abstinent or use a condom plus an additional contraceptive method that together result in a failure rate of < 1% per year during the treatment period and for at least 90 days after the last dose of venetoclax or 18 months after the last dose of obinutuzumab, whichever is longer, and agreement to refrain from donating sperm during the treatment period and for at least 90 days after the last dose of venetoclax

    Men with a pregnant partner must agree to remain abstinent or use a condom for the duration of the pregnancy.

    Abstinence is only acceptable if it is in line with the preferred and usual lifestyle of the patient. Periodic abstinence (e.g., calendar, ovulation, symptothermal, or post-ovulation methods) and withdrawal are not acceptable methods of contraception.

4.1.2 Exclusion Criteria

Patients who meet any of the following criteria will be excluded from study entry:

- Transformation of CLL to aggressive NHL (Richter's transformation or pro-lymphocytic leukemia).
- Known central nervous system involvement.
- Patients with a history of confirmed progressive multifocal leukoencephalopathy (PML).
- An individual organ/system impairment score of 4 as assessed by the CIRS definition limiting the ability to receive the treatment regimen of this trial with the exception of eyes, ears, nose, throat organ system (note that symptoms related to CLL should not be included in the patient's screening CIRS score). Investigators should consult the General Rules for Severity Rating as well as the Organ-Specific Categories in Appendix 6 when assigning scores for certain conditions (i.e., pulmonary embolism) and consider the level of morbidity associated with a patient's condition.
- Patients with uncontrolled autoimmune hemolytic anemia or immune thrombocytopenia.
- Inadequate renal function: CrCl $<$ 30 mL/min.
- History of prior malignancy, except for conditions as listed below if patients have recovered from the acute side effects incurred as a result of previous therapy:

    Malignancies surgically treated with curative intent and with no known active disease present for $\geq$ 3 years before randomization.

    Adequately treated non-melanoma skin cancer or lentigo maligna without evidence of disease.

    Adequately treated cervical carcinoma in situ without evidence of disease.

    Surgically/adequately treated low grade, early stage, localized prostate cancer without evidence of disease.

- Patients with infections requiring IV treatment (Grade 3 or 4) within the last 2 months prior to enrollment.
- History of severe allergic or anaphylactic reactions to humanized or murine monoclonal antibodies or known sensitivity or allergy to murine products.
- Hypersensitivity to chlorambucil, obinutuzumab, or venetoclax or to any of the excipients (e.g., trehalose).
- Pregnant women and nursing mothers.
- Vaccination with a live vaccine $\leq$ 28 days prior to randomization.
- Prisoners or patients who are institutionalized by regulatory or court order or persons who are in dependence to the Sponsor or an investigator.
- History of illicit drug or alcohol abuse within 12 months prior to screening, in the investigator's judgment.

- Positive test results for chronic hepatitis B virus (HBV) infection (defined as positive HBsAg serology).

  Patients with occult or prior HBV infection (defined as negative hepatitis B surface antigen [HBsAg] and positive total hepatitis B core antibody [HBcAb]) may be included if HBV DNA is undetectable, provided that they are willing to undergo monthly DNA testing. Patients who have protective titers of hepatitis B surface antibody (HBsAb) after vaccination or prior but cured hepatitis B are eligible.

- Positive test result for hepatitis C (hepatitis C virus [HCV] antibody serology testing).

  Patients who are positive for HCV antibody are eligible only if PCR is negative for HCV RNA.

- Patients with known infection with human immunodeficiency virus (HIV) or Human T-Cell Leukemia Virus 1 (HTLV-1).

  In countries where mandatory testing by health authorities is required, HIV testing will be performed.

  HTLV testing is required in patients from endemic countries (Japan, countries in the Caribbean basin, South America, Central America, sub-Saharan Africa, and Melanesia).

- Any serious medical condition or abnormality in clinical laboratory tests that, in the investigator's judgment, precludes the patient's safe participation in and completion of the study.

- Patients who have received the following agents:

  Strong and moderate CYP3A inhibitors (see Appendix 5 for examples) within 7 days prior to the first dose of study drug administration.

  Strong and moderate CYP3A inducers (see Appendix 5 for examples) within 7 days prior to the first dose of study drug administration.

  Consumed grapefruit, grapefruit products, Seville oranges (including marmalade containing Seville oranges), or star fruit within 3 days prior to the first dose of study drug and throughout venetoclax administration.

- Inability to swallow a large number of tablets.

4.2 METHOD OF TREATMENT ASSIGNMENT AND BLINDING

This is an open-label study. Initially, there will be a 12-patient safety run-in phase, (including at least 1 patient at high risk of developing TLS) wherein patients will receive obinutuzumab + venetoclax in a non-randomized fashion. After the 12th patient has reached the end of Cycle 3, a formal review will be undertaken by the Sponsor and an iDMC. After the completion of this run-in phase, including full review by the Sponsor and the iDMC, full randomization into the trial will begin.

Randomization will be performed by an IxRS. Patients will be assigned in 1:1 ratio to one of the two treatment arms through a block stratified randomization procedure.

The randomization scheme will ensure approximately equal sample sizes in the two treatment groups in regard to the following stratification factors:

- Binet stage (3 levels): A, B, or C
- Geographic region (US/Canada/Central America; Australia/New Zealand; Western Europe; Central and Eastern Europe; or Latin America).

A unique patient number will be assigned to all patients. This patient number will be used to identify the patient in the electronic data capture (EDC) system and all other data sources.

The iDMC will review unblinded safety data by treatment arm for the purpose of interim safety reviews and the planned interim analyses of efficacy. The Sponsor and study team will not have access to the unblinded information reviewed by the iDMC. Assessments by the IRC will be blinded to treatment arm.

4.3 STUDY TREATMENT

4.3.1 Formulation, Packaging, and Handling

4.3.1.1 Venetoclax

Venetoclax will be supplied by the Sponsor. For information on the formulation, packaging, and handling of venetoclax, see the pharmacy manual and the Investigator's Brochure. Section 4.3.1.3

4.3.1.2 Chlorambucil

For information on the formulation, packaging, and handling of chlorambucil, see the local prescribing information for chlorambucil.

4.3.1.3 Obinutuzumab

Obinutuzumab will be supplied centrally for all countries participating in the study as a single-dose, sterile liquid formulation in a 50-mL pharmaceutical grade glass vial containing a nominal 1000 mg obinutuzumab. The vial contains 40 mL of solution with 2.5% overfill. For information on the formulation, packaging, and handling of obinutuzumab, see the local prescribing information.

For a single dose, use a 250-mL infusion bag containing 0.9% sodium chloride and withdraw and discard 40 mL of the sodium chloride. Withdraw 40 mL of obinutuzumab from a single glass vial and inject into the infusion bag. Discard any unused portion of obinutuzumab left in the vial. Gently invert the infusion bag to mix the solution; do not shake.

Compatibility of the obinutuzumab with 0.9% sodium chloride has been tested in a concentration range from 0.2 mg/mL to 20 mg/mL. Dilutions of obinutuzumab in 0.9% sodium chloride have been found to be stable for 24 hours at 2°C–8°C and an additional 24 hours at ambient temperature and ambient room lighting. Storage conditions prior to

use are the responsibility of the user and would normally not be longer than 24 hours at 2°C–8°C.

Administration sets with polyvinyl chloride, polyurethane, or polyethylene as product contact surfaces and IV bags with polyolefin, polypropylene, polyvinyl chloride or polyurethane as product contact surfaces are compatible and may be used.

For further details, see the Obinutuzumab Investigator's Brochure.

4.3.2 Dosage, Administration, and Compliance

Venetoclax

Study patients will self-administer venetoclax tablets by mouth daily. Each dose of venetoclax will be taken with approximately 240 mL of water within 30 minutes after the patient's first meal of the day (e.g., breakfast).

On days that pre-dose PK sampling is required, the patient's first meal of the day (e.g., breakfast) will be provided in the morning at the clinic, and venetoclax dosing will occur in the clinic after completion of the meal to facilitate PK sampling.

On days when both venetoclax and obinutuzumab are given, the order of study treatment administration will be venetoclax followed by obinutuzumab (there is no minimum time required between the administration of venetoclax and the start of the obinutuzumab infusion). If vomiting occurs within 15 minutes of taking venetoclax and all expelled tablets are still intact, another dose may be given and the second dose noted in the drug log. Otherwise, no replacement dose is to be given. In cases where a dose of venetoclax is missed or forgotten, the patient should take the dose as soon as possible, ensuring that the dose is taken within 8 hours of the missed dose with food. Otherwise, the dose should not be taken. On days when patients are scheduled to have blood samples collected for PK assessments, the time of each dose of venetoclax will be recorded to the nearest minute. Venetoclax must be stored according to labeled storage conditions. There is to be no break between cycles.

All patients, including the open-label run-in phase and the randomized phase of the study, regardless of the assigned study arm, must receive prophylaxis for TLS prior to the initiation of study treatment (see Appendix 10) and after pre-infusion laboratory values are checked.

Obinutuzumab- First Infusion

Obinutuzumab will be administered by IV infusion as an absolute (flat) dose of 1000 mg. Obinutuzumab may be administered in a single day; however (as per local prescribing information), for the first administration, patients can receive their first dose of obinutuzumab over 2 consecutive days (split dose) in Cycle 1: 100 mg on Day 1 and 900 mg on Day 2. Two infusion bags should be prepared for the infusion on Days 1 and 2 (100 mg for Day 1 and 900 mg for Day 2). If the first bag is completed without

modifications of the infusion rate or interruptions, the second bag may be administered on the same day (no dose delay necessary, no repetition of premedication), provided that appropriate time, conditions, and medical supervision are available throughout the infusion. If there are any modifications of the infusion rate or interruptions during the first 100 mg, the second bag should be administered the following day. If patients require a delay of greater than 24 hours between the 100- and 900-mg infusions of obinutuzumab, see Section 4.3.1.3 and the Obinutuzumab Investigator's Brochure regarding drug stability.

From a microbiological perspective, the prepared infusion solution should be used immediately. If it is not used immediately, in-use storage times and conditions prior to use are the responsibility of the user and would normally not be longer than 24 hours at 2°C–8°C, unless dilution has taken place in controlled and validated aseptic conditions.

Obinutuzumab should be prepared by a healthcare professional using aseptic technique. Do not shake the vial.

Withdraw 40 mL of concentrate from the vial and dilute in polyvinyl chloride (PVC) or non-PVC polyolefin infusion bags containing sodium chloride 9 mg/mL (0.9%) solution for injection.

To ensure differentiation of the two infusion bags for the initial 1,000-mg dose, it is recommended to utilize bags of different sizes to distinguish between the 100-mg dose for Cycle 1, Day 1 and the 900-mg dose for Cycle 1, Day 1 (continued) or Cycle 1, Day 2. To prepare the two infusion bags, withdraw 40 mL of concentrate from the vial and dilute 4 mL into a 100-mL PVC or non-PVC polyolefin infusion bag and the remaining 36 mL in a 250-mL PVC or non-PVC polyolefin infusion bag containing sodium chloride 9 mg/mL (0.9%) solution for injection. Clearly label each infusion bag. For storage conditions of the infusion bags see Section 4.3.1.3.

Obinutuzumab must be administered in a clinical setting (inpatient or outpatient). Full emergency resuscitation facilities should be immediately available, and patients should be under close supervision of the investigator at all times. Obinutuzumab should be given as a slow IV infusion through a dedicated line. IV infusion pumps (such as Braun Infusomat Space) should be used to control the infusion rate of obinutuzumab. Do not administer as an IV push or bolus. After the end of the first infusion, the IV line should remain in place for at least 2 hours in order to be able to administer IV drugs if necessary. If no adverse events occur after 2 hours, the IV line may be removed. For subsequent infusions, the IV line should remain in place for at least 1 hour from the end of infusion, and if no adverse events occur after 1 hour, the IV line may be removed.

Hypotension may be expected with infusions; therefore, withholding of antihypertensive treatment should be considered for 12 hours prior to the obinutuzumab infusions,

throughout the obinutuzumab infusions, and for the first hour after obinutuzumab infusions.

The first infusion of obinutuzumab should be started only after administration of prophylactic medications to mitigate the risks of IRRs and TLS (see Appendix 11) and after pre-infusion laboratory values are checked.

The first obinutuzumab infusion will be administered at an initial rate of 25 mg over 4 hours. In the absence of IRRs/hypersensitivity, the rate of the infusion will be escalated in increments of 50 mg/hour every 30 minutes to a maximum rate of 400 mg/hour. If the start of infusion is delayed or adverse events occur that require delaying or stopping the infusion such that infusion in a single day is not feasible, the infusion may be split and completed on the next day.

During the initial infusion of obinutuzumab, vital signs will be obtained pre-infusion, then every 15 minutes for 90 minutes, then every 30 minutes until the end of infusion, and then every 60 minutes until the infusion line is removed. If obinutuzumab is well-tolerated without significant infusion-related symptoms, vital signs for subsequent infusions can be obtained every 30 minutes until the infusion line is removed.

Patients with pre-existing cardiac and/or pulmonary conditions or who have had a prior clinically significant cardiopulmonary adverse event with obinutuzumab should be monitored very carefully throughout the infusion and post-infusion period. Patients with prior clinically significant cardiac disease are excluded per eligibility criteria.

Prophylaxis for Infusion-Related Reactions

All obinutuzumab infusions should be administered 30–60 minutes after premedication with oral acetaminophen (e.g., 650–1000 mg) and an antihistamine such as diphenhydramine (50–100 mg).

For the first dose of obinutuzumab, premedication with corticosteroids (100 mg IV prednisolone or equivalent) is mandatory for all patients and must be administered at least 1 hour prior to the 100 mg dose on Cycle 1, Day 1 and the 900 mg dose on Cycle 1, Day 2. An equivalent dose of dexamethasone (20 mg) or methylprednisolone (80 mg) is permitted, but hydrocortisone should not be used. The required corticosteroid premedication may be modified by the investigator, in consultation with the sponsor, on the basis of observed safety from this study and review of data from other ongoing studies with obinutuzumab.

For subsequent infusions, corticosteroid premedication should be given to patients who experienced a Grade 3 IRR with the previous infusion, to patients with lymphocyte counts $> 25 \times 10^9$/L, and at the investigator's discretion.

For patients who do not experience Grade ≥3 infusion-related symptoms with their previous infusion (i.e., do not receive medication to treat the reaction symptoms and do not experience infusion interruption), premedication for subsequent infusions may be omitted at the investigator's discretion.

For patients with a high lymphocyte count or bulky lymphadenopathy, the infusion may be given extremely slowly over a longer period of time or the dose may be split and given over more than 1 day.

If a hypersensitivity or IRR develops, the infusion should be temporarily interrupted or slowed down and concomitant medication may be administered if deemed appropriate by the investigator. Investigators should consider administering the following medications/interventions in response to an IRR:

- Patients may be treated with acetaminophen/paracetamol and an antihistamine if the patient had not received them in the 4 hours prior to the start of the IRR.
- Patients may receive additional IV hydration.
- In the event of bronchospasm, urticaria, or dyspnea, patients may require antihistamines, oxygen, additional corticosteroids (e.g., 100 mg of IV prednisolone or equivalent; please note hydrocortisone should not be used to manage an IRR), and/or bronchodilators.
- In the event of hypotension, patients may require vasopressors.

Upon resolution of symptoms, the infusion will resume at half of the previous rate (the rate being used at the time that the hypersensitivity or IRR occurred), and infusion rate escalation may resume at the increments and intervals described above.

Obinutuzumab TLS Prophylaxis

- Prophylaxis of hyperuricemia and dehydration as well as laboratory monitoring are similar as described below for venetoclax dosing.
- Patients with a high tumor burden (absolute lymphocyte count [ALC] $\geq 25 \times 10^9$/L or bulky lymphadenopathy) must receive prophylaxis for TLS prior to the initiation of treatment. These patients must be well-hydrated. It is desirable to maintain a fluid intake of approximately 3 L/day, 1–2 days before the first dose of obinutuzumab. All such patients with a high tumor burden must be treated with allopurinol or a suitable alternative treatment starting 12-24 hours prior to the first infusion. Patients should continue to receive prophylaxis with allopurinol and adequate hydration prior to each subsequent infusion, if deemed appropriate by the investigator.
- If any laboratory abnormalities that are consistent with TLS are observed, patients should undergo further management and monitoring as per the Recommendations for Initial Management of Electrolyte Imbalances and Prevention of Tumor Lysis Syndrome (see Appendix 10).

- Hematology and chemistry samples will be obtained prior to obinutuzumab dosing, 24 hours after the initiation of the obinutuzumab infusion (Cycle 1 Day 3 if split dose is given; see Appendix 1 for details). Discharge of patient is dependent upon review of the 24-hour laboratory values by the investigator or designee.

Subsequent Infusions of Obinutuzumab

If the patient's previous infusion of obinutuzumab was well tolerated (defined by an absence of Grade 2 IRRs during a final infusion rate of $\geq$100 mg/hour), subsequent infusions will be administered at an initial rate of 100 mg/hour and increased by 100 mg/hour increments at 30-minute intervals, as tolerated, to a maximum rate of 400 mg/hour. If a hypersensitivity or IRR develops, the infusion should be temporarily interrupted or slowed down, and concomitant medication may be administered if deemed appropriate by the investigator. Investigators should consider administering the following medications/interventions in response to an IRR:

- Patients may be treated with acetaminophen/paracetamol and an antihistamine if the patient had not received them in the 4 hours prior to the start of the IRR.
- Patients may receive additional IV hydration.
- In the event of bronchospasm, urticaria, or dyspnea, patients may require antihistamines, oxygen, additional corticosteroids (e.g., 100 mg of IV prednisolone or equivalent; please note hydrocortisone should not be used to manage an IRR), and/or bronchodilators.
- In the event of hypotension, patients may require vasopressors.

Upon resolution of symptoms, the infusion will resume at half the previous rate (the rate being used at the time that the hypersensitivity or IRR occurred), and infusion rate escalation may resume at the increments and intervals described above. If the previous infusion rate was not well tolerated as defined above, instructions for the first infusion rate will be used.

On days when both venetoclax and obinutuzumab are given, the order of study treatment administration will be venetoclax followed by obinutuzumab (there is no minimum time required between the administration of venetoclax and the start of the obinutuzumab infusion).

Table 3 Administration of First Infusion of Obinutuzumab, if Split Over Two Days, and Subsequent Infusions of Obinutuzumab

First Infusion	Subsequent Infusions
• **On Day 1**: All patients will receive an infusion at a fixed dose of 100 mg obinutuzumab administered at a fixed rate of 25 mg/hr with no increase in infusion rate (total duration of the first infusion: 4 hours).<br>• If the patient experiences an IRR, follow the protocol (Section 4.3.2) regarding treatment but at the resolution of symptoms, restart at half the initial rate (12.5 mg/hr). Increase to 25 mg/hr after an hour but do not increase further.<br>• If the patient tolerates 100mg without dose modification and local label permits, the Investigator may continue to administer the remaining 900mg on the same day at the infusion rate described below.<br>• **On Day 2**: All patients will receive 900 mg starting at the rate of 50 mg/hr, and the rate of the infusion will be escalated in increments of 50 mg/hr every 30-minutes to a maximum rate of 400 mg/hr.<br>• If a hypersensitivity or IRR develops, the infusion should be temporarily interrupted or slowed down and concomitant medication may be administered if deemed appropriate by the investigator.<br>Investigators should consider administering the following medications/interventions in response to an IRR:<br>Patients may be treated with acetaminophen/paracetamol and an antihistamine if the patient had not received them in the 4 hours prior to the start of the IRR.<br>Patients may receive additional IV hydration.<br>In the event of bronchospasm, urticaria, or dyspnea, patients may require antihistamines, oxygen, additional corticosteroids (e.g., 100	• If the first infusion of obinutuzumab was well tolerated (defined by an absence of IRRs during a final infusion rate of ≥100 mg/hr), subsequent infusions will be administered at an initial rate of 100 mg/hr and increased by 100 mg/hr increments at 30-minute intervals, as tolerated, to a maximum rate of 400 mg/hr.<br>• If a hypersensitivity or IRR develops, the infusion should be temporarily interrupted or slowed down, and concomitant medication may be administered if deemed appropriate by the investigator.<br>Investigators should consider administering the following medications/interventions in response to an IRR:<br>Patients may be treated with acetaminophen/paracetamol and an antihistamine if the patient had not received them in the 4 hours prior to the start of the IRR.<br>Patients may receive additional IV hydration.<br>In the event of bronchospasm, urticaria, or dyspnea, patients may require antihistamines, oxygen, additional corticosteroids (e.g., 100 mg of IV prednisolone or equivalent; please note hydrocortisone should not be used to manage an IRR), and/or bronchodilators.<br>In the event of hypotension, patients may require vasopressors.<br>• Upon resolution of symptoms, the infusion will resume at half of the previous rate (the rate being used at the time that the hypersensitivity or IRR occurred), and infusion rate escalation may resume at the increments and intervals described above. If the previous infusion rate was not well

First Infusion	Subsequent Infusions
mg of IV prednisolone or equivalent; please note hydrocortisone should not be used to manage an IRR), and/or bronchodilators.<br>In the event of hypotension, patients may require vasopressors.<br>• Upon resolution of symptoms, the infusion will resume at half of the previous rate (the rate being used at the time that the hypersensitivity or IRR occurred), and infusion rate escalation may resume at the increments and intervals described above.	tolerated, as defined above, instructions for the first infusion rate will be used.

IRR = infusion-related reaction; IV = intravenous.

Guidelines for dosage modification and treatment interruption or discontinuation are provided in Section 4.6.

Any overdose or incorrect administration of study drug should be noted on the Study Drug Administration eCRF. Adverse Events associated with an overdose or incorrect administration of study drug should be recorded on the Adverse Event eCRF.

Chlorambucil

Chlorambucil will be administered to patients who are randomized to receive chlorambucil. Patients will receive 0.5mg/kg orally (tablets) on Days 1 and 15 for 12 cycles according to the Chlorambucil Summary of Product Characteristics (SmPC).

On days when both chlorambucil and obinutuzumab are given, the order of study treatment administration will be chlorambucil followed by obinutuzumab (there is no minimum time required between the administration of chlorambucil and the start of the obinutuzumab infusion).

If weight changes are observed by 10%, chlorambucil dosing must be adjusted accordingly.

4.3.3 Investigational Medicinal Products

All investigational medicinal products (IMPs) required for completion of this study (chlorambucil, obinutuzumab, and venetoclax) will be provided by the Sponsor where required by local health authority regulations. The study site will acknowledge receipt of IMPs and confirm the shipment condition and content. Any damaged shipments will be replaced.

IMPs will either be disposed of at the study site according to the study site's institutional standard operating procedure or returned to the Sponsor with the appropriate documentation. The site's method of IMP destruction must be agreed to by the Sponsor. The site must obtain written authorization from the Sponsor before any IMP is destroyed, and IMP destruction must be documented on the appropriate form.

Accurate records of all IMPs received at, dispensed from, returned to, and disposed of by the study site should be recorded on the Drug Inventory Log.

4.3.4 Non-Investigational Medicinal Products

Rasburicase

Rasburicase (Fasturtec™ in Europe; Elitek™ in the United States) enzymatically converts uric acid in the blood to allantoin and hydrogen peroxide, thereby preventing the formation of uric acid crystals and potential renal blockage. It reduces uric acid levels within 4 hours both in pediatric and adult patients, and several studies confirm its safety, effectiveness, and tolerability both in the prevention and treatment of TLS, although the prevention of acute renal failure fails in over 25% of patients.

Note: Rasburicase may be supplied by the Sponsor in countries where rasburicase is not approved or cannot be supplied locally.

4.3.5 Post-Trial Access to Venetoclax

Treatment switching from one arm to the other is not allowed, even after disease progression.

The Sponsor will offer post-trial access to venetoclax free of charge to eligible patients in accordance with the Roche Global Policy on Continued Access to Investigational Medicinal Product.

A patient will be eligible to receive study drug after the end of the study in case of withdrawal if all of the following conditions are met:

- The patient has a life-threatening or severe medical condition and requires continued study drug treatment for his or her well-being.
- There are no appropriate alternative treatments available to the patient.
- The patient and his/her physician comply with and satisfy any legal or regulatory requirements that apply to them.

A patient will not be eligible to receive study drug after the end of the study in the case of withdrawal if any of the following conditions are met:

- The study drug is commercially marketed in the patient's country and is reasonably accessible to the patient (e.g., is covered by the patient's insurance or would not otherwise create a financial hardship for the patient).
- Provision of study drug is not permitted under the laws and regulations of the patient's country.

The Roche Global Policy on Continued Access to Investigational Medicinal Product is available at http://www.roche.com/responsibility/business_ethics/ethical_standards.htm.

4.4 CONCOMITANT THERAPY

4.4.1 Permitted Therapy

Concomitant medication includes any medication (e.g., prescription drugs, over-the-counter drugs, herbal or homeopathic remedies, nutritional supplements) used by a patient from 30 days prior to the screening period. All concomitant medications should be reported to the investigator and recorded on the Concomitant Medications eCRF. Patients who use oral contraceptives, hormone-replacement therapy, or other maintenance therapy should continue their use.

4.4.2 Prohibited Therapy

Patients who require the use of any of the excluded therapies listed below will be discontinued from study treatment. Patients who are discontinued from study treatment will be followed for safety outcomes for 30 days following the patient’s last dose of

venetoclax or 90 days following the patient's last dose of obinutuzumab, whichever is later. All patients who discontinue study treatment for any reason should be followed until progression.

Use of the following therapies is prohibited during the study:

- Radiotherapy
- Immunotherapy
- Hormone therapy (other than contraceptives, hormone-replacement therapy, or megestrol acetate)
- Any therapies intended for the treatment of lymphoma/leukemia whether approved or experimental (outside of this study)

4.4.2.1 Drug–Drug Interaction

4.4.2.1.1 Patients Randomized to Arm A (Venetoclax + Obinutuzumab)

The following concomitant medications are not allowed within 7 days prior to the administration of the first dose of study drug and during the venetoclax dose ramp-up. These medications are cautionary once patients are receiving 400 mg per day of venetoclax:

- Strong and moderate CYP3A inhibitors (see Appendix 5 for examples)

  Furthermore, patients who require these medications while they are receiving 400 mg per day of venetoclax should reduce the venetoclax dose by 2-fold for moderate inhibitors and 4-fold for strong inhibitors during co-administration. See Table 4 for additional details.

- Strong and moderate CYP3A inducers (see Appendix 5 for examples)

If clinically indicated, anti-herpes and anti-pneumocystis prophylaxis should be considered. Although there is a potential for drug-drug interactions, there are likely to be limited potential clinical effects; therefore trimethoprim-sulfamethoxazole can be considered for pneumocystis prophylaxis, with close clinical monitoring.

Study M15-065 demonstrated that following co-administration of single doses of 400-mg venetoclax with 5-mg warfarin, maximum plasma concentration observed, and area under the concentration–time curve from Time 0 to infinity of R- and S-warfarin increased by approximately 18%–28%. Based on these data, warfarin has been moved from the excluded medication list to the cautionary medication list. Warfarin may be co-administered with venetoclax with caution and with the guidance of the Medical Monitor (additional international normalized ratio [INR] monitoring may also be required). Figure 2 describes the plasma concentrations of S-warfarin in patients treated with venetoclax and warfarin versus those treated with warfarin alone. Based on these data, warfarin has been moved to the cautionary list of concomitant medication.

Figure 2 Difference in Plasma S-Warfarin Concentration Following Treatment with Warfarin + Venetoclax versus Warfarin Alone

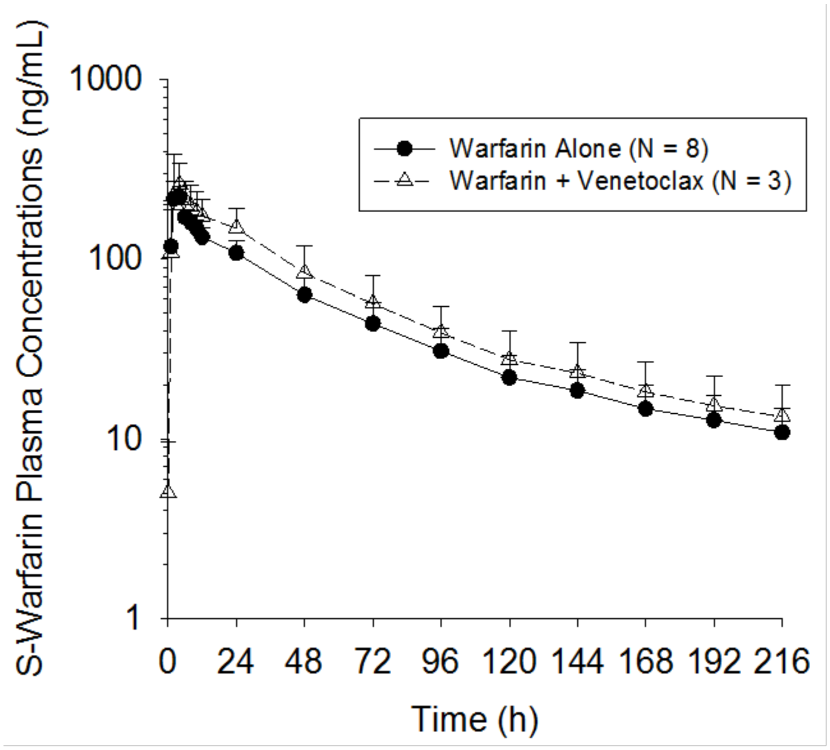

Results expressed as mean + standard deviation of S-warfarin plasma concentration over time following administration of warfarin + venetoclax and warfarin alone using a log-linear scale.

Table 4 lists categories of excluded and cautionary medications for patients randomized to Arm A (venetoclax + obinutuzumab). See Appendix 5 for a sample list of excluded and cautionary medications for patients randomized to Arm A (venetoclax + obinutuzumab).

Table 4 Excluded and Cautionary Medications for Patients Randomized to Arm A (Venetoclax+Obinutuzumab)

Excluded
Anticancer therapies, including chemotherapy, radiotherapy, or other investigational therapy (which includes targeted small molecule agents):<br>Excluded 5 half-lives prior to first dose and throughout venetoclax administration.<br>Biologic agents (e.g., monoclonal antibodies) for anti-neoplastic intent:<br>Excluded 8 weeks prior to first dose of study drug.<br>Grapefruit, grapefruit products, Seville oranges (including marmalade containing Seville oranges) or starfruit:<br>Excluded 3 days prior to first dose and throughout venetoclax administration.
Excluded during the venetoclax ramp-up period and cautionary thereafter:
Strong and moderate CYP3A inhibitors:<br>Excluded during the venetoclax ramp-up period; consider alternative medications.<br>If a patient requires use of these medications while they are receiving 400 mg per day of venetoclax, use with caution and reduce the venetoclax dose by 2-fold for moderate inhibitors and 4-fold for strong inhibitors during co-administration. After discontinuation of CYP3A inhibitor, wait for 3 days before venetoclax dose is increased back to the target dose.<br>Strong and moderate CYP3A inducers:<br>Excluded during the venetoclax ramp-up period; consider alternative medications.<br>If a patient requires use of these medications while they are receiving 400 mg per day of venetoclax, use with caution and contact the Medical Monitor for guidance.
Cautionary
Warfarin<br>Weak CYP3A inducers<br>Weak CYP3A inhibitors<br>P-gp substrates<br>BCRP substrates<br>OATP1B1/1B3 substrates<br>P-gp inhibitors<br>BCRP inhibitors<br>OATP1B1/B3 inhibitors

Note: See Appendix 5 for examples of these medications.

A sample list of cautionary and excluded medications that fall into the categories within this section can be found in Appendix 5 . It is not possible to produce a 100% exhaustive list of medications that fall into these categories; therefore, if in question, refer to the appropriate product label.

4.4.3 Other Prohibited Medication

Steroid therapy for anti-neoplastic intent with the exception of inhaled steroids for asthma, topical steroids, or replacement/stress corticosteroids are not permitted during the study at any time.

4.4.4 Prohibited Food

Use of the following foods by patients randomized to Arm A is prohibited for at least 3 days prior to initiation of venetoclax treatment and throughout venetoclax administration.

Constituents of these foods have been shown to inhibit CYP3A4, the major enzyme responsible for the metabolism of venetoclax. Consumption of these foods could lead to increased venetoclax exposure:

- Grapefruit
- Grapefruit products
- Seville oranges (including marmalade containing Seville oranges)
- Star fruit

4.5 STUDY ASSESSMENTS

Appendix 2 and Appendix 3 outline the schedule of assessments performed during the study.

4.5.1 Informed Consent Forms and Screening Log

Written informed consent for participation in the study must be obtained before performing any study-specific screening tests or evaluations. ICFs, including biobanking ICFs, for enrolled patients and for patients who are not subsequently enrolled will be maintained at the study site.

All screening evaluations must be completed and reviewed to confirm that patients meet all eligibility criteria before randomization. The investigator will maintain a screening log to record details of all patients screened and to confirm eligibility or record reasons for screening failure, as applicable.

4.5.2 Procedures for Enrollment of Eligible Subjects

A medical review of selected screening data will be performed by the GCLLSG to confirm eligibility prior to randomization.

A blood sample will be sent for central immunophenotyping for patients entered at investigational sites that can ship the samples to reach the laboratory in Cologne, Germany. The results must be available and reviewed by the GCLLSG physician prior to randomization. Exceptionally, confirmation of CLL and CD20 expression by immunophenotyping will be performed locally at the investigational site, but the results must be reviewed by the GCLLSG physician prior to randomization. A copy of the

original diagnostic report (e.g., FACS plots) will be sent to the sponsor and stored for possible future validation by an independent reviewer if required.

All patients in the randomized part of the study must commence treatment within 7 days of randomization.

For patients who experience a Grade 3 or 4 adverse event between randomization and the start of study drug, the start of the treatment may be delayed for up to 28 days. If the Grade 3 or 4 event does not improve within 28 days, the patient will be withdrawn from the study.

The investigator or designee will use the eCRF and assign a new patient number and enter the corresponding number for allocation to the treatment groups in the appropriate place on each patient's eCRF. Patient numbers will be chronologically assigned within each center as patients are enrolled into the study. Each patient number within the study will be unique.

A Subject Enrollment and Identification Code List must be maintained by the investigator.

4.5.3 Medical History and Demographic Data

Medical history includes clinically significant diseases, surgeries, cancer history (including prior cancer therapies and procedures), reproductive status, smoking history, use of alcohol and drugs of abuse, and all medications (e.g., prescription drugs, over-the-counter drugs, herbal or homeopathic remedies, nutritional supplements) used by the patient within 28 days prior to the screening visit. At this time the patient's CIRS score should be assessed to determine eligibility for entry into the study (note that symptoms related to CLL should not be included in the patient's screening CIRS score). The Instrumental Activities of Daily Life (IADL) should also be assessed by the physician (see Appendix 21). Demographic data will include age, sex and self-reported race/ethnicity.

4.5.4 Physical Examinations

A complete physical examination should include an evaluation of the head, eyes, ears, nose, and throat and the cardiovascular, dermatological, musculoskeletal, respiratory, gastrointestinal, genitourinary, and neurological systems. Any abnormality identified at baseline should be recorded on the General Medical History and Baseline Conditions eCRF.

At subsequent visits (or as clinically indicated), limited, symptom-directed physical examinations should be performed. Changes from baseline abnormalities should be recorded in patient notes. New or worsened clinically significant abnormalities should be recorded as adverse events on the Adverse Event eCRF.

4.5.5 Performance Status

Performance status (PS) will be measured using the Eastern Cooperative Oncology Group (ECOG) Performance Status Scale (see Appendix 16). It is recommended, where possible, that a patient's PS be assessed by the same person throughout the study.

4.5.6 Clinical Staging

At baseline, patients should be assessed according to Binet Staging criteria. Staging is based on the number of involved areas, as defined by the presence of enlarged lymph nodes of >1 cm in diameter (by physical examination only) or organomegaly, and on whether anemia or thrombocytopenia are present.

Areas of involvement to be assessed for staging:

- Head and neck, including the Waldeyer's ring (this counts as one area, even if more than one group of nodes is enlarged)
- Axillae (involvement of both axillae is counted as one area)
- Groins, including superficial femorals (involvement of both groins is counted as one area)
- Palpable spleen
- Palpable liver (clinically enlarged)

Binet Stage A:

Hemoglobin ≥ 100 g/L (10 g/dL) and platelets $\geq 100 \times 10^9$/L and up to 2 areas involved

Binet Stage B:

Hemoglobin ≥ 100 g/L (10 g/dL) and platelets $\geq 100 \times 10^9$/L and 3–5 areas involved

Binet Stage C:

Hemoglobin < 100 g/L (10 g/dL) and/or platelets $< 100 \times 10^9$/L and irrespective of nodal or organ enlargement

4.5.7 Criteria for Initiation of First-Line Treatment

At the time of screening, the patient must satisfy the National Cancer Institute (NCI) criteria for active disease that requires treatment:

- Evidence of progressive marrow failure as manifested by the development or worsening of anemia and/or thrombocytopenia
- Massive (i.e., ≥ 6 cm below the left costal margin) or progressive or symptomatic splenomegaly
- Massive nodes (i.e., ≥ 10 cm in the longest diameter) or progressive or symptomatic lymphadenopathy

- Progressive lymphocytosis with an increase of more than 50% over a 2-month period or lymphocyte doubling time (LDT) of less than 6 months. LDT can be obtained at intervals of 2 weeks over an observation period of 2–3 months. In patients with initial blood lymphocyte counts of $< 30 \times 10^9$/L, LDT should not be used as a single parameter to define a treatment indication. In addition, factors contributing to lymphocytosis or lymphadenopathy other than CLL (e.g., infections) should be excluded.
- Autoimmune anemia and/or thrombocytopenia that is poorly responsive to corticosteroids or other standard therapy
- Constitutional symptoms, defined as any one of more of the following disease-related symptoms or signs:

    Unintentional weight loss of $\geq$10% within the previous 6 months

    Significant fatigue (ECOG PS 2 or worse; inability to work or perform usual activities)

    Fevers $>$100.5°F or 38.0°C for $\geq$2 weeks without other evidence of infection

    Night sweats for $>$1 month without evidence of infection

4.5.8 Vital Signs

Vital signs will include measurements of pulse rate, temperature, and systolic and diastolic blood pressures while the patient is in a seated position.

4.5.9 Tumor and Response Evaluations

Response will be assessed according to the IWCLL guidelines (see Appendix 12).

The investigator assessment of response and progression, on the basis of peripheral blood counts, physical examination, bone marrow aspirate and biopsy results, and CT scans (when indicated), is the basis for individual patient treatment decisions and is considered primary for all endpoints in the study. For purposes of regulatory decision-making in the United States, PFS by IRC will be considered primary.

The primary objective of the early response assessment at Cycle 4, Day1 and Cycle 7, Day 1 is to identify disease progression. At these visits, response to treatment (CR/PR) should be determined according to the assessments planned for that visit: physical examination and laboratory tests. With the availability of imaging and bone marrow examination data at the $+3$ month follow up visit, the end of treatment response assessment can then be fully assessed according to the IWCLL Guidelines (2008; see Appendix 12).

CT imaging will not be used to determine PD because its value is questionable in a leukemia setting. Only when PD is detected by physical examination in the absence of any objective hematological progression will a CT scan of the involved nodes be performed.

4.5.10 Tumor Response

An independent review committee will review efficacy data for disease progression (see Section 9.4.3 for more details).

Baseline tumor assessments must be assessed a maximum of 4 weeks before randomization. Given that response is a secondary endpoint of the trial and that PD will be followed by physical examination, CT scans performed prior to screening as part of the regular clinical work-up of the patient will be allowed up to 8 weeks before randomization. This is to prevent the re-irradiation of patients who have recently undergone a CT scan. However, a CT scan must be performed within the 4-week period prior to randomization for those patients with signs of rapidly progressing disease at screening.

All patients should be continuously monitored for PD during the treatment period. At any time, if progression is confirmed, it should be clearly documented in the eCRF. Interim staging assessments will be performed at Cycle 4, Day 1, Cycle 7 Day 1, and Cycle 9 Day 1. In addition to an assessment of hematological status, this will also include a full physical examination to assess any lymphadenopathy and hepato/splenomegaly.

Unless disease progression is confirmed, the patient is withdrawn due to toxicity, or the patient has died, patients should receive a maximum of 6 cycles of obinutuzumab and 12 cycles of venetoclax/chlorambucil (28 daily venetoclax doses and 2 chlorambucil doses at Days 1 and 15). If there is suspicion of PD given clinical or laboratory findings before the next scheduled assessment, an unscheduled assessment should be performed.

The end of treatment response assessment must be performed 3 months after the last study treatment (no earlier than 2 months after the end of study treatment). For those patients with a CR and PR confirmed by laboratory and physical examination and CT scan, a bone marrow aspirate and biopsy must also be performed. The bone marrow aspirate sample will also be tested for MRD status by ASO-PCR.

All other follow-up tumor assessments will be done within $\pm$14 days for 3-monthly and within a month for 6-monthly assessments of the scheduled visits described in Appendix 2 and Appendix 3.

If a patient inadvertently misses a prescribed tumor evaluation or a technical error prevents the evaluation, the patient may continue treatment until the next scheduled assessment unless signs of clinical progression are present.

4.5.11 Response Criteria

Complete Response

CR requires all of the following criteria as assessed no earlier than 2 months after completion of therapy:

- Peripheral blood lymphocytes (evaluated by blood and differential count) below 4×10^9 /L (4,000/μL)
- Absence of significant lymphadenopathy (nodes $\leq$15 mm in longest diameter or any extra nodal disease) by physical examination and CT scan
- No hepatomegaly as determined by measurement below costal margin. Hepatomegaly considered to be due to CLL disease is defined as $>$3 cm below costal margin.
- No splenomegaly by physical examination as determined by measurement below the costal margin. A palpable spleen of any size is considered to be related to CLL disease.
- Absence of disease or constitutional symptoms (B symptoms)
- Blood counts above the following values:

  Neutrophils $> 1.5 \times 10^9$/L [1500/μL] (without growth factors)

  Platelets $> 100 \times 10^9$/L [100,000/μL] (without platelet transfusion or growth factors)

  Hemoglobin $>$110 g/L [11 g/dL] (without blood transfusions or erythropoietin).
- Bone marrow at least normocellular for age, $<$30% of nucleated cells being lymphocytes. Lymphoid nodules should be absent.

  Bone marrow aspirate and biopsy should be performed 3 months after the last treatment when clinical and laboratory results listed above demonstrate that a CR/cytopenic CR has been achieved. If the bone marrow is hypocellular, a repeat determination should be made in 4 weeks or when peripheral blood counts have recovered. However, this time interval should not exceed 6 months. A marrow biopsy result should be compared to a pre-treatment marrow result if available. Patients who are otherwise in a complete remission but have bone marrow nodules that can be identified histologically should be considered to have a PR. Immunohistochemistry should be performed to define whether these nodules are composed of primarily T cells, lymphocytes other than CLL cells, or CLL cells.

Complete Response and Incomplete Bone Marrow Recovery

For patients who fulfil the criteria for CR (including bone marrow) but who have persistent cytopenia, anemia, thrombocytopenia, or neutropenia, the marrow evaluation described above should be performed with scrutiny and should not show any clonal infiltrate.

Partial Response

To be considered to have a PR, patients must exhibit the following features for at least 2 months from the end of treatment:

- $\geq$50% decrease in peripheral blood lymphocyte count from the pre-treatment value

AND either

- $\geq$50% reduction in lymphadenopathy (sum of longest diameter of up to six largest lymph nodes by physical exam and 50% reduction in the sum of product of the diameter of up to six of the largest lymph nodes measured by CT scan). No increase in any node and no new enlarged lymph node. In small lymph nodes ($<$2 cm in diameter), an increase of less than 25% is not considered to be significant.

OR

- $\geq$50% reduction of liver enlargement, if enlarged at baseline, as assessed by physical examination

OR

- $\geq$50% reduction of spleen enlargement, if enlarged at baseline, as assessed by physical examination

AND at least one of the following

- Neutrophils $> 1.5 \times 10^9$/L (1500/μL) (without growth factors) or $\geq$50% increase of pretreatment value
- Platelets $> 100 \times 10^9$/L (100,000/μL) (without platelet transfusion or growth factors) or $\geq$50% increase of pretreatment value
- Hemoglobin $>$110g/L (11 g/dL) (without blood transfusions or erythropoietin) or $\geq$50% increase of pretreatment value

Progressive Disease

Progressive disease during or after therapy will be characterized by at least one of the following:

- Increase of $\geq$50% in the absolute number of circulating lymphocytes, to at least 5×10^9/L. During combination treatment and up to Cycle 9, Day 1 (or 3 months after last IV infusion), the increase should be assessed against baseline using a Day 1 (pre-cycle) nadir lymphocyte count and not interim cycle lymphocyte counts, which may not be stable. During monotherapy treatment and during follow up, increases should be assessed against the assessment at Cycle 9, Day 1 (or 3 months after last IV infusion).
- Appearance of new palpable lymph nodes ($>$15 mm in longest diameter) or any new extra nodal lesion (regardless of size)

- Increase of ≥50% in the longest diameter of any previous site of clinically significant lymphadenopathy (i.e., any lesion >10 mm at baseline). During combination treatment and up to Cycle 9, Day 1 (or 3 months after last IV infusion), the increase should be assessed against baseline. During monotherapy treatment and during follow up, increases should be assessed against the assessment at Cycle 9, Day 1 (or 3 months after last IV infusion).
- Increase of ≥50% in the enlargement of the liver and/or spleen as determined by measurement below the relevant costal margin or appearance of palpable hepatomegaly or splenomegaly that was not previously present. During combination treatment and up to Cycle 9, Day 1 (or 3 months after last IV infusion), the increase should be assessed against baseline. During monotherapy treatment and during follow up, increases should be assessed against the assessment at Cycle 9, Day 1 (or 3 months after last IV infusion).
- Transformation to a more aggressive histology (e.g., Richter's syndrome or pro-lymphocytic leukemia with >55% prolymphocytes). Whenever possible, this diagnosis should be supported by lymph node biopsy.
- After treatment, the progression of any cytopenia (unrelated to autoimmune cytopenia) as documented by:

  A decrease of hemoglobin levels by more than 20 g/L (2 g/dL) or to <100 g/L (10 g/dL), by a decrease of platelet counts by >50% or to $<100 \times 10^9$/L (100,000/μL), or by a decrease of neutrophil counts by >50% or to $<1 \times 10^9$/L that occurs no earlier than 3 months after end of therapy defines progression if the marrow biopsy demonstrates an infiltrate of clonal CLL cells.

Stable Disease

Patients who have not achieved a CR or a PR, or who have not exhibited PD, will be considered to have stable disease. Patients without CT evaluation who would otherwise meet the criteria for CR or PR will also be considered to have stable disease.

4.5.12 MRD Response Criteria

For the evaluation of MRD as part of the secondary endpoints of this study, MRD response rate will be determined as the proportion of patients with MRD-negativity measured in the peripheral blood at the completion of treatment assessment. Assessment of MRD in bone marrow is also required in patients with CR or PR and will be used to determine MRD response rate in bone marrow at the completion of treatment.

MRD response rates in the peripheral blood at completion of combination treatment assessment (Cycle 9, Day 1 or 3 months after last IV infusion) will also be determined. As well as evaluating MRD response rate in peripheral blood and bone marrow, it will further be evaluated using results of both measures, where a patient will be considered MRD positive if either the blood or bone marrow result is positive or both are missing. MRD response rates at other timepoints during treatment and follow-up where MRD is measured in the peripheral blood (see time and events schedule) will also be

summarized for ASO-PCR. Additional details on MRD methodology by ASO-PCR will be specified separately in an MRD Charter document.

Additional Exploratory Assessments of MRD Response

MRD response rate will be evaluated at each timepoint using flow cytometry and by Next-Generation Sequencing using the cutoff of $< 10^{-4}$ and also at the limit of sensitivity of these methodologies.

4.5.13 Other Disease-Specific Assessments

4.5.13.1 Complete Blood Count

Details on the required hematology panel necessary to assess response are provided in Section 3.3.7.

4.5.13.2 Bone Marrow

A bone marrow aspirate and biopsy are generally not requested at study entry. Nevertheless, CLL is a disease of the bone marrow, and it may be appropriate to evaluate a major site of involvement. In such cases, the aspirate smear must show ≥30% of all nucleated cells to be lymphoid. The percentage of atypical cells (e.g., pro-lymphocytes) should be ≤55%.

Bone marrow aspirate and biopsy should be performed at least 3 months after the last treatment when clinical and laboratory results listed above demonstrate that a CR/CRi has been achieved. If the bone marrow is hypocellular, a repeat determination should be made in 4 weeks or when peripheral blood counts have recovered. However, this time interval should not exceed 6 months. A marrow biopsy should be compared to a pre-treatment marrow if available. Patients who are otherwise in a complete remission but bone marrow nodules can be identified histologically should be considered to have PR. Immunohistochemistry should be performed to define whether these nodules are composed of primarily T cells, lymphocytes other than CLL cells, or CLL cells.

4.5.13.3 Lymphadenopathy and Hepatomegaly/Splenomegaly

A full physical examination should be performed to assess the extent of disease involvement. Up to a maximum of the six largest palpable lymph nodes, hepatomegaly, and splenomegaly should be recorded in the eCRF.

A CT scan (thorax, neck, abdomen, pelvis) will be performed at screening. Up to a maximum of the six largest bi-dimensional lesions should be reported in the eCRF. Note that these may be different from those lesions assessed by physical examination. A repeat CT scan of involved sites at baseline will be performed in patients who satisfy the clinical criteria for PR or CR after the end of treatment. When PD is detected by physical examination in the absence of any objective hematological progression, a CT scan of the involved nodes will be performed. In addition, CT scans may be performed at any time at the investigator's discretion or if clinically indicated. In patients with severe renal insufficiency at screening, CT contrast should be used according to local practice. If

necessary, magnetic resonance imaging (MRI) scans can be performed. CT without contrast is not recommended except for patients that develop renal insufficiency during treatment and have previously been assessed by CT scan. It is obligatory to use the same imaging technique for all tumor evaluations.

4.5.13.4 B-Symptoms

B-symptoms are considered to be constitutional symptoms defined as any one or more of the following disease-related symptoms or signs:

- Unintentional weight loss of 10% or more within the previous 6 months
- Significant fatigue (i.e., ECOG PS 2 or worse; inability to work or perform usual activities)
- Fevers $>$100.5°F or 38.0°C for $\geq$2 weeks without other evidence of infection
- Night sweats for $>$1 month without evidence of infection

B-symptoms should not be reported as adverse events. Worsening is generally considered a symptom (but not an objective criterion) of progression.

4.5.14 <u>Laboratory, Biomarker, and Other Biological Assessments (Local and Central)</u>

The following laboratory tests will be performed at the study site's local laboratory for analysis.

During Screening Only (Days –28 to –1)

- Lymphocyte count and immunophenotyping (this is to be done centrally, but in exceptional circumstances [e.g., sample could not be shipped in time to the central laboratory], it can be done locally)
- Assessment of major site involvement using bone marrow aspirate or biopsy at baseline
- Hematology (hemoglobin, WBC with differentials, platelets)
- Coagulation (aPTT, PT, INR)
- Biochemistry (biochemistry and electrolytes [including lactate dehydrogenase, sodium, potassium, calcium, phosphorus, chloride, bicarbonate, blood urea nitrogen, uric acid, aspartate aminotransferase, alanine aminotransferase, total bilirubin, serum creatinine, alkaline phosphatase, total protein, albumin])
- Coombs Test (Indirect and direct Coombs test should be repeated if positive)
- CrCl (should be estimated using the standard Cockcroft-Gault formula for calculated CrCl. A 24-hour urine collection may be undertaken to determine CrCl in patients with a borderline value for eligibility).
- Serum pregnancy test (in women of childbearing potential only); if serum pregnancy test has not been performed 14 days prior to dosing, a urine pregnancy test must be performed 7 days prior to dosing. If the test result is positive, patient dosing will be postponed until the patient’s status is confirmed by a serum pregnancy test.

- Immunoglobulins (Total IgA, IgG, IgM)
- A dipstick urinalysis for proteinuria will be performed at screening and follow-up for all patients.
- Hepatitis B (HBsAg) status in serum and HBcAb status in serum (using the same sample) for patients who were HBsAg-negative
- In HBcAb-positive patients, hepatitis B viral DNA (HBV DNA) and HCV RNA will be assessed.

Further Assessments

- Patients who are HBsAg-negative/HBcAb-positive with undetectable serum HBV DNA should be monitored closely (every month) for HBV DNA by a real-time PCR quantification assay (lower limit of detection: approx. 10 IU/mL) until at least 12 months after the last treatment cycle. If the HBV DNA assay becomes positive, patients should pre-emptively be treated with a nucleoside analog (i.e., lamivudine) for at least 12 months after the last cycle of therapy or be referred to a specialist, such as a gastroenterologist for management. This may not be relevant in all participating countries.
- Bone Marrow (optional at screening): If a bone marrow aspirate and/or biopsy is performed as part of the routine clinical work-up for the patient, this information should be collected in the eCRF. A bone marrow aspirate or biopsy is recommended in patients with an absolute neutrophil count (ANC) $< 1.5 \times 10^9$/L or platelets $< 75 \times 10^9$/L to demonstrate bone marrow infiltration by CLL as the cause of cytopenia or may be taken during the study to elucidate the cause of cytopenia.

Samples for the following laboratory tests will be sent to one or several central laboratories for analysis as per instructions in the laboratory manual:

MRD by ASO-PCR

- 10-mL ethylenediaminetetraacetic acid (EDTA) blood sample in all patients during screening (prior to randomization) and at repeated timepoints during treatment and follow-up (see Appendix 2 and Appendix 3). A patient will not be randomized until a viable sample has been collected and received at the testing laboratory. If a sample is found not to be viable, a further sample will be requested.
- A 4-mL EDTA bone marrow aspirate at completion of combination therapy (Cycle 9, Day 1 or 3 months after last IV infusion) and at the completion of treatment assessment (a minimum of 3 months after last treatment) for patients with CR/CRi and PR and will be assessed by ASO-PCR

MRD by Next Generation Sequencing

- 6-mL EDTA blood sample during screening/baseline and 10 mL at repeated timepoints (as per the schedule of assessments (see Appendix 2 and Appendix 3)

MRD by flow cytometry:

- 5-mL sodium heparin blood sample during screening/baseline and 10 mL at repeated timepoints (as per the Schedule of Assessments [see Appendix 2 and Appendix 3])

Lymphocyte immunophenotyping includes confirmation of diagnosis, IRR prediction, assessment of ZAP70 and CD38, and safety monitoring.

- 4-mL EDTA blood for confirmation of diagnosis by flow cytometry will be taken during screening. Analysis will include but is not limited to the following markers: CD5, CD16, CD19, CD20, CD23, light chain κ/λ restriction, ZAP-70, and CD38 expression.
- CD20 mean fluorescence intensity (MFI) on $CD19^{+}$ B cells and CD16 MFI on NK cells and monocytes (FcgR3A) and neutrophils (FcgR3B) will be captured to further assess potential predictors for IRR.
- If the central laboratory is not able to obtain a result, local immunophenotyping can be undertaken.

Safety monitoring/B-cell recovery:

- Safety Monitoring will be performed during screening and at subsequent visits during follow up. At screening, the sample for lymphocyte immunophenotyping will be used.
- 4-mL EDTA peripheral blood samples for $CD19^{+}/CD5^{+}$ and $C19^{+}/CD5^{-}$ B, as well as $CD14^{+}$ monocyte detection, will be collected from all patients at screening (lymphocyte immunophenotyping sample) and subsequent visits during treatment and follow up (see Appendix 2 and Appendix 3)
- Additional immunophenotyping samples may be collected if required for safety reasons at the discretion of the physician

Metaphase cytogenetics:

- A 10-mL sodium heparin peripheral blood sample will be taken at baseline to characterize chromosomal translocations.

Serum parameters (thymidine kinase, β2 microglobulin)

- A 10-mL whole blood sample (5-mL serum) will be taken at baseline for the testing of serum parameters (including but not limited to thymidine kinase and β2 microglobulin

CD40L stimulation, telomere length and targeted Next-Generation Sequencing

- A 10-mL EDTA peripheral blood sample will be taken <u>for additional research</u> at baseline and at time of relapse/PD. Analyses will include the assessment of cellular key pathways in CLL (e.g., pathway analyses after CD40L stimulation, assessment of telomere length, mutation analysis of genes associated with B-cell receptor signaling, and genes encoding for new treatment targets including BCL2

Genetic testing, TP53, and IGHV analysis:

- Two 10-mL sodium heparin peripheral blood samples will be taken at screening and at time of relapse/PD for the analysis of cytogenetic aberrations by FISH (-13q, -11q, -17p, trisomy [12]), TP53 mutations, and somatic hypermutation by analysis of IGHV mutational status. Targeted analysis of tumor-specific (non-inherited) genetic aberrations by next-generation sequencing of genes, including commonly mutated genes in CLL (including but not limited to ATM, TP53, NOTCH1, SF3B1, BIRC3). Any remaining samples will be stored in the GCLLSG Biobank if additional consent is given (see Section 4.5.17.1).

CLL cell count for MRD and Bcl-2 Family Analysis (IHC and Flow Cytometry):

- A 4-mL sodium heparin blood sample will be taken from all patients at screening (prior to randomization) for the quantification of CLL cells (required for MRD analysis by ASO-PCR) and for the assessment of Bcl-2 family by flow cytometry. A patient will not be randomized until a viable sample has been collected and received at the testing laboratory; if a sample is found not to be viable, a further sample will be requested.
- A 4-mL sodium heparin blood sample will be taken from all patients at time of relapse/PD for the assessment of Bcl-2 family by flow cytometry.
- A 6-mL sodium citrate blood sample will be taken from all patients at baseline and at time of relapse/PD for DNA and RNA isolation for evaluation of gene expression and mutation status of somatic genetic aberrations in Bcl-2 family genes (i.e., genes regulating apoptosis as well as other CLL disease related markers).
- A formalin-fixed paraffin embedded tissue (FFPE) block or 15 unstained, freshly cut FFPE tissue slides from lymph node biopsies that were collected as part of patient diagnosis, where available, shall be submitted for analysis of markers, including but not limited to BCL2, BCL-XL, and MCL1 by immunohistochemistry.

PK Measurements (in all patients of Treatment A; also see Appendix 4)

- A 3-mL blood sample will be collected into an EDTA tube at each PK sampling point (Appendix 4) for venetoclax.
- A 6-mL blood sample will be collected into a plain tube at each PK sampling point (Appendix 4) for obinutuzumab.

Pregnancy Test: All women who are not postmenopausal (≥12 months of non-therapy-induced amenorrhea) or surgically sterile will have a serum pregnancy test performed monthly while receiving study drug and at the Treatment Completion/Early Termination visit.

Note: sample shipment for central laboratory assessments must be made to designated laboratories as per instructions in the laboratory manual.

Total blood loss during screening is approximately 40 mL (3 tablespoons). Total blood loss at baseline is approximately 100 mL (7 tablespoons). Total blood loss during the

study is approximately 1.2 L (80 tablespoons) but varies slightly, depending upon TLS risk assessment. All samples will be destroyed at the end of analysis or at the end of the study, unless otherwise specified (see Section 3.1.2.4).

Upon optional consent, residual samples will be taken for storage in the GCLLSG Biobanking Repository (Section 4.5.17).

4.5.15 Electrocardiograms

Single ECG recordings will be obtained as outlined in the Schedule of Assessments (Appendix 2 and Appendix 3) and may be obtained at unscheduled timepoints as indicated.

All ECG recordings must be performed using a standard high-quality, high-fidelity digital electrocardiograph machine equipped with computer-based interval measurements. Lead placement should be as consistent as possible. ECG recordings must be performed after the patient has been resting in a supine position for at least 10 minutes. All ECGs are to be obtained prior to other procedures scheduled at that same time (e.g., vital sign measurements, blood draws) and should not be obtained within 3 hours after any meal. Circumstances that may induce changes in heart rate, including environmental distractions (e.g., television, radio, conversation), should be avoided during the pre-ECG resting period and during ECG recording.

For safety monitoring purposes, the investigator must review, sign, and date all ECG tracings. Paper copies of ECG tracings will be kept as part of the patient's permanent study file at the site. Digital recordings will be stored at site. The following should be recorded in the appropriate eCRF: heart rate, RR interval, QRS interval, PR duration, uncorrected QT interval, and corrected (Fridericia’s correction) QTc interval (QTcF) on the basis of the machine readings of the individual ECG tracings. Any morphologic waveform changes or other ECG abnormalities must be documented on the eCRF. If considered appropriate by the Sponsor, ECGs may be analyzed retrospectively at a central laboratory.

If, at a particular post-dose timepoint, the mean QTcF is $>$500 ms and/or $>$60 ms longer than the baseline value, another ECG must be recorded, ideally within the next 5 minutes, and ECG monitoring should continue until QTcF has stabilized on two successive ECGs. The Medical Monitor should be notified. Standard-of-care treatment may be instituted per the discretion of the investigator. A decision on study drug discontinuation should be made (Section 4.6). The investigator should also evaluate the patient for potential concurrent risk factors (e.g., electrolyte abnormalities, co-medications known to prolong the QT interval, severe bradycardia).

4.5.16 Patient-Reported and Clinician-Reported Outcomes

PRO data will be elicited from the patients in this study to more fully characterize the clinical profile of the combination of obinutuzumab and venetoclax compared with

obinutuzumab and chlorambucil in patients with previously untreated CLL and coexisting medical conditions. The PRO instruments, translated as required into the local language, will be distributed by the investigator staff and completed in their entirety by the patient at specified timepoints during the study. To ensure instrument validity and that data standards meet health authority requirements, PRO questionnaires should be self-administered at the investigational site prior to the completion of other study assessments and the administration of study treatment.

The MDASI-CLL (see Appendix 18) is a cancer-related multi-symptom, valid, and reliable self-report questionnaire for clinical and research use. It consists of 25 items over two scales that assess symptom severity and symptom interference with different aspects of a patient's life. Thirteen items (i.e., pain, fatigue, nausea, disturbed sleep, distressed, shortness of breath, remembering things, lack of appetite, drowsy, dry mouth, sadness, vomiting, and numbness or tingling) from the original MDASI-CLL ask patients to rate how severe the symptoms were when "at their worst" in the last 24 hours. An additional six items ask patients to rate how much the symptoms have interfered with six areas of function (i.e., general activity, walking, work, mood, relations with other people, and enjoyment of life) in the last 24 hours. Additionally, the MDASI-CLL contains a tumor-specific module to assess disease-specific symptoms. For this study, to specifically assess CLL symptoms and treatment side effects, patients will rate six additional symptoms (night sweats, fevers and chills, lymph node swelling, diarrhea, bruising easy or bleeding, and constipation). The MDASI-CLL items are rated 0–10 with 0 indicating that the symptom is either not present or does not interfere with the patient's activities and 10 indicating that the symptom is "as bad as you can imagine" or "interfered completely" with the patient's life. The MDASI-CLL takes approximately 5 minutes to complete. The MDASI-CLL assessment will be conducted during Day 1 of each treatment cycle. The MDASI-CLL will be administered at all follow-up visits until new leukemia treatment (NLT).

The EORTC QLQ-C30 (see Appendix 19) is a validated and reliable self-report measure (Fayers et al. 1999) consisting of thirty questions incorporated into five functional scales (physical, role, cognitive, emotional, and social scales), three symptom scales (fatigue, pain, nausea, and vomiting scales), and a global health status/global quality–of–life scale. The remaining single items (dyspnea, appetite loss, sleep disturbance, constipation, and diarrhea) assess the additional symptoms experienced by patients with cancer and the perceived financial burden of treatment. The EORTC QLQ-C30 assessment will be conducted during Day 1 of each treatment cycle. The EORTC QLQ-C30 will be administered at all follow-up visits until NLT.

The EQ-5D-3L (see Appendix 15) questionnaire is a generic, preference-based health utility measure with questions about mobility, self-care, usual activities, pain/discomfort, and anxiety/depression that are used to build a composite of the patient's health status. The EQ-5D-3L will be utilized in this study to estimate quality adjusted life years (QALY) for economic modeling. The EQ-5D-3L also contains a visual analog scale (VAS) to

assess the patient's overall health. The EQ-5D-3L questionnaire takes 5 minutes or less to complete, and assessments are made on Day 1 of each treatment cycle at the same time as the MDASI-CLL and QLQ-C30.

4.5.17 German Chronic Lymphocytic Leukaemia Study Group Biobanking Samples

4.5.17.1 Overview of the GCLLSG Biobanking

Patients will be asked to donate the remaining part of any blood samples taken for central lab assessment at the GCLLSG laboratories for later scientific research. An additional blood sample will also be requested from all patients to evaluate resistance mechanisms and clonal evaluation by genome-wide analyses (e.g., targeted sequencing, whole exome sequencing, whole genome sequencing, RNA sequencing) on samples taken pre-treatment and at time of relapse. Patients may participate in the main study without donating their left-over or additional samples for research.

Left-over and samples for additional research will be frozen in GCLLSG biobanks located at the University of Kiel, Ulm and Cologne and kept for up to 15 years after the last patient recruited. Because scientific knowledge is continuously advancing and analytical techniques are continuously improving, it is currently not clear exactly what analyses will be done and which techniques will be used. However, it is certain that the research will be aimed only at improving the understanding and treatment of CLL.

Decisions on the research to be conducted on stored samples will be made by the GCLLSG Strategy Commission, a group of representative clinicians from the GCLLSG, including the GCLLSG chairman and laboratory scientists linked to their centers. The research will include genetic testing and will be similar to and a logical extension of the central diagnostic tests (e.g., ZAP-70, CD38, IGHV mutation, chromosomal analysis) already included in the protocol.

The results of the currently unspecified research and single nucleotide polymorphism (SNP) testing will be published separately and after publication of the main study. The results will be published in aggregate; individual patients will not be identified. No individual results will be communicated to the investigator or the patient because the research is exploratory and hypothesis-generating and will likely require further confirmatory studies. In the unlikely event that SNP testing or the currently unspecified research reveals robust and definitive information which might be of importance to individual patients or their relatives, Ethics Committee (EC) approval will be sought before any communication of individual results is made to the investigator, the patient, or the patient's relatives.

Samples for genetic testing, i.e. mutation analyses, will be separated into the leukemic and non-leukemic fractions. Separation of the leukemic and non-leukemic compartment will enable definition of disease related mutations.

Such genetic samples collected for analysis of heritable DNA variations (the non-leukemic fraction) will be double coded: a new independent code will be added to the first code to increase confidentiality and data protection (see Section 8.4).

All information, including any genetic information obtained from research on the left-over blood samples, will remain confidential. Research samples will be stored securely but in a way that allows samples to be retrieved from the laboratories and destroyed, if needed (for example, if a patient wishes to withdraw his/her blood samples).

4.5.17.2 Approval by the Institutional Review Board or Ethics Committee

Collection and submission of biological samples to the GCLLSG Biobanking is contingent upon the review and approval of the exploratory research and the GCLLSG portion of the Informed Consent Form by each site's Institutional Review Board (IRB) or EC and, if applicable, an appropriate regulatory body. If a site has not been granted approval for GCLLSG Biobanking sampling, this section (Section 4.5.17) of the protocol will not be applicable at that site.

4.5.17.3 Sample Collection

The following residual samples will be collected for research purposes:

- Residual serum and blood samples extracted under the study protocol for central lab assessment at the GCLLSG labs (University of Kiel, University of Cologne, University of Ulm) may be transferred into GCLLSG biobanking depending on residual sample amount and sample numbers

Any remaining blood from the genetic testing, TP53, and IGVH analyses will be used for research purposes:

- Analyses will include but are not limited to whole exome sequencing analyses, whole genome sequencing, and RNA sequencing

Samples will be stored at the GCLLSG Central Laboratories at the Universities of Ulm and Cologne.

For all samples, dates of consent should be recorded on the associated GCLLSG Biobanking page of the eCRF. For sampling procedures, storage conditions, and shipment instructions, see the laboratory manual.

4.5.17.4 Confidentiality

Given the sensitive nature of genetic data, GCLLSG (hereafter referred to as the Sponsor) has implemented additional processes to ensure patient confidentiality for GCLLSG Biobanking specimens and associated data.

4.5.17.5 Consent to Participate in the GCLLSG Biobanking

The Informed Consent Form will contain a separate section that addresses participation in the GCLLSG Biobanking of leftover samples. The investigator or authorized designee

will explain to each patient the objectives, methods, and potential hazards of participation in the GCLLSG Biobanking. Patients will be told that they are free to refuse to participate and may withdraw their specimens at any time and for any reason during the storage period. A separate, specific signature will be required to document a patient's agreement to provide optional GCLLSG Biobanking specimens. Patients who decline to participate will not provide a separate signature.

The investigator should document whether or not the patient has given consent to participate by completing the Informed Consent eCRF.

In the event of a GCLLSG Biobanking participant's death or loss of competence, the participant's specimens and data will continue to be used as part of the GCLLSG Biobanking research.

4.5.17.6 Withdrawal from the GCLLSG Biobanking

Patients who give consent to provide GCLLSG Biobanking specimens have the right to withdraw their specimens from the GCLLSG Biobanking at any time for any reason. If a patient wishes to withdraw consent to the testing of his or her specimens, the investigator must inform the Medical Monitor in writing of the patient’s wishes through use of the GCLLSG Biobanking Patient Withdrawal Form and, if the trial is ongoing, must enter the date of withdrawal on the GCLLSG Biobanking Research Sample Withdrawal of Informed Consent eCRF. The patient will be provided with instructions on how to withdraw consent after the trial is closed. A patient’s withdrawal from Study BO25323 does not, by itself, constitute withdrawal of specimens from the GCLLSG Biobanking. Likewise, a patient’s withdrawal from the GCLLSG Biobanking does not constitute withdrawal from Study BO25323.

4.6 PATIENT, TREATMENT, STUDY, AND SITE DISCONTINUATION

4.6.1 Patient Discontinuation

Patients have the right to voluntarily withdraw from the study at any time for any reason. In addition, the investigator has the right to withdraw a patient from the study at any time.

Reasons for withdrawal from the study may include but are not limited to the following:

- Patient withdrawal of consent at any time
- Any medical condition that the investigator or Sponsor determines may jeopardize the patient’s safety if he or she continues in the study
- Investigator or Sponsor determines it is in the best interest of the patient

Every effort should be made to obtain information on patients who withdraw from the study. The primary reason for withdrawal from the study should be documented on the appropriate eCRF. However, patients will not be followed for any reason after consent has been withdrawn. Patients who withdraw from the study will not be replaced.

4.6.2 Study Treatment Discontinuation

Patients must permanently discontinue study treatment if they experience any of the following:

- Pregnancy
- Patient non-compliance
- Disease progression
- Adverse events: grade 4 IRR, grade 4 TLS

The primary reason for study treatment discontinuation should be documented on the appropriate eCRF. Patients who discontinue study treatment prematurely will not be replaced.

4.6.3 Study and Site Discontinuation

The Sponsor has the right to terminate this study at any time. Reasons for terminating the study may include but are not limited to the following:

- The incidence or severity of adverse events in this or other studies indicates a potential health hazard to patients
- Unsatisfactory patient enrollment
- Insufficient management of TLS

The Sponsor will notify the investigator of a decision to discontinue the study. The Sponsor has the right to close a site at any time.

Reasons for closing a site may include, but are not limited to, the following:

- Excessively slow recruitment
- Poor protocol adherence
- Inaccurate or incomplete data recording
- Non-compliance with the International Conference on Harmonisation (ICH) guideline for Good Clinical Practice
- No study activity (i.e., all patients have completed and all obligations have been fulfilled)

5. ASSESSMENT OF SAFETY

5.1 SAFETY PLAN

Venetoclax was granted accelerated approval by the FDA in 2016 as monotherapy for the treatment of patients with CLL who have 17p deletion and who have received at least one prior therapy (see Venetoclax US Prescribing Information 2016).

It also received conditional market approval by the EMA in 2016 as monotherapy for the treatment of CLL in the presence of 17p deletion or TP53 mutation in patients who are

unsuitable for or have failed a B-cell receptor pathway inhibitor also as monotherapy for the treatment of CLL in the absence of 17p deletion or TP53 mutation in patients who have failed both chemoimmunotherapy and a B-cell receptor pathway inhibitor (see Venetoclax European Medicines Agency Summary of Product Characteristics 2016).

Clinical experience with venetoclax in CLL is based on several ongoing Phase I, II, and III studies evaluating monotherapy or combination regimens in relapsed/refractory or previously untreated patients (see Venetoclax [ABT-199] Investigator's Brochure). Thus, the entire safety profile is not known at this time. The safety plan for this study is designed to ensure patient safety and will include specific eligibility criteria and monitoring assessments.

5.1.1 Risks Associated with Venetoclax

Clinical experience gained thus far with venetoclax has demonstrated that it is generally well tolerated, and toxicities appear to be mostly manageable and/or reversible; see the Venetoclax Investigator's Brochure and the joint Obinutuzumab/Venetoclax Investigator Brochure for more information.

On the basis of clinical data to date, the following known and potential risks with venetoclax are described below. Guidelines around the management of these risks through dose and schedule modifications are described in Appendix 2 and Appendix 3.

5.1.1.1 Tumor Lysis Syndrome

To date, the most clinically important adverse reaction associated with venetoclax in the ongoing clinical program has been TLS, primarily related to the initial ramp-up phase. TLS, including cases leading to clinical sequelae (of which two have had a fatal outcome), has been observed in patients with CLL treated with venetoclax at doses of 50 mg and higher. TLS prophylaxis and monitoring measures implemented in all protocols involving patients with CLL/SLL since May 2013, including initiation of therapy at the 20-mg dose and more gradual, five-step–dose ramp-up, have effectively reduced the severity and risk of TLS in patients with CLL/SLL.

Although there is a general consensus for a broad definition of TLS as a set of metabolic complications that can arise from treatment of rapidly proliferating neoplasm, there have been very few attempts to apply a specific definition to the syndrome (Hande and Garrow 1993; Razis et al. 1994; Kedar et al. 1995). Cairo and Bishop (2004) further built on the Hande and Garrow definition by modifying the definition to account for baseline laboratory abnormalities as well as changes occurring within 7 days following treatment. This definition was further refined in 2011 (Howard et al. 2011; see Appendix 20).

In light of potential synergy between venetoclax and obinutuzumab, rigorous TLS monitoring and prophylaxis will be instituted in this study (Section 5.1.3.1). If it is

clinically justified and by laboratory assessment, patients can be re-categorized in a lower TLS-risk group during the venetoclax ramp-up period.

5.1.1.2 Cytopenia

Effects on lymphocyte numbers are expected on the basis of the mechanism of action, and modest reductions in neutrophils have been observed with venetoclax therapy in oncology patients. The majority of serious events of neutropenia and febrile neutropenia were confounded with poor bone marrow reserves, multiple prior therapies, disease infiltrating the bone marrow, and the use of growth factor prior to entering the studies.

Thrombocytopenia and anemia have been reported with venetoclax in the ongoing single-agent Phase I dose–escalation Study M12-175 that is being conducted in heavily pretreated patients with CLL and NHL. In most cases, the condition was preexisting.

Treatment-emergent adverse events of neutropenia, anemia, and thrombocytopenia showed a higher incidence in a combination study with rituximab (Study M13-365). Cytopenias, including neutropenia, anemia, and thrombocytopenia, are known adverse reactions of anti-CD20 monoclonal antibodies. There is still insufficient evidence to suggest a higher risk when these agents are used in combination with venetoclax.

In this study, blood counts will be monitored closely throughout treatment (see the Schedule of Assessments in Appendix 2 and Appendix 3. Growth factors are permitted according to local practice, and patients will be monitored and treated promptly in case of infections. Dose interruptions or reductions will be allowed on the basis of toxicity.

5.1.1.3 Infectious Complications

Infections of various types have occurred in patients in the ongoing, single-agent, Phase I dose-escalation Study M12-175. The types of infectious events have been consistent with those anticipated in the elderly population of heavily pretreated patients with CLL. CLL can be associated with impaired immune function and increased infections, and it is unclear whether or how much the incidence could be further increased due to venetoclax treatment. Patients in this study will be closely monitored for infections and prompt therapy will be instituted, as necessary. Patients are allowed to receive concomitant prophylactic anti-infective therapy at the investigator’s discretion.

5.1.1.4 Effects on Cardiac Function

No patterns of adverse events indicating changes in cardiac function have been reported in clinical studies to date. However, since study populations in venetoclax oncology studies are likely to be elderly and have received multiple prior chemotherapeutic agents, patients enrolled in this study are required to have ECGs and assessments of LVEF at screening per investigator’s discretion and as clinically indicated afterward.

5.1.1.5 Effects on Fertility

Given non-clinical data, there is a potential for decreased spermatogenesis. Male patients considering preservation of fertility should bank their sperm before treatment with venetoclax. Long-term effects of venetoclax on either male or female reproductive potential are unknown.

5.1.1.6 Drug Interactions

Drug-drug interactions may occur with venetoclax. Venetoclax is eliminated almost entirely through the hepatic route via metabolism by CYP3A4. Specific recommendations are provided for co-administration of venetoclax with inhibitors and inducers of CYP3A (see Section 4.4.2.1.1, Table 4, and Appendix 5). Venetoclax does not appear to be a clinically significant inhibitor of CYP2C9 or CYP2C8. Venetoclax is not an inducer of CYP1A2, CYP2B6, or CYP3A4; therefore, it is not expected to have any potential for drug-drug interaction via CYP induction. Venetoclax is a substrate of the transporters P-gp and BCRP and an inhibitor of P-gp, BCRP and OATP1B1.

On a case by case basis, equivalent drugs, which do not interact with venetoclax, will be selected. If there is no substitute available for a vital drug, withdrawal of the patient from the study is necessary.

5.1.2 Risks Associated with Obinutuzumab

Important risks associated or potentially associated with obinutuzumab are TLS, thrombocytopenia (including acute thrombocytopenia), neutropenia, prolonged B-cell depletion, infections (including PML and HBV reactivation), worsening of pre-existing cardiac conditions, impaired immunization response, immunogenicity, gastrointestinal perforation, and second malignancies. Physicians should exercise caution when treating patients with a history of recurring or chronic infections or with underlying conditions that may predispose patients to infections. Signs and/or symptoms of infection should result in prompt evaluation and collection of appropriate samples for bacteriological investigation prior to starting antibiotic or other treatment. Patients may receive concomitant prophylactic anti-infective therapy at the physician's discretion.

5.1.2.1 Infusion-Related Reactions

IRRs are seen with the first infusion in virtually all patients with CLL treated with obinutuzumab. The IRRs are most commonly characterized by symptoms of hypotension, fever, chills, flushing, nausea, vomiting, hypertension, dyspnea, and fatigue. IRRs occur predominantly during the first infusion, and their incidence and intensity decrease with subsequent infusions. They generally appear early during the infusion or shortly after, or in some cases, up to 24 hours after the completion of infusion of obinutuzumab. Some patients have developed severe IRRs resulting in permanent discontinuation of obinutuzumab. In some instances, concurrent signs of TLS are observed. To mitigate the risk for IRRs, the following measures are included in the study protocol:

- Patients who have preexisting cardiac or pulmonary conditions or who have had a prior clinically-significant cardiopulmonary adverse event with obinutuzumab should be monitored carefully throughout the infusion and the post-infusion period.
- Dosing should be split for the first infusion only (100 mg on Cycle 1, Day 1 and 900 mg on Cycle 1, Day 2).

Management of IRRs and anaphylaxis is described in Section 5.3.5.1.

5.1.2.2 Tumor Lysis Syndrome

Both venetoclax and obinutuzumab cause rapid cell breakdown after initial dosing of patients with CLL, and administration of both agents individually has been associated with events of laboratory and/or clinical TLS, which can be fatal. Management of TLS is described in Appendix 10.

5.1.2.3 Neutropenia

Some patients who were treated with obinutuzumab developed NCI Common Terminology Criteria for Adverse Events (CTCAE) Grade 3–4 neutropenia, including febrile neutropenia.

Increased infections may occur due to neutropenia. Growth factors may be given during treatment with the combination, and patients will be monitored and treated promptly in case of infections.

5.1.2.4 Lymphopenia

There is a potential for clinically significant lymphopenia. Given the mechanism of action of B-cell depletion with obinutuzumab and inhibition of Bcl-2 with venetoclax, it is possible that there could be a delay in B-cell recovery.

5.1.2.5 Thrombocytopenia

Cases of Grade 3–4 thrombocytopenia have been reported with obinutuzumab.

A higher incidence of thrombocytopenia and hemorrhagic events was observed during the first cycle in patients with CLL with coexisting medical conditions treated with GClb

as compared with patients treated with RClb or chlorambucil alone in the Phase III pivotal Study BO21004/CLL11.

The incidence of all grade and fatal hemorrhagic events was similar across the study arms. However, all four fatal hemorrhagic events in GClb patients occurred in Cycle 1, compared with none in RClb-treated patients and one in chlorambucil-treated patients.

Due to the small number of patients with fatal hemorrhagic events, lack of laboratory data (platelet count), specifically on the day of the hemorrhagic event, and the presence of confounding factors in all cases (pre-existing thrombocytopenia due to CLL, concomitant medical conditions, and concomitant treatments such as platelet inhibitors, and anticoagulants), no clear relationship could be established between thrombocytopenia and fatal hemorrhagic events.

5.1.2.6 Infection

Consistent with its intended mode of action resulting in profound B-cell depletion, obinutuzumab has been shown to be associated with an increased risk of infections.

Either new infection or reactivation of viral infections may occur. Serious viral infections may include herpes virus infections (including cytomegalovirus, John Cunningham virus infection (JCV), PML, and hepatitis C virus [HCV] infection). The eligibility of patients who are thought to have passive transfer of HCV or HBV core surface antibodies from IV immunoglobulin administration must be discussed with the Medical Monitor.

Patients with HCV or HBV antibody following administration of IV immunoglobulin may be eligible only if PCR is negative for HCV or HBV, respectively. Patients with positive HBV antibodies must be willing to undergo monthly HBV DNA testing. Patients with serological evidence of HBV or HCV will not be eligible for treatment in this study.

Hepatitis B Virus Reactivation

For the subset of patients who are HBsAg negative and HBcAb positive and have undetectable HBV DNA levels at screening, HBV DNA levels must be followed approximately every 3–4 weeks during the treatment phase and then approximately every 4 weeks during the follow-up phase of the study. Treatment with GClb/ obinutuzumab + venetoclax will be held for patients with a serum HBV DNA level of ≥29 IU/mL. Patients should begin treatment with anti-viral medication immediately after the first report that shows HBV DNA ≥29 IU/mL. Retest the HBV DNA level as soon as possible to rule out a false-positive report and to confirm HBV reactivation (≥29 IU/mL) (best prior to treatment; [e.g., when prescribing the anti-viral treatment]). If HBV reactivation is confirmed in a second test, continue to treat for at least 1 year after the last dose of rituximab or obinutuzumab and immediately refer the patient to a gastroenterologist or hepatologist for additional management. If a second test does not confirm HBV reactivation and was taken prior to the start of anti-viral therapy, then anti-viral therapy may be stopped again, and GClb/ obinutuzumab + venetoclax may be

resumed. If a second test does not confirm HBV reactivation but was potentially confounded because it was taken only after the start of anti-viral therapy, then continue to treat for at least 1 year after the last dose of obinutuzumab and refer the patient to a gastroenterologist or hepatologist for additional management. Patients may resume GClb/ obinutuzumab + venetoclax after HBV DNA levels decrease to undetectable levels (< 10 IU/mL) or if retest does not confirm HBV reactivation. If the HBV DNA level exceeds 100 IU/mL and is increasing while a patient is receiving anti-viral medication, treatment with GClb / obinutuzumab + venetoclax will be discontinued.

If HBV DNA level is detectable but < 29 IU/mL, then continue with GClb/obinutuzumab + venetoclax, but retest at close intervals (e.g., every 3–4 weeks). If HBV DNA is still detectable but < 29 IU/mL on retest, then continue to administer treatment and retest at close intervals (e.g., every 3–4 weeks). If HBV DNA is ≥ 29 IU/mL on retest, then follow the instructions for HBV DNA levels ≥ 29 IU/mL (see above).

Table 5 Management of Hepatitis B Virus Reactivation

HBV DNA level of ≥29 IU/mL	• Hold GClb/ obinutuzumab + venetoclax.<br>• Begin anti-viral medication immediately after the first report showing HBV DNA ≥29 IU/mL<br>• Retest HBV DNA level as soon as possible to rule out a false-positive report and to confirm HBV-reactivation (≥29 IU/mL) (best prior to treatment; e.g., when prescribing the anti-viral treatment).<br>• If HBV reactivation is confirmed in second test, continue to treat for at least 1 year after the last dose of obinutuzumab and refer the patient to a gastroenterologist or hepatologist for additional management.<br>• If a second test does not confirm HBV reactivation and was taken prior to start of anti-viral therapy, then anti-viral therapy may be stopped again, and GClb/ obinutuzumab + venetoclax may be resumed. Retest the patient at close intervals (every 3–4 weeks).<br>• If a second test does not confirm HBV reactivation but was potentially confounded because it was taken only after the start of ant-viral therapy, then continue to treat for at least 1 year after the last dose of obinutuzumab and refer the patient to a gastroenterologist or hepatologist for additional management.<br>• Resume GClb/ obinutuzumab + venetoclax once HBV DNA levels decrease to undetectable levels (<10 IU/mL) or if retest does not confirm hepatitis B reactivation.
HBV DNA level of >100 IU/mL and increasing while on appropriate anti-viral medication	Discontinue GClb/ obinutuzumab + venetoclax.
HBV DNA level detectable but <29 IU/mL	• Continue with GClb/ obinutuzumab + venetoclax, but retest at close intervals (e.g., every 3–4 weeks).<br>If HBV DNA still detectable, but <29 IU/mL on retest<br>• Continue to administer treatment and retest at close intervals (e.g., every 3–4 weeks).<br>If HBV DNA ≥29 IU/mL on retest<br>• Follow instructions for HBV DNA level of ≥29 IU/mL (see above).

GClb = obinutuzumab + chlorambucil; HBV = hepatitis B virus.

PML has been reported in patients who have received obinutuzumab. Particular attention should be given to patients who have had significant prior immunosuppressive treatment, such as high-dose chemotherapy or a stem cell transplant, and physicians should be aware of symptoms that are suggestive of PML and consider the diagnosis of PML in any patient presenting with new-onset neurologic manifestations.

The symptoms of PML are very unspecific and can vary depending on the affected region of the brain. Motor involvement with corticospinal tract findings, sensory involvement, cerebellar deficits, and visual field defects are common. Some syndromes regarded as “cortical” (e.g., aphasia or visual-spatial disorientation) can occur.

Evaluation of PML includes, but is not limited to, consultation with a neurologist, brain MRI, and lumbar puncture to quantify DNA of JCV in the cerebrospinal fluid.

Therapy with obinutuzumab should be withheld during the investigation of potential PML and permanently discontinued in case of PML. Discontinuation or reduction of any concomitant chemotherapy or immunosuppressive therapy should also be considered. The patient should be referred to a neurologist for the treatment of PML.

Physicians should exercise caution when treating patients with a history of recurring or chronic infections or with underlying conditions that may predispose patients to infections. Signs and/or symptoms of infection should result in prompt evaluation and appropriate samples for bacteriological investigation prior to starting antibiotic or other treatment. Patients may receive concomitant prophylactic anti-infective therapy at the physician’s discretion.

5.1.2.7 Immunization

The safety of immunization with live viral vaccines following obinutuzumab therapy has not been studied. Patients who participate in this study may not receive vaccination with a live vaccine for a minimum of 28 days prior to randomization.

Investigators should review the vaccination status of potential study patients being considered for this study and follow the U.S. Centers for Disease Control and Prevention guidelines for adult vaccination with non-live vaccines intended to prevent infectious diseases prior to study therapy.

5.1.2.8 Gastrointestinal Perforation

Gastrointestinal perforations, including fatal events, have been reported in patients treated with obinutuzumab. Patients with gastrointestinal involvement should be monitored for signs of gastrointestinal perforation.

5.1.2.9 Risks Associated with the Combination of Venetoclax and Obinutuzumab

Given their mechanisms of action and review of available single-agent safety data, additive or overlapping acute toxicities for the combination of venetoclax and obinutuzumab could potentially include neutropenia and TLS. In terms of chronic toxicity, it is possible that inhibition of Bcl-2 could delay B-cell recovery following completion of obinutuzumab treatment and/or lead to combined B- and T-cell depletion. These effects, if they occur, would likely manifest as an increased incidence of infections only after several months of exposure to the combination. Given the degree of immunodeficiency associated with the underlying disease, all patients will be monitored closely for infection

and treated aggressively according to institutional guidelines. Additionally, all patients will have lymphocyte subset measurements by flow cytometry throughout the study treatment and follow-up period (also see the joint Venetoclax/Obinutuzumab Investigator's Brochure).

5.1.3 Management of Specific Adverse Events

Guidelines for management of specific adverse events are outlined in Table 6. Additional guidelines are provided in the subsections below.

Table 6 Guidelines for Management of Specific Adverse Events (for Patients Randomized to Arms A or B)

Event	Dose delay or dose modification
Grade 3 or 4 (NCI grading scale) neutropenia with or without fever and infection	• Withhold venetoclax (and obinutuzumab if neutropenia occurs during Cycles 1–6) for at least 7 days.<br>• For chlorambucil: withhold chlorambucil until adverse event resolves to Grade 2.<br>• Administer G-CSF or growth factors for neutropenia as indicated.<br>• When counts recover to ANC $\geq 1 \times 10^9$/L and/or platelets are $\geq 75 \times 10^9$/L, resume venetoclax at one dose level reduction.<br>• Reinitiate obinutuzumab.
Severe thrombocytopenia, (platelets < 25 000/μL) and/or symptomatic bleeding	• Withhold venetoclax or chlorambucil (and obinutuzumab if event occurs during Cycles 1–6) for severe thrombocytopenia (platelets < 25,000/μL) or presence of symptomatic bleeding until resolution of bleeding.<br>Platelets may be transfused at the discretion of the investigator.<br>When platelet level rises to > 50,000/μL without transfusional support for 5 consecutive days, restart venetoclax at previous doses and obinutuzumab.<br>• For a second episode of severe thrombocytopenia and/or symptomatic bleeding, withhold venetoclax or chlorambucil (and obinutuzumab if event occurs during Cycles 1–6).<br>When platelet level rises to > 50, 000/μL without transfusional support for 5 consecutive days, restart venetoclax at one dose level reduction and obinutuzumab.<br>• For subsequent episodes of severe thrombocytopenia, withhold venetoclax or chlorambucil (and obinutuzumab if event occurs during Cycles 1–6).<br>When platelet level rises to > 50,000 μL without transfusional support for 5 consecutive days, restart venetoclax at one dose level reduction and obinutuzumab.<br>• For recurrent severe thrombocytopenia in spite of dose reduction and/or symptomatic bleeding, consult the Medical Monitor regarding continuation on the protocol.

Table 6 Guidelines for Management of Specific Adverse Events (for Patients Randomized to Arms A or B) (cont.)

Non-hematologic toxicity	
Event	Dose delay or dose modification
Grade 4 IRR	• Discontinue obinutuzumab permanently. Patients may continue venetoclax or chlorambucil.
Grade 3 IRR on first dose; the same grade 3 IRR on two subsequent occasions	• First episode: To be managed at investigator's discretion.<br>• After 2 subsequent episodes of the same grade 3 event: Discontinue obinutuzumab permanently. Patients may continue venetoclax or chlorambucil.
Grade 1–2 IRR, first and subsequent episodes	• To be managed at investigator's discretion
Grade 3 or 4 TLS, first episode and subsequent episodes	• See Appendix 10.
Grade 3 or 4 non-hematologic events not specifically described above	• Delay venetoclax (and obinutuzumab if event occurs during Cycles 1–6) for a maximum of 28 days.<br>First episode: If improvement to Grade ≤ 1 or baseline, resume previous doses of venetoclax and obinutuzumab.<br>For subsequent episodes: If improvement to Grade ≤ 1 or baseline, restart venetoclax at one dose level reduction.<br>• Delay chlorambucil (and obinutuzumab if event occurs during Cycles 1–6) for a maximum of 28 days.<br>First episode: If improvement to Grade ≤ 1 or baseline, resume previous doses of chlorambucil and obinutuzumab.<br>For subsequent episodes: If improvement to Grade ≤ 1 or baseline, dose reduce chlorambucil per local guidelines.<br>• Certain treatment emergent non-hematologic adverse events (e.g., venous thromboembolic events) may be managed and become clinically stable following medical intervention but may not improve to Grade ≤ 1 according to the NCI CTCAE definitions. In such cases, if a patient is clinically stable, resumption of study drug may be possible after consultation with the Medical Monitor.
Grade 2 non-hematologic toxicity	• Delay treatment with venetoclax or chlorambucil (and obinutuzumab if event occurs during Cycles 1–6) until resolution to Grade ≤ 1 (or baseline status) for a maximum of 28 days.<br>• After resolution, resume full dose of venetoclax or chlorambucil and obinutuzumab.
Grade 1 non-hematologic toxicity	• No dose reduction or delay.

ANC = absolute neutrophil count; CTCAE = Common Terminology Criteria for Adverse Events; G-CSF = granulocyte stimulating factor; HBV = hepatitis B virus; NCI = National Cancer Institute; WHO = World Health Organization.

Gradual dose increase of venetoclax or chlorambucil following resolution of toxicity leading to a dose reduction may be considered if the patient is stable for 2 weeks on the lower dose; however, if the toxicity recurs, the patient may continue treatment on the lower dose.

Dose reduction of obinutuzumab is not permitted.

Patients who discontinue venetoclax or chlorambucil for toxicity should also discontinue obinutuzumab, although they are to continue evaluation per protocol.

Patients who interrupt all study treatments secondary to treatment-related adverse events for longer than 28 days should discontinue all study drugs although they are to continue being followed for disease progression as described in Appendix 1, Appendix 2, and Appendix 3.

The evaluation of potential treatment-induced toxicity in patients with advanced CLL may be quite difficult requiring careful consideration of both the manifestations of the underlying disease, as well as adverse reactions to the therapy under study. Some of the conventional criteria for toxicity are not applicable especially under circumstances of progressive bone marrow failure from the CLL itself.

Dose modifications for hematological toxicity in patients with CLL must consider the increased frequency of hematological compromise at the initiation of therapy. Therefore the standard criteria used for solid tumors are difficult to be applied directly; many patients would be considered to have Grade II to IV hematological toxicity at presentation.

As a consequence, dose modification decisions for patients with cytopenia (below the lower limit of the normal range) at baseline will be based on the NCI grading scale for hematological toxicity in CLL studies (see Table 7). For patients with a normal neutrophil count, platelet count, and/or hemoglobin value at baseline, the NCI CTCAE Version 4.03 will be used.

Table 7 National Cancer Institute Grading Scale for Chronic Lymphocytic Leukemia

Decrease in Platelets or Hemoglobin from Pre-Treatment (%)	Grade	ANC/μL
No change to 10	0	≥2000
11–24	1	≥1500 and <2000
25–49	2	≥1000 and <1500
50–74	3	≥500 and <1000
≥75	4	<500

ANC = absolute neutrophil count.

5.1.3.1 Prophylaxis and Management of Tumor Lysis Syndrome for Patients Being Treated with Venetoclax

TLS is a risk for patients with CLL who are treated with high cell-killing agents or obinutuzumab. Clinical data from patients with CLL treated to date with venetoclax suggest that patients with baseline lymph nodes ≥ 5 cm diameter are at a greater risk for TLS than those with baseline lymph nodes < 5 cm. In addition, the data showed that CrCl of ≤80 mL/min at screening was a secondary risk factor for TLS. A detailed description of risk factors for developing TLS following treatment with venetoclax is available in the Venetoclax Investigator's Brochure.

Based on the data review performed by the Sponsors, the following three risk categories for developing TLS after treatment with venetoclax were defined (see Table 8). These risk groups were developed using assessments of nodal disease burden obtained from imaging tests performed at screening in several other studies of venetoclax in CLL. Therefore, assigning a patient's TLS risk MUST be made based on an imaging test performed at screening (see Section 4.5.13.3). Assessments of TLS risk categorization and monitoring/prophylaxis guidance is being continuously assessed throughout the venetoclax program, and future updates to these guidelines are possible.

Table 8 Risk Categories for Developing Tumor Lysis Syndrome

TLS Risk Category	Definition
Low	All measurable lymph nodes with the largest diameter < 5 cm and $< 25 \times 10^9$/L ALC. Lymph node size will be determined by radiologic assessment.
Medium	Any measurable lymph node with the largest diameter ≥ 5 cm but < 10 cm OR $\geq 25 \times 10^9$/L ALC. Lymph node size will be determined by radiologic assessment.
High	Any measurable lymph node with the largest diameter ≥ 10 cm or the presence of both $\geq 25 \times 10^9$/L ALC AND any measurable lymph node with the largest diameter ≥ 5 cm but < 10 cm. Lymph node size will be determined by radiologic assessment.

ALC = absolute lymphocyte count; CT = computed tomography; MRI = magnetic resonance imaging; TLS = tumor lysis syndrome.

TLS Risk Assessment MUST be made based on measurements of nodal disease burden based on radiologic assessments (CT scan or MRI, see Section 4.5.13.3) performed during the screening period.

All patients enrolling in the study will be assessed at screening and categorized in a risk category as described above. Investigators may reassess patient’s TLS risk after they start treatment and may assign them to a lower risk group. For example, patients classified as TLS high-risk at screening because of an ALC of $\geq 25 \times 10^9$ AND a measurable lymph node with the largest diameter ≥ 5 cm but < 10 cm by radiologic assessment may have a re-evaluation of their TLS risk category based on their most recent ALC after initiating study treatment. If the patient’s ALC decreases to $< 25 \times 10^9$/L, the patient may be categorized as TLS medium-risk and may follow the management guidelines for the TLS medium-risk category at subsequent visits and during the venetoclax ramp-up period. Re-assessment of the patient’s TLS risk category can occur continuously during Cycle 1 and the venetoclax dose ramp-up period. If a patient is re-assigned to a lower risk group, the investigator may follow the prophylaxis guidance for the lower risk group to which they are assigned.

However, patients who are classified as high-risk because they have a lymph node with largest diameter ≥ 10 cm MAY NOT have their TLS risk re-assessed and should follow the prophylaxis plan for high-risk patients throughout the venetoclax ramp-up period.

Patients who develop signs or symptoms of TLS regardless of the risk group to which they were assigned may have additional monitoring at subsequent visits at the investigator’s discretion.

This section describes the management of patients throughout dosing (as described in Section 4.3.2) given their risk factors for developing TLS identified upon study entry.

Table 9 Summary of Tumor Lysis Syndrome Prophylaxis for Venetoclax and Monitoring Measures

TLS Risk Category	Day 1 of Dose Level	Prophylaxis Medication	Hospitalization	Hydration [a]	Laboratory Assessments [b, e]
TLS low-risk	20, 50, 100, 200, 400 mg	Oral uric acid reducer (such as allopurinol 300 mg/day) beginning at least 72 hours prior to dose and continued until the end of the ramp-up period with venetoclax is completed (C3D1).	No	Oral hydration of 1.5–2 L/day beginning at least 48 hours prior to dose and continuing for at least 24 hours after dose.	Hematology and chemistry samples will be taken predose and 8 and 24 hours after dosing.<br>Predose is defined as up to 4 hours before venetoclax administration, and results must be reviewed prior to dosing; if it is not possible to review results from a sample taken up to 4 hours predose, then it is acceptable to take a predose hematology and chemistry sample within 24 hours prior to dosing. The results of these samples must be reviewed prior to dosing. If laboratory values from this sample have demonstrated no clinically significant abnormalities, the hematology and chemistry samples drawn on the day of venetoclax administration prior to dosing are not required to be reviewed prior to dose administration. However, these predose (0–4 hours prior to dosing) laboratory samples should still be drawn, and these will serve as baseline for later laboratory values when assessing for laboratory evidence of TLS at the 8 and 24 hours after dosing timepoints.<br>The 8-hour chemistry results must be reviewed before the patient leaves the outpatient clinic that day.<br>The investigator or subinvestigator must review the 24-hour laboratory results prior to dosing on the next day.

Table 9 Summary of Tumor Lysis Syndrome Prophylaxis for Venetoclax and Monitoring Measures (cont.)

TLS medium-risk	20 and 50 mg	Oral uric acid reducer (such as allopurinol 300 mg/day) beginning at least 72 hours prior to dose and continued until the end of the ramp up period with venetoclax is completed (C3D1).	No [c,d]	Oral hydration of 1.5–2 L/day beginning at least 48 hours prior to dose and continuing for at least 24 hours after dose.<br>In addition to oral hydration, IV hydration (1.5–2 L) will be given in the outpatient setting during the clinic stay.	Hematology and chemistry samples will be taken predose and 8, and 24 hours after dosing timepoints.<br>Predose is defined as up to 4 hours before venetoclax administration, and results must be reviewed prior to dosing; if it is not possible to review results from a sample taken up to 4 hours predose, then it is acceptable to take a predose hematology and chemistry sample within 24 hours prior to dosing. The results of these samples must be reviewed prior to dosing. If laboratory values from this sample have demonstrated no clinically significant abnormalities, the hematology and chemistry samples drawn on the day of venetoclax administration prior to dosing are not required to be reviewed prior to dose administration. However, these predose (0–4 hours prior to dosing) laboratory samples should still be drawn, and these will serve as baseline for later laboratory values when assessing for laboratory evidence of TLS at the 8 and 24 hours after dosing timepoints.<br>The 8-hour chemistry results must be reviewed before the patient leaves the outpatient clinic that day.<br>The investigator or subinvestigator must review the 24-hour laboratory results prior to dosing on the next day.
	100, 200, 400 mg	Continue oral uric-acid reducer as above.		Oral hydration of 1.5–2 L/day beginning at least 48 hours prior to dose and continuing for at least 24 hours after dose.	

Table 9 Summary of Tumor Lysis Syndrome Prophylaxis for Venetoclax and Monitoring Measures (cont.)

TLS Risk Category	Day 1 of Dose Level	Prophylaxis Medication	Hospitalization	Hydration [a]	Laboratory Assessments [b, e]
TLS high-risk	20 and 50 mg	Oral uric acid reducer (such as allopurinol 300 mg/day) beginning at least 72 hours prior to dose and continued until the first week of combination therapy with venetoclax is completed.<br>Rasburicase must be administered per regional standards/institutional guidelines as prophylaxis prior to the first dose of venetoclax for high-risk patients with high uric acid levels at pre-dose (above the local laboratory ULN or the Howard et al. [2011] threshold of 8 mg/dL (475.8 μmol/L). For patients with a contraindication to rasburicase (i.e., glucose 6 phosphate dehydrogenase deficiency), the TLS risk-mitigation plan must be reviewed with the Medical Monitor. Uric acid levels following treatment with rasburicase must be analyzed using specific guidelines described in Section 3.1.2.2	Yes [d]	Oral hydration of 1.5–2 L/day beginning at least 48 hours prior to dose and continuing for at least 24 hours after dose.<br>Upon hospital admission, IV hydration should be started with a target of approximately 2–3 L per day or as clinically appropriate.	Hematology and chemistry samples will be taken predose and 8, 12, and 24 hours after dosing.<br>Predose is defined as up to 4 hours before venetoclax administration, and results must be reviewed prior to dosing; if it is not possible to review results from a sample taken up to 4 hours predose, then it is acceptable to take a predose hematology and chemistry sample within 24 hours prior to dosing. The results of these samples must be reviewed prior to dosing. If laboratory values from this sample have demonstrated no clinically significant abnormalities, the hematology and chemistry samples drawn on the day of venetoclax administration prior to dosing are not required to be reviewed prior to dose administration. However, these predose (0–4 hours prior to dosing) laboratory samples should still be drawn, and these will serve as baseline for later laboratory values when assessing for laboratory evidence of TLS.<br>The investigator or subinvestigator must review the 24-hour laboratory results prior to dosing on the next day.

Table 9 Summary of Tumor Lysis Syndrome Prophylaxis for Venetoclax and Monitoring Measures (cont.)

TLS Risk Category	Day 1 of Dose Level	Prophylaxis Medication	Hospitalization	Hydration [a]	Laboratory Assessments [b, e]
TLS high-risk	100, 200, 400 mg	Continue oral uric acid reducer as above	No [c,d]	Oral hydration of 1.5–2 L/day beginning at least 48 hours prior to dose and continuing for at least 24 hours after dose.<br>In addition to oral hydration, IV hydration (1.5–2L) will be given in the outpatient setting during the clinic stay.	Patients who are not hospitalized at these timepoints will have hematology and chemistry samples taken predose and 8 and 24 hours after dosing.<br>Predose is defined as up to 4 hours before venetoclax administration, and results must be reviewed prior to dosing; if it is not possible to review results from a sample taken up to 4 hours predose, then it is acceptable to take a predose hematology and chemistry sample within 24 hours prior to dosing. The results of these samples must be reviewed prior to dosing. If laboratory values from this sample have demonstrated no clinically significant abnormalities, the hematology and chemistry samples drawn on the day of venetoclax administration prior to dosing are not required to be reviewed prior to dose administration. However, these predose (0–4 hours prior to dosing) laboratory samples should still be drawn, and these will serve as baseline for later laboratory values when assessing for laboratory evidence of TLS.

Table 9 Summary of Tumor Lysis Syndrome Prophylaxis for Venetoclax and Monitoring Measures (cont.)

TLS Risk Category	Day 1 of Dose Level	Prophylaxis Medication	Hospitalization	Hydration [a]	Laboratory Assessments [b, e]
					The investigator or subinvestigator must review the 24-hour laboratory results prior to dosing on the next day.<br>Patients who are hospitalized at these timepoints will have chemistry and hematology samples obtained predose, 8, 12, and 24 hours postdose. These results must be reviewed promptly by the investigator or subinvestigator. The 24 hour postdose laboratory results must be reviewed by the investigator or subinvestigator before the patient leaves the hospital or receives any additional study drug.

C = cycle; CrCl = creatinine clearance; D = day; IV = intravenous; TLS = tumor lysis syndrome; ULN = upper limit of normal.

[a] For patients unable to maintain oral hydration at 1.5–2 L/day starting at least 48 hours prior to the start of treatment, IV hydration in the outpatient setting on the day of dosing during the clinic stay is recommended (unless being hospitalized) in order to assure that this full amount of hydration is achieved. For patients for whom volume overload is considered a significant risk, hospitalization should be considered.

[b] For laboratory samples drawn on days of study treatment, "predose" laboratory samples should be drawn within 0–4 hours before the dose. Other laboratory samples occurring on the same day should be obtained within a ± 15-minute window of any exact scheduled time. Any laboratory tests occurring at time intervals greater than or equal to 24 hours after dose should be obtained within a ± 2-hour window of the scheduled time. If it is not possible to review a sample taken up to 4 hours predose, then it is acceptable to take a predose hematology and chemistry sample within 24 hours prior to dosing. The results of these samples must be reviewed prior to dosing. If laboratory values from this sample have demonstrated no clinically significant abnormalities, the hematology and chemistry samples drawn on the day of venetoclax administration prior to dosing are not required to be reviewed prior to dose administration. However, these predose (0–4 hours prior to dosing) laboratory samples should still be drawn, and these will serve a baseline for later laboratory values when assessing for laboratory evidence of TLS.

Table 9 Summary of Tumor Lysis Syndrome Prophylaxis for Venetoclax and Monitoring Measures (cont.)

[c] Patients with CrCl < 80 mL/min and/or who have a higher tumor burden (defined per the discretion of the investigator) may be handled as TLS high-risk patients. Currently, limited clinical experience has been gained with venetoclax in patients with CrCl 30–50mL/min. Therefore, these patients should receive additional consideration by the investigator with regard to their management, including the decision on whether to administer IV hydration and to hospitalize the patient to facilitate monitoring and expedite response to electrolyte changes at initial dosing as well as at each first dose during the ramp-up period.

[d] Nephrology (or acute dialysis service) consultation should be considered on admission (per institutional standards or based on investigator discretion) for hospitalized patients to ensure emergency dialysis is available and the appropriate staff is aware and prepared to handle any necessary intervention for TLS. Telemetry should also be considered.

[e] Any patient who, at any dose, develops clinically significant electrolyte abnormalities must have subsequent venetoclax dose withheld until the electrolyte abnormalities resolve. Patients who develop electrolyte abnormalities should undergo aggressive management and further monitoring per Appendix 4. Any time during the ramp-up period, if venetoclax was withheld for 7 days or less, the patient may resume venetoclax at the same dose level or at one lower dose-level as determined by the investigator based on a risk assessment (including tumor burden status). The dose must be resumed at one lower dose-level if dose was withheld more than 7 days, with the exception of initial dose level of 20 mg (400 mg → 200 mg, 200mg → 100 mg, 100 mg → 50 mg, 50 mg→ 20 mg).

Initial Doses: Venetoclax 20 and 50 mg

All patients, irrespective of their TLS risk category at the first dose of venetoclax, must receive the following TLS prophylaxis measures prior to the initiation of the first doses of venetoclax:

- Administration of an oral uric acid reducer (such as allopurinol 300 mg/day) beginning at least 72 hours prior to dose and continued to the end of the venetoclax ramp-up period (Cycle 3, Day 1).
- Oral hydration consisting of fluid intake of approximately 1.5–2 L/day starting at least 48 hours prior to the start of treatment and continued for at least 24 hours after the first dose
- Serum chemistry and hematology laboratory samples must be drawn prior to administering venetoclax (predose). If clinically significant laboratory abnormalities are observed in this baseline laboratory assessment, the first dose of venetoclax must be delayed until resolution and management per the protocol and recommendations for Initial Management of Electrolyte Imbalances and Prevention of TLS must be initiated. If needed, patient should receive additional prophylactic treatment prior to the initiation of dosing.

  Predose is defined as up to 4 hours before venetoclax administration, and results must be reviewed prior to dosing; if it is not possible to review results from a sample taken up to 4 hours predose, then it is acceptable to take a predose hematology and chemistry sample within 24 hours prior to dosing. The results of these samples must be reviewed prior to dosing. If laboratory values from this sample have demonstrated no clinically significant abnormalities, the hematology and chemistry samples drawn on the day of venetoclax administration prior to dosing are not required to be reviewed prior to dose administration. However, these predose (0–4 hours prior to dosing) laboratory samples should still be drawn, and these will serve as baseline for later laboratory values when assessing for laboratory evidence of TLS.

Additional TLS prophylaxis and monitoring procedures are tailored to the individual TLS risk category as follows.

TLS Low Risk

- Low-risk patients will receive their initial doses of 20 and 50 mg venetoclax as outpatients.
- For patients unable to maintain oral hydration at 1.5–2 L/day starting at least 48 hours prior to the start of treatment, IV hydration in the outpatient setting on the day of dosing during the clinic stay is recommended in order to assure that this full amount of hydration is achieved. For patients for whom volume overload is considered a significant risk, hospitalization should be considered.
- Serum chemistry, hematology, and vital signs will be obtained prior to administering venetoclax (predose) and 8 and 24 hours after dosing. Laboratory samples should be sent and analyzed immediately.

Predose is defined as up to 4 hours before venetoclax administration, and results must be reviewed prior to dosing; if it is not possible to review results from a sample taken up to 4 hours predose, then it is acceptable to take a predose hematology and chemistry sample within 24 hours prior to dosing. The results of these samples must be reviewed prior to dosing. If laboratory values from this sample have demonstrated no clinically significant abnormalities, the hematology and chemistry samples drawn on the day of venetoclax administration prior to dosing are not required to be reviewed prior to dose administration. However, these predose (0–4 hours prior to dosing) laboratory samples should still be drawn, and these will serve as baseline for later laboratory values when assessing for laboratory evidence of TLS.

The 8-hour chemistry results must be reviewed before the patient leaves the outpatient clinic that day.

Furthermore, the investigator or subinvestigator must review the 24-hour laboratory results prior to dosing on the next day.

Additional laboratory assessments may be performed per investigator discretion.

TLS Medium Risk

- Medium-risk patients who have CrCl ≥ 80 mL/min will receive their initial doses of 20 and 50 mg venetoclax as outpatients. Patients with CrCl < 80 mL/min and/or who have higher tumor burden (defined per the discretion of the investigator) may be handled as High-Risk patients (see the High Risk section for details of hydration, laboratory, etc.).
- In addition to oral hydration stated above, IV hydration (1.5–2 L) will be given in the outpatient setting during the clinic stay. For patients for whom volume overload is considered a significant risk, hospitalization should be considered.
- Serum chemistry, hematology, and vital signs will be obtained prior to administering venetoclax (predose) and 8 and 24 hours after dosing timepoints. Laboratory samples should be sent and analyzed immediately.

Predose is defined as up to 4 hours before venetoclax administration, and results must be reviewed prior to dosing; if it is not possible to review results from a sample taken up to 4 hours predose, then it is acceptable to take a predose hematology and chemistry sample within 24 hours prior to dosing. The results of these samples must be reviewed prior to dosing. If laboratory values from this sample have demonstrated no clinically significant abnormalities, the hematology and chemistry samples drawn on the day of venetoclax administration prior to dosing are not required to be reviewed prior to dose administration. However, these predose (0–4 hours prior to dosing) laboratory samples should still be drawn, and these will serve as baseline for later laboratory values when assessing for laboratory evidence of TLS.

The 8-hour chemistry results must be reviewed before the patient leaves the outpatient clinic that day.

Furthermore, the investigator or subinvestigator must review the 24-hour laboratory results prior to dosing on the next day.

Additional laboratory assessments may be performed per investigator discretion.

TLS High Risk

- High-risk patients will be hospitalized to receive their initial doses of 20 and 50 mg venetoclax. Hospitalization will begin the evening prior to each initial dose of venetoclax and continue for 24 hours after each dose.
- Upon admission, serum chemistry and hematology laboratory samples should be drawn, and IV hydration should be started with a target of 2–3 L per day or as clinically appropriate.
- Rasburicase must be administered per regional standards/institutional guidelines as prophylaxis prior to the first dose of venetoclax for high-risk patients with high uric acid levels at pre-dose (above the local laboratory ULN or Howard et al. [2011] threshold of 8 mg/dL [475.8 μmol/L]). For patients with a contraindication to rasburicase (i.e., glucose-6-phosphate dehydrogenase deficiency), the TLS risk-mitigation plan must be reviewed with the Medical Monitor. Uric acid levels following treatment with rasburicase must be analyzed using specific guidelines described in the following:

  Please note that at room temperature, rasburicase causes enzymatic degradation of the uric acid in blood, plasma, and serum samples, which could potentially result in spuriously low plasma uric acid assay readings. The following special sample handling procedure must be followed to avoid ex vivo uric acid degradation:

  Uric acid must be analyzed in plasma.

  Blood must be collected into prechilled tubes containing heparin anticoagulant. Immediately immerse plasma samples for uric acid measurement in an ice water bath.

  Plasma samples must be prepared by centrifugation in a precooled centrifuge (4°C).

  The plasma must be maintained in an ice water bath and analyzed for uric acid within 4 hours of collection.

- Nephrology (or acute dialysis service) consultation should be considered on admission (per institutional standards or based on investigator discretion) for hospitalized patients to ensure emergency dialysis is available and the appropriate staff is aware and prepared to handle any necessary intervention for TLS. Telemetry should also be considered.
- Serum chemistry, hematology, and vital signs will be obtained prior to administering venetoclax (predose) and at 8, 12, and 24 hours after dosing. These samples are to be sent immediately to the laboratory, and the results must be reviewed promptly by the investigator or subinvestigator.

  Predose is defined as up to 4 hours before venetoclax administration, and results must be reviewed prior to dosing; if it is not possible to review results from a sample taken up to 4 hours predose, then it is acceptable to take a

predose hematology and chemistry sample within 24 hours prior to dosing. The results of these samples must be reviewed prior to dosing. If laboratory values from this sample have demonstrated no clinically significant abnormalities, the hematology and chemistry samples drawn on the day of venetoclax administration prior to dosing are not required to be reviewed prior to dose administration. However, these predose (0–4 hours prior to dosing) laboratory samples should still be drawn, and these will serve as baseline for later laboratory values when assessing for laboratory evidence of TLS.

The 24-hour post-dose laboratory results must be reviewed by the investigator or subinvestigator before the patient leaves the hospital or receives any additional study drug.

Additional laboratory assessments may be performed per investigator discretion.

Subsequent Dose Increases during the Venetoclax Ramp-Up Period 100, 200, and 400 mg Venetoclax

All patients, irrespective of their risk category, must receive the following TLS prophylaxis measures prior to subsequent dose increases of venetoclax:

- Continued administration of an oral uric acid reducer as indicated above.
- Oral hydration consisting of fluid intake of approximately 1.5–2 L/day starting at least 48 hours prior to dosing. IV hydration is encouraged at subsequent dose increases for patients unable to maintain such oral hydration. IV hydration in the outpatient setting on the day of dosing during the clinic stay is recommended in order to assure this full amount of hydration is achieved. For patients for whom volume overload is considered a significant risk, hospitalization should be considered.
- Serum chemistry and hematology laboratory samples must be drawn prior to administering venetoclax (predose). If clinically significant laboratory abnormalities are observed in this laboratory assessment, dose of venetoclax must be delayed until resolution, and management per the protocol, Recommendations for Initial Management of Electrolyte Imbalances and Prevention of Tumor Lysis Syndrome, must be initiated. If needed, patient should receive additional prophylactic treatment prior to the initiation of dosing.

  Predose is defined as up to 4 hours before venetoclax administration, and results must be reviewed prior to dosing; if it is not possible to review results from a sample taken up to 4 hours predose, then it is acceptable to take a predose hematology and chemistry sample within 24 hours prior to dosing. The results of these samples must be reviewed prior to dosing. If laboratory values from this sample have demonstrated no clinically significant abnormalities, the hematology and chemistry samples drawn on the day of venetoclax administration prior to dosing are not required to be reviewed prior to dose administration. However, these predose (0 to 4 hours prior to dosing) laboratory samples should still be drawn, and these will serve as baseline for later laboratory values when assessing for laboratory evidence of TLS.

Additional TLS prophylaxis and monitoring procedures are tailored to the individual TLS risk category as follows.

TLS Low Risk

- Low-risk patients will receive the subsequent dose increases (100, 200, and 400 mg venetoclax) as outpatients.
- Serum chemistry, hematology, and vital signs will be obtained prior to administering venetoclax (predose) and at 8 and 24 hours after dosing. Laboratory samples should be sent and analyzed immediately.

  Predose is defined as up to 4 hours before venetoclax administration, and results must be reviewed prior to dosing; if it is not possible to review results from a sample taken up to 4 hours predose, then it is acceptable to take a predose hematology and chemistry sample within 24 hours prior to dosing. The results of these samples must be reviewed prior to dosing. If laboratory values from this sample have demonstrated no clinically significant abnormalities, the hematology and chemistry samples drawn on the day of venetoclax administration prior to dosing are not required to be reviewed prior to dose administration. However, these predose (0 to 4 hours prior to dosing) laboratory samples should still be drawn, and these will serve as baseline for later laboratory values when assessing for laboratory evidence of TLS.

  The 8-hour chemistry results must be reviewed before the patient leaves the outpatient clinic that day.

  Furthermore, the investigator or subinvestigator must review the 24-hour laboratory results prior to dosing on the next day.

  Additional laboratory assessments may be performed per investigator discretion.

TLS Medium Risk

- Medium-risk patients who have CrCl $\geq$80 mL/min will receive their subsequent dose increases as outpatient. Patients with CrCl $<$80 mL/min and/or who have high tumor burden (defined per the discretion of the investigator) may be hospitalized.
- For patients who receive this subsequent dose increases as outpatient, serum chemistry, hematology, and vital signs will be obtained prior to administering venetoclax (predose) and at 8 and 24 hours after dosing. Laboratory samples should be sent and analyzed immediately.

  Predose is defined as up to 4 hours before venetoclax administration, and results must be reviewed prior to dosing; if it is not possible to review results from a sample taken up to 4 hours predose, then it is acceptable to take a predose hematology and chemistry sample within 24 hours prior to dosing. The results of these samples must be reviewed prior to dosing. If laboratory values from this sample have demonstrated no clinically significant abnormalities, the hematology and chemistry samples drawn on the day of venetoclax administration prior to dosing are not required to be reviewed prior to dose administration. However, these predose (0 to 4 hours prior to dosing)

laboratory samples should still be drawn, and these will serve as baseline for later laboratory values when assessing for laboratory evidence of TLS.

The 8-hour chemistry results must be reviewed before the patient leaves the outpatient clinic that day.

Furthermore, the investigator or subinvestigator must review the 24-hour laboratory results prior to dosing on the next day.

Additional laboratory assessments may be performed per investigator discretion.

- For patients hospitalized during subsequent dose increases, serum chemistry, hematology, and vital signs will be obtained prior to dosing (predose, defined as up to 4 hours before venetoclax dose) and 8, 12, and 24 hours after dosing. These samples are to be sent immediately to the laboratory, and the results must be reviewed promptly by the investigator or subinvestigator. The 24-hour after dosing laboratory results must be reviewed by the investigator or subinvestigator before the patient leaves the hospital or receives any additional study drug.
- IV hydration should be started with a target of approximately 2–3 L per day or as clinically appropriate for patients who are hospitalized.

TLS High Risk

- High-risk patients with CrCl of ≥ 80 mL/min will receive the subsequent dose increases as outpatients. Patients with CrCl < 80 mL/min and/or high tumor burden (defined per the discretion of the investigator) may be hospitalized. Hospitalization will begin the evening prior to the dose of venetoclax and continue for 24 hours after the dose.
- IV hydration (1.5–2 L) will be given in the outpatient setting during the clinic stay. For patients who are hospitalized, IV hydration should be started with a target of approximately 2–3 L per day or as clinically appropriate.
- For patients who are not hospitalized, serum chemistry, hematology, and vital signs will be obtained prior to administering venetoclax and at 8 and 24 hours after dosing timepoints. Laboratory samples should be sent and analyzed immediately.

  Predose is defined as up to 4 hours before venetoclax administration, and results must be reviewed prior to dosing; if it is not possible to review results from a sample taken up to 4 hours predose, then it is acceptable to take a predose hematology and chemistry sample within 24 hours prior to dosing. The results of these samples must be reviewed prior to dosing. If laboratory values from this sample have demonstrated no clinically significant abnormalities, the hematology and chemistry samples drawn on the day of venetoclax administration prior to dosing are not required to be reviewed prior to dose administration. However, these predose (0 to 4 hours prior to dosing) laboratory samples should still be drawn, and these will serve as baseline for later laboratory values when assessing for laboratory evidence of TLS.

  The 8-hour chemistry results must be reviewed before the patient leaves the outpatient clinic that day.

Furthermore, the investigator or subinvestigator must review the 24-hour laboratory results prior to dosing on the next day.

- For patients who are hospitalized during subsequent dose increases, serum chemistry, hematology, and vital signs will be obtained prior to dosing (predose, defined as up to 4 hours before venetoclax dose) and 8, 12, and 24 hours after dosing. These samples are to be sent immediately to the laboratory, and the results must be reviewed promptly by the investigator or subinvestigator. The 24-hour after dosing laboratory results must be reviewed by the investigator or subinvestigator before the patient leaves the hospital or receives any additional study drug.

  Additional laboratory assessments may be performed per investigator discretion.

Any patient who, at any dose, develops clinically significant electrolyte abnormalities must have his or her subsequent venetoclax dose withheld until the electrolyte abnormalities resolve. Patients who develop electrolyte abnormalities should undergo aggressive management and further monitoring per Appendix 10, Recommendations for Initial Management of Electrolyte Imbalances and Prevention of Tumor Lysis Syndrome. Any time during the ramp-up period, if venetoclax was withheld for 7 days or less, the patient may resume venetoclax at the same dose level or at one lower dose level as determined by the investigator based on a risk assessment (including tumor burden status). The dose must be resumed at one lower dose-level if interruption lasted more than 7 days, with the exception of the initial dose level of 20 mg (400 mg → 200 mg, 200 mg → 100 mg, 100 mg → 50 mg, 50 mg → 20 mg). All patients must receive the intended dose for at least 7 days before increasing to the next ramp-up dose.

For patients who are at high risk of TLS:

- Hospitalized patients should receive TLS prophylaxis as described above for initial venetoclax dosing. Nephrology (or acute dialysis service) must be consulted/contacted on admission (per institutional standards) to ensure emergency dialysis is available and the appropriate staff are aware and prepared to handle any necessary intervention for TLS. Telemetry should also be considered.

5.1.3.2 Prophylaxis for Infections

If clinically indicated, anti-infective prophylaxis for viral, fungal, bacterial, or Pneumocystis infections is permitted. Although there is a potential for drug−drug interactions, there is likely to be limited potential clinical effects, therefore trimethoprim sulfamethoxazole can be considered for Pneumocystis prophylaxis with close clinical monitoring. The Medical Monitor should also be consulted regarding any consideration of the use of azoles as anti-fungal prophylaxis or therapy, because of the potential for drug−drug interactions.

5.1.3.3 Prophylaxis for Infusion-Related Reactions

The rate of infusion of obinutuzumab may need to be slowed for those patients that experience infusion reactions, and medications to treat IRRs (including epinephrine for

subcutaneous injections, corticosteroids, and diphenhydramine for IV injection) and resuscitation equipment should be available for immediate use during dosing.

5.1.3.4 Life-Threatening Infusion-Related Reactions and Anaphylaxis

In the event of a life-threatening IRR (which may include pulmonary or cardiac events) or IgE-mediated anaphylactic reaction, obinutuzumab should be discontinued and no additional obinutuzumab should be administered. Patients who experience any of these reactions should receive aggressive symptomatic treatment and will be discontinued from study treatment.

5.2 SAFETY PARAMETERS AND DEFINITIONS

Safety assessments will consist of monitoring and recording adverse events, including serious adverse events and adverse events of special interest, measurement of protocol-specified safety laboratory assessments, measurement of protocol-specified vital signs, and other protocol-specified tests that are deemed critical to the safety evaluation of the study.

Certain types of events require immediate reporting to the Sponsor, as outlined in Section 5.4.

5.2.1 Adverse Events

According to the ICH guideline for Good Clinical Practice, an adverse event is any untoward medical occurrence in a clinical investigation patient administered a pharmaceutical product, regardless of causal attribution.

An adverse event can therefore be any of the following:

- Any unfavorable and unintended sign (including an abnormal laboratory finding), symptom, or disease temporally associated with the use of a medicinal product, whether or not considered related to the medicinal product
- Any new disease or exacerbation of an existing disease (a worsening in the character, frequency, or severity of a known condition), except as described in Section 5.3.5.10.
- Recurrence of an intermittent medical condition (e.g., headache) not present at baseline
- Any deterioration in a laboratory value or other clinical test (e.g., ECG, X-ray) that is associated with symptoms or leads to a change in study treatment or concomitant treatment or discontinuation from study drug
- Adverse events that are related to a protocol-mandated intervention, including those that occur prior to assignment of study treatment (e.g., screening invasive procedures such as biopsies).

5.2.2 Serious Adverse Events (Immediately Reportable to the Sponsor)

A serious adverse event is any adverse event that meets any of the following criteria:

- Fatal (i.e., the adverse event actually causes or leads to death)
- Life threatening (i.e., the adverse event, in the view of the investigator, places the patient at immediate risk of death)

  This does not include any adverse event that had it occurred in a more severe form or was allowed to continue might have caused death.

- Requires or prolongs inpatient hospitalization (see Section 5.3.5.11).
- Results in persistent or significant disability/incapacity (i.e., the adverse event results in substantial disruption of the patient's ability to conduct normal life functions)
- Congenital anomaly/birth defect in a neonate/infant born to a mother exposed to study drug
- Significant medical event in the investigator's judgment (e.g., may jeopardize the patient or may require medical/surgical intervention to prevent one of the outcomes listed above)

The terms "severe" and "serious" are not synonymous. Severity refers to the intensity of an adverse event (e.g., rated as mild, moderate, or severe, or according to NCI CTCAE criteria; Section 5.3.3); the event itself may be of relatively minor medical significance (such as severe headache without any further findings).

Severity and seriousness need to be independently assessed for each adverse event recorded on the eCRF.

Serious adverse events are required to be reported by the investigator to the Sponsor immediately (i.e., no more than 24 hours after learning of the event; see Section 5.4.2 for reporting instructions).

5.2.3 Adverse Events of Special Interest (Immediately Reportable to the Sponsor)

Adverse events of special interest are required to be reported by the investigator to the Sponsor immediately (i.e., no more than 24 hours after learning of the event; see Section 5.4.2 for reporting instructions).

Adverse events of special interest for this study will include the following:

- Cases of potential drug-induced liver injury that include an elevated ALT or AST in combination with either an elevated bilirubin or clinical jaundice, as defined by Hy's law (see Section 5.3.5.7)

- Suspected transmission of an infectious agent by the study drug, as defined below

  Any organism, virus, or infectious particle (e.g., prion protein transmitting transmissible spongiform encephalopathy), pathogenic or non-pathogenic, is considered an infectious agent. A transmission of an infectious agent may be suspected from clinical symptoms or laboratory findings that indicate an infection in a patient exposed to a medicinal product. This term applies only when a contamination of the study drug is suspected.

5.3 METHODS AND TIMING FOR CAPTURING AND ASSESSING SAFETY PARAMETERS

The investigator is responsible for ensuring that all adverse events (see Section 5.2.1 for definition) are recorded on the Adverse Event eCRF and reported to the Sponsor in accordance with instructions provided in this section and in Sections 5.4, 5.5 and 5.6.

For each adverse event recorded on the Adverse Event eCRF, the investigator will make an assessment of seriousness (see Section 5.2.2 for seriousness criteria), severity (Section 5.3.3) and causality (Section 5.3.4).

5.3.1 Adverse Event Reporting Period

Investigators will seek information on adverse events at each patient contact. All adverse events, whether reported by the patient or noted by study personnel, will be recorded in the patient’s medical record and on the Adverse Event eCRF.

After informed consent has been obtained but prior to initiation of study drug, only adverse events and serious adverse events caused by a protocol-mandated intervention (e.g., invasive procedures such as biopsies, discontinuation of medications) should be reported (see Section 5.4.2 for instructions for reporting serious adverse events).

After initiation of study drug, all adverse events, regardless of relationship to study drug, will be reported until 28 days after the last dose of venetoclax, chlorambucil, or obinutuzumab. Grade 3–4 adverse events should be reported for 6 months, and major infections (Grade 3–4) should be reported throughout the study and for 2 years after the last dose of study treatment, irrespective of causality (see Appendix 14 for duration and reporting of adverse events).

Serious Adverse Events

Throughout the study (including follow-up), all serious adverse events (regardless of causality) will be documented in the eCRF. Details regarding the reporting period for SAEs are found in Appendix 14.

5.3.2 Eliciting Adverse Event Information

A consistent methodology of non-directive questioning should be adopted for eliciting adverse event information at all patient evaluation timepoints.

Examples of non-directive questions include the following:

"How have you felt since your last clinic visit?"

"Have you had any new or changed health problems since you were last here?"

5.3.3 Assessment of Severity of Adverse Events

The adverse event severity grading scale for the NCI CTCAE (v4.03) will be used for assessing adverse event severity. Table 10 and Appendix 9 will be used for assessing severity for adverse events that are not specifically listed in the NCI CTCAE.

Table 10 Adverse Event Severity Grading Scale for Events Not Specifically Listed in NCI CTCAE

Grade	Severity
1	Mild; asymptomatic or mild symptoms; clinical or diagnostic observations only; or intervention not indicated
2	Moderate; minimal, local, or non-invasive intervention indicated; or limiting age-appropriate instrumental activities of daily living [a]
3	Severe or medically significant, but not immediately life-threatening; hospitalization or prolongation of hospitalization indicated; disabling; or limiting self-care activities of daily living [b,c]
4	Life-threatening consequences or urgent intervention indicated [d]
5	Death related to adverse event [d]

CTCAE = Common Terminology Criteria for Adverse Events; NCI = National Cancer Institute.

[a] Instrumental activities of daily living refer to preparing meals, shopping for groceries or clothes, using the telephone, managing money, etc.

[b] Examples of self-care activities of daily living include bathing, dressing and undressing, feeding oneself, using the toilet, and taking medications, as performed by patients who are not bedridden.

[c] If an event is assessed as a "significant medical event," it must be reported as a serious adverse event (see Section 5.4.2 for reporting instructions), per the definition of serious adverse event.

[d] Grade 4 and 5 events must be reported as serious adverse events (see Section 5.4.2 for reporting instructions), per the definition of serious adverse event in Section 5.4.2.

5.3.4 Assessment of Causality of Adverse Events

Investigators should use their knowledge of the patient, the circumstances surrounding the event, and an evaluation of any potential alternative causes to determine whether or not an adverse event is considered to be related to the study drug, indicating "yes" or "no" accordingly.

The following guidance should be taken into consideration:

- Temporal relationship of event onset to the initiation of study drug

- Course of the event, considering especially the effects of dose reduction, discontinuation of study drug, or reintroduction of study drug (as applicable)
- Known association of the event with the study drug or with similar treatments
- Known association of the event with the disease under study
- Presence of risk factors in the patient or use of concomitant medications known to increase the occurrence of the event
- Presence of non–treatment-related factors that are known to be associated with the occurrence of the event

For patients receiving combination therapy, causality will be assessed individually for each protocol-mandated therapy.

5.3.5 Procedures for Recording Adverse Events

Investigators should use correct medical terminology/concepts when recording adverse events on the Adverse Event eCRF. Avoid colloquialisms and abbreviations.

Only one adverse event term should be recorded in the event field on the Adverse Event eCRF.

5.3.5.1 Infusion-Related Reactions

Adverse events that occur during or within 24 hours after obinutuzumab administration should be captured as diagnosis of IRR on an Adverse Event eCRF.

5.3.5.2 Diagnosis versus Signs and Symptoms

A diagnosis (if known) should be recorded on the Adverse Event eCRF rather than individual signs and symptoms (e.g., record only liver failure or hepatitis rather than jaundice, asterixis, and elevated transaminases). However, if a constellation of signs and/or symptoms cannot be medically characterized as a single diagnosis or syndrome at the time of reporting, each individual sign or symptom should be recorded on the Adverse Event eCRF. If a diagnosis is subsequently established, all previously reported signs and symptoms should be nullified and replaced by a single diagnosis, with a starting date that corresponds to the starting date of the first symptom of the eventual diagnosis.

5.3.5.3 Adverse Events That Are Secondary to Other Events

In general, adverse events that are secondary to other events (e.g., cascade events or clinical sequelae) should be identified by their primary cause, with the exception of severe or serious secondary events. A medically significant secondary adverse event that is separated in time from the initiating event should be recorded as an independent event on the Adverse Event eCRF. For example:

- If vomiting results in mild dehydration with no additional treatment in a healthy adult, only vomiting should be reported on the eCRF.

- If vomiting results in severe dehydration, both events should be reported separately on the eCRF.
- If a severe gastrointestinal hemorrhage leads to renal failure, both events should be reported separately on the eCRF.
- If dizziness leads to a fall and consequent fracture, all three events should be reported separately on the eCRF.
- If neutropenia is accompanied by an infection, both events should be reported separately on the eCRF.

All adverse events should be recorded separately on the Adverse Event eCRF if it is unclear as to whether the events are associated.

5.3.5.4 Persistent or Recurrent Adverse Events

A persistent adverse event is one that extends continuously, without resolution, between patient evaluation timepoints. Such events should only be recorded once on the Adverse Event eCRF. The initial severity (intensity) of the event will be recorded at the time the event is first reported. If a persistent adverse event becomes more severe, the most extreme intensity should also be recorded on the Adverse Event eCRF. If the event becomes serious, it should be reported to the Sponsor immediately (i.e., no more than 24 hours after learning that the event became serious; see Section 5.4.2 for reporting instructions). The Adverse Event eCRF should be updated by changing the event from "non-serious" to "serious," providing the date that the event became serious, and completing all data fields related to serious adverse events.

A recurrent adverse event is one that resolves between patient evaluation timepoints and subsequently recurs. Each recurrence of an adverse event should be recorded as a separate event on the Adverse Event eCRF.

5.3.5.5 Abnormal Laboratory Values

Not every laboratory abnormality qualifies as an adverse event. A laboratory test result must be reported as an adverse event if it meets any of the following criteria:

- Accompanied by clinical symptoms
- Results in a change in study treatment (e.g., dosage modification, treatment interruption, or treatment discontinuation)
- Results in a medical intervention (e.g., potassium supplementation for hypokalemia) or a change in concomitant therapy
- Clinically significant in the investigator’s judgment

In this study, certain abnormal values may not qualify as adverse events. Hematologic parameters should be evaluated as described in Table 2 and Appendix 9. Granulocyte colony-stimulating factor used as prophylaxis would not be considered an adverse event but should be reported as a concomitant medication.

It is the investigator's responsibility to review all laboratory findings. Medical and scientific judgment should be exercised in deciding whether an isolated laboratory abnormality should be classified as an adverse event.

If a clinically significant laboratory abnormality is a sign of a disease or syndrome (e.g., alkaline phosphatase and bilirubin $5 \times$ ULN associated with cholestasis), only the diagnosis (i.e., cholestasis) should be recorded on the Adverse Event eCRF.

If a clinically significant laboratory abnormality is not a sign of a disease or syndrome, the abnormality itself should be recorded on the Adverse Event eCRF, along with a descriptor indicating if the test result is above or below the normal range (e.g., "elevated potassium," as opposed to "abnormal potassium"). If the laboratory abnormality can be characterized by a precise clinical term per standard definitions, the clinical term should be recorded as the adverse event. For example, an elevated serum potassium level of 7 mEq/L should be recorded as "hyperkalemia."

Observations of the same clinically significant laboratory abnormality from visit to visit should not be repeatedly recorded on the Adverse Event eCRF, unless the etiology changes. The initial severity of the event should be recorded, and the severity or seriousness should be updated any time the event worsens.

5.3.5.6 Abnormal Vital Sign Values

Not every vital sign abnormality qualifies as an adverse event. A vital sign result must be reported as an adverse event if it meets any of the following criteria:

- Accompanied by clinical symptoms
- Results in a change in study treatment (e.g., dosage modification, treatment interruption, or treatment discontinuation)
- Results in a medical intervention or a change in concomitant therapy
- Clinically significant in the investigator's judgment

It is the investigator's responsibility to review all vital sign findings. Medical and scientific judgment should be exercised in deciding whether an isolated vital sign abnormality should be classified as an adverse event.

If a clinically significant vital sign abnormality is a sign of a disease or syndrome (e.g., high blood pressure), only the diagnosis (i.e., hypertension) should be recorded on the Adverse Event eCRF.

Observations of the same clinically significant vital sign abnormality from visit to visit should not be repeatedly recorded on the Adverse Event eCRF, unless the etiology changes. The initial severity of the event should be recorded, and the severity or seriousness should be updated any time the event worsens.

5.3.5.7 Abnormal Liver Function Tests

The finding of an elevated ALT or AST ($> 3 \times$ ULN) in combination with either an elevated total bilirubin ($> 2 \times$ ULN) or clinical jaundice in the absence of cholestasis or other causes of hyperbilirubinemia is considered to be an indicator of severe liver injury. Therefore, investigators must report as an adverse event the occurrence of either of the following:

- Treatment-emergent ALT or AST $> 3 \times$ ULN in combination with total bilirubin $> 2 \times$ ULN
- Treatment-emergent ALT or AST $> 3 \times$ ULN in combination with clinical jaundice

The most appropriate diagnosis or (if a diagnosis cannot be established) the abnormal laboratory values should be recorded on the Adverse Event eCRF (see Section 5.3.5.2) and reported to the Sponsor immediately (i.e., no more than 24 hours after learning of the event), either as a serious adverse event or an adverse event of special interest (see Section 5.4.2).

5.3.5.8 Deaths

For this protocol, mortality is an efficacy endpoint. Deaths that occur during the protocol-specified adverse event reporting period (see Section 5.3.1) that are attributed by the investigator solely to progression of CLL should be recorded only on the Study Completion/Early Discontinuation eCRF and not on the Adverse Event eCRF. All other on-study deaths, regardless of relationship to study drug, must be recorded on the Adverse Event eCRF and immediately reported to the Sponsor (see Section 5.4.2). An independent monitoring committee will monitor the frequency of deaths from all causes.

Death should be considered an outcome and not a distinct event. The event or condition that caused or contributed to the fatal outcome should be recorded as the single medical concept on the Adverse Event eCRF. Generally, only one such event should be reported. The term "sudden death" should be used only for the occurrence of an abrupt and unexpected death due to presumed cardiac causes in a patient with or without preexisting heart disease, within 1 hour after the onset of acute symptoms or, in the case of an unwitnessed death, within 24 hours after the patient was last seen alive and stable. If the cause of death is unknown and cannot be ascertained at the time of reporting, "unexplained death" should be recorded on the Adverse Event eCRF. If the cause of death later becomes available (e.g., after autopsy), "unexplained death" should be replaced by the established cause of death.

During survival follow-up, deaths attributed to progression of CLL should be recorded only on the Survival eCRF.

5.3.5.9 Preexisting Medical Conditions

A preexisting medical condition is one that is present at the screening visit for this study. Such conditions should be recorded on the General Medical History and Baseline Conditions eCRF.

A preexisting medical condition should be recorded as an adverse event only if the frequency, severity, or character of the condition worsens during the study. When recording such events on the Adverse Event eCRF, it is important to convey the concept that the preexisting condition has changed by including applicable descriptors (e.g., “more frequent headaches”).

5.3.5.10 Lack of Efficacy or Worsening of Chronic Lymphocytic Leukemia

Events that are clearly consistent with the expected pattern of progression of the underlying disease (such as transformation to more aggressive histology) should not be recorded as adverse events. These data will be captured as efficacy assessment data only. If there is any uncertainty as to whether an event is due to disease progression, it should be reported as an adverse event.

5.3.5.11 Hospitalization or Prolonged Hospitalization

Any adverse event that results in hospitalization (i.e., in-patient admission to a hospital) or prolonged hospitalization should be documented and reported as a serious adverse event (per the definition of serious adverse event in Section 5.2.2), except as outlined below.

The following hospitalization scenarios are not considered to be serious adverse events:

- Hospitalization for respite care
- Planned hospitalization required by the protocol (e.g., for monitoring for potential TLS)
- Hospitalization for a preexisting condition, provided that all of the following criteria are met:
    - The hospitalization was planned prior to the study or was scheduled during the study when elective surgery became necessary because of the expected normal progression of the disease
    - The patient has not experienced an adverse event
- Hospitalization due solely to progression of the underlying cancer

The following hospitalization scenarios are not considered to be serious adverse events, but should be reported as adverse events instead:

- Hospitalization that was necessary because of patient requirement for outpatient care outside of normal outpatient clinic operating hours that is required per protocol or per local standard of care.

5.3.5.12 Adverse Events Associated with an Overdose or Error in Drug Administration

An overdose is the accidental or intentional use of a drug in an amount higher than the dose being studied. An overdose or incorrect administration of study treatment is not itself an adverse event, but it may result in an adverse event. All adverse events

associated with an overdose or incorrect administration of study drug should be recorded on the Adverse Event eCRF. If the associated adverse event fulfills serious criteria, the event should be reported to the Sponsor immediately (i.e., no more than 24 hours after learning of the event; Section 5.4.2).

No safety data related to overdosing of venetoclax are available.

5.3.5.13 Patient-Reported Outcome Data

Instructions on the PRO questionnaires in paper format will include a disclaimer to let patients know that the site staff will not be reviewing the answers to the questionnaire and therefore patients should alert the site staff about any problems experienced. Site staff will review PRO questionnaires for completeness only. If it's noted that the patient has written any words on the PRO instrument that is not a pre-defined response (e.g., comments in the margin of the questionnaire, comments in an open text field) that could be an adverse event or serious adverse event, site staff will alert the investigator, who will determine if the criteria for an adverse event have been met and will document the outcome of this assessment in the patient's medical record per site practice. If the event meets the criteria for an adverse event, it will be reported on the Adverse Event eCRF.

5.4 IMMEDIATE REPORTING REQUIREMENTS FROM INVESTIGATOR TO SPONSOR

Certain events require immediate reporting to allow the Sponsor to take appropriate measures to address potential new risks in a clinical trial. The investigator must report such events to the Sponsor immediately; under no circumstances should reporting take place more than 24 hours after the investigator learns of the event. The following is a list of events that the investigator must report to the Sponsor within 24 hours after learning of the event, regardless of relationship to study drug:

- Serious adverse events
- Adverse events of special interest
- Pregnancies

The investigator must report new significant follow-up information for these events to the Sponsor immediately (i.e., no more than 24 hours after becoming aware of the information). New significant information includes the following:

- New signs or symptoms or a change in the diagnosis
- Significant new diagnostic test results
- Change in causality based on new information
- Change in the event's outcome, including recovery
- Additional narrative information on the clinical course of the event

Investigators must also comply with local requirements for reporting serious adverse events to the local health authority and IRB/EC.

5.4.1 Emergency Medical Contacts

Medical Monitor Contact Information for All Sites

Medical Monitor: Maneesh Tandon, MB ChB

Telephone No.: +44 (0)1707 366 344

Mobile Telephone No.: +44 (0)7825 845 332

To ensure the safety of study patients, an Emergency Medical Call Center Help Desk will access the Roche Medical Emergency List, escalate emergency medical calls, provide medical translation service (if necessary), connect the investigator with a Roche Medical Monitor, and track all calls. The Emergency Medical Call Center Help Desk will be available 24 hours per day, 7 days per week. Toll-free numbers for the Help Desk and Medical Monitor contact information will be distributed to all investigators (see “Protocol Administrative and Contact Information & List of Investigators”).

5.4.2 Reporting Requirements for Serious Adverse Events and Adverse Events of Special Interest

Adverse events of special interest for this study will include the following (Appendix 14):

- Cases of potential drug-induced liver injury that include an elevated ALT or AST in combination with either an elevated bilirubin or clinical jaundice, as defined by Hy’s law (see Section 5.3.5.7)
- Suspected transmission of an infectious agent by the study drug, as defined below

5.4.2.1 Events That Occur prior to Study Drug Initiation

After informed consent has been obtained but prior to initiation of study drug, only serious adverse events caused by a protocol-mandated intervention should be reported. A paper Serious Adverse Event Reporting Form and fax cover sheet should be completed and faxed to Roche and AbbVie Safety Risk Management or its designee immediately (i.e., no more than 24 hours after learning of the event), using the fax numbers provided to investigators (see "Protocol Administrative and Contact Information & List of Investigators").

5.4.2.2 Events That Occur after Study Drug Initiation

After initiation of study drug, all adverse events, regardless of relationship to study drug, will be reported until 28 days after the last dose of venetoclax, chlorambucil, or obinutuzumab. Grades 3 to 5 AEs should be reported for 6 months and major infections (Grades 3 to 5) for 2 years after last dose of study treatment irrespective of causality. In all cases the reporting period will end when the next CLL treatment has been given. Related serious adverse events including all second primary malignancies will be reported indefinitely (Section 5.6). *For further details, see Appendix 14.*

Investigators should record all case details that can be gathered immediately (i.e., within 24 hours after learning of the event) on the Adverse Event eCRF and submit the report

via the EDC system. A report will be generated and sent to AbbVie and Roche Safety Risk Management by the EDC system.

In the event that the EDC system is unavailable, a paper Serious Adverse Event Reporting Form and fax cover sheet should be completed and faxed to Roche and AbbVie Safety Risk Management or its designee immediately (i.e., no more than 24 hours after learning of the event), using the fax numbers provided to investigators (see "Protocol Administrative and Contact Information & List of Investigators"). Once the EDC system is available, all information will need to be entered and submitted via the EDC system.

Instructions for reporting post-study adverse events are provided in Section 5.6.

5.4.3 Reporting Requirements for Pregnancies

5.4.3.1 Pregnancies in Female Patients

Female patients of childbearing potential will be instructed to immediately inform the investigator if they become pregnant during the study or within 28 days after the last dose of venetoclax (or within 18 months of the last dose of obinutuzumab). A Pregnancy Report eCRF should be completed by the investigator immediately (i.e., no more than 24 hours after learning of the pregnancy) and submitted via the EDC system. A pregnancy report will automatically be generated and sent to AbbVie and Roche Safety Risk Management. In the event that the EDC system is unavailable, a Clinical Trial Pregnancy Reporting Form and fax cover sheet should be completed and faxed to AbbVie and Roche Safety Risk Management or its designee immediately (i.e., no more than 24 hours after learning of the pregnancy), using the fax numbers provided to investigators. Once the EDC system is available, all information will need to be entered and submitted via the EDC system.

Pregnancy should not be recorded on the Adverse Event eCRF. The investigator should discontinue study drug and counsel the patient, discussing the risks of the pregnancy and the possible effects on the fetus. Monitoring of the patient should continue until conclusion of the pregnancy. Any serious adverse events associated with the pregnancy (e.g., an event in the fetus, an event in the mother during or after the pregnancy, or a congenital anomaly/birth defect in the child) should be reported on the Adverse Event eCRF.

5.4.3.2 Pregnancies in Female Partners of Male Patients

Male patients will be instructed through the Informed Consent Form to immediately inform the investigator if their partner becomes pregnant during the study or within 28 days after completing treatment with venetoclax or 18 months after the last dose of obinutuzumab. Male patients who received study treatment should not attempt to father a child for at least 90 days after the last dose of venetoclax or 18 months after the last dose of obinutuzumab, whichever is longer, and agree to refrain from donating sperm during the treatment period and for at least 90 days after the last dose of venetoclax. A

Pregnancy Report eCRF should be completed by the investigator immediately (i.e., no more than 24 hours after learning of the pregnancy) and submitted via the EDC system. Attempts should be made to collect and report details of the course and outcome of any pregnancy in the partner of a male patient exposed to study drug. The pregnant partner will need to sign an Authorization for Use and Disclosure of Pregnancy Health Information to allow for follow-up on her pregnancy. Once the authorization has been signed, the investigator will update the Pregnancy Report eCRF with additional information on the course and outcome of the pregnancy. An investigator who is contacted by the male patient or his pregnant partner may provide information on the risks of the pregnancy and the possible effects on the fetus, to support an informed decision in cooperation with the treating physician and/or obstetrician.

In the event that the EDC system is unavailable, follow reporting instructions provided in Section 5.4.3.1.

5.4.3.3 Abortions

Any abortion should be classified as a serious adverse event (as the Sponsor considers abortions to be medically significant), recorded on the Adverse Event eCRF, and reported to the Sponsor immediately (i.e., no more than 24 hours after learning of the event; Section 5.4.2).

5.4.3.4 Congenital Anomalies/Birth Defects

Any congenital anomaly/birth defect in a child born to a female patient exposed to study drug or the female partner of a male patient exposed to study drug should be classified as a serious adverse event, recorded on the Adverse Event eCRF, and reported to the Sponsor immediately (i.e., no more than 24 hours after learning of the event; see Section 5.4.2).

5.5 FOLLOW-UP OF PATIENTS AFTER ADVERSE EVENTS

5.5.1 Investigator Follow-Up

The investigator should follow each adverse event until the event has resolved to baseline grade or better, the event is assessed as stable by the investigator, the patient is lost to follow-up, or the patient withdraws consent. Every effort should be made to follow all serious adverse events considered to be related to study drug or trial-related procedures until a final outcome can be reported.

During the study period, resolution of adverse events (with dates) should be documented on the Adverse Event eCRF and in the patient’s medical record to facilitate source data verification. If, after follow-up, return to baseline status or stabilization cannot be established, an explanation should be recorded on the Adverse Event eCRF.

All pregnancies reported during the study should be followed until pregnancy outcome. If the EDC system is not available at the time of pregnancy outcome, follow reporting instructions provided in Section 5.4.3.1.

5.5.2 Sponsor Follow-Up

For serious adverse events, non-serious adverse events, and pregnancies, the Sponsor or a designee may follow up by telephone, fax, electronic mail, and/or a monitoring visit to obtain additional case details and outcome information (e.g., from hospital discharge summaries, consultant reports, autopsy reports) in order to perform an independent medical assessment of the reported case.

5.6 POST-STUDY ADVERSE EVENTS

If the investigator becomes aware of a serious adverse event with a suspected causal relationship to the study drug that occurs after the end of the study in a subject treated by him or her, the investigator should report the serious adverse event to the Sponsor.

The investigator should report these events directly to AbbVie and Roche Safety Risk Management or designee via telephone, email or via fax machine using the Serious Adverse Event Reporting Form and fax cover sheet and via Email or eFax to AbbVie (see “Protocol Administrative and Contact Information & List of Investigators”).

5.7 EXPEDITED REPORTING TO HEALTH AUTHORITIES, INVESTIGATORS, INSTITUTIONAL REVIEW BOARDS, AND ETHICS COMMITTEES

The Sponsor will promptly evaluate all serious adverse events and non-serious adverse events against cumulative product experience to identify and expeditiously communicate possible new safety findings to investigators, IRBs, ECs, and applicable health authorities based on applicable legislation.

To determine reporting requirements for single adverse event cases, the Sponsor will assess the expectedness of these events using the following reference documents:

- Combined Venetoclax /Obinutuzumab Investigator's Brochure
- SmPC for chlorambucil

The Sponsor will compare the severity of each event and the cumulative event frequency reported for the study with the severity and frequency reported in the applicable reference document.

Reporting requirements will also be based on the investigator's assessment of causality and seriousness, with allowance for upgrading by the Sponsor as needed.

Certain adverse events are anticipated to occur in the study population at some frequency independent of study drug exposure and will be excluded from expedited reporting. These anticipated events include known consequences of the underlying disease or condition under investigation (e.g., symptoms, disease progression) and events unlikely to be related to the underlying disease or condition under investigation but common in the study population independent of drug therapy (e.g., cardiovascular events in an elderly population). These events are listed in Appendix 11.

These adverse events may occur alone or in various combinations and are considered expected for reporting purposes for this protocol.

The list of Medical Dictionary for Regulatory Activities (MedDRA) preferred terms associated with these events, is available upon request.

Although exempted from expedited reporting to Health Authorities and IRBs as individual cases, these Serious Adverse Events (as defined in Section 5.4.2) must be reported within 24 hours of the site being made aware of the event.

The iDMC will monitor the safety events in this study. Any recommendations made by the iDMC during the study that do not favor the test product will be submitted to the health authorities.

6. STATISTICAL CONSIDERATIONS AND ANALYSIS PLAN

A detailed statistical analysis plan (SAP) will specify in more detail analyses that are outlined in this section of the protocol.

6.1 DETERMINATION OF SAMPLE SIZE

The sample size for the study is determined given the requirements to perform a hypothesis test for clinically relevant statistical superiority in the primary endpoint of PFS.

Estimates of the number of events required to demonstrate efficacy with regard to PFS are based on the following assumptions:

- Log-rank test at the two-sided 0.05 level of significance
- Median PFS for obinutuzumab and chlorambucil control arm (27 months)
- 80% power to detect HR = 0.65 for the comparison of obinutuzumab + venetoclax experimental arm versus GClb, with median PFS for obinutuzumab + venetoclax increased to 41.5 months
- Exponential distribution of PFS
- Annual drop-out rate of 10%
- One interim analysis for efficacy after 75% of PFS events, utilizing a stopping boundary according to the γ family error spending function with parameter $\gamma = -9.21$.

The addition of an optional early interim analysis requires no adjustment to the sample size, as the impact on the statistical power calculation is negligible.

Based on these assumptions, a total of 170 PFS events are required for the final analysis of PFS.

The minimum detectable difference at the final analysis corresponds approximately to an HR = 0.74.

The sample size calculation was performed using EAST version 6.2.

6.2 SUMMARIES OF CONDUCT OF STUDY

Enrollment, major protocol deviations, study drug administration, and patient disposition will be summarized by treatment arm in all randomized patients. A summary of patient disposition will include whether treatment was completed or discontinued early and the reason for early treatment discontinuation. Descriptive statistics will be used in evaluating the conduct of the study.

6.3 SUMMARIES OF TREATMENT GROUP COMPARABILITY

Demographic and baseline characteristics, such as age, sex, race/ethnicity, and baseline ECOG performance status, will be summarized by treatment arm in all randomized patients. Descriptive statistics will be used to evaluate treatment group comparability.

6.4 EFFICACY ANALYSES

The primary and secondary efficacy analyses will include all randomized patients, with patients grouped according to the treatment assigned at randomization (ITT population).

6.4.1 Primary Efficacy Endpoint

The primary efficacy endpoint is investigator-assessed PFS, defined as the time from randomization to the first occurrence of progression or relapse (determined using standard IWCLL guidelines [2008]), or death from any cause, whichever occurs first. Progression-free survival on the basis of independent review committee (IRC) assessments will be considered primary for U.S. regulatory purposes (details will be provided in the SAP). For patients who have not progressed, relapsed, or died at the time of analysis, PFS will be censored on the date of the last disease assessment. If no disease assessments were performed after the baseline visit, PFS will be censored at the time of randomization + 1 day. All patients, including patients who discontinue all components of study therapy prior to disease progression (e.g., for toxicity), will continue in the study and will be followed for progressive disease and survival regardless of whether or not they subsequently receive new anti-leukemic therapy.

The primary objective of the study is to test the following hypothesis:

- Progression-free survival of obinutuzumab + venetoclax versus GClb (i.e.,
  H0: obinutuzumab + venetoclax = GClb versus
  H1: obinutuzumab + venetoclax ≠ GClb)

Treatment comparisons will be made using a two-sided log-rank test (at 0.05 significance-level, adjusted for the interim *analyses*), stratified by Binet stage. If the null hypothesis is rejected and the observed HR is favorable for the obinutuzumab + venetoclax experimental arm, then it is concluded that obinutuzumab + venetoclax significantly lowers the risk of PFS events more than GClb.

A two-sided non-stratified log-rank test will be performed to support the primary analysis. Median PFS and the 95% confidence limits will be estimated using Kaplan-Meier survival methodology, with the Kaplan-Meier survival curve presented to provide a visual description. PFS rates for 1, 2, and 3 years after randomization with 95% CIs will be reported. Estimates of the treatment effect will be expressed as HR including 95% confidence limits estimated through a Cox proportional-hazards analysis stratified by Binet stage. Primary analysis for FDA submission will be based on assessment of PFS by an Independent Review Committee (IRC).

6.4.2 Secondary Efficacy Endpoints

The secondary endpoints of this study are:

- PFS based on IRC-assessments (primary outcome for U.S. regulatory purposes), defined as the time from randomization to the first occurrence of progression or relapse or death from any cause
- ORR (defined as rate of a clinical response of CR, Cri, or PR) at the completion of treatment assessment, as determined by the investigator according to the IWCLL guidelines (2008)
- *Complete response rate (CRR; defined as rate of a clinical response of CR or CRi) at the completion of treatment assessment, as determined by the investigator according to the IWCLL guidelines (2008)*
- MRD response rate (determined as the proportion of patients with MRD-negativity) measured in the peripheral blood at the completion of treatment assessment and also MRD response rate as measured in the bone marrow at the completion of treatment, both measured by ASO-PCR
- OS, defined as the time between the date of randomization and the date of death due to any cause. Patients who were not reported as having died at the time of the analysis will be censored at the date when they were last known to be alive.
- MRD response rates in the peripheral blood at completion of combination treatment assessment (Cycle 9, Day 1 or 3 months after last IV infusion) and also MRD response rate as measured in the bone marrow, both measured by ASO-PCR
- ORR at completion of combination treatment response assessment (Cycle 7, Day 1 or 28 days after last IV infusion)
- Duration of response, defined as the time from the first occurrence of a documented OR (CR, CRi or PR) to the time of progressive disease as determined by the investigator, or death from any cause
- Best response achieved (CR, CRi, PR, stable disease, or PD) up to and including the assessment at completion of treatment assessment (within 3 months of last day of treatment)
- EFS, defined as the time between date of randomization and the date of disease progression/relapse as assessed by the investigator, death, or start of a new anti-leukemic therapy. Patients without an EFS event will be will be censored on the date of the last disease assessment.

- Time to new anti-leukemic treatment, defined as the time between the date of randomization and the date of first intake of new anti-leukemic therapy. Patients who have not taken new anti-leukemic therapy will be censored at their last assessment prior to the analysis or date of death.

The above secondary outcome measures of MRD will be on the basis of assessment performed centrally by ASO-PCR with MRD negativity defined using a cutoff of 10^{-4} (less than 1 cell in 10,000 leukocytes).

MRD response rate will further be evaluated at completion of treatment using results of both peripheral blood and bone marrow where a patient will be considered MRD positive if either blood or bone marrow result is positive or both are missing. A cross-tabulation will be provided of MRD status in peripheral blood and in bone marrow in patients with both samples available.

MRD response rates at other timepoints during treatment and follow-up where MRD is measured in the peripheral blood (see Schedule of Assessments [Appendix 2 and Appendix 3]) will also be summarized. Further details regarding MRD analyses will be provided in the SAP.

Secondary time-to-event endpoints will be analyzed using the same statistical methods described for the primary analysis of PFS.

Sensitivity analyses for PFS utilizing different censoring mechanism (such as for patients withdrawing due to toxicity) will be performed to assess the robustness of the primary analysis of PFS. These will be described in the SAP.

Response rates in the treatment groups will be compared using Cochran-Mantel-Haenszel test stratified by Binet stage. Rates and 95% CIs will be reported for each treatment group.

Type 1 error control for statistical testing of selected secondary endpoints will be specified in the SAP.

6.5 SAFETY ANALYSES

Safety endpoints include adverse events, serious adverse events, and adverse events of special interest. The safety analyses will include all randomized patients who received at least one dose of study treatment (venetoclax, obinutuzumab, or chlorambucil), with patients grouped according to the treatment actually received.

Treatment exposure will be summarized, including the number of cycles received by each patient, and the cumulative dose will be summarized by treatment arm. Verbatim descriptions of adverse events will be mapped to MedDRA thesaurus terms. All adverse events occurring during or after the first treatment will be summarized by treatment arm and NCI CTCAE grade. In addition, all serious adverse events will be summarized.

Deaths reported during the study treatment period and those reported after treatment completion/discontinuation will be summarized by treatment arm.

Adverse events leading to early treatment discontinuation and early study withdrawal will be summarized by arm and reason. Laboratory data with values outside of the normal ranges will be identified. Additionally, select laboratory data will be summarized by treatment arm and grade using the NCI CTCAE. Of note, abnormal laboratory data that are clinically significant will be reported as adverse events and summarized in the adverse event tables. Vital signs and other physical findings will be summarized by treatment arm.

6.6 PHARMACODYNAMIC ANALYSES

For each visit at which $CD19^{+}$ $CD5^{+}$ and $CD19^{+}$ $CD5^{-}$ B-cell measurements are taken, B-cell data will be listed for individual patients by treatment arm:

- Absolute counts
- Percent relative to the baseline counts for the individual
- Extent of CD19+B-cell depletion (nadir)
- Duration of depletion
- Time to Recovery

$CD19^{+}$ B-cell measurements will be summarized for each visit by the three treatment arms. The parameters summarized will be $CD19^{+}$ B-cell counts, percentage of baseline counts, nadir, time to nadir, duration of depletion, and time to recovery. Exploratory analyses will be performed to assess the possible relationship between the pharmacokinetics of obinutuzumab and B-cell depletion.

6.7 PHARMACOKINETIC ANALYSES

PopPK methods will be used to characterize the pharmacokinetics of venetoclax and obinutuzumab in this study in conjunction with appropriate historical data. Potential correlations of exposure with dose, demographics, pharmacodynamic variables, safety, and efficacy outcomes may be explored as warranted by the data. The results from the popPK analysis may be reported separately from the Clinical Study Report.

Concentrations of venetoclax and obinutuzumab may be summarized by timepoint. Additional PK analyses will be conducted as appropriate.

6.8 PATIENT-REPORTED OUTCOME ANALYSES

The secondary PRO endpoints for the study are as follows:

- To compare disease and treatment-related symptoms following treatment with the combination of obinutuzumab+ venetoclax compared with GClb in patients with previously untreated CLL and coexisting medical conditions between arms as measured by MDASI-CLL.

- To evaluate changes in role functioning and health-related quality of life (HRQoL) following treatment with the combination of obinutuzumab + venetoclax compared with GClb in patients with previously untreated CLL and coexisting medical conditions between arms as measured by EORTC QLQ-C30

The analysis of all other scales will be considered an exploratory endpoint.

A repeated measures for mixed models will be used to compare changes from baseline between the arms.

Scoring for the MDASI-CLL and EORTC QLQ-C30 questionnaires will be based on the corresponding user manual. For scales with more than 50% of the constituent items completed, a pro-rated score will be computed that is consistent with the scoring manuals and validation papers. For subscales with less than 50% of the items completed, the subscale will be considered as missing.

Summary statistics of the MDASI-CLL and EORTC QLQ-C30 scales and their changes from baseline will be calculated at each assessment timepoint for both study arms. Analysis details of these patient-reported outcomes will be provided in the SAP.

6.9 HEALTH ECONOMICS ANALYSIS

Health economics data, as assessed by the EQ-5D-3L, will be evaluated for patients with a baseline assessment and at least one post-baseline EQ-5D-3L assessment that generate a score. Scores at baseline and change from baseline scores for each timepoint will be quantified using descriptive statistics.

The results from the health economics data analysis may be reported separately from the Clinical Study Report.

6.10 EXPLORATORY ANALYSES

- The relationship between various baseline markers and clinical outcome parameters including the primary PFS outcome will be assessed in patients from both arms of the study (including but not limited to CLL FISH (17p-, 11q-, 13p-, +12q), IGHV mutation status, p53 mutation status, serum parameters, Bcl-2 expression and other CLL disease markers.
- Exploratory analyses of MRD negativity by timepoint will also be performed using new technologies including flow cytometry and Next-Generation Sequencing with MRD negativity defined using a cutoff of 10^{-4} (less than 1 cell in 10,000 leukocytes) for comparison with ASO-PCR and secondly by the limit of sensitivity of each of the above technologies.

Also, exploratory analyses will be performed, including graphical analyses, of the relationship between MRD (on the basis of peripheral blood results by ASO-PCR) and PFS.

6.11 INTERIM ANALYSES

In addition to the periodic safety data reviews, the iDMC will evaluate efficacy and safety at *the* formal interim *analysis* of PFS and recommend if the study should be stopped early for efficacy (Section 9.4.2).

Summaries and analyses will be prepared by an independent data coordinating center and presented by treatment arm for the iDMC's review.

An interim efficacy analysis may be conducted at or after 1 year after the last patient's last venetoclax dose (i.e., Month 37 of the study [August 2018]), provided that at least 85 PFS events (50% of the total of 170 PFS events) have occurred. If 85 PFS events have not been observed by 1 year after the last patient's last venetoclax dose, then the interim analysis may be conducted once a minimum of 85 PFS events have occurred. PFS will be tested at the significance level determined using the gamma family error spending function with parameter γ*=–21.12 so that the overall two-sided type I error rate will be maintained at the 0.05 level. This gamma family boundary only allows the study to stop for efficacy if a treatment effect HR of 0.35 or better is observed when the interim analysis is based on 85 events.*

Because this option is added to mitigate a potential delay in the study read-out, the Sponsor's decision to conduct this interim analysis will be based on a number of factors including, but not limited to, the number of events observed by 1 year after the last patient's last venetoclax dose and the subsequent predicted time to reach 110 events. If the Sponsor does not conduct this interim analysis or if this interim analysis is conducted and is negative, then the Sponsor will proceed with the later original interim analysis as follows.

Provided the above early interim analysis is not done or not passed, theoriginal interim analysis for efficacy will be performed once *a minimum of* 110 PFS events have occurred. PFS will be tested at the significance level determined using the gamma family error spending function with parameter γ=–9.21 so that the overall two-sided type I error rate will be maintained at the 0.05 level. *If the early interim analysis (at a minimum of 85 events) is performed and is passed, thise later original interim analysis will not be undertaken.*

The final analysis *is designed to be conducted* after 170 events have occurred. The significance level will be adjusted to incorporate the α spent at *either* interim *analysis done*, so that the overall two-sided type I error rate will be maintained at the 0.05 level.

7. DATA COLLECTION AND MANAGEMENT

7.1 DATA QUALITY ASSURANCE

The Sponsor will be responsible for data management of this study, including quality checking of the data. Data entered manually will be collected via EDC through use of

eCRFs. Sites will be responsible for data entry into the EDC system. In the event of discrepant data, the Sponsor will request data clarification from the sites, which the sites will resolve electronically in the EDC system.

The Sponsor will produce an EDC Study Specification document that describes the quality checking to be performed on the data. Central laboratory and other electronic data (i.e., IRC data) will be sent directly to the Sponsor, using the Sponsor's standard procedures to handle and process the electronic transfer of these data.

eCRFs and correction documentation will be maintained in the EDC system's audit trail. System backups for data stored by the Sponsor and records retention for the study data will be consistent with the Sponsor's standard procedures.

Paper PRO questionnaires will be faxed or couriered from the site to a data entry center.

7.2 ELECTRONIC CASE REPORT FORMS

eCRFs are to be completed through use of a Sponsor-designated EDC system. Sites will receive training and have access to a manual for appropriate eCRF completion. eCRFs will be submitted electronically to the Sponsor and should be handled in accordance with instructions from the Sponsor.

All eCRFs should be completed by designated, trained site staff. eCRFs should be reviewed and electronically signed and dated by the investigator or a designee.

At the end of the study, the investigator will receive patient data for his or her site in a readable format on a compact disc that must be kept with the study records. Acknowledgement of receipt of the compact disc is required.

7.3 SOURCE DATA DOCUMENTATION

Study monitors will perform ongoing source data verification to confirm that critical protocol data (i.e., source data) entered into the eCRFs by authorized site personnel are accurate, complete, and verifiable from source documents.

Source documents (paper or electronic) are those in which patient data are recorded and documented for the first time. They include, but are not limited to, hospital records, clinical and office charts, laboratory notes, memoranda, patient-reported outcomes, evaluation checklists, pharmacy dispensing records, recorded data from automated instruments, copies of transcriptions that are certified after verification as being accurate and complete, microfiche, photographic negatives, microfilm or magnetic media, X-rays, patient files, and records kept at pharmacies, laboratories, and medico-technical departments involved in a clinical trial.

Before study initiation, the types of source documents that are to be generated will be clearly defined in the Trial Monitoring Plan. This includes any protocol data to be

entered directly into the eCRFs (i.e., no prior written or electronic record of the data) and considered source data.

Source documents that are required to verify the validity and completeness of data entered into the eCRFs must not be obliterated or destroyed and must be retained per the policy for retention of records described in Section 7.5.

To facilitate source data verification, the investigators and institutions must provide the Sponsor direct access to applicable source documents and reports for trial-related monitoring, Sponsor audits, and IRB/EC review. The study site must also allow inspection by applicable health authorities.

7.4 USE OF COMPUTERIZED SYSTEMS

When clinical observations are entered directly into a study site's computerized medical record system (i.e., in lieu of original hardcopy records), the electronic record can serve as the source document if the system has been validated in accordance with health authority requirements pertaining to computerized systems used in clinical research. An acceptable computerized data collection system allows preservation of the original entry of data. If original data are modified, the system should maintain a viewable audit trail that shows the original data as well as the reason for the change, name of the person making the change, and date of the change.

7.5 RETENTION OF RECORDS

Records and documents pertaining to the conduct of this study and the distribution of IMP, including eCRFs, PRO data, Informed Consent Forms, laboratory test results, and medication inventory records, must be retained by the Principal Investigator for at least 15 years after completion or discontinuation of the study, or for the length of time required by relevant national or local health authorities, whichever is longer. After that period of time, the documents may be destroyed, subject to local regulations.

No records may be disposed of without the written approval of the Sponsor. Written notification should be provided to the Sponsor prior to transferring any records to another party or moving them to another location.

8. ETHICAL CONSIDERATIONS

8.1 COMPLIANCE WITH LAWS AND REGULATIONS

This study will be conducted in full conformance with the ICH E6 guideline for Good Clinical Practice and the principles of the Declaration of Helsinki, or the laws and regulations of the country in which the research is conducted, whichever affords the greater protection to the individual. The study will comply with the requirements of the ICH E2A guideline (Clinical Safety Data Management: Definitions and Standards for Expedited Reporting). Studies conducted in the United States or under a U.S. Investigational New Drug (IND) application will comply with U.S. FDA regulations and

applicable local, state, and federal laws. Studies conducted in the European Union or European Economic Area will comply with the E.U. Clinical Trial Directive (2001/20/EC).

8.2 INFORMED CONSENT

The Sponsor's sample Informed Consent Form (and ancillary sample Informed Consent Forms such as a Child's Assent or Caregiver's Informed Consent Form, if applicable) will be provided to each site. If applicable, it will be provided in a certified translation of the local language. The Sponsor or its designee must review and approve any proposed deviations from the Sponsor's sample Informed Consent Forms or any alternate consent forms proposed by the site (collectively, the "Consent Forms") before IRB/EC submission. The final IRB/EC-approved Consent Forms must be provided to the Sponsor for health authority submission purposes according to local requirements

The Informed Consent Form will contain a separate section that addresses the use of remaining mandatory samples for optional exploratory research. The investigator or authorized designee will explain to each patient the objectives of the exploratory research. Patients will be told that they are free to refuse to participate and may withdraw their specimens at any time and for any reason during the storage period. A separate, specific signature will be required to document a patient's agreement to allow any remaining specimens to be used for exploratory research. Patients who decline to participate will not provide a separate signature.

The Consent Forms must be signed and dated by the patient or the patient's legally authorized representative before his or her participation in the study. The case history or clinical records for each patient shall document the informed consent process and that written informed consent was obtained prior to participation in the study.

The Consent Forms should be revised whenever there are changes to study procedures or when new information becomes available that may affect the willingness of the patient to participate. The final revised IRB/EC-approved Consent Forms must be provided to the Sponsor for health authority submission purposes.

Patients must be re-consented to the most current version of the Consent Forms (or to a significant new information/findings addendum in accordance with applicable laws and IRB/EC policy) during their participation in the study. For any updated or revised Consent Forms, the case history or clinical records for each patient shall document the informed consent process and that written informed consent was obtained using the updated/revised Consent Forms for continued participation in the study.

A copy of each signed Consent Form must be provided to the patient or the patient's legally authorized representative. All signed and dated Consent Forms must remain in each patient's study file or in the site file and must be available for verification by study monitors at any time.

For sites in the United States, each Consent Form may also include patient authorization to allow use and disclosure of personal health information in compliance with the U.S. Health Insurance Portability and Accountability Act of 1996 (HIPAA). If the site utilizes a separate Authorization Form for patient authorization for use and disclosure of personal health information under the HIPAA regulations, the review, approval, and other processes outlined above apply except that IRB review and approval may not be required per study site policies.

8.3 INSTITUTIONAL REVIEW BOARD OR ETHICS COMMITTEE

This protocol, the Informed Consent Forms, any information to be given to the patient, and relevant supporting information must be submitted to the IRB/EC by the Principal Investigator and reviewed and approved by the IRB/EC before the study is initiated. In addition, any patient recruitment materials must be approved by the IRB/EC.

The Principal Investigator is responsible for providing written summaries of the status of the study to the IRB/EC annually or more frequently in accordance with the requirements, policies, and procedures established by the IRB/EC. Investigators are also responsible for promptly informing the IRB/EC of any protocol amendments (Section 9.6).

In addition to the requirements for reporting all adverse events to the Sponsor, investigators must comply with requirements for reporting serious adverse events to the local health authority and IRB/EC. Investigators may receive written IND safety reports or other safety-related communications from the Sponsor. Investigators are responsible for ensuring that such reports are reviewed and processed in accordance with health authority requirements and the policies and procedures established by their IRB/EC, and archived in the site's study file.

8.4 CONFIDENTIALITY

The Sponsor maintains confidentiality standards by coding each patient enrolled in the study through assignment of a unique patient identification number. This means that patient names are not included in data sets that are transmitted to any Sponsor location.

Patient medical information obtained by this study is confidential and may be disclosed to third parties only as permitted by the Informed Consent Form (or separate authorization for use and disclosure of personal health information) signed by the patient, unless permitted or required by law.

Medical information may be given to a patient's personal physician or other appropriate medical personnel responsible for the patient's welfare, for treatment purposes.

Data generated by this study must be available for inspection upon request by representatives of the U.S. FDA and other national and local health authorities, Sponsor monitors, representatives, and collaborators, and the IRB/EC for each study site, as appropriate.

8.5 FINANCIAL DISCLOSURE

Investigators will provide the Sponsor with sufficient, accurate financial information in accordance with local regulations to allow the Sponsor to submit complete and accurate financial certification or disclosure statements to the appropriate health authorities. Investigators are responsible for providing information on financial interests during the course of the study and for 1 year after completion of the study (i.e., 5 years after last patient enrolled)

9. STUDY DOCUMENTATION, MONITORING, AND ADMINISTRATION

9.1 STUDY DOCUMENTATION

The investigator must maintain adequate and accurate records to enable the conduct of the study to be fully documented, including but not limited to the protocol, protocol amendments, Informed Consent Forms, and documentation of IRB/EC and governmental approval. In addition, at the end of the study, the investigator will receive the patient data, including an audit trail containing a complete record of all changes to data.

9.2 PROTOCOL DEVIATIONS

The investigator should document and explain any protocol deviations. The investigator should promptly report any deviations that might have an impact on patient safety and data integrity to the Sponsor and to the IRB/EC in accordance with established IRB/EC policies and procedures.

9.3 SITE INSPECTIONS

Site visits will be conducted by the Sponsor or an authorized representative for inspection of study data, patients' medical records, and eCRFs. The investigator will permit national and local health authorities, Sponsor monitors, representatives, and collaborators, and the IRBs/ECs to inspect facilities and records relevant to this study.

9.4 ADMINISTRATIVE STRUCTURE

9.4.1 Roles and Responsibilities

Roles and responsibilities between GCLLSG, AbbVie Inc., F. Hoffmann-La Roche Ltd, and Covance, Inc. will be described in separate documents.

9.4.2 Independent Data Monitoring Committee

The iDMC will review the safety data once the randomization is opened. A first safety analysis is planned to occur at the earliest of the following being reached:

- 100 patients randomized and treated for 3 months

OR

- 9 months have elapsed since the first patient was randomized.

OR

- a new case of treatment-related death

Before the first safety analysis occurs the iDMC will review safety data approximately every month depending on the rate of initial recruitment into the study. All further iDMC reviews will take place approximately twice per year and the iDMC will review all safety data collected during the study and the results of any interim *analyses* performed.

For each review, the iDMC will be provided with:

- General toxicity (NCI CTCAE): Grade 3 and Grade 4 adverse events, all serious adverse events
- Laboratory data (hematology and biochemistry)
- Any adverse event that requires discontinuation of the study drug
- Patient deaths
- Concomitant medications

Following each meeting, the iDMC will recommend to the Sponsor whether the study may continue according to the protocol, or the iDMC may suggest changes be made to the protocol on the basis of the outcome of the data review. In exceptional cases, the iDMC may recommend stopping the study or closing a treatment arm due to safety reasons or an overwhelming benefit or lack of efficacy.

An Independent Data Coordinating Center (iDCC) that is independent of the Sponsor will prepare analyses for review.

Further details about the definition, the role as well as the responsibility of the iDMC will be provided in a separate document, the iDMC Charter.

9.4.3 Independent Review Committee

Response will be assessed according to the IWCLL guidelines (2008). The investigator assessment of response and progression will be considered primary for all endpoints described in the study, except for the U.S. regulatory purposes, where the primary endpoint will be IRC-assessed PFS. The IRC will be composed of at least three experts (two reviewers and one adjudicator). IRC assessment will be based on peripheral blood counts, bone marrow biopsy results, and reports of physical examination. Further details about the responsibility and procedures of the IRC will be provided in a separate document, the IRC Charter.

9.5 PUBLICATION OF DATA AND PROTECTION OF TRADE SECRETS

The results of this study may be published or presented at scientific meetings. If this is foreseen, the investigator agrees to submit all manuscripts or abstracts to the Sponsor prior to submission. This allows the Sponsor to protect proprietary information and to

provide comments based on information from other studies that may not yet be available to the investigator.

The Sponsor will comply with the requirements for publication of study results. In accordance with standard editorial and ethical practice, the Sponsor will generally support publication of multicenter trials only in their entirety and not as individual center data. In this case, a coordinating investigator will be designated by mutual agreement.

Authorship will be determined by mutual agreement and in line with International Committee of Medical Journal Editors authorship requirements. Any formal publication of the study in which contribution of Sponsor personnel exceeded that of conventional monitoring will be considered as a joint publication by the investigator and the appropriate Sponsor personnel.

Any inventions and resulting patents, improvements, and/or know-how originating from the use of data from this study will become and remain the exclusive and unburdened property of the Sponsor, except where agreed otherwise.

9.6 PROTOCOL AMENDMENTS

Any protocol amendments will be prepared by the Sponsor. Protocol amendments will be submitted to the IRB/EC and to regulatory authorities in accordance with local regulatory requirements.

Approval must be obtained from the IRB/EC and regulatory authorities (as locally required) before implementation of any changes, except for changes necessary to eliminate an immediate hazard to patients or changes that involve logistical or administrative aspects only (e.g., change in Medical Monitor or contact information).

10. REFERENCES

Böttcher S, Ritgen M, Fischer K, et al. Minimal residual disease quantification is an independent predictor of progression-free and overall survival in chronic lymphocytic leukemia: a multivariate analysis from the randomized GCLLSG CLL8 trial. J Clin Oncol 2012;30:980–8.

Calin GA, Dumitru CD, Shimizu M, et al. Frequent deletions and down-regulation of micro- RNA genes miR15 and miR16 at 13q14 in chronic lymphocytic leukemia. Proc Natl Acad Sci 2002;99:15524–9.

Cairo MS and Bishop M. Tumour lysis syndrome: new therapeutic strategies and classification. Br J Haematol 2004;127(1):3–11.

Chen H, Cantor A, Meyer J, et al. Can older cancer patients tolerate chemotherapy? A prospective pilot study. Cancer 2003;97:1107–14.

Chiorazzi N, Rai KR, Ferrarini M. Chronic lymphocytic leukemia. N Engl J Med 2005;352:804–15.

Conwell Y, Forbes NT, Cox C, et al. Validation of a measure of physical illness burden at autopsy: the Cumulative Illness Rating Scale. J Am Geriatr Soc 1993;41:38–41.

Cramer P, Goede V, Jenke P, et al. Impact of different chemotherapy regimen in comorbid patients with advanced chronic lymphocytic leukemia: metaanalysis of two phase-III-trials of the German CLL study group [abstract]. Blood 2006;108:2480.

Dreger P, Schnaiter A, Zenz T, et al. TP53, SF3B1, and NOTCH1 mutations and outcome of allotransplantation for chronic lymphocytic leukemia: six-year follow-up of the GCLLSG CLL3X trial. Blood 2013;121:3284–8

Extermann M, Overcash J, Lyman GH, et al. Comorbidity and functional status are independent in older cancer patients. J Clin Oncol 1998;16:1582–7.

Fayers P, Aaronson N, Bjordal K, et al. on behalf of the EORTC Quality of Life Study Group. The EORTC QLQ-C30 scoring manual: 2nd ed. Brussels: the European Organisation for Research and Treatment of Cancer. 1999:78pp.

Fesik SW. Promoting apoptosis as a strategy for cancer drug discovery. Nat Rev Cancer. 2005;5:876–85.

Fink AM, Böttcher S, Ritgen M, et al. Prediction of poor outcome in CLL patients following first-line treatment with fludarabine, cyclophosphamide and rituximab. Leukemia 2013;27:1949–52.

Firat S, Byhardt RW, Gore E. Co-morbidity and Karnofksy performance score are independent prognostic factors in stage III non-small-cell lung cancer: an institutional analysis of patients treated on four RTOG studies. Radiation Therapy Oncology Group. Int J Radiat Oncol Biol Phys 2002;54:357–64.

Fischer, K, Al-Sawaf O, Fink, A-M, et al. Safety and efficacy of venetoclax and obinutuzumab in patients with previously untreated Chronic Lymphocytic Leukemia (CLL) and coexisting medical conditions: final results of the run-in phase of the randomized CLL14 trial (BO25323) [abstract]. American Society of Hematology 2016: abstract 2054.

Goede V, Cramer P, Busch R, et al. Distribution and impact of co morbidity in chronic lymphocytic leukemia: meta-analysis of two phase III trials of the German CLL Study Group (GCLLSG). Leuk Lymphoma 2005;46:105.

Goede V, Fischer K, Busch R, et al. Obinutuzumab plus chlorambucil in patients with CLL and coexisting conditions. N Engl J Med 2014;370:1101–10.

Hall SF, Rochon PA, Streiner DL, et al. Measuring co morbidity in patients with head and neck cancer. Laryngoscope 2002;112:1988–96.

Hallek M; On behalf of the German CLL Study Group. Prognostic factors in chronic lymphocytic leukemia. Ann Oncol 2008;19(Suppl 4):iv51–3.

Hallek M, Fischer K, Fingerle-Rowson G, et al. Addition of rituximab to fludarabine and cyclophosphamide in patients with chronic lymphocytic leukaemia: a randomised, open-label, phase 3 trial. Lancet 2010;376:1164–74.

Hande KR, Garrow GC. Acute tumor lysis syndrome in patients with high-grade non Hodgkin's lymphoma. Am J Med 1993;94(2):133–9.

Howard SC, Jones DP, Pui CH. The tumor lysis syndrome. New Engl J Med 2011;364:1844–54.

Jemal A, Siegel R, Ward E, et al. Cancer statistics, 2007. CA Cancer J Clin 2007;57:43–66.

Kedar A, Grow W, Neiberger RE. Clinical versus laboratory tumor lysis syndrome in children with acute leukemia. Pediatr Hematol Oncol 1995;12(2):129–34.

Keller BK, Potter JF. Predictors of mortality in outpatient geriatric evaluation and management clinic patients. J Gerontol 1994;49:M246–51.

Kikushige Y, Ishikawa F, Miyamoto T, et al. Self-renewing hematopoietic stem cell is the primary target in pathogenesis of human chronic lymphocytic leukemia. Cancer Cell 2011;20:246–59.

Linn BS, Linn MW, Gurel L. Cumulative Illness Rating Scale. J Am Geriatr Soc 1968;16662–6.

Mauro FR, Foa R, Giannarelli D, et al. Clinical characteristics and outcome of young chronic lymphocytic leukemia patients: a single institution study of 204 cases. Blood 1999;94:448–54.

Mansouri L, Grabowski P, Degerman S, et al. Short telomere length is associated with NOTCH1/SF3B1/TP53 aberrations and poor outcome in newly diagnosed chronic lymphocytic leukemia patients. Am J Hematol 2013;88:647–51.

Matutes E, Owusu-Ankomah K, Morilla R, et al. The immunological profile of B-cell disorders and proposal of a scoring system for the diagnosis of CLL. Leukemia 1994;8:1640–5.

Mérino D, Bouillet P. The Bcl-2 family in autoimmune and degenerative disorders. Apoptosis. 2009 Apr;14:570–83.

Miller MD, Paradis CF, Houck PR, et al. Rating chronic medical illness burden in geropsychiatric practice and research: application of the Cumulative Illness Rating Scale. Psychiatry Res 1992;41:237–48.

Molica S. Sex differences in incidence and outcome of chronic lymphocytic leukemia patients. Leuk Lymphoma 2006;47:1477–80.

Morton LM, Wang SS, Devesa SS, et al. Lymphoma incidence patterns by WHO subtype in the United States, 1992–2001. Blood 2006;107:265–76.

National Cancer Institute. Surveillance, Epidemiology, and End Results Cancer Statistics Review (SEER CSR) [resource on the Internet]. [cited 2010 March 29]. Available from: http://seer.cancer.gov.

Parmelee PA, Thuras PD, Katz IR, et al. Validation of the Cumulative Illness Rating Scale in a geriatric residential population. J Am Geriatr Soc 1995;43:130–7.

Puente XS, Pinyol M, Quesada V, et al. Whole-genome sequencing identifies recurrent mutations in chronic lymphocytic leukaemia. Nature 2011;475:101–5.

Quesada V, Conde L, Villamor N, et al. Exome sequencing identifies recurrent mutations of the splicing factor SF3B1 gene in chronic lymphocytic leukemia. Nat Genet 2011;44:47–52.

Razis E, Arlin ZA, Ahmed T, et al. Incidence and treatment of tumor lysis syndrome in patients with acute leukemia. Acta Haematol 1994;91(4):171–4.

Rozman C, Montserrat E. Chronic lymphocytic leukemia. N Engl J Med 1995;333:1052–7.

Schnaiter A, Paschka P, Rossi M, et al. NOTCH1, SF3B1, and TP53 mutations in fludarabine-refractory CLL patients treated with alemtuzumab: results from the CLL2H trial of the GCLLSG. Blood 2013;122:1266–70.

Souers AJ, Leverson JD, Boghaert ER et al. ABT-199, a potent and selective BCL-2 inhibitor, achieves antitumor activity while sparing platelets. Nat Med. 2013 Feb;19:202–8.

Stilgenbauer S, Schnaiter A, Paschka P, et al. Gene mutations and treatment outcome in chronic lymphocytic leukemia: results from the CLL8 trial. Blood 2014;123:3247–54.

Touzeau C, Dousset C, Le Gouill S, et al. The Bcl-2 specific BH3 mimetic ABT-199: a promising targeted therapy for t(11;14) multiple myeloma. Leukemia 2014;28:210–2.

Vandenberg CJ, Cory S. ABT-199, a new Bcl-2-specific BH3 mimetic, has in vivo efficacy against aggressive Myc-driven mouse lymphomas without provoking thrombocytopenia. Blood 2013;121:2285–8.

Venclexta® (venetoclax) United States Prescribing Information, AbbVie Inc. and Genentech USA Inc. 2016. Available from: http://www.accessdata.fda.gov/drugsatfda_docs/label/2016/208573s000lbl.pdf

Venclyxto® (venetoclax) European Medicines Agency Summary of Product Characteristics, AbbVie Ltd. 2016. Available from: http://www.ema.europa.eu/docs/en_GB/document_library/EPAR_-_Product_Information/human/004106/WC500218800.pdf

Waldman E, Potter JF. A prospective evaluation of the cumulative illness rating scale. Aging (Milano) 1992;4:171–8.

Watson L, Wyld P, Catovsky D. Disease burden of chronic lymphocytic leukemia within the European Union. Eur J Haematol 2008;81:253–8.

Zenz T, Häbe S, Denzel T, et al. Detailed analysis of p53 pathway defects in fludarabine-refractory chronic lymphocytic leukemia (CLL): dissecting the contribution of 17p deletion, TP53 mutation, p53-p21 dysfunction, and miR34a in a prospective clinical trial. Blood 2009;114:2589–97.

Zenz T, Mertens D, Küppers R, et al. From pathogenesis to treatment of chronic lymphocytic leukaemia. Nat Rev Cancer 2010a;10:37–50.

Zenz T, Vollmer D, Trbusek M, et al. TP53 mutation profile in chronic lymphocytic leukemia: evidence for a disease specific profile from a comprehensive analysis of 268 mutations. Leukemia 2010b;24:2072–9.

Appendix 1
Schedule of Assessments Treatment-Period Visits (RAMP-UP to 400 mg)

	Screening	Cycle 1						Cycle 2							
Day	–28 to –1	1 [a]	2	8	15	22 (20 mg)	23	1 (50 mg)	2	8 (100 mg)	9	15 (200 mg)	16	22 (400 mg)	23
Informed Consent [b]	x														
Consent for GCLLSG biobanking (optional) [b]	x														
Demographic data	x														
TLS Lab-Based Risk Assessment and monitoring [c]	x	x	x			x	x	x [d]	x	x	x	x	x	x	x
General medical history, CIRS score, IADL and baseline conditions	x														
Vital signs [e]	x	x	x	x	x	x	x	x		x		x		x	
Weight	x	x						x							
Height	x														
Complete physical examination [f]	x														
Clinical Staging [f]	x														
Lymphadenopathy (during physical exam/Binet staging at screening)	x														

Appendix 1
Schedule of Assessments Treatment Period Visits (RAMP-UP to 400 mg) (cont.)

	Screening	Cycle 1						Cycle 2							
Day	–28 to –1	1 [a]	2	8	15	22 (20 mg)	23	1 (50 mg)	2	8 (100 mg)	9	15 (200 mg)	16	22 (400 mg)	23
Liver/spleen (by physical examination)	x														
ECG 12 lead	x	As clinically indicated													
LVEF	As clinically indicated														
ECOG performance status	x	x						x							
B symptoms	x	x						x							
CT scan assessment [g]	x	If PD is detected and confirmation of CR/PR at 3 months post treatment													
Bone marrow biopsy [h]	(x)														
Hospitalization		(x)				Mandatory hospitalization for patients at high-risk for TLS on Day 1 of 20-mg and 50-mg doses and possibly Day 1 of 100-mg, 200-mg, and 400-mg doses in treatment Arm A only									
Obinutuzumab (Arms A and B) [i]		x	(x)	x	x			x							
Venetoclax Arm A						Daily, as per dosing chart schedule (20, 50, 100, 200, 400mg) for 12 cycles starting C1D22									

Appendix 1
Schedule of Assessments Treatment Period Visits (RAMP-UP to 400 mg) (cont.)

	Screening	Cycle 1						Cycle 2							
Day	–28 to –1	1 [a]	2	8	15	22 (20 mg)	23	1 (50 mg)	2	8 (100 mg)	9	15 (200 mg)	16	22 (400 mg)	23
Chlorambucil Arm B		x			x			x				x			
Concomitant medications	x	x	x	x	x	x	x	x	x	x	x	x	x	x	x
Adverse events [b]	x	x	x	x	x	x	x	x	x	x	x	x	x	x	x
Serious AEs and Grade 3 and 4 infections [b]	x	x	x	x	x	x	x	x	x	x	x	x	x	x	x
PRO questionnaires (MDASI-CLL, EQ-5D-3L, and EORTC-QLQ C30) [t]		x						x							
**Local laboratory** [j]															
Immunoglobulins	x														
HBV, HCV, HTLV1 (if applicable)[k]	x														
Coombs test	x														
Coagulation (aPTT, PT, INR)	x														
Pregnancy test [l]	x	x						x							
Urinalysis [m]	x														
Hematology [n]	x	x	x	x	x	x	x	x	x	x	x	x	x	x	x
Chemistry [o]	x	x	x	x	x	x	x	x	x	x	x	x	x	x	x

Appendix 1
Schedule of Assessments Treatment Period Visits (RAMP-UP to 400 mg) (cont.)

	Screening	Cycle 1						Cycle 2							
Day	–28 to –1	1 [a]	2	8	15	22 (20 mg)	23	1 (50 mg)	2	8 (100 mg)	9	15 (200 mg)	16	22 (400 mg)	23
**Covance Central Laboratory** [j]															
Serum parameters (β2 microglobulin, thymidine kinase)		x[p]													
CLL markers (DNA/RNA)		x[p]													
Flow cytometry (BCL2 family and CLL counts)	x														
Optional tumor tissue for Bcl-2 family analysis by IHC (formalin fixed tissue)		x[q]													
MRD (ASO-PCR) [r]	x														
MRD (NGS)	x														
**Academic Laboratory** [j]															
Evaluation of resistance by CD40L, simulation		x[p,s]													
Metaphase cytogenetics		x[p]													
GCLLSG Biobanking sample		x[s]													

Appendix 1
Schedule of Assessments Treatment Period Visits (RAMP-UP to 400 mg) (cont.)

	Screening	Cycle 1						Cycle 2								
Day	–28 to –1	1 [a]	2	8	15	22 (20 mg)	23	1 (50 mg)	2	8 (100 mg)	9	15 (200 mg)	16	22 (400 mg)	23
Genetic analysis, (IGVH mutation status, cytogenetics (FISH), gene mutations and resistance markers	x														
Blood for lymphocyte immunophenotyping (for diagnosis, safety monitoring, IRR prediction, ZAP70/CD38)	x														
MRD (flow)	x														

AE = adverse event; ASO-PCR = allele-specific oligonucleotide polymerase chain reaction; C = cycle; CIRS = Cumulative Illness Rating Scale; CLL = chronic lymphocytic leukemia; CR = complete response; CT = computed tomography; D = day; ECOG = Eastern Cooperative Oncology Group; eCRF = electronic Case Report Form; EORTC = European Organization for Research and Treatment of Cancer; EQ-5D-3L = EuroQol 5-Dimension questionnaire; GCLLSG = German CLL Study Group; IADL = Instrumental Activity of Daily Living IHC = immunohistochemistry; INR = international normalized ratio; IRR = infusion-related reaction; LVEF = left ventricular ejection fraction; MDASI-CLL = M.D. Anderson Symptom Inventory-Chronic Lymphocytic Leukemia; MRD = minimal residual disease; MRI = magnetic resonance imaging; NGS = next-generation sequencing; PD = disease progression; PR = partial response; PRO = patient-reported outcome; Tmt = treatment; TLS = tumor lysis syndrome.

Notes: All assessments *during treatment period and at the follow-up Day 28 visit* should be performed within 7 days of the scheduled visit, unless otherwise specified. On treatment days, all assessments should be performed prior to dosing, unless otherwise specified. On treatment days: pre-infusion laboratory samples should be drawn 0–4 hours before the start of infusion. On Day 1 of each venetoclax dose ramp-up (e.g., 20 mg [C1D22], 50 mg [C2D1], 100 mg [C2D8], 200 mg [C2D15], 400 mg [C2D22]), laboratory analyses will be required predose and at 8 and 24 hours postdose to ensure that there are no abnormal changes. If the patient is an inpatient, an additional 12 hours post-dose initiation hematology and chemistry is required. Laboratory values must be read by a clinician prior to taking the next venetoclax dose.

[a] Samples should be taken after randomization number has been assigned.

[b] After informed consent has been obtained but prior to initiation of study drug, only adverse events and serious adverse events caused by a protocol-mandated intervention should be reported. After initiation of study drug, all adverse events will be reported until 28 days after the last dose. After this period, the investigator is not required to actively monitor patients for adverse events; however, the Sponsor should be notified if the investigator becomes aware of any post study serious adverse events or adverse events of special interest. The investigator should follow each adverse event until the event has resolved to baseline grade or better, the event is assessed as stable by the investigator, the patient is lost to follow-up, or the patient withdraws consent. Every effort should be made to follow all serious adverse events considered to be related to study drug or trial-related procedures until a final outcome can be reported.

[c] Chemistry and hematology must also be assessed within 0–4 hours prior and 24 hours after the first obinutuzumab and venetoclax dose, and within 0–4 hours prior and 24 hours after each new (increased) venetoclax dose during the venetoclax dose-ramp up phase. If it is not possible to review results from a sample taken up to 4 hours predose, then it is acceptable to take a predose hematology and chemistry sample within 24 hours prior to dosing. The results of these samples must be reviewed prior to dosing. If laboratory values from this sample have demonstrated no clinically significant abnormalities, the hematology and chemistry samples drawn on the day of venetoclax administration prior to dosing are not required to be reviewed prior to dose administration. However, these predose (0–4 hours prior to dosing) laboratory samples should still be drawn, and these will serve as baseline for later laboratory values when assessing for laboratory evidence of TLS.

[d] For Cycle 2 only, in addition to Day 1, the TLS assessment must be performed on Day 8, Day 15, and Day 22.

[e] Includes: pulse rate and systolic and diastolic blood pressure while the patient is in a seated position, and temperature.
High TLS risk subjects must have vital signs performed before and 8, 12, 24 hours after venetoclax 20mg and 50 mg dose ramp up.

[f] Physical examination includes evaluation of the head, eyes, ears, nose, and throat, and the cardiovascular, dermatological, musculoskeletal, respiratory, gastrointestinal, genitourinary, and neurological systems. Record abnormalities observed at baseline on the General Medical History and Baseline Conditions eCRF. At subsequent visits, record new or worsened clinically significant abnormalities on the Adverse Event eCRF. Binet staging will be assessed using data collected at the screening visit. Physical examinations may be performed 1 day prior to a planned treatment visit to facilitate scheduling.

[g] CT Scan allowed 8 weeks prior to randomization and required as confirmation of CR/PR at 3months after end of treatment. MRI only if contrast enhanced CT not possible.

[h] A bone marrow aspirate and biopsy is optional at screening (investigator discretion if clinically indicated) and must be taken at the end of treatment response assessment at the end of combination therapy at 9 months (Cycle 9) in patients with a CR or a PR.

[i] Obinutuzumab may be given over two days if subject is considered high risk for infusion related reaction (IRR),or as per standard of care (SOC).

[j] For samples drawn on days of study treatment, predose laboratory samples should be drawn within 0–4 hours before the dose. Other laboratory samples occurring on the same day should be obtained within a ±15-minute window of any scheduled time. Any laboratory tests occurring at time intervals greater than or equal to 24 hours after dose should be obtained within a ± 2-hour window of the scheduled time.

[k] Certain patients (occult or prior HBV infection) require monthly DNA testing, although the data is not collected for the study.

[l] All women who are not postmenopausal (≥ 12 months of non-therapy-induced amenorrhea) or surgically sterile will have a serum pregnancy test at screening. If serum pregnancy test has not been performed 14 days prior to the first dose, a urine pregnancy test must be performed 7 days prior to the first dose. If a urine pregnancy test is positive, it must be confirmed by a serum pregnancy test. Further, all women who are not postmenopausal (≥ 12 months of non-therapy-induced amenorrhea) or surgically sterile will have a serum pregnancy test monthly while receiving study drug and at the Treatment Completion/Early Termination visit.

[m] Includes: dipstick pH, specific gravity, glucose, protein, ketones, blood) and microscopic examination (sediment, RBCs, WBCs, casts, crystals, epithelial cells, bacteria).

[n] Includes: WBC count, RBC count, hemoglobin, hematocrit, platelet count, differential count (neutrophils, eosinophils, basophils, monocytes, lymphocytes, other cells.

[o] Includes: sodium, potassium, chloride, bicarbonate, glucose, BUN or urea, creatinine, total protein, albumin, phosphorus, calcium, total and direct bilirubin, alkaline phosphatase, ALT, AST, uric acid, LDH.

[p] Sample should be taken after randomization number is assigned and before first dose.

[q] If formalin fixed specimen of bone marrow biopsy (also including lymph node or other biopsies) are collected at baseline by the site as per standard clinical assessment of the patient, a sample should be provided for IHC analysis of Bcl-2 family.

[r] Blood. Additional bone marrow aspirate at completion of combination therapy (Cycle 9, Day 1 or 3 months after last IV infusion) and at the completion of treatment assessment (a minimum of 3 months after last treatment) for patients with CR/CRi and PR.

[s] For patients who have signed the additional consent for GCLLSG biobanking, residual sample will be sent to the GCLLSG for analysis.

[t] PRO questionnaires scheduled for administration during a clinic visit should be completed prior to the performance of non-PRO assessments and the administration of study treatment.

Appendix 2
Schedule of Assessments Cycle 3–Cycle 12 and Follow-Up Day 28

Assessment	C3	C4	C5	C6	C7	C8	C9	C10	C11	C12	Day 28 after Treatment Completion/Early Termination [a]
General medical history											
Vital signs (Day 1 of each cycle only)	x	x	x	x	x	x	x	x	x	x	x
Weight	x	x	x	x	x	x	x	x	x	x	
Complete physical examination		x			x		x				x
Lymphadenopathy (during physical examination)		x			x		x				x
Liver and spleen by physical examination		x			x		x				x
ECG 12 lead	As clinically indicated										
LVEF	As clinically indicated										
ECOG performance status	x	x	x	x	x	x	x	x	x	x	
B-symptoms	x	x	x	x	x	x	x	x	x	x	
CT scan if PD is detected lymph nodes only	x										
Bone marrow aspirate and biopsy (MRD) CR/PR							x				
Obinutuzumab Arm A and B	x	x	x	x							
Chlorambucil Arm B (Days 1 and 15)	x	x	x	x	x	x	x	x	x	x	
Venetoclax Arm A	Daily dosing 400 mg for 10 cycles										
Staging and response assessment		x			x		x				x
CT scan confirmation of CR/PR											

Appendix 2
Schedule of Assessments Cycle 3–Cycle 12 and Follow-Up Day 28 (cont.)

Assessment	C3	C4	C5	C6	C7	C8	C9	C10	C11	C12	Day 28 after Treatment Completion/Early Termination [a]
Concomitant medication	x	x	x	x	x	x	x	x	x	x	x
AEs	x	x	x	x	x	x	x	x	x	x	x
SAEs/Grade3/4 infections	x	x	x	x	x	x	x	x	x	x	x
PRO questionnaires (MDASI-CLL, EQ-5D-3L, EORTC QLQ C30) [b]	x	x	x	x	x	x	x	x	x	x	x
**Local laboratory**											
Hematology (Days 1 and 15)	x	x	x	x	x	x	x	x	x	x	x
Biochemistry (Days 1 and 15)	x	x	x	x	x	x	x	x	x	x	x
Pregnancy Test [c]	x	x	x	x	x	x	x	x	x	x	
**Covance Central Laboratory**											
MRD (ASO-PCR)					x		x			x	
MRD (NGS)					x		x			x	
PK samples for obinutuzumab [d]		x (Arm A only)									
PK samples for venetoclax [d]		x (Arm A only)									
**Academic Laboratory**											
MRD (flow)					x		x			x	
Blood for safety monitoring/*B-cell recovery*											x

Appendix 2
Schedule of Assessments Cycle 3–Cycle 12 and Follow-Up Day 28 (cont.)

AE = adverse event; ASO-PCR = allele-specific oligonucleotide polymerase chain reaction; C = cycle; CR = complete response; CT = computed tomography; ECOG = Eastern Cooperative Oncology Group; EORTC = European Organization for Research and Treatment of Cancer; EQ-5D-3L = EuroQol 5-Dimension questionnaire; IRR = infusion-related reaction; LVEF = left ventricular ejection fraction; MDASI-CLL = M.D. Anderson Symptom Inventory-Chronic Lymphocytic Leukemia; MRD = minimal residual disease; NGS = next-generation sequencing; PD = disease progression; PK = pharmacokinetic; PR = partial response; PRO = patient-reported outcome; SAE = serious adverse event; TLS = tumor lysis syndrome.

Notes: For laboratory samples drawn on days on study treatment, predose laboratory samples should be drawn within 0–4 hours before the dose. Other laboratory samples occurring on the same day should be obtained within a ± 15-minute window of any scheduled time. Any laboratory tests occurring at time intervals greater than or equal to 24 hours after dose should be obtained within a ± 2–hour window of the scheduled time.

[a] Patients who discontinue all study drug prior to completing 12 cycles of treatment should have their early termination visit performed 28 days after the last dose of study drug was administered. Patients who discontinue study drug prior to completing 12 cycles of therapy should continue to be followed for disease progression per the schedule in Appendix 3.

[b] PRO questionnaires scheduled for administration during a clinic visit should be completed prior to the performance of non-PRO assessments and the administration of study treatment.

[c] All women who are not postmenopausal (≥ 12 months of non-therapy-induced amenorrhea) or surgically sterile will have a serum pregnancy test monthly while receiving study drug and at the Treatment Completion/Early Termination visit.

[d] See Appendix 4 for Schedule of PK Assessments.

Appendix 3
Schedule of Assessments (Follow-Up Period)

	Follow-Up Visits<br>Duration from Last Study Drug Administration until Disease Progression			Survival<br>(All Patients)
Assessment	+3 months after Treatment Completion/Early Termination [a]	+6, 9, 12, 15 and18 months after Treatment Completion/Early Termination	+24 months after Treatment Completion/Early Termination then every 6 months until 5 years from last patient enrolled	Every year after disease progression until 5 years from last patient enrolled
Complete physical examination	x	x	x	
Weight	x	x	x	
Vital signs [b]	x	x	x	
ECG 12-lead	As clinically indicated	As clinically indicated	As clinically indicated	
LVEF	As clinically indicated	As clinically indicated	As clinically indicated	
Lymphadenopathy during physical examination [c]	x	x	x	
Liver/spleen (by physical examination)	x	x	x	
ECOG performance status	x	x	x	
B symptoms	x	x	x	
CT scan assessment (or MRI if performed at screening)	x			
CT scan of involved nodes at time of PD if determined by physical examination alone	x	x	x	
Concomitant medications	x	x	x	x
Response assessment	x	x	x	

Appendix 3
Schedule of Assessments (Follow-Up Period) (cont.)

	Follow-Up Visits<br>Duration from Last Study Drug Administration until Disease Progression			Survival<br>(All Patients)
Assessment	+3 months after Treatment Completion/Early Termination [a]	+6, 9, 12, 15 and18 months after Treatment Completion/Early Termination	+24 months after Treatment Completion/Early Termination then every 6 months until 5 years from last patient enrolled	Every year after disease progression until 5 years from last patient enrolled
Follow-up for PD/disease transformation	x	x	x	
After PD continue to follow-up for NLT	x	x	x	x
PRO questionnaire (MDASI-CLL, EQ-5D-3L, and EORTC-QLQ C30) until NLT [d]	x	x	x	
Collection of NLT				x
Grade 3 and 4 adverse events (until 6 months after the end of treatment)	x	x		
Unrelated SAEs	x	x	x	
Grade 3 and 4 infections (until 2 years after the end of treatment)	x	x	x	
Related SAEs and secondary malignancies, indefinitely	x	x	x	x
Hematology [e]	x	x	x	
Chemistry [f]	x	x	x	
Immunoglobulin (IgA, IgG, IgM)	x	x	x	
Urinalysis [g]	x			
GCLLSG biobanking [h]	x (at timepoint of refractory disease or PD only)			

Appendix 3
Schedule of Assessments (Follow-Up Period) (cont.)

	Follow-Up Visits<br>Duration from Last Study Drug Administration until Disease Progression			Survival<br>(All Patients)
Assessment	+3 months after Treatment Completion/Early Termination [a]	+6, 9, 12, 15 and18 months after Treatment Completion/Early Termination	+24 months after Treatment Completion/Early Termination then every 6 months until 5 years from last patient enrolled	Every year after disease progression until 5 years from last patient enrolled
Genetic analysis (IGHV mutation status, Cytogenetics [FISH], gene mutations and resistance markers)	x (at time point of refractory disease or PD only)			
Evaluation of resistance by CD40L stimulation	x (at time point of refractory disease or PD only)			
Flow cytometry (BCL2)	x (at time point of refractory disease or PD only)			
BCL2 family and CLL markers (DNA/RNA)	x (PD only)			
MRD (blood)	x [i]	x [i]	*x* [j]	
Bone Marrow Aspirate	x [k]			
Bone Marrow Biopsy	x [k]			
Blood for safety monitoring/ B-cell recovery [l]		x *(12 and 18 months only)*	x	

AE = adverse event; ASO-PCR = allele-specific oligonucleotide polymerase chain reaction; CLL = chronic lymphocytic leukemia; CR = complete response; CT = computed tomography; ECOG = Eastern Cooperative Oncology Group; eCRF = electronic Case Report Form; EORTC = European Organization for Research and Treatment of Cancer; EQ-5D-3L = EuroQol 5-Dimension questionnaire; FISH = fluorescence in situ hybridization; GCLLSG = German CLL Study Group; MDASI-CLL = M.D. Anderson Symptom Inventory-Chronic Lymphocytic Leukemia; MRD = minimal residual disease; MRI = magnetic resonance imaging; NLT = next anti-leukemia treatment; PD = progressive disease; PK = pharmacokinetic; PR = partial response; PRO = patient-reported outcome; RCR = Roche Clinical Repository; SAE = serious adverse event.

Notes: All assessments *during treatment period and at the follow-up Day 28 visit* should be performed within 7 days of the scheduled visit, unless otherwise specified. On treatment days, all assessments should be performed prior to dosing, unless otherwise specified. *Following the end of treatment assessment (3 months after treatment completion/early termination) all other follow-up assessments, whether tumor assessments or other study assessments, will be done within ±14 days for 3-monthly and within a month for 6-monthly assessments of the scheduled visits.*

[a] Visit should be performed no earlier than 2 months and no later than 3 months after end of treatment. If the patient is in CR or PR following the CT scan a bone marrow examination should be performed a minimum of 3 months after the end of the treatment. This visit also correlates to 2 months after the Day 28 after Treatment Completion/Early Termination visit.

[b] Includes pulse rate, respiratory rate and systolic and diastolic blood pressure while the patient is in a seated position, and temperature.

[c] Physical examination includes evaluation of the head, eyes, ears, nose, and throat, and the cardiovascular, dermatological, musculoskeletal, respiratory, gastrointestinal, genitourinary, and neurological systems. Record abnormalities observed at baseline on the General Medical History and Baseline Conditions eCRF. At subsequent visits, record new or worsened clinically significant abnormalities on the Adverse Event eCRF

[d] *PRO questionnaires scheduled for administration during a clinic visit should be completed prior to the performance of non-PRO assessments and the administration of study treatment.*

[e] Includes: WBC count, RBC count, hemoglobin, hematocrit, platelet count, differential count (neutrophils, eosinophils, basophils, monocytes, lymphocytes, other cells.

[f] Includes: sodium, potassium, chloride, bicarbonate, glucose, BUN or urea, creatinine, total protein, albumin, phosphorus, calcium, total and direct bilirubin, alkaline phosphatase, ALT, AST, uric acid, LDH.

[g] Includes: dipstick pH, specific gravity, glucose, protein, ketones, blood) and microscopic examination (sediment, RBCs, WBCs, casts, crystals, epithelial cells, bacteria).

[h] For patients who have signed the additional consent for GCLLSG biobanking, residual sample will be sent to the GCLLSG for analysis.

[i] Blood for MRD assessment will include 10 mL of blood at all timepoints for ASO-PCR; 5 or 6 mL at baseline and 10 mL at other timepoints for analysis by NGS and flow cytometry

[j] *Blood for MRD assessment will include 10 mL of blood for ASO-PCR and 10 mL for analysis by NGS at 24 months after treatment completion/early termination and then every 6 months thereafter (until 5 years from last patient enrolled).*

[k] A bone marrow aspirate must be taken within 8 weeks from patients who achieve a CR/PR for central assessment of MRD. Bone marrow aspirate (separate sample, assessed at a local laboratory) and biopsy to confirm CR is required.

[l] For samples drawn on days of study treatment, predose laboratory samples should be drawn within 0–4 hours before the dose. Other laboratory samples occurring on the same day should be obtained within a ± 15-minute window of any scheduled time. Any laboratory tests occurring at time intervals ≥ 24 hours after dose should be obtained within a ± 2-hour window of the scheduled time.

Appendix 4
Schedule of Pharmacokinetic Assessments

Pharmacokinetics is defined in this protocol as a dual timepoint drug level testing (18 mL blood at Cycle 4, Day 1 in all venetoclax [GDC-0199] patients [Arm A]).

Blood samples to assess venetoclax and obinutuzumab concentrations in plasma and serum, respectively, will be collected at the following timepoints in patients who are randomized to Treatment A:

Visit	Timepoint	Sample type and analyte
Cycle 4, Day 1	Pre venetoclax dose	Plasma for venetoclax
	4 hours (± 1 hour) post- venetoclax dose	Plasma for venetoclax
	Pre-obinutuzumab infusion	Serum for obinutuzumab
	End of obinutuzumab infusion	Serum for obinutuzumab

Appendix 5
Sample List of Excluded and Cautionary Medications for Patients Randomized to Arm A (Venetoclax + Obinutuzumab)

Type	Medication
Excluded during the venetoclax ramp-up period and cautionary after patients are on 400 mg/day of venetoclax	
Strong CYP3A inducers	Avasimibe, carbamazepine (Tegretol®), phenobarbital, phenytoin (Dilantin®), rifampin (Rifadin®), and St. John's wort
Moderate CYP3A inducers	Bosentan, efavirenz, etravirine, modafinil, and nafcillin
Strong CYP3A inhibitors [a]	Boceprevir, clarithromycin, conivaptan, indinavir, itraconazole, ketoconazole, mibefradil, lopinavir/ritonavir, nefazodone, nelfinavir, ritonavir, posaconazole, saquinavir, telaprevir, telithromycin, and voriconazole
Moderate CYP3A inhibitors [a]	Amprenavir, aprepitant, atazanavir, ciprofloxacin, crizotinib[b], darunavir/ritonavir, diltiazem, erythromycin, fluconazole, fosamprenavir, imatinib [b], and verapamil
Cautionary throughout the study	
Warfarin	—
Weak CYP3A inducers	Amprenavir, aprepitant, armodafinil, clobazamechinacea, pioglitazone, prednisone, rufinamide, and vemurafenib [b]
Weak CYP3A inhibitors	Alprazolam, amiodarone, amlodipine, atorvastatin, bicalutamide[b], cilostazol, cimetidine, cyclosporine [b], fluoxetine, fluvoxamine, ginkgo, goldenseal, isoniazid, nilotinib[b], oral contraceptives, pazopanib[b], ranitidine, ranolazine, tipranavir/ritonavir, ticagrelor, and zileuton
P-gp substrates	Aliskiren, ambrisentan, colchicine, dabigatran etexilate, digoxin, everolimus[b], fexofenadine, lapatinib[b], loperamide, maraviroc, nilotinib[b], ranolazine, saxagliptin, sirolimus[b], sitagliptin, talinolol, tolvaptan, and topotecan [b]
BCRP substrates	Methotrexate[b], mitoxantrone[b], irrinotecan[b], lapatinib[b], rosuvastatin, sulfasalazine, and topotecan[b]
OATP1B1/1B3 substrates	Atrasentan, atorvastatin, ezetimibe, fluvastatin, glyburide, rosuvastatin, simvastatin acid, pitavastatin, pravastatin, repaglinide, telmisartan, valsartan, and olmesartan
P-gp inhibitors	Amiodarone, azithromycin, captopril, carvedilol, dronedarone, felodipine, quercetin, ronalzine, and ticagrelor

Appendix 5
Sample List of Excluded and Cautionary Medications for Patients Randomized to Arm A (Venetoclax + Obintuzumab) (cont.)

Type	Medication
BCRP inhibitors	Gefitinib[b], cyclosporine[b]
OATP1B1/B3 inhibitors	Gemfibrozil, eltrombopag, cyclosporine[b], tipranavir

Note: This is not an exhaustive list. For an updated list, see the following link: http://www.fda.gov/Drugs/DevelopmentApprovalProcess/DevelopmentResources/DrugInteractionsLabeling/ucm080499.htm In addition to the medications listed in this table, subjects receiving venetoclax should not consume grapefruit, grapefruit products, Seville oranges (including marmalade containing Seville oranges), or star fruits.

[a] After discontinuation of a strong or moderate CYP3A inhibitor, wait for 3 days before venetoclax dose is increased back to the initial maintenance/target dose.

[b] These are anticancer agents; contact Medical Monitor before use.

Appendix 6
The Modified Cumulative Illness Rating Scale (CIRS)

Body system	Score				
1. Cardiac (heart only)	0	1	2	3	4
2. Hypertension (rating is based on severity; organ damage is rated separately)	0	1	2	3	4
3. Vascular (blood, blood vessels and cells, bone marrow, spleen, lymphatics)	0	1	2	3	4
4. Respiratory (lungs, bronchi, trachea below the larynx)	0	1	2	3	4
5. EENT (eye, ear, nose, throat, larynx)	0	1	2	3	4
6. Upper GI (esophagus, stomach, and duodenum; pancreas; do not include diabetes)	0	1	2	3	4
7. Lower GI (intestines, hernias)	0	1	2	3	4
8. Hepatic (liver and biliary tree)	0	1	2	3	4
9. Renal (kidneys only)	0	1	2	3	4
10. Other GU (ureters, bladder, urethra, prostate, genitals)	0	1	2	3	4
11. Muscolo-skeletal-integumentary (muscle, bone, skin)	0	1	2	3	4
12. Neurological (brain, spinal cord, nerves, do not include dementia)	0	1	2	3	4
13. Endocrine-Metabolic (includes diabetes, thyroid; breast; systemic infections; toxicity)	0	1	2	3	4
14. Psychiatric/Behavioral (includes dementia, depression, anxiety, agitation/delirium, psychosis)	0	1	2	3	4

PHILOSOPHY AND DEVELOPMENT OF THE SCALE

Compiling and quantifying medical problems in the elderly population would allow meaningful comparison of medical burden and treatment outcomes in elderly patients with variable and complex medical problems.

The Cumulative Illness Rating Scale (CIRS) was initially developed by Linn et al. and published in JAGS 1968; it appeared immediately a user friendly but comprehensive review of medical problems by organ systems, based on a 0–4 rating, yielding a cumulative score.

This scale was successively revised by Miller et al. to reflect common problems of the elderly with an emphasis on morbidity using specific examples and was renamed CIRS for Geriatrics (CIRS-G) (Miller et al. 1992); moreover, Miller and Towers provided also a manual of guidelines for scoring their version (Miller et al. 1991).

Then, in 1995, Parmelee et al. validated a Modified CIRS version, based on a 1–5 rating and with some differences in categories, in a geriatric residential population (Parmelee et al. 1995).

Finally, Mistry et al. used this latter Modified CIRS version with a 0 thru 4 rating to measure medical burden in psychogeriatric participants of the UPBEAT program, showing that inclusion of acute medical conditions did not undermine the usefulness of the CIRS (Mistry et al. 2004).

Based on the version of Miller and Towers, current guidelines were adapted to the Modified CIRS version and updated.

EDUCATION OF RATER

Nurses, physician assistants, nurse practitioners, or physicians are required to have a necessary background for completing this scale. Due to the judgment required, some physician consultation may be necessary to clarify complex medical problems or their severity.

THE MINIMUM DATABASE REQUIRED

It is expected that every patient should have a complete history and physical examination with a detailed problem list, height and weight (to calculate body mass index, BMI), and baseline labs including a complete blood count and differential, chemistry profile to include electrolytes, liver and kidney function, serum B12, thyroid function, cholesterol level, hemoglobin A1c (for diabetic patients), and an EKG. For rating psychiatric conditions, the rater is expected to be familiar with the Mini Mental Status Examination (Folstein et al. 1975), and the Diagnostic and Statistical Manual IV (DSM IV) APA 1994). A checklist of needed information is available in the Appendix.

RATING SUGGESTIONS (GENERAL PRINCIPLES)

Every single disease must be classified in the appropriate system. If there are several problems in the same system, only the most severe is rated. Example: for a patient suffering from a well-controlled angina (Rated 2) and terminal heart failure (Rated 4), only the higher rated condition would be scored in the Cardiac system (e.g., rating is 4).

The spread of a cancer may lead to rate the condition in more than one category. For example, a lung cancer with bone metastases treated with nonsteroidal anti-inflammatory drugs (NSAIDs) is Rated 4 in Respiratory and 2 in Musculoskeletal.

Appendix 6
The Modified Cumulative Illness Rating Scale (CIRS) (cont.)

General rules for severity rating:

1. No problem affecting that system or past problem without clinical relevance.
2. Current mild problem or past significant problem.
3. Moderate disability or morbidity and/or requires first line therapy.
4. Severe problem and/or constant and significant disability and/or hard to control chronic problems (complex therapeutic regimen).
5. Extremely severe problem and/or immediate treatment required and/or organ failure and/or severe functional impairment.

LEVEL 0

No problem or healed minor injuries; past childhood illnesses (chickenpox); minor surgery (carpal tunnel completely healed, caesarean); uncomplicated healed fractures; other past problems healed without sequel, residual or complication (pneumonia).

LEVEL 1

Any current medical problem that causes mild discomfort or disability, or has occasional exacerbations, having only minor impact on morbidity (asthma controlled with PRN bronchodilators, occasional heartburn relieved with PRN antacids). Medical problems that are not currently active but were significant problems in the past (passage of a kidney stone) or required major surgery (hysterectomy, cholecystectomy, appendectomy).

LEVEL 2

Medical conditions that require daily treatment or first line therapy (asthma controlled with inhaled steroids, gastro-esophageal reflux treated with daily medication, osteoarthritis requiring daily NSAID, etc.) and/or have moderate disability or morbidity.

LEVEL 3

Chronic conditions that are not controlled with first line therapy (asthma needing continuous corticosteroid therapy, symptomatic angina despite medical regimes, heart failure with symptoms or uncontrolled hypertension despite complex therapeutic regimen) and/or constant significant disability, but not severe disability.

LEVEL 4

Any acute condition that requires immediate treatment or hospitalization (unstable angina, acute myocardial infarction, stroke, but also bladder outlet obstruction)

and/or extremely severe problems; organ failure (end-stage renal disease needing dialysis, oxygen-dependent chronic obstructive pulmonary disease, terminal heart failure); severe sensory impairment (almost complete blindness or deafness, being wheelchair bound) and/or severely affected quality of life, severe impairment in function; delirium by medical (organic) conditions.

RATING MALIGNANCIES

Consistent scoring of severity ratings for various malignancies is a difficult problem. Each malignancy has its own rating system and prognostic indicators, the complexity of which would quickly exceed the aim of the intended simplicity and ease of use of CIRS.

The following general guidelines are intended to provide a reasonably accurate delineation of medical burden for cancer without excessive complexity.

- Level 1: Cancer diagnosed in the remote past without evidence of recurrence or sequel in the past 10 years or skin cancer excised in the past without major sequel (other than melanoma).
- Level 2: No evidence of recurrence or sequel in the past 5 years.
- Level 3: Required chemotherapy, radiation, hormonal therapy or surgical procedure for cancer in the past 5 years.
- Level 4: Recurrent malignancy or metastasis (other than to lymph glands) or palliative treatment stage.

These ratings are to be made in the appropriate organ category for a given malignancy.

ORGAN-SPECIFIC CATEGORIES

The following organ-specific categories will attempt to provide guidelines for consistent rating of comparable severity. Common conditions will be stressed with the focus on the “judgment strategy” that can be applied to other problems not listed.

If there are several problems in the same system, only the most severe is rated.

HEART

In this category, only heart and coronary disease have to be considered (not vascular): coronary arteries disease, heart failure, valvular heart diseases, heart disease secondary to hypertension, endocardities, miocardities, pericardities, arrhythmias (extrasystoles, bundle-branch blocks, atrial fibrillation, PMK placement), heart malignancies. Functional impact must be considered too (e.g., NYHA II heart failure has different value between dependent and independent persons).

0. No problems

1. Remote MI (>5 years ago); occasional [exertion] angina; asymptomatic valvular disease
2. CHF compensated with meds (NYHA I-II); daily anti-angina meds; left ventricular hypertrophy; atrial fibrillation, bundle branch block, daily anti-arrhythmic drugs (even for prophylaxis); PMK placement for asymptomatic bradycardia (relieved by Holter EKG monitoring); valvular disease requiring medical treatment
3. Previous MI (<5 years ago); abnormal stress test; status post (previous) percutaneous coronary angioplasty, coronary artery bypass graft surgery or other cardiac surgery (valve replacement); moderate CHF (NYHA II–III) or complex medical treatment; bifascicular block; PMK placement for cardiogenic syncope; pericardial effusion or pericarditis
4. Acute coronary syndrome, unstable angina or acute MI; intractable CHF (NYHA III–IV acute or chronic); marked restriction to the normal activity of daily living secondary to cardiac status

HYPERTENSION

Consider only hypertension severity; organ damage (complications) should be considered into the respective categories.

0. Normotension
1. Borderline hypertension; hypertension compensated with salt restriction and weight loss, drug free (when drug therapy is indicated, but the patient does not take meds, the score is at least 2)
2. Daily antihypertensive meds: hypertension controlled by 1 pill therapy (even fixed doses combinations)
3. Hypertension requiring two or more pills for control
4. Malignant hypertension, or hypertension non-controlled by complex therapeutic regimen

VASCULAR-HEMATOPOIETIC

Artery disease: carotid atherosclerosis, peripheral arteries disease (PAD), aneurysms (every site);

Venous disease: venous insufficiency, varices, deep venous thrombosis (DVT), pulmonary embolism, primary pulmonary hypertension;

Hematopoietic disease: anemia, leucopenia, thrombocytopenia, hematological malignancy;

Lymphopoietic disease: chronic lymphatic edema, lymphoma, spleen and thymus disease;

Immunologic disease: systemic lupus erythematosus, systemic sclerosis (scleroderma), sarcoidosis, hypersensitivity

5. No problem
6. Venous insufficiency, varices, lymphedema; carotid stenosis <70%; hemoglobin 10–12 g/dL (in females), 12–14 g/dL (in males); anemia of chronic "inflammatory" disease
7. Previous DVT; one symptom of atherosclerosis disease (claudication, bruit, amaurosis fugax, absent pedal pulses) or daily meds (e.g., anti-platelets drugs); PAD IIa–IIb by Fontaine; carotid stenosis >70%; aortic aneurysm <4 cm; hemoglobin 8–10 g/dL (in females), 10–12 g/dL (in males); anemia secondary to iron, B12 vitamin or folate deficiency, or to chronic renal failure; total white blood cell (WBC) 2000–4000/mmc; mild thrombocytopenia (50000–150000/mmc)
8. DVT or recent DVT (<6 months ago); two or more symptoms of atherosclerosis (see above); PAD Fontaine III or recent/previous angioplasty (with or without stenting); hemoglobin <8g/dL (in females), <10 g/dL (in males); dyserythropoietic anemia; WBC <2000/mmc; severe thrombocytopenia (<50000/mmc)
9. Pulmonary embolism (acute or recent/previous); atherosclerosis requiring surgical intervention (e.g., aortic aneurysm >4 cm, symptomatic carotid stenosis >70%, PAD Fontaine IV or amputation for vascular causes, etc.); recent/previous vascular surgery; any hematological or vascular malignancy (including multiple myeloma)

In case of immunological disease, score should be assigned by considering blood abnormalities, stadium of organ damage and/or functional disability (2: symptoms controlled by daily meds; 3: symptoms not well controlled; 4: symptoms impossible to be controlled or short time poor prognosis).

RESPIRATORY

In this category COPD, asthma, emphysema, restrictive pulmonary interstitial lung diseases, malignancies of lung and pleura, pneumonia, and smoking status are considered.

10. No problem
11. Recurrent episodes of acute bronchitis; currently treated asthma with prn inhalers when required; cigarette smoker >10 but <20 pack years

Appendix 6
The Modified Cumulative Illness Rating Scale (CIRS) (cont.)

12. Instrumental diagnosis of COPD or pulmonary interstitial disease (X-ray, TC, spirometry); daily prn inhalers (≤ 2 pharmacological classes); two or more episodes of pneumonia in the last 5 years; cigarette smoker < 20 but < 40 pack-years
13. Exertion dyspnea secondary to limited respiratory capacity, not well controlled by daily meds; required oral steroids for lung disease; daily prn inhalers (3 pharmacological classes); acute pneumonia treated as an outpatient
14. Chronic supplementation of oxygen; respiratory failure requiring assisted ventilation, or previous (at least one episode); any lung or pleural neoplasm; acute pneumonia requiring hospitalization

Smoking is an important respiratory and cardiovascular risk, so it is considered as a disease, and it is rated according to *lifetime pack-years*:

Number of cigarette packs smoked per day $\times$ Number of years smoked in their lifetime

e.g., 1 pack-year $=$ 20 cigarettes/die (1 pack) $\times$ 1 year

Ex-smokers should be rated too, but those who have been smoke-free for the most recent 20 years would merit a lower rating than those who are currently smoking.

Examples:

a) Patient smoking 20 cig/die (1 pack) for 25 years $=$ 25 pack-years $-$ CIRS score: 2
b) Patient smoking 40 cig/die (2 packs) for 25 years $=$ 50 pack-years $-$ CIRS score: 3
c) Ex-smoker of 20 cig/die (1 pack) for 25 years, he stopped 5 years ago $-$ CIRS score: 2
d) Ex-smoker of 20 cig/die (1 pack) for 25 years, he stopped 20 years ago $-$ CIRS score: 1

Classification of COPD could be more specific when instrumental data (objective evidence) are available: blood gases, forced expiratory volume in 1 second (FEV1), etc.

EYES, EARS, NOSE & THROAT, AND LARYNX

To simplify the potential complexity of this category it was decided to score according to the severity of the disability created by sensory diseases (degree of limited autonomy and communication), and avoid rating each type of pathology. Sensory impairments should be rated after instrumental correction (corrective lenses, hearing aid, etc.).

Eyes: glaucoma, cataracts, macular degeneration (diabetic/hypertensive retinopathy), any other pathology

Ears: otitis, dizziness, any cause of hearing impairment

Nose & Throat: rhinitis, pharyngitis, nasal polyps, sinusitis, malignancies

Larynx: dysphonia, acute and chronic laryngitis, malignancies

15. No problems
16. Corrected vision with glasses; mild hearing loss; chronic sinusitis
17. Difficulty in reading newspaper or drive although glasses; required hearing aid; chronic sinonasal complaints requiring medication; vertigo/dizziness requiring daily meds
18. Severe low vision, partially blind (required an escort to venture out, unable to read newspaper); severe ear impairment (conversational heading still impaired with hearing aid); laryngeal dysphonia (not neurological dysarthria)
19. Functional blindness/deafness: unable to read, recognize a familiar face, unable to conversational heading, even if "organically" he is not completely blind or deaf; laryngectomy (every cause, especially malignancies); required surgical intervention for vertigo; aphonia secondary to laryngeal impairment.

UPPER GASTROINTESTINAL SYSTEM

This category is comprehensive of the intestinal tract from esophagus to duodenum, and pancreatic trees: dysphagia, GERD, hiatal hernia, esophageal diverticula, any type of gastritis (consider also *H. Pylori* eradication or not), gastric/duodenal ulcer, acute or chronic pancreatitis, malignancies (comprehensive of gastric lymphoma).

Ensure that type 1 diabetes is rated under "metabolic."

20. No problem
21. Hiatal hernia, GERD or gastritis requiring prn meds; previous ulcer (>5 years ago); previous *H. Pylori* eradication therapy (>5 years ago)
22. Daily proton pump inhibitor/anti-acid meds; documented gastric or duodenal ulcer or *H. Pylori* eradication therapy within 5 years
23. Active gastric or duodenal ulcer; positive fecal occult blood test; any swallowing disorder or dysphagia; chronic pancreatitis requiring supplemental pancreatic enzymes for digestion; previous episode of acute pancreatitis
24. Any type of malignancies (see "Rating Malignancies"); previous gastric surgery because of cancer; history of perforated ulcer (gastric surgery not because of cancer, ulcorrhaphy); melena/heavy bleeding from upper GI source; acute pancreatitis

Appendix 6
The Modified Cumulative Illness Rating Scale (CIRS) (cont.)

LOWER GASTROINTESTINAL SYSTEM

Comprehensive of the rest of the GI system, from small bowel to anus:
Whipple's disease, diverticulosis, irritable bowel, malignancies. Constipation is rated, too, by type and frequency of laxatives required, or by history of impaction.

25. No problems, previous appendectomy, previous hernia repair (without complications)
26. Constipation managed with prn meds; active hemorrhoids; intestinal hernia requiring surgery; previous hernia repair with complications (intestinal adherences, laparocele, etc.); irritable bowel syndrome (few symptoms)
27. Constipation requiring daily bulk laxatives (psyllium, policarbophil, sterculia, guar gum, etc.), or stool softeners; diverticulosis (previous diverticulitis); inflammatory bowel disease in remission with meds (>5 years ago)
28. Bowel impaction/diverticulitis within the last year; daily use of stimulant (irritant) or osmotic laxatives (bisacodyl, senna, glycerol, sodium docusate; lactulose, polyethylene glycol) or enemas; chronic bowel inflammation in remission with meds (<5 years ago)
29. Diverticulitis flare up; active inflammatory disease; current impaction; hematochezia/active bleeding from lower GI source; bowel carcinoma

LIVER AND BILIARY TREES

Comprehensive of liver, gallbladder, biliary trees, portal system: acute and chronic hepatitis (viral, alcoholic, toxic, autoimmune, idiopathic), cirrhosis, portal hypertension, hemochromatosis, primary biliary cirrhosis, cholelithiasis, cholangitis, primary malignancies. As the hepato-biliary system is difficult to assess through the physical examination, therefore, laboratory results must be used.

30. No problem
31. History of hepatitis (actually normal values of transaminases); cholecystectomy
32. Cholelithiasis; chronic hepatitis or previous hepatitis (<5 years ago) or any other liver disease (hemochromatosis, primary biliary cirrhosis) with mildly elevated transaminases (within 3-times normal values); heavy alcohol use within 5 years (to rate in "psychiatric", too)
33. Chronic hepatitis or any other liver disease with marked elevation of transaminases (>3-times normal values); elevated bilirubin
34. Acute cholecystitis; any biliary obstruction; active hepatitis/liver cirrhosis; any liver or biliary tree carcinoma

RENAL

This category is exclusive of kidney: kidney stones, acute/chronic renal failure, glomerulonephritis; nephrosic/nephritic syndrome; active/chronic pyelonephritis, diabetic or hypertensive nephropathy (albuminuria/proteinuria), renal carcinoma. Bence-Jones proteinuria in multiple myeloma should not be considered.

35. No problem
36. Asymptomatic kidney stone; kidney stone passage within the last ten years; pyelonephritis within 5 years; kidney cysts without hematuria
37. Serum creatinine $>$1.5 but $<$3 mg/dL without diuretic or antihypertensive medication (particularly ACE-inhibitors or SRAA blockers); kidney calculi requiring daily meds
38. Serum creatinine $>$3 mg/dL or $<$1.5 mg/dL in conjunction with diuretics, antihypertensive, or bicarbonate therapy; active pyelonephritis; nephrosic syndrome; colic symptoms treated as an outpatient
39. Required dialysis; renal carcinoma; colic symptoms requiring hospitalization

GENITOURINARY

Ureters, bladder, urethra. Genitals, prostate, testicles, penis, seminal vesicles.

Uterus, ovaries. *Mammary gland is rated under "metabolic".*

This category is comprehensive of all GU tract impairments: ureteral or bladder stones, benign prostate hypertrophy (BPH), urinary tract infections (UTI's), prolapses, etc. Urinary incontinence and indwelling catheter should also be considered.

40. No problem
41. Stress incontinence; BPH without urinary symptoms; hysterectomy or ovariectomy (uterine fibroma, benign neoplasm)
42. Pathological pap smear (or 2 consecutives abnormal); frequent UTIs (3 or more in the past year) in female or current UTIs; urinary incontinence (not stress) in females; BPH with urinary symptoms (frequency, urgency, hesitancy); status post TURP; any urinary diversion procedure; indwelling catheter; bladder calculi
43. Prostatic cancer in situ (e.g., incidentally found during TURP); vaginal bleeding; cervical carcinoma in situ; hematuria (any cause); urinary incontinence (not stress) in males; bladder polyps
44. Acute urinary retention; current urosepsis; any GU malignancies except as above

MUSCULOSKELETAL/INTEGUMENT

This is a very broad category, including: osteoarthritis, osteoporosis, any bone fracture; primary neoplasm (bone, muscle, connective tissue, skin), distinguishing melanoma from other localized skin cancers; rheumatoid arthritis and polymyalgia rheumatica; muscular

injuries (rotator cuff, long head of the biceps); pressure sores; any dermatological disease.

The scores of this category are strictly correlated to the disability they cause; for the evaluation of the level of disability, refer to BADL and IADL.

NOTICE: Score the severity of each illness according to the level of disability caused by the same illness in this category, without considering the disability caused by other diseases. For example: a patient affected both by osteoarthritis and hemiplegia from a previous stroke has a high level of disability, but you have to score 2 for disability by osteoarthritis (in this category) and 4 for disability by stroke (in the neurological category); for a patient with both a deforming rheumatoid arthritis and a previous stroke without remaining outcomes you have to score 4 for disability from arthritis (in this category) and 2 for disability from stroke (in the neurological category).

45. No problem
46. Requires PRN meds for osteoarthritis (NSAID) or has mildly limited IADL from joint pathology; excised skin cancers (except melanoma); skin infections requiring antibiotics within a year
47. Daily anti-osteoarthritis meds (NSAID) or use of assistive devices or little limitation in ADL (previous arthroprosthesis or treated fracture with a low level of remaining disability); osteoporosis without vertebral fractures; daily meds for chronic skin diseases (even local, as psoriasis or pressure sores); non-metastatic melanoma; daily meds for rheumatoid arthritis (except steroids) with a low level of disability
48. Osteoarthritis with a moderate level of disability in ADL; requires chronic treatment with steroids for arthritic conditions or joints' deformities or severely impaired; osteoporosis with vertebral compression fractures
49. Wheelchair bound for osteomuscular disease; severe joint deformities or severely impaired usage; osteomyelitis; any bone or muscle or connective tissue neoplasm (see "Rating Malignancies"); metastatic melanoma.

Fractures and/or arthroprosthesis (both recent and old) have to be scored according to the level of disability they cause (considering outcomes too), in order to avoid confusion about possible classifications of different fractures or joints. The same is true for muscular diseases.

CENTRAL AND PERIPHERAL NERVOUS SYSTEM

This category includes the "somatic" pathologies of the central and peripheral nervous system: any kind of stroke, neurodegenerative diseases (Parkinson's disease and parkinsonism, multiple sclerosis, amyotrophic lateral sclerosis, etc.), myelopathies, traumas with neurological outcomes, primary or secondary epilepsy, neuropathies (diabetic, alcoholic, any other etiology), primary tumors, chronic headaches (migraine), insomnia, etc. It must carefully estimate the severity and prognosis of the illness but also the functional impairment that the illness causes.

50. No problem (or fewer convulsions in childhood)
51. Frequent headaches requiring PRN meds without impairment in Advanced ADL; previous TIA (one event); previous epilepsy, actually not treated, without crisis since more than 10 years ago.
52. Chronic headache requiring daily meds (even for prophylaxis) or with regularly functional impairment in Advanced ADL (bed rest, job withdrawal, etc.); actual TIA or more than one previous TIA; previous stroke without significant residual; mild severity neurodegenerative diseases (see above), treated and well controlled; epilepsy controlled with drugs.
53. Previous stroke with mild residual dysfunction (hemiparesis, dysarthria); any neurosurgical procedure; moderate severity neurodegenerative diseases (see above), not well controlled by meds; epilepsy in treatment but with periodic crisis.
54. Acute stroke or previous stroke with severe residual dysfunction (hemiplegia, aphasia, severe vascular dementia) or more than one previous stroke (multi-infarct encephalopathy); severe neurodegenerative diseases (see above) causing disability in ADL; neurological coma.

Alzheimer's disease and dementia should not be rated into this category (Psychiatric and behavioral diseases): Alzheimer's disease should be listed only under psychiatric disorders; if dementia stems from vascular and/or mixed dementia and/or other neurological condition (e.g., Parkinson's Disease), both "neurologic" and "psychiatric" categories should be endorsed at the appropriate level for severity, considering in this category the stroke and the multi-infarct encephalopathy responsible for the cognitive impairment (score 3 for stroke with remaining outcomes, score 4 for multi-infarct encephalopathy).

ENDOCRINE-METABOLIC SYSTEM AND BREAST (SYSTEMIC INFECTIONS AND POISONINGS)

Type 1 and Type 2 diabetes (organ damage should be considered into the respective categories, like for hypertension), obesity and dyslipidemia (hypercholesterolemia)

represent the core of this category; it includes also hypo- and hyper-thyroidism, hypo- and hyper-parathyroidism, adrenal pathologies (Cushing' or Addison' disease), hypogonadism, hypopituitarism, etc. Malignancies of these glands, both benignant (like thyroid nodules) and malignant (like thyroid or adrenal cancer, vipoma, etc.) are included too.

Even if it is an exocrine gland, breast was included in this category because the authors did not find a more appropriate one; so it also includes breast cancer.

Moreover, it includes: electrolyte disorders, sepsis, systemic infections (like tuberculosis, syphilis, AIDS) scored according to their severity and the functional impairment they cause (see general indications) and poisonings (chronic by metals or acute by pesticides or carbon monoxide).

55. No problem
56. Diabetes and/or dyslipidemia compensated with diet; mild obesity (BMI 30-35 kg/m2); hypothyroidism in replacement therapy (L-thyroxin); hyperthyroidism caused by Plummer' adenoma surgically treated.
57. Diabetes compensated with oral hypoglycemic drugs or insulin (hemoglobin $A_{1c} < 7\%$); dyslipidemia well controlled by daily meds (c-LDL lower than the recommended target according to the individual global cardiovascular risk); moderate obesity (BMI 35–45 kg/m^2); hyperthyroidism in pharmacologic treatment; asymptomatic or surgically treated hyperparathyroidism; fibrocystic breast disease.
58. Diabetes not well compensated by therapy (hemoglobin A_{1c} 7–8.5%, presence of complications); dyslipidemia not well controlled (c-LDL higher than the recommended target according to the individual global cardiovascular risk; for instance, c-LDL $>$100 mg/dL in patients with previous myocardial infarction or stroke); severe obesity (BMI $>$45 kg/m^2); symptomatic hyperparathyroidism (e.g., hypercalcaemia); replacement therapy for adrenal failure; any electrolytes disorder requiring hospitalization.

59. Uncontrolled diabetes (hemoglobin $A_{1c} > 8.5\%$) or one diabetic ketoacidosis or nonketotic hyperosmolar coma during the past year; genetic uncontrolled dyslipidemia; acute adrenal failure during hormonal replacement therapy; any neoplasm of thyroid, breast, adrenal gland (see “Rating Malignancies”).

NOTICE: when the patient is not treated with drug therapy for diabetes or dyslipidemia but he should be for the optimal control of the pathology (for instance, hemoglobin $A_{1c} > 7\%$, total cholesterol > 250 mg/dL), score the pathology according to the laboratory values, which really define its severity.

PSYCHIATRIC AND BEHAVIORAL DISEASES

This category includes both dementia and related behavioral disorders (psychosis, anxiety, depression, agitation) and all the pre-existing and/or not related to dementia psychiatric disorders. Since this is the only item analyzing patient’s mental status (all the others refer to physical status), it is very important to evaluate it considering carefully further information derived from the Comprehensive Geriatric Assessment (MMSE; Geriatric Depression Scale, Neuro-Psychiatric Inventory if available).

60. No psychiatric problem or history thereof
61. Minor psychiatric condition or history thereof: previous (occasional) psychiatric treatment without hospitalization; major depressive event and/or use of antidepressants more than 10 years ago without hospitalization; occasional use of minor tranquilizers (e.g., BDZ; even if as hypnotherapy for insomnia); mild cognitive impairment (MMSE 25-28).
62. A history of major depression (according to DSM-IV criteria) within the last 10 years (treated or untreated); mild dementia (MMSE 20-25); previous admission to Psychiatric Department for any reason; history of substance abuse (more than ten years ago, including alcoholism).
63. Current major depression (according to DSM-IV criteria) or more than two previous major depression episodes in the past 10 years; moderate dementia (MMSE 15–20); current and usual usage of daily anti-anxiety meds (even as hypnotherapy for insomnia); current or within the past ten years substance abuse or dependence (according to DSM-IV criteria); requires daily antipsychotic medication; previous attempt at suicide.
64. Current mental illness requiring psychiatric hospitalization, institutionalization, or intensive outpatient management (psychiatric emergency, as attempt at suicide or severe depression with suicide purpose, acute psychosis or acute decompensation of chronic psychosis, severe substance abuse; severe agitation from dementia); severe dementia (MMSE < 15); delirium (acute confusion or altered

mental status for medical (organic) reasons: in this case you have to codify also the medical cause in its own category with the appropriate level of severity).

It could be requested psychiatric consult for this category; dementia and depression, the most frequent diseases in the elderly, can be scored in details using the MMSE and GDS. The severity of any mental disorder (dementia, depression, anxiety, psychosis, substance abuse and all the others) has to be scored according to the level of functional impairment or disability they cause.

REFERENCES

Linn BS, Linn MW, Gurel L. Cumulative Illness Rating Scale. J Am Geriatr Soc 1968; 16:622-6.

Miller MD, Paradis CF, Houck PR, Mazumdar S, Stack JA, Rifai H, Mulsant B, Reynolds CF III. Rating chronic medical illness burden in geropsychiatric practice and research: application of the Cumulative Illness Rating Scale. Psychiatry Res 1992; 41:237-48.

Miller MD, Towers A. A manual guidelines for scoring the Cumulative Illness Rating Scale for Geriatrics (CIRS-G). Pittsburg, PA: University of Pittsburgh; 1991.

Parmelee PA, Thuras PD, Katz IR, Lawton MP. Validation of the Cumulative Illness Rating Scale in a geriatric residential population. J Am Geriatr Soc 1995; 43:130-7.

Mistry R, Gokhman I, Bastani R, Gould R, Jimenez E, Maxwell A, McDermott C, Rosansky J, Van Stone W, Jarvik L; UPBEAT Collaborative Group. Measuring medical burden using CIRS in older veterans enrolled in UPBEAT, a psychogeriatric treatment program: a pilot study. J Gerontol A Biol Sci Med Sci 2004; 59:1068-75.

Folstein MF, Folstein SE, McHugh PR. “Mini Mental State”: a pratical method for grading the cognitive state of patients for the clinician. J Psichiatr Res 1975; 12:189-98.

American Psychiatric Association. Diagnostic and Statistical Manual of Mental Disorders: DSM-IV. 4th ed. Washington: Amer. Psychiatric Assn., 1994.

Yesavage JA, Brink TL, Rose TL, et al. Development and validation of a geriatric depression screening scale: a preliminary report. J Psichiatr Res 1983; 17:37-49.

Cummings JL, Mega M, Gray K, Rosenberg-Thompson S, Carusi DA, Gornbein J. The Neuropsychiatric Inventory: comprehensive assessment of psychopathology in dementia. Neurology 1994; 44:2308-14.

CHECKLIST

Medical history

1. Timing of events and/or interventions (how long ago underwent surgery for…; how long ago had myocardial infarction or stroke, etc.) and evaluation of functional impairment

2. Drugs list (fundamental), including laxatives and tranquilizers (even hypnoinducent)
3. Symptoms of atherosclerotic disease (TIA, angina, claudication, amaurosis)
4. Etiological diagnosis (reasonably reliable) of anemia
5. Degree of vascular stenosis or aneurism dimension (by Doppler and/or ultrasound and/or TC data, when available)
6. Information about smoking status (how many cigarettes per day for how many years, when stopped)
7. Glasses use? With this aid, the patient is able to read a newspaper? Requires an escort to venture out?
8. Any hearing aid? (you should evaluate possibility to communicate with patient)
9. “Peptic history” of the patient (including previous eradication therapy for H. Pylori)
10. Urinary symptoms, incontinence, presence of bladder catheter (even from BADL)

PHYSICAL EXAMINATION

a) Height (m^2) and weight (kg) (measured, not reported, if possible) to calculate BMI
b) Blood pressure, heart rate, cardiac murmurs, peripheral arterial pulses
c) Joint pain or passive stiffness limitation (non–X-ray-based diagnosis of osteoarthritis)
d) Neurological residual (dysarthria/aphasia, hemiparesis/hemiplegia)

BASELINE LABORATORY SAMPLES

- Blood count: hemoglobin, WBC and platelets
- Creatinine, electrolytes
- Cholesterol levels (total, HDL) and triglycerides
- AST, ALT, fractioned bilirubin
- Thyroid function and serum B12 (when indicated)
- Hemoglobin A_{1c} (for diabetic patients)

Appendix 7
Standard Cockcroft and Gault Formula for Calculated Creatinine Clearance

For serum creatinine concentration in mg/dL:

$$\text{CrCl (mL/min)} = \frac{(140\text{-age}^{+}) \times (\text{wt}) \times 0.85 \text{ (if female), or } \times 1.0 \text{ (if male)}}{72 \times \text{serum creatinine (mg/dL)}}$$

For serum creatinine concentration in μmol/L:

$$\text{CrCl (mL/min)} = \frac{(140\text{-age}^{+}) \times (\text{wt}) \times 0.85 \text{ (if female), or} \times 1.0 \text{ (if male)}}{0.81 \times \text{serum creatinine}}$$

$$= \qquad (\mu\text{mol/L})$$

$^{+}$age in years, weight (wt) in kilograms

Reference: Cockcroft and Gault 1976

Appendix 8
Body Surface Area Calculation (BSA) Calculation Using the Mosteller Formula

The following formula is to be used when calculating BSA:

$BSA(m^2) = ([Height\ (cm) \times Weight\ (kg)]/3600)^{½}$

Appendix 9
National Cancer Institute Common Terminology Criteria

In the present study, toxicities will be graded according to the National Cancer Institute (NCI) Common Terminology Criteria for Adverse Events (CTCAE), Version 4.03.

A pdf of the NCI CTCAE v4.03 can be downloaded from the following Website:

> http://evs.nci.nih.gov/ftp1/CTCAE/CTCAE_4.03_2010-06-14_QuickReference_8.5x11.pdf

Investigators who do not have access to Internet can contact the Data Center to receive a hard copy of this document by mail.

Appendix 10
Recommendations for Initial Management of Electrolyte Imbalances and Prevention of Tumor Lysis Syndrome

PRIOR TO OBINUTUZUMAB INFUSION

Patients with a high tumor burden (absolute lymphocyte count $\geq 25 \times 10^9$/L or bulky lymphadenopathy) must receive prophylaxis for tumor lysis syndrome (TLS) prior to the initiation of treatment. These patients must be well-hydrated. It is desirable to maintain a fluid intake of approximately 3 liters/day, 1–2 days before the first dose of obinutuzumab. All such patients with a high tumor burden must be treated with allopurinol or a suitable alternative treatment starting 12-24 hours prior to the first infusion. Patients should continue to receive repeated prophylaxis with allopurinol and adequate hydration prior to each subsequent infusion, if deemed appropriate by the investigator.

FIRST DOSE OF VENETOCLAX OR DOSE ESCALATION

- Within the first 24 hours after either the first dose or dose escalation, if any laboratory criteria below are met, the patient should be hospitalized for monitoring and the investigator notified. No additional venetoclax doses should be administered until resolution. A rapidly rising serum potassium level is a medical emergency.
- Nephrology (or acute dialysis service) must be consulted/contacted on admission (per institutional standards to ensure emergency dialysis is available).
- Intravenous (IV) fluids (e.g., D5 1/2 normal saline) should be initiated at a rate of at least 1 mL/kg/h rounded to the nearest 10 mL (target 150–200 mL/hr; not $<$50 mL/hr). Modification of fluid rate should also be considered for individuals with specific medical needs.
- Monitor for symptoms or signs of tumor lysis syndrome (TLS) (e.g., fever, chills, tachycardia, nausea, vomiting, diarrhea, diaphoresis, hypotension, muscle aches, weakness, paresthesias, mental status changes, confusion, and seizures). If any clinical features are observed, recheck potassium, phosphorus, uric acid, calcium, and creatinine within 1 hour STAT.
- Vital signs should be taken at time of all blood draws or any intervention.
- The management recommendations below focus on the minimum initial responses required. If a diagnosis of TLS is established, ongoing intensive monitoring and multidisciplinary management will be as per institutional protocols.

In addition to the recommendations for patients with chronic lymphocytic leukemia/ small lymphocytic lymphoma receiving first dose of venetoclax:

- For potassium increase $\geq$0.5 mmol/L from baseline, or any value $>$5.0 mmol/L, recheck potassium, phosphorus, uric acid, calcium, and creatinine within 1 hour STAT and follow first guideline.

- For phosphorus increase of > 0.5 mg/dL AND > 4.5 mg/dL, administer phosphate binder and recheck potassium, phosphorus, uric acid, calcium, and creatinine within 1 hour STAT.

Appendix 11
Adverse Events Commonly Associated with Chronic Lymphocytic Leukemia Study Population and/or Progression of Chronic Lymphocytic Leukemia

DISEASE-RELATED EVENTS

- Lymphadenopathy
- Splenomegaly
- Hepatomegaly
- Leukemia cutis (macules, papules, plaques, nodules, ulcers, or blisters)
- Lymphocytosis
- Cytopenias (neutropenia, anemia and thrombocytopenia)
- Febrile neutropenia
- Autoimmune hemolytic anemia
- Autoimmune thrombocytopenia
- Hypogammaglobulinemia
- Infections (bacterial, viral, and fungal)
- Second cancers (Kaposi's sarcoma, malignant melanoma, squamous cell skin cancer, basal cell carcinoma, cancers of the larynx, colorectal cancer and cancers of the lung)
- Fatigue
- Weight loss
- Pyrexia
- Bruising
- Minor hemorrhages
- Pain (any type)

Appendix 11
Adverse Events Commonly Associated with Chronic Lymphocytic Leukemia (CLL) Study Population and/or Progression of CLL (cont.)

POPULATION-RELATED COEXISTING MEDICAL CONDITIONS

- Hypertension
- Rheumatoid arthritis/osteoarthritis
- Hyperlipidemia
- Peptic ulcer
- Inflammatory bowel disease
- Coronary artery disease
- Peripheral vascular disease
- Cardiomyopathy
- Valvular disease
- Atrial fibrillation
- Diabetes mellitus
- Chronic obstructive pulmonary disease
- Cerebrovascular accident
- Transient ischemia attack

Appendix 12
International Workshop on Chronic Lymphocytic Leukemia (IWCLL) Guidelines

Blood First Edition Paper, prepublished online January 23, 2008; DOI 10.1182/blood-2007-06-093906

Guidelines for the Diagnosis and Treatment of Chronic Lymphocytic Leukemia: A Report from the International Workshop on Chronic Lymphocytic Leukemia (IWCLL) updating the National Cancer Institute-Working Group (NCI-WG) 1996 guidelines

Michael Hallek, Bruce D. Cheson, Daniel Catovsky, Federico Caligaris-Cappio, Guillaume Dighiero, Hartmut Döhner, Peter Hillmen, Michael J. Keating, Emili Montserrat, Kanti R. Rai, Thomas J. Kipps

From the Klinik I für Innere Medizin, Universität zu Köln, Köln, Germany; the Georgetown University Hospital, Lombardi Cancer Center, Washington, DC; the Institute of Cancer Research, London, United Kingdom; the Universita Vita-Salute San Raffaele, Milano, Italy; the Institute Pasteur, Montevideo, Uruguay; the University of Ulm, Ulm, Germany; the Pinderfields Hospital, Wakefield, United Kingdom; the Department. of Leukemia, University of Texas, MD Anderson Cancer Center, Houston, TX; the Hospital Clinic, Barcelona, Spain; the Division of Hematology/Oncology, Long Island Jewish Medical Center, New Hyde Park, NY; and the Rebecca and John Moores Cancer Center, University of California, San Diego, La Jolla, CA.

Address for correspondence:
Michael Hallek, MD
Klinik I für Innere Medizin
Universität zu Köln
Joseph-Stelzmann Str. 9
50924 Köln
Tel.: 0221/478 4400
Fax: 0221/478 5455
E mail: michael.hallek@uni-koeln.de

Copyright © 2008 American Society of Hematology

In 1988 and 1996, a National Cancer Institute-sponsored Working Group (NCI-WG) on chronic lymphocytic leukemia (CLL) published guidelines for the design and conduct of clinical trials for patients with CLL to facilitate comparisons between different treatments and to establish definitions that could be used in scientific studies on the biology of this disease.[1,2] The Food and Drug Administration (FDA) also adopted these guidelines in their evaluation and approval of new drugs. During the last decade, considerable progress has been made in defining new prognostic markers, diagnostic parameters and treatment options, prompting the IWCLL-sponsored Working Group to revise the 1996 criteria (Tables 1-4).

1. Diagnosis of CLL

The World Health Organization (WHO) classification of hematopoietic neoplasias describes CLL as leukemic, lymphocytic lymphoma, being only distinguishable from SLL (small lymphocytic lymphoma) by its leukemic appearance.[3] In the WHO classification CLL is always a disease of neoplastic B-cells, while the entity formerly described as T-CLL is now called T-cell prolymphocytic leukemia (T-PLL).[4]

It is important to verify that the patient has CLL and not some other lymphoproliferative disease that can masquerade as CLL, such as hairy cell leukemia, or leukemic manifestations of mantle cell lymphoma, marginal zone lymphoma, splenic marginal zone lymphoma with circulating villous lymphocytes or follicular lymphoma. To achieve this, it is essential to evaluate the blood count, blood smear, and the immune phenotype of the circulating lymphoid cells (see below,1.1. and 1.2.).

1.1. *Blood*

The diagnosis of CLL requires the presence of ≥ 5000 B-lymphocytes/µL in the peripheral blood for the duration of at least 3 months. The clonality of the circulating B-lymphocytes needs to be confirmed by flow cytometry. The leukemia cells found in the blood smear are characteristically small, mature lymphocytes with a narrow border of cytoplasm and a dense nucleus lacking discernable nucleoli and having partially aggregated chromatin. These cells may be found admixed with larger or atypical cells, cleaved cells or prolymphocytes, which may comprise up to 55% of the blood lymphocytes.[5] Finding prolymphocytes in excess of this percentage would favor a diagnosis of prolymphocytic leukemia (B-cell PLL). Gumprecht nuclear shadows, or smudge cells, found as cell debris, are other characteristic morphologic features found in CLL.

CLL or SLL might be suspected in otherwise healthy adults who have an absolute increase in the clonal B lymphocytes, but who have less than 5000 B-lymphocytes/µL in the blood. However, in the absence of lymphadenopathy or organomegaly (as defined by physical examination and CT scans), cytopenias, or disease-related symptoms, the presence of fewer than 5000 B-lymphocytes per µL blood is defined as "monoclonal B-lymphocytosis" (MBL).[6] The presence of a cytopenia caused by a typical marrow infiltrate defines the diagnosis of CLL regardless of the number of peripheral blood B-lymphocytes or of the lymph node involvement. MBL seems to progress to frank CLL at a rate of 1-2 % per year.[7]

The definition of SLL requires the presence of lymphadenopathy and the absence of cytopenias caused by a clonal marrow infiltrate. Moreover, the number of B-lymphocytes in the peripheral blood should not exceed 5000/µL. In SLL, the diagnosis should be confirmed by histopathological

evaluation of a lymph node biopsy whenever possible.

1.2. Immunophenotype

CLL cells co-express the T-cell antigen CD5 and B-cell surface antigens CD19, CD20, and CD23. The levels of surface immunoglobulin, CD20, and CD79b are characteristically low compared to those found on normal B cells.[8,9] Each clone of leukemia cells is restricted to expression of either kappa or lambda immunoglobulin light chains.[8] Variations of the intensity of expression of these markers may exist and do not prevent inclusion of a patient in clinical trials for CLL.

In contrast, B-cell PLL cells do not express CD5 in half of the cases, and typically express high levels of CD20 and surface Ig.[10] Also, the leukemia cells of mantle cell lymphoma, despite also expressing B cell surface antigens and CD5, generally do not express CD23.

1.3. Other tests performed at diagnosis

The tests described in section 1.3. are not needed to establish the diagnosis of CLL, but may help to predict the prognosis or to assess the tumor burden. With the exception of molecular genetics (FISH), the application of these tests should not be used in routine practice to influence therapy and is not generally recommended. However, certain parameters, such as immunoglobulin mutational status, are useful for predicting the clinical course in individual cases. These tests can be recommended for patients who want a better prediction of the rate at which their disease might progress but it should be emphasized that the indication for treatment does *not* depend on any of these tests but on the clinical stage and the disease activity (see Section 4).

1.3.1 Molecular Genetics

Using interphase fluorescence-in situ hybridization (FISH), cytogenetic lesions can be identified in more than 80% of all CLL cases.[11] The most common deletions are in the long arm of chromosome 13 (del(13q14.1)). Additional, frequent chromosomal aberrations comprise deletions and/or trisomy of chromosome 12, deletions in the long arm of chromosomes 11 (del(11q)) and 6 (del(6q)), and in the short arm of chromosome 17 (del(17p)).[11] When stimulated in vitro, CLL cells can have detectable chromosomal translocations, which are of potential prognostic significance.[12] However, certain translocations can help distinguish other lymphoproliferative diseases from CLL (e.g. t(11;14), which generally is found in mantle cell lymphoma).

There is increasing evidence from prospective clinical trials that detection of certain chromosomal deletions has prognostic significance. Patients with leukemia cells that have del(17p) have an inferior prognosis and appear resistant to standard chemotherapy regimens employing alkylating drugs and/or purine analogues.[13,14] In a retrospective analysis on several chromosomal aberrations as detected by FISH, patients who had CLL cells with chromosomal aberrations del(11q) and del(17p) had an inferior outcome compared to patients who had leukemia cells with a normal karyotype or del(13q) as the sole genetic abnormality.[11] On the other hand, patients with leukemia cells having del(17p) may respond to therapy with alemtuzumab, either alone or in combination with other anti-leukemia agents.[15,16] Detection of these cytogenetic abnormalities has apparent prognostic value and may influence therapeutic decisions. For clinical trials, it is recommended that cytogenetics be performed prior to treating a patient on protocol. Additional genetic defects may be acquired during the course of the disease;[17] therefore, the repetition of FISH analyses seems justified prior to subsequent, second- and third-

line treatment.

1.3.2 Mutational status of IgV_H, VH3.21 usage, and expression of ZAP-70 or CD38

The leukemia cells express immunoglobulin that may or may not have incurred somatic mutations in the immunoglobulin heavy chain variable region genes (IgV_H genes). The outcome of patients with leukemia cells that use an unmutated IgV_H gene is inferior to those patients with leukemia cells that use a mutated IgV_H gene.[18,19] In addition, the VH3.21 gene usage is an unfavorable prognostic marker independent of the IgV_H mutational status.[20] Leukemia-cell expression of ZAP-70 and CD38 was found to correlate with the expression of unmutated IgV_H genes and to predict a poor prognosis.[21-27] However, the association between expression of ZAP-70 or CD38 with the expression of unmutated IgV_H genes is not absolute. It is uncertain whether leukemia-cell expression of unmutated IgV_H genes or ZAP-70 predict the response to treatment or overall survival, once therapy is required.[14,28] Taken together, further clinical trials are needed to standardize the assessment of these parameters and to determine whether they should affect the management of patients with CLL.

1.3.3 Serum markers

Several studies have found that serum markers CD23, thymidine kinase, and $ß_2$-microglobulin may predict survival or progression-free survival.[29-35] Assays for these markers should be standardized and used in prospective clinical trials to validate their relative value to the management of patients with CLL.

1.3.4 Marrow Examination

Characteristically more than 30% of the nucleated cells in the aspirate are lymphoid. Although the type of marrow infiltration (diffuse versus non-diffuse) reflects the tumor burden and provides some prognostic information, recent studies of the German and Spanish study groups suggest that the prognostic value of BM biopsy may now be superseded by new prognostic markers.

A marrow aspirate and biopsy generally are *not* required for the diagnosis of CLL. However, a marrow biopsy and aspirate can help evaluate for factors that might contribute to cytopenias (anemia, thrombocytopenia) that may or may not be directly related to leukemia-cell infiltration of the marrow. Because such factors could influence the susceptibility to drug-induced cytopenias, a marrow biopsy is recommended prior to initiating therapy. It is recommended to repeat a marrow biopsy in patients with persisting cytopenia after treatment to uncover disease- versus therapy-related causes.

2. Clinical Staging

There are two widely accepted staging methods for use in both patient care and clinical trials: the Rai[36] and the Binet system.[37] The original Rai classification was modified to reduce the number of prognostic groups from five to three.[38] As such, both systems now describe three major subgroups with discrete clinical outcomes. These two staging systems are simple, inexpensive, and can be applied by physicians worldwide. Both solely rely on a physical examination and standard laboratory tests, and do not require ultrasound, computed tomography, or magnetic resonance imaging. These two systems are outlined in the following.

2.1. Rai staging system

The modified Rai classification defines low-risk disease as patients who have lymphocytosis with leukemia cells in the blood and/or marrow (lymphoid cells >30%) (formerly considered Rai stage 0). Patients with lymphocytosis, enlarged nodes in any site, and splenomegaly and/or hepatomegaly (lymph nodes being palpable or not) are defined as having intermediate risk disease (formerly considered Rai stage I or stage II). High risk disease includes patients with disease-related anemia (as defined by a hemoglobin (Hb) level less than 11 g/dl) (formerly stage III) or thrombocytopenia (as defined by a platelet count of less than 100 x 10^9/L) (formerly stage IV).

2.2. Binet staging system

Staging is based on the number of involved areas, as defined by the presence of enlarged lymph nodes of greater than 1 cm in diameter or organomegaly, and on whether there is anemia or thrombocytopenia.

Area of involvement considered for staging

(1) Head and neck, including the Waldeyer ring (this counts as one area, even if more than one group of nodes is enlarged).
(2) Axillae (involvement of both axillae counts as one area).
(3) Groins, including superficial femorals (involvement of both groins counts as one area).
(4) Palpable spleen.
(5) Palpable liver (clinically enlarged).

Stage A. Hb ≥ 10 g/dL and platelets ≥ 100 X 10^9/L and up to two of the above involved.

Stage B. Hb ≥ 10 g/dL and platelets ≥ 100 X 10^9/L and organomegaly greater than that defined for stage A (i.e. three or more areas of nodal or organ enlargement).

Stage C. All patients who have Hb of less than 10 g/dL and/or a platelet count of less than 100 x 10^9/L, irrespective of organomegaly.

3. Eligibility Criteria for Clinical Trials

The selection of CLL patients for clinical trials is similar to that for patients with other malignancies. Phase I-II clinical trials commonly, although not invariably, are intended for patients who have had prior therapy. It may be worth considering the inclusion of patients with SLL in some phase I-II trials exploring new agents in CLL. However, for SLL the response assessment should be done according to the lymphoma guidelines. The combination of new agents with standard therapy as part of phase II studies may be investigated in both untreated and previously treated patients. Phase III clinical trials are used to compare the clinical outcome employing new treatment modalities with that using current standard therapy. Other requirements for eligibility with respect to age, clinical stage, performance status, organ function, or status of disease activity should be defined for each study.

3.1. Performance Status and Fitness

Prior to inclusion in a trial, the performance status as defined by the Eastern Cooperative Oncology Group (ECOG) should be 0-3. Future clinical trials involving elderly patients ideally should assess the comorbidity (fitness) and/or functional activity of patients (e.g. such as that defined by “cumulative illness rating scale” (CIRS) or the “Charlson” score).[39,40]

3.2. Organ Function Eligibility for Clinical Trials

Most chemotherapy agents have potential toxicity for the liver, kidneys, heart, lungs, nervous

system, or other organ systems. Therefore, organ function requirements should be guided by the known or suspected toxicity of each agent based on preclinical studies or prior clinical studies. Patients enrolled on protocols evaluating agents with known or suspected toxicity for a given organ(s) should have documented the specific organ function prior to therapy.

3.3. Infectious Disease Status

The status of specific infectious diseases as outlined in section 3.5 should be documented. Patients with active infections requiring systemic antibiotics, antifungal or antiviral drugs should have their infection resolved prior to initiating therapy in a clinical trial.

3.4. Second Malignancies

Patients with a second malignancy, other than basal cell carcinoma of the skin or in situ carcinoma of the cervix or the breast, generally are not considered candidates for entry into clinical trials unless the tumor was successfully treated with curative intent at least 2 years prior to trial entry.

3.5. Required Pretreatment Evaluation

Parameters considered necessary for a complete pretreatment evaluation may differ depending on whether or not the patient is treated in a clinical protocol. Therefore, a clear distinction is made in sections 3.5 and 5 between recommendations for general practice and the requirements for clinical trials (Tables 1, 2, and 3). If not indicated otherwise, recommendations are identical for clinical trials and general practice. In general, studies for defining these parameters should be performed within 2 weeks of clinical trial enrollment (except for marrow aspirate and biopsy and computed tomography (CT) scans (see sections 3.5.1 and 3.5.2).

3.5.1. Essential pretreatment tests (see Table 1)

3.5.1.1. Physical examination: The bidimensional diameters of the largest palpable lymph nodes in each of the following sites should be recorded: cervical, axillary, supraclavicular, inguinal, and femoral. The size of the liver and spleen, as assessed by palpation, should also be recorded.

3.5.1.2. Assessment of performance status (ECOG score).

3.5.1.3. A complete blood cell count (CBC; white blood cell count, hemoglobin and hematocrit, platelet count) and differential count, including both percent and absolute number of lymphocytes, and reticulocyte count should be performed. Reporting the proportion of prolymphocytes is desirable when these are present.

3.5.1.4. Marrow biopsy: Prior to initiating treatment in a clinical trial with potentially myelosuppressive agents, patients should undergo a unilateral marrow aspirate and biopsy. Repeat marrow biopsies may be compared with the pre-treatment marrow specimen.

3.5.1.5. Serum chemistry (e.g., creatinine, bilirubin, LDH, transaminases, alkaline phosphatase).

3.5.1.6. Serum immunoglobulin levels.

3.5.1.7. Direct antiglobulin test (DAT).

3.5.1.8. Chest radiograph.

3.5.1.9. Human immunodeficiency virus (HIV)

Patients who are infected with HIV should be given special consideration because of the potential

risks for immune suppression with most anti-leukemia therapies and the potential for compounded myelotoxicity of treatment with anti-retroviral therapy.

3.5.1.10. Cytomegalovirus (CMV)

Therapies associated with the potential for reactivation of infection with CMV, such as alemtuzumab or allogeneic stem cell transplantation, should include plans for monitoring for active CMV disease and/or for providing anti-CMV therapy.[41] These should cover screening or early diagnosis of CMV reactivation and its subsequent management. However, a positive CMV serology does not represent a contraindication for alemtuzumab treatment or allogeneic stem cell transplantation. As a general recommendation for patients treated with alemtuzumab, close monitoring and/or therapy for active CMV disease should be considered for patients found to have evidence for increased levels of CMV in the blood by the polymerase chain reaction (PCR), even in the absence of clinical symptoms. Also, evaluation and therapy for CMV is recommended for any patient with clinical symptoms of active CMV infection.

3.5.1.11. Hepatitis B and Hepatitis C

Before initiating treatment, the evaluation for infection with hepatitis B virus (HBV) and hepatitis C virus (HCV) is recommended, since reactivation of HBV and HCV infections may occur under therapy with immunosuppressive or myelosuppressive drugs. Chronic HBV carriers as defined by positive surface antigen (HBsAg) undergoing chemotherapy should receive prophylactic therapy with nucleoside analogs such as lamivudine to prevent HBV reactivation.[42,43]

3.5.2 Additional pretreatment tests (Table 1)

The following tests may be performed in clinical trials or in the presence of specific clinical problems.

3.5.2.1. The assessment of molecular cytogenetics (FISH) prior to therapy is recommended.

3.5.2.2. Computed tomography (CT) scans: CT scans generally are *not* required for the initial evaluation, or follow up. Moreover, the staging of CLL does not use CT scans but relies on physical examination and blood counts. A recent study has found that patients in Rai stage 0 but with detectable abdominal disease by CT scans may have a more aggressive disease.[44] Therefore, clinical studies evaluating the use of CT scans in CLL are strongly encouraged. Moreover, enlarged lymph nodes if detected only by CT scan do not change the clinical Binet or Rai stage.

In *clinical trials* where the treatment intent is to maximize complete remissions chest, abdominal and pelvic CT scans are recommended to evaluate the response to therapy. CT scans should be performed prior to the start of therapy and at the first restaging following therapy if previously abnormal.

3.5.2.3. Other imaging methods: Except in some patients with Richter’s transformation, positron emission tomography (PET) scans do *not* provide information that is useful in the management of CLL. Similarly, nuclear magnetic resonance imaging and other imaging techniques are generally *not* useful in the management of CLL.

3.5.2.4. Abdominal ultrasound: In some countries, the use of abdominal ultrasound is popular to assess the extent of lymphadenopathy and organomegaly in CLL. While it may be used in the clinical management of individual patients, this methodology is strongly investigator-dependent and should therefore not be used for the response evaluation in clinical trials.

3.5.2.5. A lymph node biopsy is generally *not* required, unless such tissue is necessary for companion scientific studies or in rare cases with difficult diagnosis. A lymph node biopsy is

requested to establish the diagnosis of a transformation into an aggressive lymphoma (Richter's syndrome).

4. Indications for Treatment

4.1. *Primary Treatment Decisions (Table 2)*

Criteria for initiating treatment may vary depending on whether or not the patient is treated in a clinical trial. In general practice, newly diagnosed patients with asymptomatic early-stage disease (Rai 0, Binet A), should be monitored without therapy unless they have evidence of disease progression. Studies from both the French Cooperative Group on CLL,[45] the Cancer and Leukemia Group B (CALGB),[46] the Spanish Group Pethema,[47] and the Medical Research Council (MRC)[48] in the UK in patients with early-stage disease confirm that the use of alkylating agents in patients with such early-stage disease does not prolong surviva1. This result was confirmed by a meta-analysis.[49] In one study, treated patients with early-stage disease had an increased frequency of fatal epithelial cancers compared to untreated patients.[45] Therefore, the potential benefit, if any, of an early-intervention therapy with anti-leukemia drugs, alone or in combination with monoclonal antibodies, requires further study.

Whereas patients at intermediate (stage I and II) and high risk (stage III and IV) according to the modified Rai classification or at Binet stage B or C usually benefit from the initiation of treatment, some of these patients (in particular Rai intermediate risk or Binet stage B) can be monitored without therapy until they have evidence for progressive or symptomatic disease.

Active disease should be clearly documented for protocol therapy. At least one of the following criteria should be met:

(1) Evidence of progressive marrow failure as manifested by the development of, or worsening of, anemia and/or thrombocytopenia

(2) Massive (i.e., > 6 cm below the left costal margin) or progressive or symptomatic splenomegaly

(3) Massive nodes (i.e., >10 cm in longest diameter) or progressive or symptomatic lymphadenopathy

(4) Progressive lymphocytosis with an increase of >50% over a 2-month period, or lymphocyte doubling time (LDT) of less than 6 months. LDT can be obtained by linear regression extrapolation of absolute lymphocyte counts (ALC) obtained at intervals of two weeks over an observation period of 2-3 months; patients with initial blood lymphocyte counts of less than 30.000/µl may require a longer observation period to determine the LDT. Also, factors contributing to lymphocytosis or lymphadenopathy other than CLL (e.g., infections) should be excluded.

(5) Autoimmune anemia and/or thrombocytopenia poorly responsive to corticosteroids or other standard therapy (see 10.2).

(6) A minimum of any one of the following disease-related symptoms must be present:

    (a) Unintentional weight loss ≥ 10% within the previous 6 months.

    (b) Significant fatigue (i.e., ECOG PS 2 or worse; cannot work or unable to perform usual activities).

    (c) Fevers of greater than 100.5° F or 38.0° C for 2 or more weeks without other evidence of infection.

    (d) Night sweats for more than 1 month without evidence of infection.

Hypogammaglobulinemia or monoclonal or oligoclonal paraproteinemia does not by itself constitute a basis for initiating therapy. However it is recommended to assess the change of these protein abnormalities, if patients are treated.

Patients with CLL may present with a markedly elevated leukocyte count; however, the symptoms associated with leukocyte aggregates that develop in patients with acute leukemia rarely occur in patients with CLL. Therefore, the absolute lymphocyte count should not be used as the sole indicator for treatment

4.2. *Second-Line Treatment Decisions*

In general, second-line treatment decisions follow the same indications as those used for initiation of first-line treatment. Patients who have resistant disease, a short time to progression after the first treatment, and/or leukemia cells with del(17p) often do not respond to standard chemotherapy and have a relatively short survival. Therefore, such patients should be offered investigative clinical protocols including allogeneic hematopoietic stem cell transplantation.[50-54]

5. Definition of Response, Relapse and Refractory Disease (Tables 3 and 4)

Assessment of response should include a careful physical examination and evaluation of the blood and marrow. Imaging studies, in particular CT scans, generally are not required except to monitor the response to therapy in clinical trials (Table 3).

5.1. Complete remission (CR) requires all of the following criteria as assessed at least 3 months after completion of therapy:

5.1.1. Absence of clonal lymphocytes in the peripheral blood (as defined in section 1.1 and 1.2).

5.1.2. Absence of significant lymphadenopathy (e.g. lymph nodes greater than 1,5 cm in diameter) by physical examination. In clinical trials a CT scan of the abdomen, pelvis and thorax should be performed if previously abnormal. Lymph nodes should not be larger than 1,5 cm in diameter.

5.1.3. No hepatomegaly or splenomegaly by physical examination. In clinical trials a CT scan of the abdomen should be performed at response assessment if found to be abnormal prior to therapy or if physical exam is inconclusive at the time of evaluation.

5.1.4. Absence of constitutional symptoms.

5.1.5. Blood counts above the following values:

5.1.5.1. Polymorphonuclear leukocytes ≥ 1.500/µL.

5.1.5.2. Platelets > 100.000/µL.

5.1.5.3. Hemoglobin > 11,0 g/dL (untransfused).

5.1.6. For patients in clinical trials (Table 3): a marrow aspirate and biopsy should be performed at least 3 months after the last treatment and if clinical and laboratory results listed in 5.1.1 to 5.1.5 demonstrate that a CR has been achieved. The marrow should be analyzed by flow cytometry and/or immunohistochemistry to demonstrate that the marrow is free of clonal B-CLL cells. Cases with residual CLL cells by conventional (not 4-colour; see below) flow cytometry or immunohistochemistry are defined as PR.

In some cases, lymphoid nodules can be found (formerly used to define nodular PR), which often reflect residual disease.[55,56] Therefore, these nodules should be assessed by immunohistochemistry to define whether they are comprised primarily of T cells or lymphocytes other than CLL cells or of CLL cells. The category of “nodular PR” should no longer be used. If the marrow is found to be hypocellular, a repeat marrow biopsy should be performed after 4-6 weeks, provided that the blood counts have recovered, as defined in 5.1.5. Marrow biopsies should be compared with that of the pre-treatment marrow. In some cases, it is necessary to postpone the marrow biopsy until after all the other criteria to define a CR (5.1.1 to 5.1.5) have been satisfied. However, this time interval should not exceed 6 months after the last treatment.

5.1.7. A controversial issue is how best to categorize the response of patients who fulfill all the criteria for a CR (including the marrow examinations described in 5.16), but who have a persistent anemia or thrombocytopenia or neutropenia apparently unrelated to CLL, but related to drug toxicity. We recommend that these patients should be considered as a different category of remission, CR with incomplete bone marrow recovery (CRi). For the definition of this category, CRi, the marrow evaluation (see 5.1.6.) should be performed with scrutiny and not show any clonal infiltrate. In clinical trials, CRi patients should be monitored prospectively to determine whether their outcome differs from that of patients with detectable residual disease or with non-cytopenic CR.

5.2. Partial remission (PR) is defined by the criteria described in 5.21, 5.22, and/or 5.23 (if abnormal prior to therapy), as well as one or more of the features listed in 5.24. To define a PR at least one of these parameters needs to be documented for a minimal duration of 2 months (Table 4). Constitutional symptoms persisting for more than 1 month should also be documented.

5.2.1. A decrease in the number of blood lymphocytes by below 50% or more from the value prior to therapy.

5.2.2. Reduction in lymphadenopathy (to be assessed by CT scans in clinical trials[57] and by palpation in general practice) as defined by:

5.2.2.1 A decrease lymph node size by below 50% or more in

- the sum products of up to 6 lymph nodes, or
- in one lymph node diameter if only a single lymph node was present prior to therapy.

5.2.2.2 No increase in any lymph node, and no new enlarged lymph node. In small lymph nodes (< 2 cm), an increase of < 25% is not considered to be significant.

5.2.3. A decrease in the size of the liver and/or spleen by 50% more as defined by CT scan in clinical trials or by palpation or ultrasound in general practice.

5.2.4. The blood count should show one of the following results:

5.2.4.1. Polymorphonuclear leukocytes at 1.500/µL or more or 50% improvement over baseline without G-CSF support.

5.2.4.2. Platelet counts greater than 100.000/µL or 50% improvement over baseline.

5.2.4.3. Hemoglobin greater than 11,0 g/dL or 50% improvement over baseline without red blood cell transfusions or erythropoietin support.

5.3. Progressive disease is characterized by at least one of the following:

5.3.1. Lymphadenopathy: progression of lymphadenopathy is discovered almost uniformly by blood counts. Therefore, imaging methods are not needed to follow CLL progression. Progression of lymphadenopathy may be documented by physical examination. Disease progression occurs, if one of the following events is observed:

- Appearance of any new lesion such as enlarged lymph nodes (> 1,5 cm), splenomegaly, hepatomegaly or other organ infiltrates.
- An increase by 50% or more in greatest determined diameter of any previous site. A lymph node of 1-1,5 cm must increase by 50% or more to a size greater than 1,5 cm in the longest axis. A lymph node of more than 1,5 cm must increase to more than 2,0 cm in the longest axis.
- An increase of 50% or more in the sum of the product of diameters of multiple nodes.

5.3.2. An increase in the liver or spleen size by 50% or more or the de novo appearance of hepatomegaly or splenomegaly.

5.3.3. An increase in the number of blood lymphocytes by 50% or more with at least 5,000 B lymphocytes per µL.

5.3.4. Transformation to a more aggressive histology (e.g. Richter's syndrome). Whenever possible, this diagnosis should be established by lymph node biopsy.

5.3.5. Occurrence of cytopenia (neutropenia, anemia or thrombocytopenia) attributable to CLL.

5.3.5.1 During therapy: cytopenias may occur as a side effect of many therapies and should be assessed according to Table 5. During therapy, cytopenias cannot be used to define disease progression. Each protocol should define the amount of drug(s) to be administered with such cytopenias.

5.3.5.2. Post treatment: The progression of any cytopenia (unrelated to autoimmune cytopenia), as documented by a decrease of Hb levels by more than 2 g/dl or to less than 10 g/dl, or by a decrease of platelet counts by more than 50% or to less than 100.000/µl, which occurs at least 3 months after treatment, defines disease progression, if the marrow biopsy demonstrates an infiltrate of clonal CLL cells.

5.4. Stable disease: Patients who have not achieved a CR or a PR, and who have not exhibited PD, will be considered to have stable disease (which is equivalent to a non-response).

5.5. Responses that should be considered clinically beneficial include CR and PR; all others (e.g. stable disease, non-response, progressive disease, or death from any cause) should be rated as a treatment failure.

5.6. Duration of response and progression-free survival

Duration of response should be measured from the end of the last treatment until evidence of progressive disease (as defined above). Progression-free survival (PFS) is defined as the interval between the first treatment day to the first sign of disease progression. Event-free survival is defined as the interval between the first treatment day to the first sign of disease progression, or treatment for relapse, or death (whichever occurs first). Survival duration is defined as the interval between the first treatment day to death.

5.7. Relapse

Relapse is defined as a patient who has previously achieved the above criteria (5.1-5.2) of a CR or PR, but *after* a period of 6 or more months, demonstrate evidence of disease progression (see 5.3).

5.8. Refractory disease

[0]Refractory disease is defined as treatment failure (as defined in 5.5) or disease progression *within* 6 months to the last anti-leukemic therapy. For the definition of "high risk CLL" justifying the use of allogeneic stem cell transplantation,[58] the disease should be refractory to a purine-analogue based therapy or to autologous hematopoietic stem cell transplantation.

5.9. Minimal residual disease

The complete eradication of the leukemia is an obvious desired endpoint. New detection technologies such as multicolor flow cytometry and real-time quantitative PCR have determined that many patients who achieved a complete response by the 1996 NCI-WG guidelines have detectable minimal residual disease (MRD). While eradication of MRD may improve prognosis, prospective clinical trials are needed to define whether additional treatment intended solely to eradicate MRD provides a significant benefit to clinical outcome. The techniques for assessing MRD have undergone a critical evaluation and have become fairly standard.[59] Either four-color flow cytometry (MRD Flow) or allele-specific oligonucleotide PCR are reliably sensitive down to a level of approximately one CLL cell in 10.000 leukocytes. As such, patients will be defined as having a clinical remission in the absence of MRD when they have blood or marrow with less than one CLL cell per 10.000 leucocytes. The blood generally can be used for making this assessment except during the period within 3 months of completing therapy, particularly for patients treated with alemtuzumab, rituximab and other antibodies targeting CLL. In such cases it is essential to assess the marrow for MRD. Therefore, future clinical trials that aim toward achieving long-lasting complete remissions should include at least one test to assess MRD, because the lack of leukemia persistence using these sensitive tests seems to have a strong, positive prognostic impact [60-62].

6. Factors Requiring Stratification at Inclusion in a Clinical Phase III Trial

6.1. Patients ideally should be stratified with regard to previous treatment versus no previous treatment, and as purine analogue-sensitive versus purine-analogue refractory in studies for which prior therapy is allowed.

6.2. If more than one clinical stage is allowed, patients ideally should be stratified for stage.

6.3. Patients ideally should be stratified based upon whether or not they have leukemia cells with del(17p) or del(11q).

7. Assessment of Toxicity

Evaluation of treatment-related toxicity requires careful consideration of both the manifestations of the underlying disease and the anticipated adverse reactions to the agents used in therapy. For this reason, some of the conventional criteria used for assessing toxicity are not applicable to clinical studies involving patients with hematological malignancies in general, or CLL in particular. An example is hematological toxicity; patients with advanced CLL generally have cytopenias that may be caused by the underlying CLL and/or prior therapy. A few guidelines are presented to help evaluate for treatment-induced toxicity in CLL.

7.1. *Hematological Toxicity*

Evaluation of hematological toxicity in patients with CLL must take into consideration that many patients have low blood cell counts at the initiation of therapy. Therefore, the standard criteria used for solid tumors cannot be applied, as many CLL patients then would be considered to have grade II to IV hematological toxicity at the initiation of treatment. Furthermore, the absolute

blood neutrophil counts rarely are used at the initiation of therapy to modify the treatment dose, since these values typically are unreliable in CLL patients with lymphocytosis. However, the increasing use of more effective therapeutic agents, particularly those with neutropenia as a dose-limiting toxicity (e.g. nucleoside analogs), can result in clinically significant myelosuppression. Therefore, the 1996 guidelines proposed a new dose-modification scheme for quantifying hematological deterioration in patients with CLL, which included alterations in the dose of myelosuppressive agents based on the absolute neutrophil count. This dose modification scheme has proven very helpful in the context of several large prospective trials in CLL and should be retained (Table 5).

7.2. Infectious Complications

Patients with CLL are at increased risk for infection because of compromised immune function, which might be related to the disease itself and/or to the consequences of therapy. Nevertheless, the rate(s) of infection following treatment can be used in assessing the relative immune-suppressive effects of a given therapy. The etiology of the infection should be reported and categorized as bacterial, viral, or fungal, and as proven or probable. The severity of infections should be quantified as minor (requiring either oral antimicrobial therapy or symptomatic care alone), major (requiring hospitalization and systemic antimicrobial therapy), or fatal (death as a result of the infection).

Particular attention should be given to monitoring for symptoms or laboratory evidence of infection with CMV in patients treated with agents like alemtuzumab (alone or in combination) or with allogeneic stem cell transplantation. In contrast, the infection rate seems low in patients younger than 65 years treated with fludarabine-based first line therapy, where no monitoring or routine anti-infective prophylaxis is required.[63]

7.3. Tumor lysis syndrome

CLL patients rarely experience tumor lysis syndrome after therapy with a purine analogue based regimen.[64] However, this might not be the case following treatment with newer agents or novel treatment modalities. For this reason, patients in early phase clinical trials should be monitored for possible tumor lysis syndrome, which should be treated appropriately. If observed, the occurrence and severity of tumor lysis syndrome should be recorded in clinical trials.

7.4. Non-hematological Toxicities

Other non-hematological toxicities should be graded according to the latest version of the NCI Common Toxicity Criteria (CTC).

8. Reporting of Clinical Response Data

Clear and careful reporting of data is an essential part of any clinical trial. In clinical studies involving previously treated patients, patients who are relapsed or refractory should be clearly distinguished. Relapse and refractory disease are defined above (5.7 and 5.8). For those patients who have relapsed, it is also useful to describe the quality and duration of their prior response.

9. Treatment endpoints

Given the recent increase of treatment options for CLL patients, the choice of treatment and the endpoints of clinical trials may depend on the fitness of the patients (see 3.1). For example, the number of MRD negative complete remissions or the overall survival might be appropriate endpoints in physically fit patients. In contrast, trials on patients with reduced physical fitness might choose the time to progression or health-related quality of life as trial endpoints. Moreover, recent data suggest that the quality of life in CLL patients is reduced as compared to the normal population and only moderately increased by some of the current treatment options.[65-68] Therefore, further studies assessing the health-related quality of life in CLL are strongly encouraged.

10. Supportive care and management of complications

10.1. Indications for growth factors in CLL

While under myelosuppressive (chemo-)therapy, growth factors such as granulocyte-colony-stimulating factor (G-CSF) should be given according to the guidelines of the American Society of Clinical Onclogy.[69] The use of G-CSF also might benefit patients who experience prolonged cytopenias following treatment with alemtuzumab. Similarly, some CLL patients with anemia may benefit from erythropoiesis stimulating factors, if used according to recently published guidelines.[70,71] However, it should be pointed out that CLL-related cytopenias are often efficiently corrected by an appropriate anti-leukemic therapy.

10.2. Autoimmune hemolytic anemia (AIHA) or autoimmune thrombocytopenia (ITP)

ITP and AIHA as a single abnormality caused by CLL initially should be treated with glucocorticoids and not chemotherapy. Second line treatment options for AIHA include splenectomy, intravenous immunoglobulins, and/or immunosuppressive therapy with agents such as cyclosporine A, azathioprin, or low-dose cyclophosphamide. Good responses also have been obtained with antibody therapy using agents as rituximab or alemtuzumab.[72-74] Treatment refractory autoimmune cytopenias can be an indication for chemotherapy or chemoimmunotherapy directed at the underlying CLL.[75] In this regard, the Binet or Rai staging systems do not distinguish between ITP/AIHA or marrow infiltration as the cause for anemia or thrombocytopenia that results in classifying a patient as having stage C or high-risk disease.

ACKNOWLEDGMENT

This work was supported in part by the Competence Network Malignant Lymphoma of the German Ministery for Education and Research (BMBF).

The following colleagues are credited for their active participation in the original version of the Guidelines: Charles A. Schiffer, Martin M. Oken, David H. Boldt, Sanford J. Kempin, and Kenneth A. Foon. The following colleagues are credited for their active participation of the second, 1996 version of the guidelines: John M. Bennett, Michael Grever, Neil Kay, and Susan O'Brien.

We wish to thank the following colleagues for reading and commenting on the current version of the guidelines: Drs Francesc Bosch (Barcelona, Spain), John Byrd (Columbus, Ohio, USA), Barbara Eichhorst (Cologne, Germany), Terry Hamblin (London, UK), Neil Kay (Rochester, Minnesota, USA), Eva Kimby (Stockholm, Sweden), Estella Matutes (London , UK), Stefano Molica (Catanzaro, Italy), Stephen Mulligan (Sydney, Australia), Susan O'Brien (Houston, Texas, USA), David Oscier (Bournemouth, UK), John Seymour (Melbourne, Australia).

We acknowledge the support of the German Cancer Aid (Deutsche Krebshilfe), the Kompetenznetz Maligne Lymphome (Competence Network Malignant Lymphoma) of the German Ministry for Education and Research (BMBF) and the European Leukemia Network (ELN) to Michael Hallek and Hartmut Döhner, of the National Institutes of Health PO1-CA081534 to the CLL Research Consortium (CRC), the Arbib Foundation to Daniel Catovsky, the Karches Family Foundation, Prince Family Foundation and the Nash Family Foundation to Kanti R. Rai.

Authorship

Michael Hallek, Bruce D. Cheson, Daniel Catovsky, Federico Caligaris-Cappio, Guillaume Dighiero, Hartmut Döhner, Peter Hillmen, Michael Keating, Emili Montserrat, Kanti R. Rai, Thomas J. Kipps all wrote the paper.

Conflict of Interest Disclosure
The authors declare no competing financial interests.

TABLES

Table 1. Pretreatment Evaluation of Patients with CLL

Diagnostic test	Section of guidelines	General practice	Clinical trial
Tests to establish the diagnosis	1.		
Complete blood count and differential count	1.1	Always	Always
Immunophenotyping of lymphocytes	1.2.	Always	Always
Assessment prior to treatment	3.5.1		
History and physical, performance status	3.5.1.1, 3.5.1.2	Always	Always
Complete blood count and differential	3.5.1.3	Always	Always
Marrow aspirate and biopsy	3.5.1.4	Desirable	Desirable
Serum chemistry, serum immunoglobulin, direct antiglobulin test	3.5.1.5, 3.5.1.6, 3.5.1.7	Always	Always
Chest radiograph	3.5.1.8	Always	Always
Infectious disease status	3.3	Always	Always
Additional tests prior to treatment	3.5.2		
Cytogenetics (FISH) for del(13q), del(11q), del(17p), add(12), del(6q) in the peripheral blood lymphocytes	3.5.2.1	Desirable	Always
IgVH mutational status, ZAP-70, and CD38	1.2	No	Always
CT scan of chest, abdomen, and pelvis	3.5.2.2	No	Desirable; always if CR is the desired endpoint.
MRI, Lymphangiogram, gallium scan, PET scans	3.5.2.3	No	No
Abdominal ultrasound	3.5.2.4	Possible	No

General practice is defined as the use of accepted treatment options for a CLL patient not enrolled on a clinical trial.

Abbreviations: No, not generally indicated; RQ, indicated if a research question; PBS, peripheral blood smear; MRI, magnetic resonance imaging; PET, positron emission tomography; FISH, fluorescence in situ hybridization.

Table 2. Recommendations Regarding Indications for Treatment in CLL

	General practice	Clinical trial
Treat with Rai stage 0	No*	RQ
Treat with Binet stage A	No*	RQ
Treat with Binet stage B or Rai stage I or Rai stage II	Possible*	Possible*
Treat with Binet stage C or Rai stage III or Rai stage IV	Yes	Yes
[redacted]	[redacted]	[redacted]
Treatment of active/progressive disease	Yes	Yes
Treat without active/progressive disease	No	RQ

General practice is defined as the use of accepted treatment options for a CLL patient not enrolled on a clinical trial.

Abbreviations: No, not generally indicated; RQ, indicated if a research question.

*Treatment is indicated, if the disease is active as defined in section 4.

Table 3. Recommendations regarding the response assessment in CLL patients

Diagnostic test	Section of guidelines	General practice	Clinical trial
History, physical examination	5.1.2, 5.1.3, 5.1.4, 5.2.2, 5.2.3, 5.3.1, 5.3.2	Always	Always
Immunophenotyping of peripheral blood lymphocytes	5.1.1	If clinical and hematological response indicates CR	If clinical response and hematological response indicates CR
CBC and differential count	5.1.5, 5.2.4, 5.3.3, 5.3.5	Always	Always
Marrow aspirate and biopsy	5.1.6	At cytopenia of uncertain cause	At CR or cytopenia of uncertain cause
Assessment for minimal residual disease	5.9	No	If a long-lasting CR is the desired endpoint
Ultrasound of the abdomen	5.1.2, 5.1.3, 5.2.2, 5.2.3, 5.3.1, 5.3.2	Possible, if previously abnormal	No
CT scans of chest, pelvis, and abdomen	5.1.2, 5.1.3, 5.2.2, 5.2.3, 5.3.1, 5.3.2	No	Indicated if previously abnormal and otherwise in CR

General practice is defined as the use of accepted treatment options for a CLL patient not enrolled on a clinical trial.

Table 4 - Response definition after treatment for CLL patients, using the parameters of Tables 1 and 3

Parameter	CR	PR	PD	SD
Lymphadenopathy [1)]	None above 1,5 cm	Decrease ≥ 50%	Increase ≥ 50%	Change of -49% to +49%
Liver and/or spleen size	Normal size	Decrease ≥ 50%	Increase ≥ 50%	Change of -49% to +49%
Constitutional symptoms	None	Any	Any	Any
Polymorphonuclear leukocytes	> 1500/µl	> 1500/µl or > 50% improvement over baseline	Any	Any
Circulating clonal B-lymphocytes	Nil	Decrease ≥ 50% from baseline	Increase ≥ 50% over baseline	Change of -49% to +49%
Platelet count	> 100.000/µl	> 100.000/µl or increase ≥ 50% over baseline	Decrease of ≥ 50% from baseline secondary to CLL	Change of -49 to +49%
Hemoglobin	> 11,0 g/dl (untransfused and without erythropoietin)	> 11 g/dl or increase ≥ 50% over baseline	Decrease of > 2 g/dl from baseline secondary to CLL	Increase < 11,0 g/dl or < 50% over baseline, or decrease < 2 g/dl
Marrow	Normocellular, < 30% lymphocytes, no B-lymphoid nodules. Hypocellular marrow defines CRi (5.1.6.).	≥ 30% lymphocytes, or B-lymphoid nodules, or not done	Increase of lymphocytes to more than 30% from normal	No change in marrow infiltrate

1) sum of the products of multiple lymph nodes (as evaluated by CT scans in clinical trials, or by physical exam or ultrasound in general practice).

CR: complete remission, all of the criteria have to be met; PR: partial remission, at least one of the criteria has to be met; PD: progressive disease: at least one of the above criteria has to be met; SD; all of the above criteria have to be met.

Table 5. Grading Scale for Hematological Toxicity in CLL Studies

Grade[#]	Decrease in Platelets* or Hb° (nadir) From Pretreatment value (%)	absolute neutrophil count/µL§ (nadir)
0	No change to 10%	≥ 2,000
1	11%-24%	≥ 1,500 and < 2,000
2	25%-49%	≥ 1,000 and < 1,500
3	50%-74%	≥ 500 and < 1,000
4	≥ 75%	< 500

* Platelet counts must be below normal levels for grades 1-4. If, at any level of decrease the platelet count is <20.000/µL, this will be considered grade 4 toxicity, unless a severe or life-threatening decrease in the initial platelet count (e.g., 20.000/µL) was present pretreatment, in which case the patient is not evaluable for toxicity referable to platelet counts.

° Hb levels must be below normal levels for grades 1-4. Baseline and subsequent Hb determinations must be performed before any given transfusions. The use of erythropoietin is irrelevant for the grading of toxicity, but should be documented.

# Grades: 1, mild; 2, moderate; 3, severe; 4, life-threatening; 5, fatal. Death occurring as a result of toxicity at any level of decrease from pretreatment will be recorded as grade 5.

§ If the absolute neutrophil count (ANC) reaches less than 1,000/µL, it should be judged to be grade 3 toxicity. Other decreases in the white blood cell count, or in circulating granulocytes, are not to be considered, since a decrease in the white blood cell count is a desired therapeutic end point. A gradual decrease in granulocytes is not a reliable index in CLL for stepwise grading of toxicity. If the ANC was less than 1,000/µL prior to therapy, the patient is not evaluable for toxicity referable to the ANC. The use of G-CSF is irrelevant for the grading of toxicity, but should be documented.

References

1. Cheson BD, Bennett JM, Rai KR, et al. Guidelines for clinical protocols for chronic lymphocytic leukemia (CLL). Recommendations of the NCI-sponsored working group. Am J Hematol. 1988;29:153-163.
2. Cheson BD, Bennett JM, Grever M, et al. National Cancer Institute-Sponsored Working Group guidelines for chronic lymphocytic leukemia: revised guidelines for diagnosis and treatment. Blood. 1996;87:4990-4997.
3. Müller-Hermelink HK, Montserrat E, Catovsky D, Harris NL. Chronic lymphocytic leukemia/small lymphocytic lymphoma. In: Jaffe ES, Harris NL, Stein H, Vardiman JW, eds. World Health Organization Classification of Tumours Pathology and Genetics of Tumours of Haematopoietic and Lymphoid Tissues. Lyon: IARCPress; 2001:127-130.
4. Catovsky D, Ralfkiaer E, Müller-Hermelink HK. T-cell prolymphocytic leukaemia. In: Jaffe ES, Harris NL, Stein H, Vardiman JW, eds. World Health Organization Classification of Tumours Pathology and Genetics of Tumours of Haematopoietic and Lymphoid Tissues. Lyon: IARCPress; 2001:195--196.
5. Melo JV, Catovsky D, Galton DAG. The relationship between chronic lymphocytic leukaemia and prolymphocytic leukaemia. IV. Analysis of survival and prognostic features. Br J Haematol. 1986;63:377-387.
6. Marti GE, Rawstron AC, Ghia P, et al. Diagnostic criteria for monoclonal B-cell lymphocytosis. Br J Haematol. 2005;130:325-332.
7. Rawstron AC, Bennett FL, M. O’Connor SJM, et al. Monoclonal B-cell Lymphocytosis (MBL): a precursor state for Chronic Lymphocytic Leukemia (CLL). 2007:submitted.
8. Moreau EJ, Matutes E, A'Hern RP, et al. Improvement of the chronic lymphocytic leukemia scoring system with the monoclonal antibody SN8 (CD79b). Am J Clin Pathol. 1997;108:378-382.
9. Ginaldi L, De Martinis M, Matutes E, Farahat N, Morilla R, Catovsky D. Levels of expression of CD19 and CD20 in chronic B cell leukaemias. J Clin Pathol. 1998;51:364-369.
10. Catovsky D, Müller-Hermelink HK, Montserrat E, Harris NL. B-cell prolymphocytic leukaemia. In: Jaffe ES, Harris NL, Stein H, Vardiman JW, eds. World Health Organization Classification of Tumours Pathology and Genetics of Tumours of Haematopoietic and Lymphoid Tissues. Lyon: IARCPress; 2001:131-132.
11. Döhner H, Stilgenbauer S, Benner A, et al. Genomic Aberrations and Survival in Chronic Lymphocytic Leukemia. N Engl J Med. 2000;343:1910-1916.
12. Mayr C, Speicher MR, Kofler DM, et al. Chromosomal translocations are associated with poor prognosis in chronic lymphocytic leukemia. Blood. 2006;107:742-751.
13. Döhner H, Fischer K, Bentz M, et al. p53 gene deletion predicts for poor survival and non-response to therapy with purine analogs in chronic B-cell leukemias. Blood. 1995;85:1580-1589.
14. Grever MR, Lucas DM, Dewald GW, et al. Comprehensive assessment of genetic and molecular features predicting outcome in patients with chronic lymphocytic leukemia: results from the US Intergroup Phase III Trial E2997. J Clin Oncol. 2007;25:799-804.
15. Stilgenbauer S, Dohner H. Campath-1H-induced complete remission of chronic lymphocytic leukemia despite p53 gene mutation and resistance to chemotherapy. N Engl J Med. 2002;347:452-453.
16. Lozanski G, Heerema NA, Flinn IW, et al. Alemtuzumab is an effective therapy for chronic lymphocytic leukemia with p53 mutations and deletions. Blood. 2004;103:3278-3281.

17. Shanafelt TD, Witzig TE, Fink SR, et al. Prospective evaluation of clonal evolution during long-term follow-up of patients with untreated early-stage chronic lymphocytic leukemia. J Clin Oncol. 2006;24:4634-4641.
18. Damle RN, Wasil T, Fais F, et al. Ig V gene mutation status and CD38 expression as novel prognostic indicators in chronic lymphocytic leukemia [see comments]. Blood. 1999;94:1840-1847.
19. Hamblin TJ, Davis Z, Gardiner A, Oscier DG, Stevenson FK. Unmutated Ig V(H) genes are associated with a more aggressive form of chronic lymphocytic leukemia [see comments]. Blood. 1999;94:1848-1854.
20. Thorselius M, Krober A, Murray F, et al. Strikingly homologous immunoglobulin gene rearrangements and poor outcome in VH3-21-using chronic lymphocytic leukemia patients independent of geographic origin and mutational status. Blood. 2006;107:2889-2894.
21. Orchard JA, Ibbotson RE, Davis Z, et al. ZAP-70 expression and prognosis in chronic lymphocytic leukaemia. Lancet. 2004;363:105-111.
22. Crespo M, Bosch F, Villamor N, et al. ZAP-70 expression as a surrogate for immunoglobulin-variable-region mutations in chronic lymphocytic leukemia. N Engl J Med. 2003;348:1764-1775.
23. Rassenti LZ, Huynh L, Toy TL, et al. ZAP-70 compared with immunoglobulin heavy-chain gene mutation status as a predictor of disease progression in chronic lymphocytic leukemia. N Engl J Med. 2004;351:893-901.
24. Ibrahim S, Keating M, Do KA, et al. CD38 expression as an important prognostic factor in B-cell chronic lymphocytic leukemia. Blood. 2001;98:181-186.
25. Lin K, Sherrington PD, Dennis M, Matrai Z, Cawley JC, Pettitt AR. Relationship between p53 dysfunction, CD38 expression, and IgV(H) mutation in chronic lymphocytic leukemia. Blood. 2002;100:1404-1409.
26. Ghia P, Guida G, Stella S, et al. The pattern of CD38 expression defines a distinct subset of chronic lymphocytic leukemia (CLL) patients at risk of disease progression. Blood. 2003;101:1262-1269.
27. Boonstra JG, van Lom K, Langerak AW, et al. CD38 as a prognostic factor in B cell chronic lymphocytic leukaemia (B-CLL): comparison of three approaches to analyze its expression. Cytometry B Clin Cytom. 2006;70:136-141.
28. Byrd JC, Gribben JG, Peterson BL, et al. Select high-risk genetic features predict earlier progression following chemoimmunotherapy with fludarabine and rituximab in chronic lymphocytic leukemia: justification for risk-adapted therapy. J Clin Oncol. 2006;24:437-443.
29. Hallek M, Langenmayer I, Nerl C, et al. Elevated serum thymidine kinase levels identify a subgroup at high risk of disease-progression in early, non-smoldering chronic lymphocytic leukemia. Blood. 1999;93:1732-1737.
30. Keating MJ, Lerner S, Kantarjian H, Freireich EJ, O'Brien S. The serum ß2-microglobulin (ß2m) level is more powerful than stage in predicting response and survival in chronic lymphocytic leukemia (CLL). Blood. 1995;86 (Suppl. I):606a.
31. Reinisch W, Willheim M, Hilgarth M, et al. Soluble CD23 reliably reflects disease activity in B-cell chronic lymphocytic leukemia. J Clin Oncol. 1994;12:2146-2149.
32. Sarfati M, Chevret S, Chastang C, et al. Prognostic importance of serum soluble CD23 level in chronic lymphocytic leukemia. Blood. 1996;88:4259-4264.
33. Wierda WG, O'Brien S, Wang X, et al. Prognostic nomogram and index for overall survival in previously untreated patients with chronic lymphocytic leukemia. Blood. 2007;109:4679-4685.
34. Magnac C, Porcher R, Davi F, et al. Predictive value of serum thymidine kinase level for Ig-V mutational status in B-CLL. Leukemia. 2003;17:133-137.

35. Matthews C, Catherwood MA, Morris TC, et al. Serum TK levels in CLL identify Binet stage A patients within biologically defined prognostic subgroups most likely to undergo disease progression. Eur J Haematol. 2006;77:309-317.
36. Rai KR, Sawitsky A, Cronkite EP, Chanana AD, Levy RN, Pasternack BS. Clinical staging of chronic lymphocytic leukemia. Blood. 1975;46:219-234.
37. Binet JL, Auquier A, Dighiero G, et al. A new prognostic classification of chronic lymphocytic leukemia derived from a multivariate survival analysis. Cancer. 1981;48:198-204.
38. Rai KR. A critical analysis of staging in CLL. In: Gale RP, Rai KR, eds. Chronic Lymphocytic Leukemia: Recent Progress and Future Directions. New York: Alan R.Liss.; 1987:253-264.
39. Extermann M, Overcash J, Lyman GH, Parr J, Balducci L. Comorbidity and functional status are independent in older patients. J Clin Oncol. 1998;16:1582-1587.
40. Balducci L, Extermann M. Management of cancer in the older person: a practical approach. Oncologist. 2000;5:224-237.
41. O'Brien SM, Keating MJ, Mocarski ES. Updated guidelines on the management of cytomegalovirus reactivation in patients with chronic lymphocytic leukemia treated with alemtuzumab. Clin Lymphoma Myeloma. 2006;7:125-130.
42. Yagci M, Acar K, Sucak GT, Aki Z, Bozdayi G, Haznedar R. A prospective study on chemotherapy-induced hepatitis B virus reactivation in chronic HBs Ag carriers with hematologic malignancies and pre-emptive therapy with nucleoside analogues. Leuk Lymphoma. 2006;47:1608-1612.
43. Rossi G, Pelizzari A, Motta M, Puoti M. Primary prophylaxis with lamivudine of hepatitis B virus reactivation in chronic HbsAg carriers with lymphoid malignancies treated with chemotherapy. Br J Haematol. 2001;115:58-62.
44. Muntanola A, Bosch F, Arguis P, et al. Abdominal computed tomography predicts progression in patients with Rai stage 0 chronic lymphocytic leukemia. J Clin Oncol. 2007;25:1576-1580.
45. Dighiero G, Maloum K, Desablens B, et al. Chlorambucil in indolent chronic lymphocytic leukemia. N Engl J Med. 1998;338:1506-1514.
46. Shustik C, Mick R, Silver R, Sawitsky A, Rai K, Shapiro L. Treatment of early chronic lymphocytic leukemia: intermittent chlorambucil versus observation. Hematol Oncol. 1988;6:7-12.
47. Montserrat E, Fontanillas M, Estape J, for the Spanish PETHEMA Group. Chronic lymphocytic leukemia treatment: an interim report of PETHEMA trials. Leuk Lymphoma. 1991;5:89-92.
48. CLL trialists´ collaborative group. Chemotherapeutic options in chronic lymhocytic leukemia. J Natl Cancer Inst. 1999;91:861-868.
49. Chemotherapeutic options in chronic lymphocytic leukemia: a meta-analysis of the randomized trials. CLL Trialists' Collaborative Group. J Natl Cancer Inst. 1999;91:861-868.
50. Moreno C, Villamor N, Colomer D, et al. Allogeneic stem-cell transplantation may overcome the adverse prognosis of unmutated VH gene in patients with chronic lymphocytic leukemia. J Clin Oncol. 2005;23:3433-3438.
51. Dreger P, Brand R, Milligan D, et al. Reduced-intensity conditioning lowers treatment-related mortality of allogeneic stem cell transplantation for chronic lymphocytic leukemia: a population-matched analysis. Leukemia. 2005;19:1029-1033.
52. Gribben JG, Zahrieh D, Stephans K, et al. Autologous and allogeneic stem cell transplantations for poor-risk chronic lymphocytic leukemia. Blood. 2005;106:4389-4396.
53. Schetelig J, Thiede C, Bornhauser M, et al. Evidence of a graft-versus-leukemia effect in

chronic lymphocytic leukemia after reduced-intensity conditioning and allogeneic stem-cell transplantation: the Cooperative German Transplant Study Group. J Clin Oncol. 2003;21:2747-2753.
54. Caballero D, Garcia-Marco JA, Martino R, et al. Allogeneic transplant with reduced intensity conditioning regimens may overcome the poor prognosis of B-cell chronic lymphocytic leukemia with unmutated immunoglobulin variable heavy-chain gene and chromosomal abnormalities (11q- and 17p-). Clin Cancer Res. 2005;11:7757-7763.
55. Oudat R, Keating MJ, Lerner S, O'Brien S, Albitar M. Significance of the levels of bone marrow lymphoid infiltrate in chronic lymphocytic leukemia patients with nodular partial remission. Leukemia. 2002;16:632-635.
56. Noy A, Verma R, Glenn M, et al. Clonotypic polymerase chain reaction confirms minimal residual disease in CLL nodular PR: results from a sequential treatment CLL protocol. Blood. 2001;97:1929-1936.
57. Cheson BD, Pfistner B, Juweid ME, et al. Revised response criteria for malignant lymphoma. J Clin Oncol. 2007;25:579-586.
58. Dreger P, Corradini P, Kimby E, et al. Indications for allogeneic stem cell transplantation in chronic lymphocytic leukemia: the EBMT transplant consensus. Leukemia. 2007;21:12-17.
59. Rawstron AC, Villamor N, Ritgen M, et al. International standardized approach for flow cytometric residual disease monitoring in chronic lymphocytic leukaemia. Leukemia. 2007;21:956-964.
60. Moreton P, Kennedy B, Lucas G, et al. Eradication of Minimal Residual Disease in B-Cell Chronic Lymphocytic Leukemia After Alemtuzumab Therapy Is Associated With Prolonged Survival. J Clin Oncol. 2005;23:2971-2979.
61. Wendtner CM, Ritgen M, Schweighofer CD, et al. Consolidation with alemtuzumab in patients with chronic lymphocytic leukemia (CLL) in first remission--experience on safety and efficacy within a randomized multicenter phase III trial of the German CLL Study Group (GCLLSG). Leukemia. 2004;18:1093-1101.
62. Bosch F, Ferrer A, Lopez-Guillermo A, et al. Fludarabine, cyclophosphamide and mitoxantrone in the treatment of resistant or relapsed chronic lymphocytic leukaemia. Br J Haematol. 2002;119:976-984.
63. Eichhorst BF, Busch R, Schweighofer C, Wendtner CM, Emmerich B, Hallek M. Due to low infection rates no routine anti-infective prophylaxis is required in younger patients with chronic lymphocytic leukaemia during fludarabine-based first line therapy. Br J Haematol. 2007;136:63-72.
64. Cheson BD, Frame JN, Vena D, Quashu N, Sorensen JM. Tumor lysis syndrome: an uncommon complication of fludarabine therapy of chronic lymphocytic leukemia. J Clin Oncol. 1998;16:2313-2320.
65. Eichhorst BF, Busch R, Obwandner T, Kuhn-Hallek I, Herschbach P, Hallek M. Health-related quality of life in younger patients with chronic lymphocytic leukemia treated with fludarabine plus cyclophosphamide or fludarabine alone for first-line therapy: a study by the German CLL Study Group. J Clin Oncol. 2007;25:1722-1731.
66. Molica S. Quality of life in chronic lymphocytic leukemia: a neglected issue. Leuk Lymphoma. 2005;46:1709-1714.
67. Holzner B, Kemmler G, Kopp M, Nguyen-Van-Tam D, Sperner-Unterweger B, Greil R. Quality of life of patients with chronic lymphocytic leukemia: results of a longitudinal investigation over 1 yr. Eur J Haematol. 2004;72:381-389.
68. Levy V, Porcher R, Delabarre F, Leporrier M, Cazin B, Chevret S. Evaluating treatment strategies in chronic lymphocytic leukemia: use of quality-adjusted survival analysis. J Clin

Epidemiol. 2001;54:747-754.
69. Ozer H, Armitage JO, Bennett CL, et al. 2000 update of recommendations for the use of hematopoietic colony-stimulating factors: evidence-based, clinical practice guidelines. American Society of Clinical Oncology Growth Factors Expert Panel. J Clin Oncol. 2000;18:3558-3585.
70. Ludwig H, Rai K, Blade J, et al. Management of disease-related anemia in patients with multiple myeloma or chronic lymphocytic leukemia: epoetin treatment recommendations. Hematol J. 2002;3:121-130.
71. Lichtin A. The ASH/ASCO clinical guidelines on the use of erythropoietin. Best Pract Res Clin Haematol. 2005;18:433-438.
72. Rodon P, Breton P, Courouble G. Treatment of pure red cell aplasia and autoimmune haemolytic anaemia in chronic lymphocytic leukaemia with Campath-1H. Eur J Haematol. 2003;70:319-321.
73. Hamblin TJ. Autoimmune complications of chronic lymphocytic leukemia. Semin Oncol. 2006;33:230-239.
74. Zaja F, Vianelli N, Sperotto A, et al. Anti-CD20 therapy for chronic lymphocytic leukemia-associated autoimmune diseases. Leuk Lymphoma. 2003;44:1951-1955.
75. Gupta N, Kavuru S, Patel D, et al. Rituximab-based chemotherapy for steroid-refractory autoimmune hemolytic anemia of chronic lymphocytic leukemia. Leukemia. 2002;16:2092-2095.

blood

Prepublished online January 23, 2008;
doi:10.1182/blood-2007-06-093906

Guidelines for the diagnosis and treatment of chronic lymphocytic leukemia: a report from the International Workshop on Chronic Lymphocytic Leukemia (IWCLL) updating the National Cancer Institute-Working Group (NCI-WG) 1996 guidelines

Michael Hallek, Bruce D. Cheson, Daniel Catovsky, Federico Caligaris-Cappio, Guillaume Dighiero, Hartmut Dohner, Peter Hillmen, Michael J. Keating, Emili Montserrat, Kanti R. Rai and Thomas J. Kipps

Information about reproducing this article in parts or in its entirety may be found online at:
http://www.bloodjournal.org/site/misc/rights.xhtml#repub_requests

Information about ordering reprints may be found online at:
http://www.bloodjournal.org/site/misc/rights.xhtml#reprints

Information about subscriptions and ASH membership may be found online at:
http://www.bloodjournal.org/site/subscriptions/index.xhtml

Advance online articles have been peer reviewed and accepted for publication but have not yet appeared in the paper journal (edited, typeset versions may be posted when available prior to final publication). Advance online articles are citable and establish publication priority; they are indexed by PubMed from initial publication. Citations to Advance online articles must include digital object identifier (DOIs) and date of initial publication.

Blood (print ISSN 0006-4971, online ISSN 1528-0020), is published weekly by the American Society of Hematology, 2021 L St, NW, Suite 900, Washington DC 20036.
Copyright 2011 by The American Society of Hematology; all rights reserved.

Appendix 13

Schedule of Response and Minimal Residual Disease Assessments

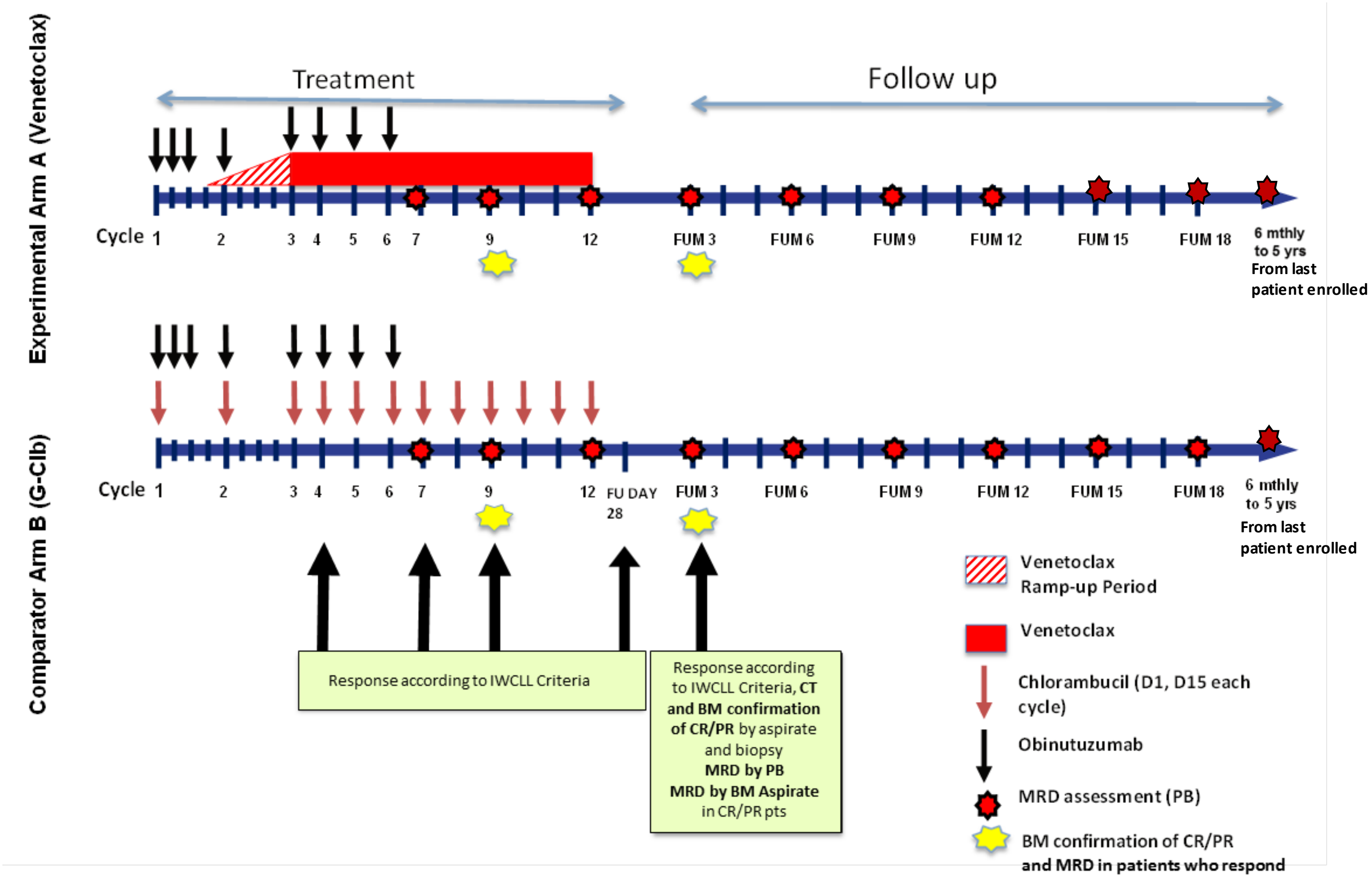

BM = bone marrow aspirate and biopsy; CR = complete response; CT = computed tomography; D = day; FUM = follow-up months; GClb = obinutuzumab + chlorambucil; IWCLL = International Workshop on Chronic Lymphocytic Leukemia; MRD = minimal residual disease; PB = peripheral blood; PE = physical exam; PR = partial response.

Appendix 14
Duration and Reporting of Adverse Events

	RELATED		UNRELATED	
	**Post-Treatment Reporting Period**	**Follow-up**	**Post-treatment Reporting Period**	**Follow-up**
AEs Grades 1 and 2	28 days	Not required	28 days	Not required
AEs Grades 3 and 4	6 months or NLT	Until resolution to ≤ Grade 2	6 months or NLT	Until resolution to ≤ Grade 2
Major infections (Grades 3 and 4)	2 years or NLT	Until resolution stabilization or end of study	2 years or NLT	Until resolution stabilization or 1 year after onset
SAEs	Indefinitely	Until resolution stabilization or end of study	Throughout follow-up period	Until resolution stabilization or 1 year after onset
Secondary malignancies	Indefinitely	Not required	Indefinitely	Not required

AE = adverse event; SAE = serious adverse event; NLT = next leukemia treatment.

Appendix 15
EQ-5D-3L Questionnaire

By placing a checkmark in one box in each group below, please indicate which statements best describe your own health state today.

Mobility

I have no problems in walking about ❑

I have some problems in walking about ❑

I am confined to bed ❑

Self-Care

I have no problems with self-care ❑

I have some problems washing or dressing myself ❑

I am unable to wash or dress myself ❑

Usual Activities (*e.g. work, study, housework, family or leisure activities)*

I have no problems with performing my usual activities ❑

I have some problems with performing my usual activities ❑

I am unable to perform my usual activities ❑

Pain/Discomfort

I have no pain or discomfort ❑

I have moderate pain or discomfort ❑

I have extreme pain or discomfort ❑

Anxiety/Depression

I am not anxious or depressed ❑

I am moderately anxious or depressed ❑

I am extremely anxious or depressed ❑

Appendix 15
EQ-5D-3L Questionnaire (cont.)

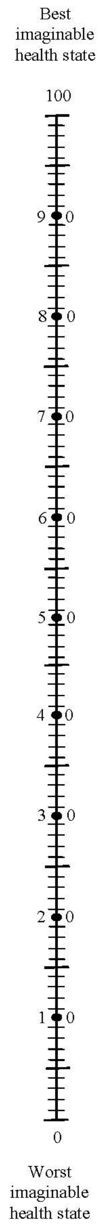

To help people say how good or bad a health state is, we have drawn a scale (rather like a thermometer) on which the best state you can imagine is marked 100 and the worst state you can imagine is marked 0.

We would like you to indicate on this scale how good or bad your own health is today, in your opinion. Please do this by drawing a line from the box below to whichever point on the scale indicates how good or bad your health state is today.

Your own health state today

Appendix 16
Eastern Cooperative Oncology Group Performance Status

Grade	Description
0	Fully active, able to carry on all pre-disease performance without restriction
1	Restricted in physically strenuous activity but ambulatory and able to carry out work of a light or sedentary nature (e.g., light housework or office work)
2	Ambulatory and capable of all self-care but unable to carry out any work activities. Up and about > 50% of waking hours
3	Capable of only limited self-care, confined to a bed or chair > 50% of waking hours
4	Completely disabled. Cannot carry on any self-care. Totally confined to bed or chair
5	Dead

Appendix 17
Matutes Scoring System

Flow cytometric analysis of peripheral blood or bone marrow is performed for expression of the cell surface markers listed in the table below. The scores for each marker are summed.

	0 Points	1 Point
CD5	Negative	Positive
CD23	Negative	Positive
FMC7	Positive	Negative
S Ig	Strong	Weak
CD22	Strong	Weak

Note: A score of ≥ 4 is indicative of CLL; a score ≤ 3 should prompt consideration of an alternative diagnosis.

Source: http://bloodref.com/lymphoid/cll/cll-score

Matutes E, Owusu-Ankomah K, Morilla R, et al. The immunological profile of B-cell disorders and proposal of a scoring system for the diagnosis of CLL. Leukemia 1994;8:1640–5.

Appendix 18
M.D. Anderson Symptom Inventory (MDASI-CLL)

Part I. How severe are your symptoms?

People with cancer frequently have symptoms that are caused by their disease or by their treatment. We ask you to rate how severe the following symptoms have been ***in the last 24 hours***. Please select a number from 0 (symptom has not been present) to 10 (the symptom was as bad as you can imagine it could be) for each item.

	Not Present 0	1	2	3	4	5	6	7	8	9	As Bad As You Can Imagine 10
1. Your pain at its WORST?	○	○	○	○	○	○	○	○	○	○	○
2. Your fatigue (tiredness) at its WORST?	○	○	○	○	○	○	○	○	○	○	○
3. Your nausea at its WORST?	○	○	○	○	○	○	○	○	○	○	○
4. Your disturbed sleep at its WORST?	○	○	○	○	○	○	○	○	○	○	○
5. Your feeling of being distressed (upset) at its WORST?	○	○	○	○	○	○	○	○	○	○	○
6. Your shortness of breath at its WORST?	○	○	○	○	○	○	○	○	○	○	○
7. Your problem with remembering things at its WORST?	○	○	○	○	○	○	○	○	○	○	○
8. Your problem with lack of appetite at its WORST?	○	○	○	○	○	○	○	○	○	○	○
9. Your feeling drowsy (sleepy) at its WORST?	○	○	○	○	○	○	○	○	○	○	○
10. Your having a dry mouth at its WORST?	○	○	○	○	○	○	○	○	○	○	○
11. Your feeling sad at its WORST?	○	○	○	○	○	○	○	○	○	○	○
12. Your vomiting at its WORST?	○	○	○	○	○	○	○	○	○	○	○
13. Your numbness or tingling at its WORST?	○	○	○	○	○	○	○	○	○	○	○

SAMPLE

Copyright 2000 The University of Texas M. D. Anderson Cancer Center
All rights reserved.

Appendix 18
M.D. Anderson Symptom Inventory (MDASI-CLL) (cont.)

	Not Present 0	1	2	3	4	5	6	7	8	9	As Bad As You Can Imagine 10
14. Your night sweats at their WORST?	○	○	○	○	○	○	○	○	○	○	○
15. Fevers and chills at their WORST?	○	○	○	○	○	○	○	○	○	○	○
16. Lymph node swelling at its WORST?	○	○	○	○	○	○	○	○	○	○	○
17. Your diarrhea at its WORST?	○	○	○	○	○	○	○	○	○	○	○
18. Your bruising easily or bleeding at its WORST?	○	○	○	○	○	○	○	○	○	○	○
19. Your constipation at its WORST?	○	○	○	○	○	○	○	○	○	○	○

Part II. How have your symptoms **interfered** with your life?

Symptoms frequently interfere with how we feel and function. Ho[illegible]uch [illegible]ve[illegible]r symptoms interfered with the following items *in the last 24 hours*? Please select a number from 0 (s[illegible] no[illegible]terfered) to 10 (symptoms interfered completely) for each item.

	Did Not Interfere 0	1	2	3	4	5	6	7	8	9	Interfered Completely 10
20. General activity?	○	○	○	○	○	○	○	○	○	○	○
21. Mood?	○	○	○	○	○	○	○	○	○	○	○
22. Work (including work around the house)?	○	○	○	○	○	○	○	○	○	○	○
23. Relations with other people?	○	○	○	○	○	○	○	○	○	○	○
24. Walking?	○	○	○	○	○	○	○	○	○	○	○
25. Enjoyment of life?	○	○	○	○	○	○	○	○	○	○	○

SAMPLE

Copyright 2000 The University of Texas M. D. Anderson Cancer Center
All rights reserved.

Appendix 19
European Organisation for Research and Treatment of Cancer Quality of Life Questionnaire Core 30 (EORTC QLQ-C30)

EORTC QLQ-C30 (version 3)

We are interested in some things about you and your health. Please answer all of the questions yourself by circling the number that best applies to you. There are no "right" or "wrong" answers. The information that you provide will remain strictly confidential.

Please fill in your initials: |__|__|__|__|

Your birthdate (Day, Month, Year): |__|__|__|__|__|__|__|__|

Today's date (Day, Month, Year): 31 |__|__|__|__|__|__|__|__|

		Not at All	A Little	Quite a Bit	Very Much
1.	Do you have any trouble doing strenuous activities, like carrying a heavy shopping bag or a suitcase?	1	2	3	4
2.	Do you have any trouble taking a long walk?	1	2	3	4
3.	Do you have any trouble taking a short walk outside of the house?	1	2	3	4
4.	Do you need to stay in bed or a chair during the day?	1	2	3	4
5.	Do you need help with eating, dressing, washing yourself or using the toilet?	1	2	3	4

**During the past week:**		Not at All	A Little	Quite a Bit	Very Much
6.	Were you limited in doing either your work or other daily activities?	1	2	3	4
7.	Were you limited in pursuing your hobbies or other leisure time activities?	1	2	3	4
8.	Were you short of breath?	1	2	3	4
9.	Have you had pain?	1	2	3	4
10.	Did you need to rest?	1	2	3	4
11.	Have you had trouble sleeping?	1	2	3	4
12.	Have you felt weak?	1	2	3	4
13.	Have you lacked appetite?	1	2	3	4
14.	Have you felt nauseated?	1	2	3	4
15.	Have you vomited?	1	2	3	4

Please go on to the next page

Specimen

Appendix 19
European Organisation for Research and Treatment of Cancer Quality of Life Questionnaire Core 30 (EORTC QLQ-C30) (cont.)

**During the past week:**	Not at All	A Little	Quite a Bit	Very Much
16. Have you been constipated?	1	2	3	4
17. Have you had diarrhea?	1	2	3	4
18. Were you tired?	1	2	3	4
19. Did pain interfere with your daily activities?	1	2	3	4
20. Have you had difficulty in concentrating on things, like reading a newspaper or watching television?	1	2	3	4
21. Did you feel tense?	1	2	3	4
22. Did you worry?	1	2	3	4
23. Did you feel irritable?	1	2	3	4
24. Did you feel depressed?	1	2	3	4
25. Have you had difficulty remembering things?	1	2	3	4
26. Has your physical condition or medical treatment interfered with your <u>family</u> life?	1	2	3	4
27. Has your physical condition or medical treatment interfered with your <u>social</u> activities?	1	2	3	4
28. Has your physical condition or medical treatment caused you financial difficulties?	1	2	3	4

For the following questions please circle the number between 1 and 7 that best applies to you

29. How would you rate your overall health during the past week?

1	2	3	4	5	6	7
Very poor						Excellent

30. How would you rate your overall quality of life during the past week?

1	2	3	4	5	6	7
Very poor						Excellent

© Copyright 1995 EORTC Study Group on Quality of Life. All rights reserved. Version 3.0

Specimen

Appendix 20
Guidelines for Defining Tumor Lysis Syndrome

All tumor lysis syndrome events should be graded per the National Cancer Institute Common Terminology Criteria for Adverse Events, Version 4.03 criteria.

Howard et al. (2011) defined laboratory tumor lysis syndrome as the presence of two or more electrolyte changes above or below the thresholds described above occurring during the same 24-hour period within 3 days before the start of therapy or 7 days after the start of therapy. For the purposes of this study, this window applies to the initiation of any study therapy and each dose escalation of venetoclax. Furthermore, this assessment assumes that a patient has or will receive adequate hydration (± alkalinization) and a hypouricemic agent(s).

Table 1 Howard Definition of Laboratory Tumor Lysis Syndrome

Laboratory Assessment	Range
Uric acid	$>$476 μmol/L ($>$8.0 mg/dL)
Potassium	$>$6.0 mmol/L ($>$6.0 mEq/L)
Phosphorous	$>$1.5 mmol/L ($>$4.5 mg/dL)
Corrected calcium	$<$1.75 mmol/l ($<$7.0 mg/dL) or ionized calcium $<$1.12 (0.3 mmol/L) [a]

Note: Howard et al. (2011) defined laboratory tumor lysis syndrome as the presence of two or more electrolyte changes above or below the thresholds described above occurring during the same 24-hour period within 3 days before the start of therapy or 7 days afterward. For the purposes of this study, this window applies to the initiation of any study therapy and each dose escalation of venetoclax. Furthermore, this assessment assumes that a patient has or will receive adequate hydration (± alkalinization) and a hypouricemic agent(s).

[a] The corrected calcium level in mg/dL is the measured calcium in mg/dL + (0.8 × [4-albumin in g/dL]).

Table 2 Howard Definition of Clinical Tumor Lysis Syndrome

The presence of laboratory TLS and one or more of the following criteria:
Creatinine[a]: An increase in serum creatinine level of 0.3 mg/dL (26.5 µmol/L); a single value > 1.5 times the ULN of the age appropriate normal range if no baseline creatinine measurement is available; or the presence of oliguria, defined as average urine output of < 0.5 mL/kg/hour for 6 hours
Cardiac dysrhythmia or sudden death probably or definitely caused by hyperkalemia
Cardiac dysrhythmia, sudden death, seizure, neuromuscular irritability (tetany, paresthesias, muscle twitching, carpopedal spasm, Trousseau's sign, Chvostek's sign, layngospasm or bronchospasm), hypotension, or heart failure probably or definitely caused by hypocalcemia[b]

TLS = tumor lysis syndrome; ULN = upper limit of normal.

[a] Acute kidney injury is defined as an increase in the creatinine level of ≥ 0.3 mg/dL (26.5 umol/L) or a period of oliguria lasting ≥ 6 hours. By definition, if acute kidney injury is present, the patient has clinical TLS.

[b] Not directly attributable to a therapeutic agent.

REFERENCE

Howard SC, Jones DP, Pui CH. The tumor lysis syndrome. New Engl J Med 2011;364:1844–54.

Appendix 21
The Lawton Instrumental Activities of Daily Living Scale

The Lawton Instrumental Activities of Daily Living Scale

A. Ability to Use Telephone

1. Operates telephone on own initiative; looks up and dials numbers 1
2. Dials a few well-known numbers 1
3. Answers telephone, but does not dial 1
4. Does not use telephone at all 0

B. Shopping

1. Takes care of all shopping needs independently 1
2. Shops independently for small purchases 0
3. Needs to be accompanied on any shopping trip 0
4. Completely unable to shop 0

C. Food Preparation

1. Plans, prepares, and serves adequate meals independently 1
2. Prepares adequate meals if supplied with ingredients 0
3. Heats and serves prepared meals or prepares meals but does not maintain adequate diet 0
4. Needs to have meals prepared and served 0

D. Housekeeping

1. Maintains house alone with occasion assistance (heavy work) 1
2. Performs light daily tasks such as dishwashing, bed making 1
3. Performs light daily tasks, but cannot maintain acceptable level of cleanliness 1
4. Needs help with all home maintenance tasks 1
5. Does not participate in any housekeeping tasks 0

E. Laundry

1. Does personal laundry completely 1
2. Launders small items, rinses socks, stockings, etc 1
3. All laundry must be done by others 0

F. Mode of Transportation

1. Travels independently on public transportation or drives own car 1
2. Arranges own travel via taxi, but does not otherwise use public transportation 1
3. Travels on public transportation when assisted or accompanied by another 1
4. Travel limited to taxi or automobile with assistance of another 0
5. Does not travel at all 0

G. Responsibility for Own Medications

1. Is responsible for taking medication in correct dosages at correct time 1
2. Takes responsibility if medication is prepared in advance in separate dosages 0
3. Is not capable of dispensing own medication 0

H. Ability to Handle Finances

1. Manages financial matters independently (budgets, writes checks, pays rent and bills, goes to bank); collects and keeps track of income 1
2. Manages day-to-day purchases, but needs help with banking, major purchases, etc 1
3. Incapable of handling money 0

Scoring: For each category, circle the item description that most closely resembles the client's highest functional level (either 0 or 1).

Source: Lawton MP, Brody EM. Assessment of older people: self-maintaining and instrumental activities of daily living. Gerontologist 1969;9(3):179–86.
