# Supplementary material for: Transcriptomic profiles and 5-year results from the randomized CLL14 study of venetoclax plus obinutuzumab versus chlorambucil plus obinutuzumab in chronic lymphocytic leukemia
Source: Nat Commun. 2023 Apr 18;14:2147. doi: 10.1038/s41467-023-37648-w (PMC10113251; doi:10.1038/s41467-023-37648-w)
Supplement: Supplementary file 2 — Reporting Summary [file 41467_2023_37648_MOESM2_ESM.pdf]

nature portfolio

Corresponding author(s): Othman Al-Sawaf
Michael Hallek
Kirsten Fischer

Last updated by author(s): Mar 5, 2023

Reporting Summary

Nature Portfolio wishes to improve the reproducibility of the work that we publish. This form provides structure for consistency and transparency in reporting. For further information on Nature Portfolio policies, see our Editorial Policies and the Editorial Policy Checklist.

Statistics

For all statistical analyses, confirm that the following items are present in the figure legend, table legend, main text, or Methods section.

n/a	Confirmed	
☐	☒	The exact sample size (*n*) for each experimental group/condition, given as a discrete number and unit of measurement
☐	☒	A statement on whether measurements were taken from distinct samples or whether the same sample was measured repeatedly
☐	☒	The statistical test(s) used AND whether they are one- or two-sided<br>*Only common tests should be described solely by name; describe more complex techniques in the Methods section.*
☐	☒	A description of all covariates tested
☐	☒	A description of any assumptions or corrections, such as tests of normality and adjustment for multiple comparisons
☐	☒	A full description of the statistical parameters including central tendency (e.g. means) or other basic estimates (e.g. regression coefficient) AND variation (e.g. standard deviation) or associated estimates of uncertainty (e.g. confidence intervals)
☐	☒	For null hypothesis testing, the test statistic (e.g. *F*, *t*, *r*) with confidence intervals, effect sizes, degrees of freedom and *P* value noted<br>*Give P values as exact values whenever suitable.*
☒	☐	For Bayesian analysis, information on the choice of priors and Markov chain Monte Carlo settings
☒	☐	For hierarchical and complex designs, identification of the appropriate level for tests and full reporting of outcomes
☒	☐	Estimates of effect sizes (e.g. Cohen's *d*, Pearson's *r*), indicating how they were calculated

Our web collection on statistics for biologists contains articles on many of the points above.

Software and code

Policy information about availability of computer code

Data collection	Data entered manually was collected via a Sponsor-designated electronic data capture (EDC) system through use of the electronic Case Report Forms (eCRFs).
Data analysis	Data analyses were perfomred using SPSS version 28 (SPSS, Chicago, IL, USA), SAS version 9.4 (SAS Institue, Cary, NS, USA) and R version 3.6.1 (R Foundation, Vienna, Austria).<br><br># RNAseq data processing and annotation<br>DESeq2_1.32.0<br>limma_3.48.3<br>igis_0.7.09<br>biomaRt2.48.3<br>CIBERSORTx_2019<br><br># Gene Set Enrichment<br>GSVA_1.40.1<br>fgsea_1.18.0<br><br>#Cluster<br>Seurat_4.0.4

Visualization
EnhancedVolcano_1.13.2 c
fgsea_1.18.0
ggplot2_3.3.5

For manuscripts utilizing custom algorithms or software that are central to the research but not yet described in published literature, software must be made available to editors and reviewers. We strongly encourage code deposition in a community repository (e.g. GitHub). See the Nature Portfolio guidelines for submitting code & software for further information.

Data

Policy information about availability of data

All manuscripts must include a data availability statement. This statement should provide the following information, where applicable:

- Accession codes, unique identifiers, or web links for publicly available datasets
- A description of any restrictions on data availability
- For clinical datasets or third party data, please ensure that the statement adheres to our policy

Individual patient-level data, including de-identified clinical metadata, raw RNAseq data, and processed RNAseq data are available to qualified researchers at the European Genome-Phenome Archive under accession number EGAS00001006596. To request access to such data, researchers can contact the corresponding authors, who will facilitate the review by the GCLLSG/Roche/AbbVie data access committees. The data will be released to such requesters with necessary agreements to enforce terms such as security, patient privacy, and consent of specified data use, consistent with evolving, applicable data protection laws.
Hallmark genesets were used from the Molecular Signatures Database (MSigDB) (https://www.gsea-msigdb.org/gsea/msigdb/).
GRCh38.p10 was used for read alignment (https://www.ncbi.nlm.nih.gov/assembly/GCF_000001405.36/).

Human research participants

Policy information about studies involving human research participants and Sex and Gender in Research.

Reporting on sex and gender	Sex and gender were not considered in the study design. Self-reported sex was collected and used in the analyses.
Population characteristics	Patients were considered eligible for the study if they were 18 years or older, had previously untreated active CLL requiring treatment as per iwCLL criteria and were considered unfit due to coexisting conditions, as indicated by a cumulative illness rating scale (CIRS) greater than 6 and/or an impaired renal function (creatinine clearance < 70 ml/min). Further inclusion criteria were a platelet count of at least 30 G/l (10 G/l if due to bone marrow involvement), total haemoglobin of at least 9 g/dl without transfusion support (unless CLL related) and a life expectancy greater than 6 months. The full list of eligibility criteria is outlined in the study protocol (supplementary material).<br>In the Clb-Obi arm, 33.8% are females, 66.2% are males; 20.4% have Binet A, 37% Binet B and 42.6% Binet C disease. The median cumulative illness rating scale (CIRS) score was 8. The median age was 71. In the Ven-Obi arm, 32.4% are females, 67.6% are males; 21.3% have Binet A, 35.2% have Binet B and 43.5% have Binet C disease. The median CIRS score was 9, the median age was 72.
Recruitment	Participants were approached during routine clinical visits and screened for eligibility/consented if inclusion criteria were met and no exclusion criterial were identified. We do not anticipate a bias in recruitment of participants in this study.
Ethics oversight	The study was registered at US and EU clinical trial registries (NCT02242942, EudraCT 2014-001810-24) and approved by the central ethics committee of the University of Cologne as well as by the ethical review boards responsible for each study site. The study was performed according to the principles of the Declaration of Helsinki. All patients provided written informed consent to participate.

Note that full information on the approval of the study protocol must also be provided in the manuscript.

Field-specific reporting

Please select the one below that is the best fit for your research. If you are not sure, read the appropriate sections before making your selection.

☒ Life sciences ☐ Behavioural & social sciences ☐ Ecological, evolutionary & environmental sciences

For a reference copy of the document with all sections, see nature.com/documents/nr-reporting-summary-flat.pdf

Life sciences study design

All studies must disclose on these points even when the disclosure is negative.

Sample size	The sample size for the study was determined given the requirements to perform a hypothesis test for clinically relevant statistical superiority in the primary endpoint of the investigator-assessed progression-free survival (PFS). The assumptions used for the original sample size calculation were given as follows:

	1. Log-rank test at the two-sided 0.05 level of significance<br>2. Median PFS for chlorambucil—obinutuzumab control arm (27 months)<br>3. 80% power to detect HR = 0.65 for the comparison of venetoclax—obinutuzumab experimental arm versus chlorambucil—obinutuzumab, with median PFS for venetoclax—obinutuzumab increased to 41.5 months<br>4. Exponential distribution of PFS<br>5. Annual dropout rate of 10%<br>6. One interim analysis for efficacy after 75% of PFS events, utilizing a stopping boundary according to the family error spending function with parameter = 9.21<br>Based on these assumptions, a total of 170 PFS events were calculated to be required for the final analysis of PFS. Subsequently the timing of the interim analysis has changed at a minimum f 110 (65%) events. However, this change did not require changes to the sample size, as the impact on the statistical power calculation was negligible.
Data exclusions	All randomly assigned patients were included in the efficacy analyses (intention-to-treat population). All randomly assigned patients who received at least one dose of study medication (ie, obinutuzumab, venetoclax, or chlorambucil) were included in the safety analyses (safety population). No data were excluded from the analyses.
Replication	To check robustness of the primary analysis of PFS and underlying assumptions, the following sensitivity analyses for PFS (both investigatorassessed<br>and IRC assessed) were performed on the intention-to-treat population, which supported the robustness of the results:<br>• An unstratified log-rank test for the primary PFS comparison between treatment arms.<br>• The impact of patients' initiation of non-protocol-specified anti-CLL therapy without meeting the criteria of disease progression/relapse on PFS was assessed by censoring these patients at the start date of the non-protocol-specified anti CLL treatment. Stopping only one component of the randomized study treatment was not considered a reason for censoring patients.<br>• To assess the impact of missing assessments on PFS, an analysis on PFS was performed by censoring those patients who progressed, relapsed or died after missing more than one visit consecutively at their last adequate response assessment date before the missed visits.
Randomization	Patients were randomly assigned 1:1 to either the Ven-Obi arm or the Clb-Obi arm using a Web and voice mail system based on a computergenerated<br>randomization schedule. A block size of six was used to balance the randomization. Patient stratification was based on Binet stage (A, B or C) and geographic region (US/Canada/Central America; Australia/New Zealand; Western Europe; Central and Eastern Europe; or Latin America). Allocation was not blinded to site investigators and patients.
Blinding	This study as open-label and no placebo was used, therefore blinding was not needed.

Reporting for specific materials, systems and methods

We require information from authors about some types of materials, experimental systems and methods used in many studies. Here, indicate whether each material, system or method listed is relevant to your study. If you are not sure if a list item applies to your research, read the appropriate section before selecting a response.

Materials & experimental systems

n/a	Involved in the study	
☒	☐	Antibodies
☒	☐	Eukaryotic cell lines
☒	☐	Palaeontology and archaeology
☒	☐	Animals and other organisms
☐	☒	Clinical data
☒	☐	Dual use research of concern

Methods

n/a	Involved in the study	
☒	☐	ChIP-seq
☒	☐	Flow cytometry
☒	☐	MRI-based neuroimaging

Clinical data

Policy information about clinical studies

All manuscripts should comply with the ICMJEguidelines for publication of clinical research and a completedCONSORT checklist must be included with all submissions.

Clinical trial registration	NCT02242942, EudraCT 2014-001810-24
Study protocol	Study protocol in the accompanying supplementary material.
Data collection	Enrollment between 7 August 2015 and 4 August 2016. Patient follow-up and sample collection ongoing.<br>Data cut-off for this report was November 8th 2021. Data were collected at 196 sites (academic centres, community hospitals and practices) in 21 countries, a full list is provided in the supplementary material.
Outcomes	The primary endpoint was progression-free survival (PFS) as assessed by the investigator, pre-defined as the time from randomization to the first occurrence of disease progression (according to iwCLL criteria, i.e. measured by peripheral blood, bone marrow, physical examination and/or imaging) or death from any cause.<br>Secondary endpoints included overall survvial (defined as the time from randomization to death from any cause), time to next treatment (defined as the time from randomization to initiation of new anti-leukemic therapy or death from any cause), overall response (according to iwCLL criteria), complete response (according to iwCLL criteria), and MRD response rate as measured by allele-specific oligonucleotide polymerase chain reaction [ASO-PCR].
