# Supplementary material for: Transcriptomic profiles and 5-year results from the randomized CLL14 study of venetoclax plus obinutuzumab versus chlorambucil plus obinutuzumab in chronic lymphocytic leukemia
Source: Nat Commun. 2023 Apr 18;14:2147. doi: 10.1038/s41467-023-37648-w (PMC10113251; doi:10.1038/s41467-023-37648-w)
Supplement: Supplementary file 3 — Description of Additional Supplementary Files [file 41467_2023_37648_MOESM3_ESM.pdf]

Description of Additional Supplementary Files

Supplementary Data 1: Differentially expressed genes between patients with MRD+ and MRD6. Based on 404 pre-treatment samples, filtered for logFC > 0.5, pval <0.01.

Supplementary Data 2: Differentially expressed genes between relapse and baseline. Based on 45 relapse and paired pre-treatment samples, filtered for logFC >2 and adjPVal <0.05.
